# Global-, regional- and country-level estimates of the work-related burden of diseases and accidents in 2019

**DOI:** 10.5271/sjweh.4132

**Published:** 2024-03-01

**Authors:** Jukka Takala, Päivi Hämäläinen, Riitta Sauni, Clas-Håkan Nygård, Diana Gagliardi, Subas Neupane

**Affiliations:** 1 International Commission on Occupational Health (ICOH), Monte Porzio Catone (Rome), Italy.; 2 Unit of Health Sciences, Faculty of Social Sciences, Tampere University, Tampere, Finland.; 3 Ministry of Social Affairs and Health, Tampere, Finland.; 4 Occupational Health Services and Occupational Medicine, Tampere University, Tampere, Finland.

**Keywords:** cancer, circulatory disease, death, disability at work, disorder at work, economic loss, global country, global region, hazardous substance, occupational injury, work-related disease

## Abstract

**Objective:**

This study provides the global-, regional- and country-level estimates on the work-related burden of diseases and accidents for 2019, including deaths, disability adjusted life years (DALY) and economic losses.

**Methods:**

Data on occupational illnesses and injuries from international organizations, institutions, and public websites were used. Risk ratios (RR) and population attributable fractions (PAF) for the risk factor-outcome pairs were derived from the literature. Estimated mortality and DALY for a group of seven major diseases covering 120 risk-outcome pairs attributable to work were calculated for 181 countries.

**Results:**

Globally, 2.9 million deaths were attributed to work, with 2.58 million deaths due to work-related diseases and 0.32 million related to occupational injuries. Globally, work-related diseases with a long latency period are increasing, while the number of occupational injuries has decreased. Work-related circulatory diseases were the major cause of 912 000 deaths globally, followed by 843 000 work-related malignant neoplasms. In high-income, American, Eastern European and Western Pacific World Health Organization (WHO) regions, however, work-related malignant neoplasms comprised the biggest disease group. DALY attributable to work were estimated to be 180 million in 2019, with an associated economic loss of 5.8% of global GDP. New estimates of psychosocial factors increased the global loss.

**Conclusions:**

The burden of work-related diseases and injuries increased by 26% from 2.3 million annual deaths in 2014 to 2.9 million in 2019. The DALY attributable to work have also substantially increased from 123 million in 2014 to 180 million in 2019 (47% increase). We found large regional and country variations.

Work covers all productive activities around the world. Without work and it associated outcomes, we would not have resources for functioning societies. The global gross domestic product (GDP) is created by work and the workforce. These resources make it possible to have education systems; social services; protection of the young, old and vulnerable parts of the population; food production; housing; and the construction of infrastructure. Work and the workforce are involved in efforts to achieve practically all the United Nations 17 Sustainable Development Goals.

Employment can be both beneficial and harmful for health, depending on the form and quality of job, work exposures and working conditions, even though it is widely acknowledged that a poor work environment is associated with poor health outcomes (1, 2).

This paper is a comprehensive overview and an update to the global estimates of occupational injuries and work-related diseases made by the International Labour Organization (ILO) (3) over the last 25 years in collaboration with the World Health Organization (WHO) and the International Commission of Occupational Health (ICOH) with support from the International Social Security Association (ISSA). Such estimates support the zero-harm mindset and vision where hazards and risks at work are preventable and can be anticipated well in advance. They also enable preparedness against unforeseen pandemics and other future negative developments.

The objective of this study was to estimate the magnitude of occupational burden and provide global-, regional-, and country-level estimates covering 181 countries on the work-related burden of occupational accidents and diseases for 2019 in terms of deaths, disability adjusted life years (DALY) and economic loss as a percentage of total GDP.

## Methods

### Overview

We utilized data on employment, mortality rates, statistical reports on occupational burden of disease and injury, reported accidents, surveys on self-reported occupational illnesses and injuries, attributable fractions, and the most recent information from published papers, documents, and electronic data sources of international and regional organizations, in particular the ILO, the WHO, and European Union (EU) institutions, agencies, and public websites.

The disease estimates are based on WHO regional sources (4) and injury rates were obtained from ILOSTAT reports (ilostat.ilo.org). The WHO and ILO data for most countries were used for 2019 while, for a few specific countries, some of the occupational injury rates were used from previous years due to incomplete reporting of national data to ILO. Where the data did not exist, we used proxy data on injury rates from countries from the same region where the working conditions and economy are comparable. We have also presented the work-related cause-specific fatal and non-fatal outcome estimates for previously available years from 1998 to study the trend of work-related diseases.

Data on the fatal injuries were available as per 100 000 employed population in national statistics, or per 1 million working hours which were converted to 100 000 full-time employed.

### Occupational diseases

The ILO has established and updated the list of occupational diseases regularly (5). The list covers 106 disease outcomes, and each disease may have many exposure-outcome pairs. For example, occupational cancer has 21 listed exposure agents, and diseases caused by exposure to chemicals cover 41 agents and substances. These lists are open ended and additional exposures can be covered when new carcinogens at work are identified, such as those recognized by the International Agency for Research on Cancer (IARC). Many ILO and WHO member states use IARC Group 1 (confirmed) and 2A (probable) agents carcinogenic to humans (6). The criteria of IARC, WHO and ILO for the identification and recognition of occupational diseases are based on epidemiological, clinical, and pathological data; occupational background and job analyses; identification and evaluation of occupational risk factors; and the role of other risk factors.

Altogether we covered 120 exposure–outcome pairs, including 5 psychosocial risk factors. The original occupational exposure–outcome pairs for diseases and disorders were established and directly based on an earlier study by Nurminen & Karjalainen (7). Many new studies have reconfirmed Nurminen and Karjalainen’s previous population attributable fractions (PAF). However, some PAF have been updated, for example, those related to communicable diseases, respiratory diseases, and malignant neoplasms. The supplementary material (www.sjweh.fi/article/4132) includes further tables where updated exposure–outcome pairs and PAF are provided based on recent studies. An attributable fraction can be interpreted as “the fraction of a disease or injury which would not have occurred had the exposure factor been non-existent in the population in question” (7). These exposure–outcome pairs are based on a job-exposure matrix (JEM), which provides data on the prevalence of specific work exposure type and level. Causes of death, numbers and proportions of subjects exposed, and risk ratios obtained from the epidemiologic literature were used to estimate the PAF and disease burden.

### Statistical methods

We used several health outcomes identified at work based on the ILO’s criteria and legal instruments, which encompass injuries, diseases, and disorders including both fatal and nonfatal cases. We calculated years of life lost (YLL), years lived with disability (YLD), and their combination as DALY using ILO and WHO data sources directly (8, 9, see supplementary material) or as adjusted by the Institute of Health Metrics and EvaluationGlobal Burden of Disease and Injury Global Burden of Disease and Injury’s global burden of disease and injury (GBD) studies (8). While the GBD process provides regular updates, the most recent data available are for 2019, which covers only a selected group of occupational risks. Therefore, the GBD 2019 data would not be comparable to past ILO estimates and – if directly used – would be a significant underestimation of the scope of the problem. The differences between ILO and GBD 2019 outcomes have been reported elsewhere (9).

### Injuries and diseases

We gathered mortality data from the WHO (for diseases) and the ILO (for injuries), relying on member state reports that follow the previously established ILO methodology (9–11). YLL were calculated from the death records from each country and region, multiplied by the average YLL per death as per the GBD 2019 data for the respective country or region. For YLD, we have used GBD 2019 data, accounting for the severity of both injuries and illnesses. In the case of circulatory diseases, we excluded all deaths for individuals aged 15–29 and those aged >70 years, including only one-third of the deaths. For other diseases, one-third of the deaths were considered for individuals aged 15–29 years (10). To ensure comparability across countries, we adjusted the YLD from GBD data based on injuries and illness obtained from ILO and WHO data. We calculated the YLD and DALY estimates for each country, region, and the world. To account for the limited coverage of occupational risks in GBD 2019, we developed an adjustment process to align the estimates with a broader range of occupational risks:

YLD_GBD2019_adj_ was adjusted multiplied by the factor obtained by the ratio of YLL_ILOmethod2019_/ YLL_GBD2019_ as follows:


$$YLD_{GBD2019\_adj}\;\times \;\frac{YLL_{ILOmethod2019}}{YLL_{GBD2019}}$$

The estimated number of fatal diseases covered a broader range of occupational risks. When there were more fatal cases, we also anticipated an increase in the number of non-fatal cases, both for injuries and illnesses:


$$YLD_{GBD2019injuries\_adj}\;\times \;\frac{YLL_{ILOmethod2019injuries}}{YLL_{GBD2019injuries}}$$

$$YLD_{GBD2019diseases\_adj}\;\times \;\frac{YLL_{ILOmethod2019diseases}}{YLL_{GBD2019diseases}}$$

$$DALY_{i}=YLL_{i}+YLD_{i}$$

Where, i refers to different categories of diseases.

We calculated the costs in monetary terms by linking the number of DALY for each country and region with the respective GDP for the same country or region. To express these costs as a percentage of GDP, we divided the DALY value for each country or region by total employment. This represents the share of the maximum hypothetical number of years that could have been achieved if no one died or experienced temporary/permanent disability. This figure was derived from the number of people employed under full employment conditions (12).

To assess the work-relatedness of these health outcomes, we used PAF. Detailed information on the attributable fractions and general methodology are available elsewhere (10, 13). The PAF values were sourced from existing literature (7, 14, 15), primarily relying on scientifically reported exposure conditions and JEM from high-income countries and limited data from low-income countries, see the suupplementary material. Specifically, for chronic obstructive pulmonary disease (COPD) and asthma, we utilized PAF values from Driscoll et al's study (16), while for neoplasms, we drew from the study of Rushton et al (15). In the case of colorectal, skin, prostate and other unspecified neoplasms, we used PAF values from Morrell et al (17). It is worth noting that, in a previous comparison of various estimation methods, we argued that the method employed in this study provides the most comprehensive estimates (16).

### Estimation of fatal outcomes

Typically, the denominator used to calculate the fatal injury rates is the number of employed persons. However, in our case covering also disease estimates, we have used the full labor force or data on the total population by age groups. This is because, in many low-income countries, the number of people formally employed is only a small fraction of those who work as self-employed, subsistence farmers, and individuals working in the informal sector, who are often not included in the statistics.

### Fatal occupational injuries

We estimated the number of fatal occupational injuries based on the latest fatality rates, calculated as fatalities per 100 000 workers, from selected ILO member states that reported injury data in three economic sectors: (i) agriculture including farming, fishing, and forestry; (ii) industry including mining, manufacturing, energy production and construction; and (iii) services.

The results are presented for individual countries and regions, using the WHO regions: African region (AFRO), American region (AMRO), Eastern Mediterranean region (EMRO), European region (EURO), South-East Asia region (SEARO) and Western-Pacific region (WPRO). In addition, we grouped relevant countries into a separate high-income region (HIGH) based on the World Bank classification for 2019.

Data on the labor force in each economic sector for each country were obtained from the Central Intelligence Agency’s (CIA) World Factbook. It’s worth noting that these values are also available in the ILOSTAT database, but they are presented as percentages of total employment. In our analysis, we considered both the labor force and total employment, which includes paid and self-employment. These values were then used to calculate the fatality rates and the number of fatalities by economic sector for each country and region.

### Non-fatal occupational injuries

To estimate non-fatal occupational injuries (those causing ≥4 days of absence from work), which are often underreported to ILOSTAT by most countries, we used an average ratio of fatal-to-non-fatal injuries, considering both lower and upper limit estimates (10). The ratio average of 14 EU member states (Belgium, Denmark, Germany, Finland, France, Ireland, Italy, Luxembourg, The Netherlands, Austria, Portugal, Spain, the United Kingdom and Sweden) – 0.11– was used to calculate the lower limit value. Similarly, the corresponding average ratio from Finland, France and Germany – 0.06 – was used to calculate the upper limit value. To estimate the lower and upper limits for each country in 2019, we employed the following method:

Estimated number of non fatal injuries (Lower Limit)=(No.of fatalities ×100)/0.11

Estimated number of non fatal injuries (Upper Limit) = (No.of fatalities×100)/0.06

For the EU's full 28 member states, the EUROSTAT reported data was considered more reliable and used instead of ILOSTAT basic data.

### Fatal work-related diseases

WHO statistics provide comprehensive data covering all diseases globally. These diseases are categorized into seven groups: communicable diseases, malignant neoplasms, neuropsychiatric conditions, circulatory diseases, respiratory diseases, digestive diseases, and genitourinary diseases. Fatal work-related diseases were estimated by PAF (7, 9, 10) for males and females separately. The PAF for these seven disease groups were used to calculate fatal work-related diseases, as presented in the supplementary material, tables S1 and S2. The PAF for communicable diseases were initially based on the HIGH region and later adjusted for non-HIGH regions (9–11).

In 2016, the Collegium Ramazzini released its 19^th^ Statement on COPD in occupational settings (18). As a result, new PAF for COPD were used to estimate deaths caused by chronic respiratory diseases. Regional estimates were calculated based on the WHO regional mortality data, using specific PAF for each region (see the supplementary material). The number of fatal work-related diseases of each country was determined by multiplying the WHO region’s estimate by the country’s labor force proportion. The country data is just an approximate estimate, especially for low-income countries.

In calculating the number of deaths resulting from exposure to hazardous substances in the workplace, we utilized specific attributable fractions for chemical exposure to ensure comparability with previous estimates (10, 15, 17). Additionally, we calculated 95% confidence intervals (CI) around the estimates of fatal work-related diseases in all regions.

### Disability adjusted life years and cost estimates

The methods for calculating DALY are explained above. These calculations are based on adjusted GBD estimates. This adjustment is necessary because GBD data does not cover all occupational risks, in order to minimize underestimation (19). We derived DALY calculations from the estimated number of deaths within major disease groups. First, we obtained YLL and YLD from the GBD data sources, which were then adjusted based on new mortality data from our study.

For musculoskeletal disorders, we directly used DALY data from the GBD source, as ILO and WHO data on musculoskeletal disorders using the previous methodology were unavailable. We incorporated psychosocial factors contributing to mental disorders and cardiovascular diseases based on the European Survey of Workplace Risk Prevalence, assessed using specific indicators (20). The PAF for 35 European countries (including the 28 EU member states) were obtained from a referenced study (20), which relied on the comprehensive regular European survey. We extrapolated these PAF values for use in other countries worldwide. In 2015, we calculated the fractions covering risk factors for cardiovascular diseases and mental disorders resulting from five psychosocial work exposures: job strain, effort–reward imbalance, job insecurity, long working hours, and bullying, for each individual exposure and for all countries collectively (20).

The economic costs associated with DALY were determined using the method described above. These calculations focused exclusively on the economic losses stemming from work-related DALY, without considering any other expenses. The resulting percentages were then expressed as a reduction in the GDP, measured in monetary terms (12).

## Results

### Mortality

**Figure 1:**
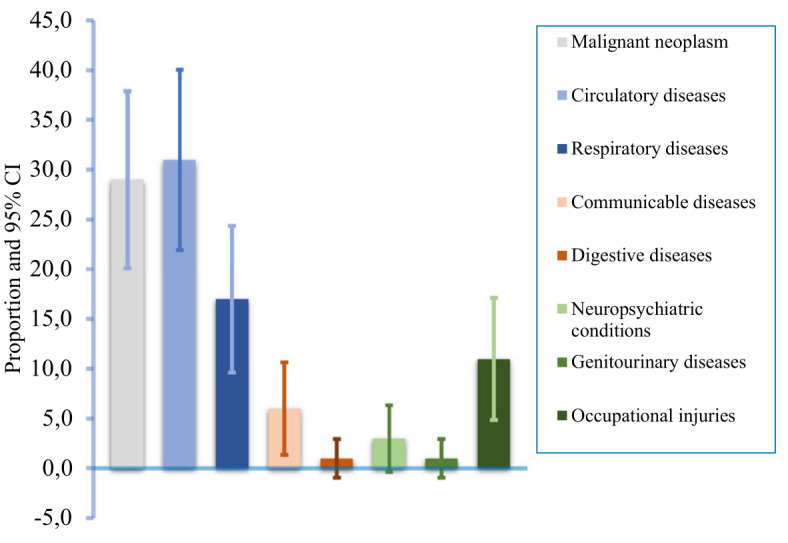
The proportion of disease specific work-related mortality with their 95% confidence intervals (CI) in 2019, globally.

Globally, an estimated 2.90 million work-related deaths occurred in 2019, increased from 2.78 million death from 2015 (table 1, figure 1). About, one-third of the total work-related deaths (31%) were due to circulatory diseases, while malignant neoplasm contributed 29%, respiratory diseases 17%, and occupational injuries contributed 11%. Other diseases such as work-related communicable diseases contributed 6%, while neuropsychiatric conditions contributed 3% and work-related digestive disease and genitourinary diseases contributed 1% each. The breakdown of the estimated malignant neoplasm death and other diseases attributed to hazardous substances is presented in tables 2a and 2b. The contribution of malignant neoplasms and circulatory diseases to total work-related deaths increased from 2015, while deaths due to occupational injuries decreased. Although work-related injury deaths and non-fatal injuries rates were on a decreasing trend, the total deaths and non-fatal outcomes were on the rise (supplementary figures S1a and S1b). Full global, regional, and country data and results are available in the supplementary material, which also includes the division of countries within each WHO region and the HIGH region, extracted separately from WHO regions.

**Table 1:** Cause-specific work-related deaths in 2019 by region in absolute figures and percentages as bracketed and compared with world data in 2015.

Causes of death	HIGH		AFRO		AMRO		EMRO		EURO		SEARO		WPRO		WORLD 2019		WORLD 2015
	N (%)		N (%)		N (%)		N (%)		N (%)		N (%)		N (%)		N (%)		N (%)
Communicable diseases	9718 (1.9)		55 390 (21.1)		6623 (3.5)		12 516 (7.4)		2562 (1.3)		88 153 (11.6)		10 793 (1.3)		185 755 (6.4)		229 983 (8.3)
Malignant neoplasms	234 348 (45.2)		36 130 (13.7)		64 901 (34.0)		29 951 (17.6)		74 600 (38.6)		120 204 (15.8)		282 697 (34.9)		842 830 (29.0)		742 235 (26.7)
Neuropsychiatric conditions	40 059 (7.7)		2979 (1.1)		11474 (6.0)		3081 (1.8)		9841 (5.1)		12759 (1.7)		19 201 (2.4)		99 394 (3.4)		48 116 (1.7)
Circulatory diseases	135 202 (26.1)		74 692 (28.4)		53 941 (28.3)		77 415 (45.6)		70 822 (36.6)		250 901 (33.1)		249 749 (30.8)		912 723 (31.4)		863 173 (30.9)
Respiratory diseases	73 699 (14.2)		23 407 (8.9)		28 609 (15.0)		24 638 (14.5)		19 193 (9.9)		186 306 (24.5)		142 637 (17.6)		498 490 (17.2)		475 589 (17.0)
Digestive diseases	3591 (0.7)		4189 (1.6)		2249 (1.2)		1886 (1.1)		1696 (0.9)		8879 (1.2)		3820 (0.5)		26 310 (0.9)		25 914 (0.9)
Genitourinary diseases	5690 (1.1)		2501 (1.0)		3241 (1.7)		2173 (1.3)		1342 (0.7)		7587 (1.0)		5609 (0.7)		28 143 (1.0)		18 955 (0.7)
Occupational injuries	16 342 (3.2)		63 857 (24.3)		19 745 (10.4)		18 147 (10.7)		13 231 (6.9)		84 414 (11.1)		96 313 (11.9)		312 050 (10.7)		380 500 (13.7)
Total	518 649		263 145		190 784		169 807		193 288		759 203		810 818		2 905 694		2 784 465

**Table 2a:** Deaths attributed to hazardous substances including dusts, vapours and fumes, for men and women in regions in 2019. [(HIGH=high-income group, AFRO=Africa region, AMRO=America region, EMRO=Eastern Mediterranean region, ERO=European region, SEAR=South-East Asia region, and WPRO=Western Pacific region.]

Cause of deaths		AFRO			AMRO			EMRO			EURO			SEARO			WPRO	
		Men	Women		Men	Women		Men	Women		Men	Women		Men	Women		Men	Women
Malignant neoplasms		10 237	4804		47 876	14 104		13 541	3812		91 330	18 292		45 271	12 327		167 511	25 696
	Mouth & oropharynx	108	35		220	42		151	41		431	66		1 236	270		590	99
	Oesophagus cancer	441	115		987	97		307	75		1 267	142		1 472	270		6726	653
	Stomach cancer	367	32		1281	86		452	27		2282	146		1675	147		10009	436
	Colon rectum cancers	156	78		680	326		142	57		1 384	603		601	285		2078	689
	Liver cancer	44	12		77	26		52	12		105	29		122	26		386	71
	Pancreas cancer	1	1		8	4		1	0		14	7		5	2		19	7
	Trachea, bronchus, lung	3131	390		30 134	5989		6806	485		63479	6372		25 404	2923		126 295	14 117
	Melanoma & skin	654	113		1809	216		342	44		2 177	352		767	89		1398	253
	Breast cancer	0	2577		0	4 971		0	2265		0	7251		0	5709		0	6175
	Cervix uteri cancer	0	530		0	269		0	78		0	211		0	570		0	455
	Ovary cancer	0	64		0	147		0	52		0	238		0	182		0	199
	Prostate cancer	349	0		975	0		134	0		1218	0		425	0		792	0
	Bladder cancer	367	72		1723	192		822	60		3670	308		1163	113		2876	244
	Leukaemia	67	35		256	113		85	36		333	152		228	98		373	153
	Other malignant neoplasms	4551	751		9 727	1624		4 247	579		14970	2414		12171	1641		15971	2144
Neuropsychiatric conditions		395	210		2089	3176		418	436		2 507	4110		1456	1752		2329	3421
Circulatory diseases		3030	4272		4739	3501		4619	3369		8680	7062		13242	8988		14989	9587
Respiratory diseases		16 856	6592		35 650	12983		22 758	5596		36 528	10 170		138 983	47 323		128 260	31 693
	COPD	11 534	2566		33 987	11338		17 387	2751		34 740	8365		114 387	28 579		121 875	28 403
	Asthma	5258	3992		945	957		5305	2810		1216	1278		24 138	18 380		5817	2928
	Other respiratory diseases	64	34		718	688		66	35		571	528		459	363		568	362
Genitourinary diseases		753	613		1620	1625		735	738		944	1078		2 284	1836		1860	1776
TOTAL		31 272	16 491		91 974	35 389		42 072	13 951		139 990	40 713		201 236	72 226		314 949	72 174

**Table 2b:** Deaths attributed to hazardous substances including dusts, vapors and fumes, for men and women in the world in 2019.

Cause of death		WORLD		
		Men	Women	Total
Malignant neoplasms ^a, b^		375 767	79 035	454 802
	Mouth & oropharynx	2737	554	3291
	Oesophagus cancer	11 200	1352	12 552
	Stomach cancer	16 066	875	16 941
	Colon rectum cancers	5041	2039	7080
	Liver cancer	786	178	964
	Pancreas cancer	48	21	68
	Trachea, bronchus, lung	255 248	30 276	285 524
	Melanoma & skin	7146	1067	8213
	Breast cancer	0	28 949	28 949
	Cervix uteri cancer	0	2113	2113
	Ovary cancer	0	882	882
	Prostate cancer	3893	0	3893
	Bladder cancer	10 621	990	11 611
	Leukaemia	1342	587	1930
	Other malignant neoplasms	61 639	9153	70 791
Neuropsychiatric conditions		9194	13 105	22 299
Circulatory diseases ^c^		49 299	36 779	86 078
Respiratory diseases		379 035	114 357	493 392
	COPD ^d^	333 909	82 001	415 911
	Asthma ^d^	42 680	30 346	73 025
	Other respiratory diseases ^e^	2446	2010	4456
Genitourinary diseases		8197	7667	15 864
TOTAL		821 492	250 944	1 072 436

^a^ The attributable fractions of cancers were taken from the article of Rushton et al (15). ^b^ The attributable fractions of colorectal, skin, prostate, and other unspecified cancers were taken from Morrell et al (17), see further references in (16). ^c^ All deaths for people 15-29 were excluded and for over 70 years old, only one third of deaths were included. ^d^ Attributable fraction was used from Driscoll et al (16). ^e^ One third of the figures were considered for those aged 15-29 in global burden of disease (16).

**Figure 2:**
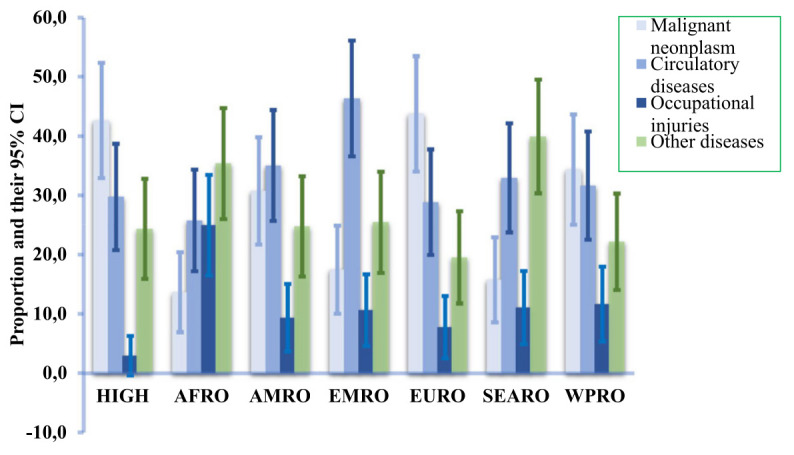
Work-related estimated diseases-specific mortality per 100 000 employed people with their 95% confidence intervals (CI) in 2019 by regions. See countries included in the regions in the supplementary material. (HIGH=high-income group, AFRO=Africa region, AMRO=America region, EMRO=Eastern Mediterranean region, ERO=European region, SEAR=South-East Asia region, and WPRO=Western Pacific region).

The contribution of diseases to total work-related deaths varies across regions (figure 2). In the HIGH region, malignant neoplasms accounted for the highest percentage (43%), followed by circulatory diseases (30%), other diseases (24%), and occupational injuries 3%. Malignant neoplasms (cancers) represented the most significant cause of mortality in HIGH countries, as well as in the EURO and WPRO regions. The group of “other diseases” had a greater impact in the AFRO and SEARO regions due to higher mortality associated with communicable and respiratory diseases attributed to work in these regions. It’s worth noting that AFRO and SEARO are densely populated developing regions.

### Disability adjusted life years

**Figure 3:**
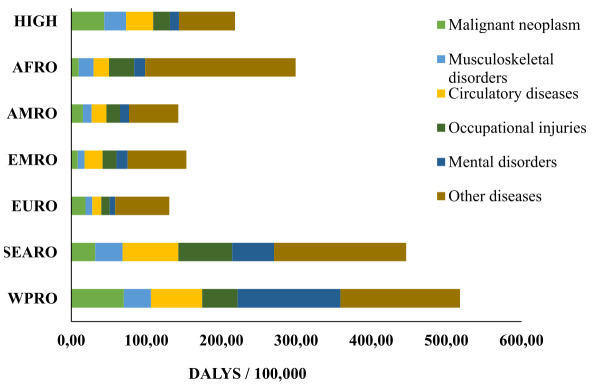
Cause specific work-related disability adjusted life years (DALYs) by regions in the year 2019. (HIGH=high-income group, AFRO=Africa region, AMRO=America region, EMRO=Eastern Mediterranean region, ERO=European region, SEARO=South-East Asia region, and WPRO=Western Pacific region).

In 2019, globally, the DALY attributed to work were 180 million (figure 3). The primary causes of DALY were circulatory diseases (14%), occupational injuries (13%), malignant neoplasm (11%), musculoskeletal disorders (9%), mental disorders (8%), and other diseases (46%). A major part of work-related diseases and disorders within the “other diseases” group consisted of respiratory diseases and communicable diseases, which are prevalent globally. The cause specific DALY also showed regional variations (figure 3 and world maps in the supplementary material).

### Economic loss

The total economic loss resulting from work-related DALY amounted to 6% of the global GDP, with variation among countries and regions (supplementary figure S3). The highest economic loss was 9% of GDP in EMRO, 8% in AFRO, 8% in EURO, 7% in SEARO, 5% in AMRO, 5% in WPRO, and the lowest in the HIGH region (4%). It’s worth noting that the HIGH region significantly influences the absolute monetary loss due to its high GDP.

## Discussion

The work-related burden was estimated as 2.9 million deaths in 2019 globally, with circulatory diseases as the largest contributor (31%), followed by malignant neoplasms (29%). Deaths due to occupational injuries contributed 11% of the total work-related deaths. Additionally, there were 180 million DALY attributable to work in 2019 globally. We observed regional variations in the burden of work-related diseases, with malignant neoplasms being the leading cause of death in the HIGH region, while circulatory diseases topped the list in low-income regions.

The global work-related death burden increased by 4% between 2015 and 2019, rising from 2.78 million to 2.9 million deaths (table 1). However, deaths due to occupational injuries decreased by 22%. Similarly, DALY attributable to work increased by 47% in the same period, from 123 million in 2015 to 180 million in 2019 (11, 12, 19). One of the reasons for this increment could be due to inclusion of psychosocial factors in the total estimates, which were inadequately covered in previous estimates. In 2019, there were 2.59 million deaths due to fatal work-related illnesses, marking an increase of 0.2 million compared to 2015 estimates (8, 12, 19, see supplementary material). Globally, it is estimated there are nearly 8000 work-related deaths every day: 855 from occupational injuries and 7100 from work-related diseases. The number of non-fatal occupational injuries was estimated to be 402 million, significantly higher than in 2015. This increase can be partially attributed to a more accurate estimate used in the previous assessments compared to 2019.

The decrease in deaths due to occupational injuries when compared to previous years (2011, and 2015) (11) maybe the result of applying a lower fatal accident rate in highly populated Asian regions. It’s important to note that relatively incomplete reporting of fatal accident still persists in most parts of the world, potentially contributing to this decrease rather than indicating a genuine improvement in accident prevention. Additionally, many countries have been slow in reporting their data, meaning that 2019 data may become more complete in the future. Furthermore, fluctuations in reporting can be attributed to lower employment data in highly populated areas worldwide, including China and India leading to fewer reported deaths.

A total decline in employment was observed in three WHO regions: EURO, SEARO and WPRO compared to earlier years (data from 2011 and 2015) (9, 11). This decline also had an impact on reducing the number of fatal occupational injuries along with the lower rate per 100 000 workers. The lower rate is likely the result of favorable trends in developed countries, particularly in the high-income sector, as well as the use of better reporting proxy country rates for countries with no available data, such as Malaysia as a proxy for some other SEARO countries. As a result, the estimated number of fatal occupational injuries decreased from 380 500 to 312 000. However, we observed an increase in fatal occupational injuries in HIGH countries, rising from 10 757 to 16 342. These trends were likely due to better reporting systems in these countries, and more countries being listed under the HIGH group than in earlier periods. In the HIGH group, there is a significant subgroup that includes relatively new EU member states, such as Estonia, Latvia, Lithuania, Romania, Bulgaria, Slovenia, in addition to those countries that were already part of the HIGH group in earlier periods. In other regions, aside from SEARO and WPRO, there were no significant changes in the occurrence of occupational injuries.

COPD is caused by exposures to a multitude of vapors, gases, dusts and fumes. These exposures pose serious risks in workplaces and are often under-regulated. COPD is known to be associated with exposure to coal mine dust, asbestos, silica, welding and cutting gases and fumes, cement dust, diesel exhausts, spray painting, organic solvents, and possibly man-made mineral fibers (18). It is estimated that occupational exposures were responsible for 15–20% of COPD cases, making them a significant contributor to COPD (21). Deaths due to COPD constituted more than 80% of all chronic respiratory disease estimates (22).

In cause-specific mortality, the largest increases in mortality from 2015 (9, 11) were seen in circulatory diseases, followed by cancer and respiratory diseases, while work-related communicable diseases declined. This has increased in the years since 2019 when COVID-19 was not yet visible in data sources.

### Comparison with previous literature

Our estimates are mostly in line with previous global estimates, despite methodological differences in the exposure estimates and the coverage of the occupational exposures. An earlier study based on GBD data shows 1.53 million DALY (95% CI 1.39–1.68) attributable to occupational risk factors, accounting for 2.8% of deaths and 3.2% of DALY from all cause (23). The disability adjustments of the DALY concept are based on a global average individual perception of disease outcomes, which usually differs from occupational disability established by national employment or injury compensation systems. The GBD data considers that if someone is unable to move, it is equal to about 40% disability of full life ability (8), while in most cases, it could be close to 100% disability to work. The highest total burden (as determined by DALY) was brought on by ergonomic exposures related to low back disorders, injury risk factors, and noise. The GBD study covered only a limited number of exposure–outcome pairs; therefore, the GBD results underestimate the occupational burden of diseases and injuries. The WHO released data on WHO/ILO estimates based on 19 specifically estimated exposure–outcome pairs, showing that extensive working hours, >55 hours a week, resulted in 745 000 cardiovascular deaths (24). This is just one exposure–outcome pair. Our study estimated that there were 912 000 circulatory deaths globally while considering shift and night work, strain/ischemic heart disease (IHD), engine exhaust, carbon monoxide/IHD, noise at work, traffic noise/IHD, environmental tobacco smoke at work/IHD, shift work and strain/cerebrovascular diseases, environmental smoke at work/cerebrovascular disease. Additional causative agent of death in our study were carcinogens, particulate exposures, gases and fumes, as well as injury risk factors.

Occupational cancer data from WHO published estimates, as well as data from GBD, only consider IARC Group 1 carcinogens and cover a limited number of chemical agents as causes. This resulted in 291 661 deaths in 2016, of which 209 481 were caused by asbestos. The GBD estimate for occupational cancer was 314 980, with 229 402 deaths attributed to asbestos in 2016. According to 2019 data from GBD (23), there were 333 867 deaths for all selected pairs, with 239 334 deaths linked to asbestos.

Our data in this study showed 843 000 occupational cancer deaths based on a wider dataset comprising 85 exposure–outcome pairs of both confirmed human carcinogens (IARC Group 1) and probable human carcinogens (IARC Group 2A) in 2019. The latest asbestos estimates, based on mesothelioma deaths, were estimated at 35 087, indicating a clear underestimation of asbestos-related deaths by GBD (25, 26). Asbestos exposure is strongly linked to mesothelioma incidence and mortality, and mesothelioma deaths may be used as a proxy for asbestos exposure. This indicates a higher number of other diseases caused by asbestos. As a result, a more realistic estimate of asbestos-related deaths could be of 289 621 in the workplace, and 304 841 when including environmental and semi-occupational causalities (9). This highlights the importance of the selection criteria for exposure–outcome pairs, a limited number of such pairs results in limited outcomes.

The selection of fewer exposure–outcome pairs, as seen in GBD (23), conflicts with the greater number of diseases and disorders covered by the ILO’s list of occupational diseases. The ILO list was developed by a Committee of Experts nominated by governments, employers’ organization, and workers’ organizations, and it was adopted by the ILO Governing Body (5).

To illustrate the difference in coverage of exposures–outcomes, consider a recent study conducted by the Swedish Work Environment Authority (27). This national study estimated that there were 3200–5500 work-related deaths in Sweden in 2019, taking into account the associations between the risks and outcomes but with less conclusive evidence for the higher number. In comparison, the ILO estimate for Sweden was 4200 deaths in 2017 (27), and when applying the same ILO methodology in this study, it was 4443 in 2019. The WHO’s published data for Sweden in 2016 showed 1856 work-related deaths, which clearly reflects the limited number of exposure–outcome pairs (27). According to Sweden’s own statistics, there were 37 fatal occupational injuries in 2016, while the GBD estimate put it at 95 deaths; our estimate was 35 in 2019, based on data from earlier years.

The global estimate for occupational injury-related deaths by GBD was 311 492 in 2019, with some data coming from earlier years, and among them, 61 780 deaths occurred in China (23). However, China’s own statistics reported 69 434 deaths related to occupational injuries in 2015. Our estimate for global occupational injuries related deaths was 310 615, of which 82 305 occurred in China when considering all sectors, including agriculture (8, 10, 28), see the supplementary material for details.

### Limitations

We utilized data from WHO and ILO for the year 2019, although some of the occupational injury rates used for estimations are from previous years due to incomplete reporting of national data to the ILO. This could introduce bias, particularly because many developing countries do not report their injury data. Additionally, the regional averages may disproportionately highlight countries with better reporting, resulting in an under-estimation of occupational injury rates on a global scale.

The availability and quality of source data used to support the research serve as further limitations on our estimations of the attributable burden. There is a lack of information regarding the associations between several occupational exposures and health outcomes. Moreover, when it comes to exposure measurement, data availability patterns vary over time and between different geographical areas. Even when available, these data may rely on poorly comparable methods, such as self-reporting. The exposures measurement from multiple exposure–outcome pairs were used to calculate the attributable fractions. While, we have estimated the CI, it’s important to note that most available research and statistics provide more comprehensive coverage for HIGH countries yielding more accurate data. In contrast, less developed regions and individual country estimates within those regions are likely to be less accurate and potentially under-estimated. This limitation may result in more systematic and substantial variations in results compared to the calculated CI. Furthermore, individual country source data could have influenced regional outcomes.

### Concluding remarks

Globally, 2.9 million work-related deaths occurred in 2019 which represents a 26% increase from 2015. DALY attributable to work were 180 million globally, an increase of 47% from 2015 to 2019. Regional variations in the burden of work-related diseases were found. Estimated fatal occupational injuries declined in 2019 compared to 2015. The economic loss from the work-related diseases was 5.8% of the global GDP. For the first time these estimates also cover psychosocial factors, which increased the global loss from past estimates.

We covered 120 occupational exposures–outcome pairs in our estimates. It is important to explore a broad picture of the exposures–outcome for policy decisions and to set priorities for action at workplace level. Given the 106 categories of the diseases in the ILO list of occupational diseases and the multiplied number of risk-exposure pairs, it is evident that the real estimates resulting from these pairs are of high magnitude. Evidence of many further factors has already been considered by several governments in high-income countries, while the majority of UN member states have so far shown limited progress in prevention and preparedness.

## Supplementary material

Supplementary material files 1 and 2

Global, regional and country data results in Excel
