# Supplementary material for: Global-, regional- and country-level estimates of the work-related burden of diseases and accidents in 2019
Source: Scand J Work Environ Health. 2024 Feb 28;50(2):73–82. doi: 10.5271/sjweh.4132 (PMC10927068; doi:10.5271/sjweh.4132)
Supplement: Supplementary material files 1 and 2 [file SJWEH-50-73-S001.pdf]

Global-, regional- and country-level estimates of the work-related burden of diseases and accidents in 2019[1]

by Jukka Takala, DSC,[2] Päivi Hämäläinen, PhD, Riitta Sauni, MD, PhD, Clas-Håkan Nygård, PhD, Diana Gagliardi, MD, PhD, Subas Neupane, PhD

1. *Supplementary material*
2. *Correspondence to: Jukka Takala, Unit of Health Sciences, Faculty of Social Sciences, Tampere University, Tampere, Finland.*
   [E-mail: jstakala@gmail.com and jukka.takala@tuni.fi]

Supplementary Annex . Additional Tables and Figures and Updated population attributable fractions and related references. Pages 1 - 21.

Supplementary Data Table covering consolidated global, regional and country data on work-related diseases, injuries and calculation methods. Excel formatted data converted to delimited pdf-file. Pages 22 - 119

Supplement, Table S1. Comparison of population attributable fractions of diseases due to workplace exposure not necessarily classified as occupational diseases [10]

	Attributable fraction									
	Nurminen and Karjalainen (2001)		Rushton et al. (2008) [c]		Steenland et al. (2003) [d]		Morrel at al. (1998)[a]		Leigh at al. (1997)[b]	
**Causes**	**Men**	**Women**	**Men**	**Women**	**Men**	**Women**	**Men**	**Women**	**Men**	**Women**
Communicable diseases	4.8	32.5								
Cances	13.8	2.2	8.0	1.5	3.3-7.3	0.8-1.0			6-10	
Respiratory diseases	6.8	1.1							10[c]	
Circulatory diseases	14.4	6.7			6.3		1.0	1.0	5-10	
Mental health disorders	6.6	1.8				1.0	1.0		1-3	
Digestive diseases	2.3	1.5								
Genitourinary system	3.0	0.4				1.0	1.0		1-3	

a Cover only deaths due to occupational exposure to hazardous substances
b Pneumoconiosis are not included in the figure of Leigh at al.
c The table and reference above are from Hämäläinen [10] of 2010, updated newer ref. by Rushton [15] of 2012,
d The table and reference above are from Hämäläinen [10] of 2010, updated newer ref. by Steenland [14] of 2011,

Note:
There are two different documents by both Rushton (2008 & 2012), and by Steenland (2003 & 2011).
See reference list below after "Supplement , Table S3" for later references, and Hämäläinen (2010) for earlier ones.

Supplement, Table S2: Population attributable fractions (PAF) used in the estimation of fatal work-related illnesses

**Causes of Death**	**High-income region†**		**All other regions‡**	
	**Men**	**Women**	**Men**	**Women**
Communicable diseases	4.80	32.50	3.05	20.68
Malignant neoplasms	13.80	2.20	13.80	2.20
**Neuropsychiatric conditions**	6.60	1.80	6.60	1.80
**Circulatory diseases**	14.40	6.70	14.40	6.70
**Respiratory diseases**				
**COPD**	18.00	6.00	18.00	6.00
**Asthma**	21.00	13.00	21.00	13.00
**Others**	1.00	1.00	1.00	1.00
**Digestive diseases**	2.30	1.50	2.30	1.50
Genitourinary system diseases	3.00	0.40	3.00	0.40

†Attributable fractions from Nurminen & Karjalainen [7]

‡Revised attributable fractions [29]

COPD: Chronic Obstructive Pulmonary Disease

Supplement, Figure S1 a, b: Trend of work-related diseases-specific deaths **a)** rates/100,000 employed and **b)** total deaths (in thousands) from 1998 to 2019.

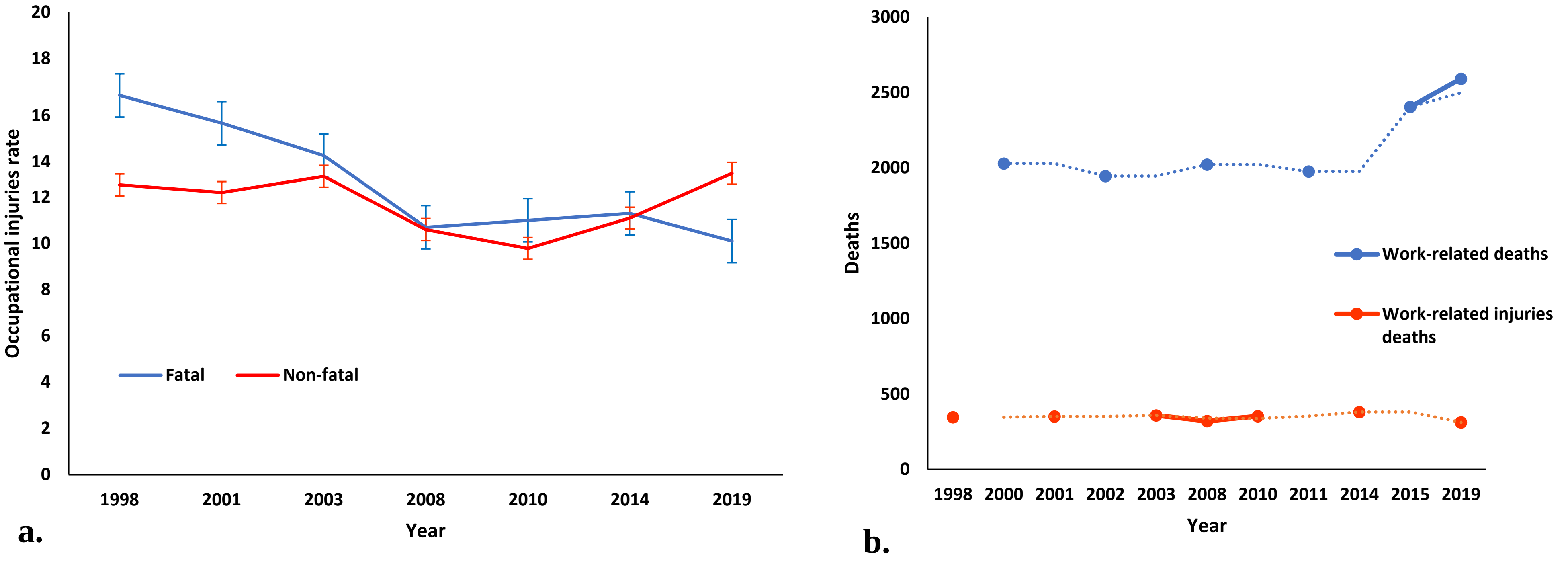

Supplement, Figure S2 a, b: Word-map of the global estimates of a) malignant neoplasm and b) circulatory deaths/100,000 employed.

a.

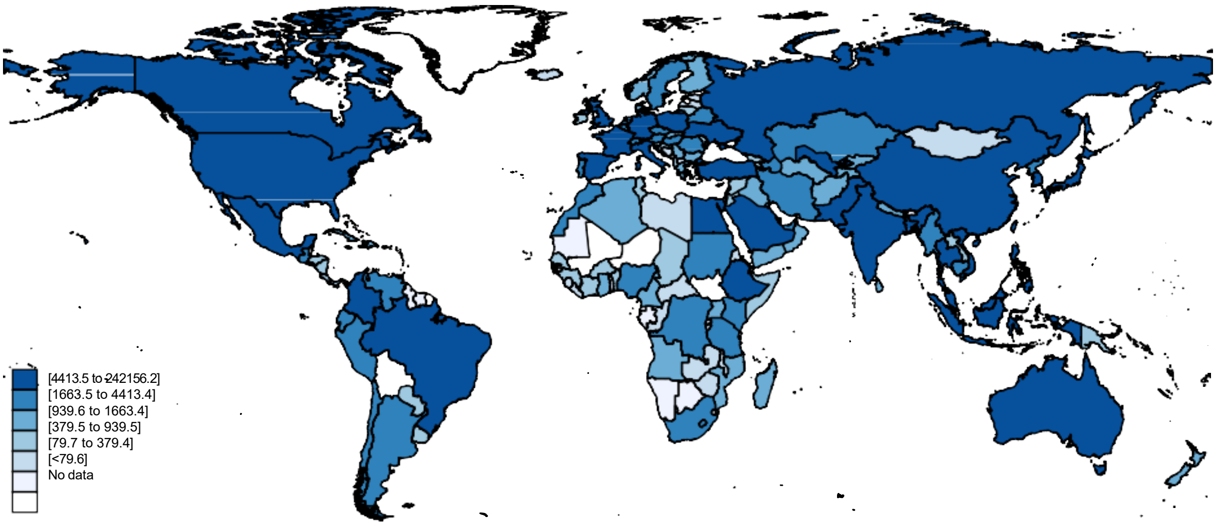

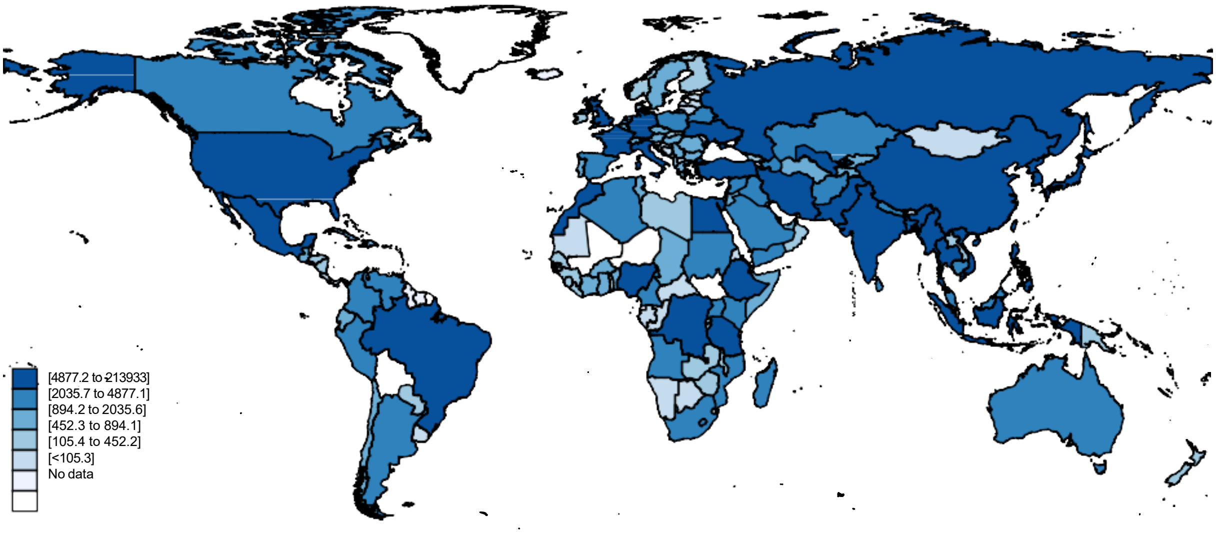

b.

.

Supplement, Figure S3 a, b: Word map of DALYs/100,000 employed for a) malignant neoplasm and b) circulatory diseases.

a.

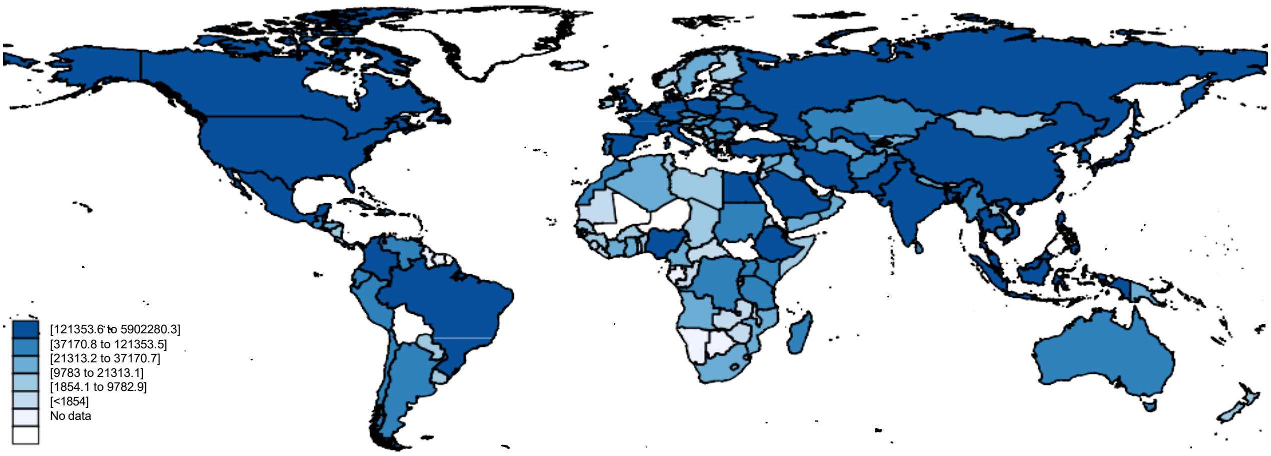

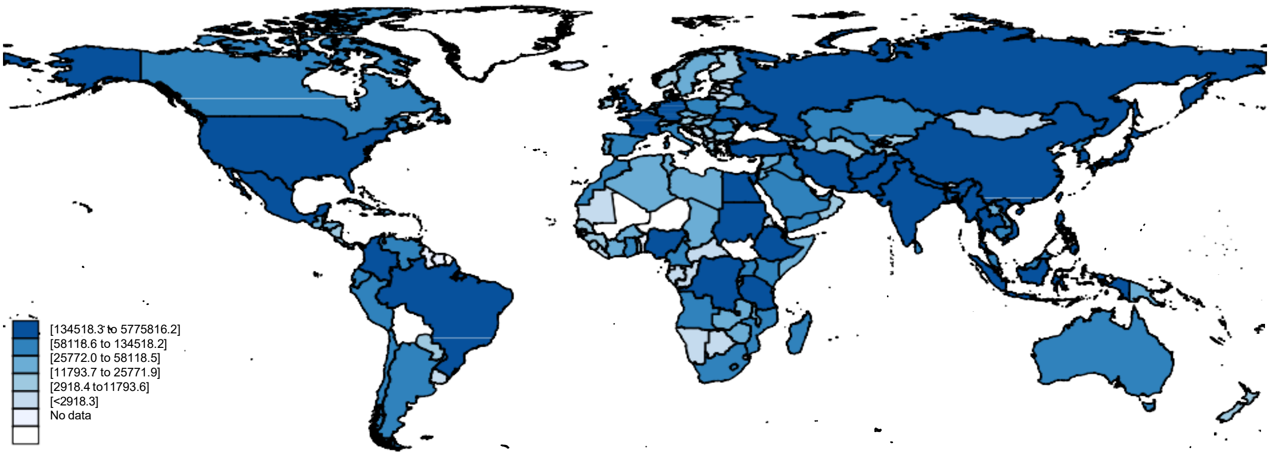

b.

Supplement, Figure S4: World map showing the annual economic loss in terms of percentage of GDP.

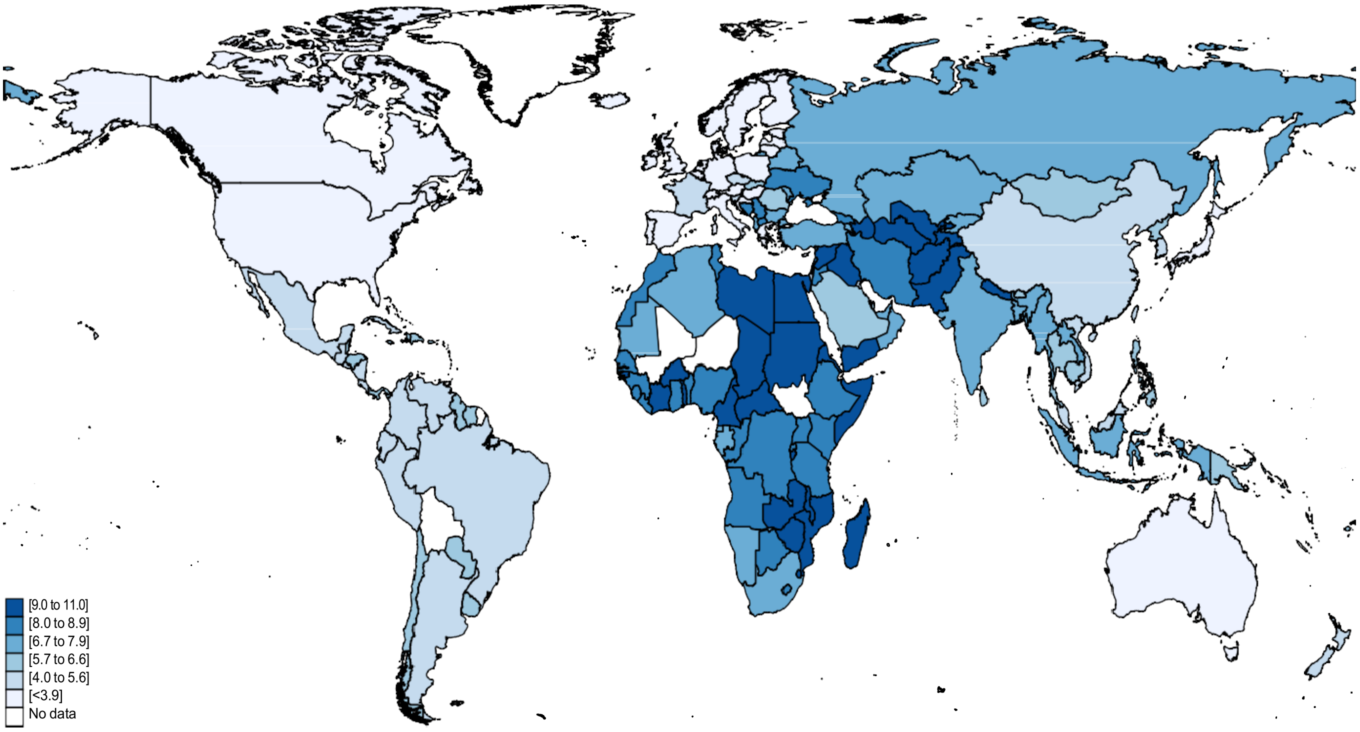

Supplement , Table S 3 . Up-dated AttributableFraction Estimates of Nurminen M. and Karjalainen A. For original paper, see reference nr.7 below.

Note: Updated new references are in italics below and in full at the end of this file. All new references are global.

Cause of death [a]	Attributable fraction [b] (%) Total	Men	Women	Potentially implicated exposure or work environment [c]	Study design and data analysis	Primary source of quantitative estimate [e] *Further updated sources added in italics*
Total deaths (all codes)	6.7	10.2	2.1			
Infectious diseases (all A and B codes)	8.8	4.8	32.5			
Tuberculosis (A15—A18, B90)	6.0	0.6	≈75	Health and social worker (infection)	Based on register data	Karjalainen et al, 1998, Finland (11); National Public Health Institute, 1998, Finland (268); *Alele F, 2019; (31), NIOSH/CDC, 2016 (32)*
Pneumococcal disease (A40.3)	14.3	15.6	10.9	Environmental tobacco smoke	Adjusted for age, gender, race, level of education, chronic illness, study site, and status of children in the houshold	Nuorti et al, 2000, United States (264) *Torén et al. 2020, (33)*
Malignant neoplasms, by site or type (all C codes)	8.4	13.8	2.2			
Oral cavity (C01-C06)	0.8	1.2	0.3	Combined risk factors	·	·
		0.3	0.0	Polycyclic aromatic hydrocarbons (high level of exposure)	Restricted to men; adjusted for age, geographic region, and alcohol and tobacco consumption	Gustavsson et al, 1998, Sweden (317); *Paget-Bailly et al, 2012, (34),*
		0.9	0.3	Hydrocarbon solvents	Restricted to men; adjusted for age and social class	Kauppinen et al, 1998, Finland (14) *Paget-Bailly et al, 2012, (34);*
Pharynx (C09-C14)	1.9	2.0	0.5	Combined risk factors	·	
		1.1	0.2	Welding fumes (metal dust, irritant gases, polycyclic aromatic hydrocarbons)	Restricted to men; adjusted for age, geographical region, and alcohol and tobacco consumption	Gustavsson et al, 1998, Sweden (317), *Awan et al, 2018 (37)*
		0.9	0.3	Hydrocarbon solvents	Restricted to men; adjusted for age and social class	Kauppinen et al, 1998, Finland (14); *Paget-Bailly et al, 2012, (34)*
Esophagus (C15)	3.6	6.4	0.2	Combined risk factors	·	
		6.1	0.1	Polycyclic aromatic hydrocarbons	Restricted to men; adjusted for age, geographical region, and alcohol and tobacco consumption	Gustavsson et al, 1998, Sweden (317), .
		0.3	0.1	Hydrocarbon solvents	Restricted to men; adjusted for age and social class.	Kauppinen et al, 1998, Finland (14) *Jansson et al, 2016, (35)*
Stomach (C16)	8.0	10.3	5.4	Farming and rearing of live stock (grain dust, animal contact, herbicides, diesel fuels)	Restricted to decedents; adjusted for age, gender, cigarette smoking in the 1960s, alcohol consumption in the 1960s, and diabetes	Partanen et al, 1994, Finland (108)
Colon (C18)	2.5	5.6	0.0	Combined risk factors	·	·
		2.3	0.0	Asbestos	Restricted to decedents; adjusted for age, gender, cigarette smoking in the 1960s, alcohol consumption in the1960s, and diabetes	Kauppinen et al, 1995, Finland (109) *Oddone et al, 2014 (36)*
		3.4	0.0	Welding and soldering fumes and gases (polycyclic aromatic hydrocarbons and other combustion products)	Restricted to decedents; adjusted for age, gender, cigarette smoking in the 1960s, alcohol consumption in the1960s, and diabetes	Kauppinen et al, 1995, Finland (109) *Santibanez et al; 2018 (38)*

(continued)

Cause of death [a]	Attributable fraction [b] (%)			Potentially implicated exposure or work environment [c]	Study design and data analysis [d]	Primary source of quantitative estimate [e] *Further updated sources added (in italics)*
	Total	Men	Women			
Rectum (C20)	1.7	3.1	0.1	Combined risk factors	.	.
		2.9	0.0	Asbestos	Restricted to decedents; adjusted for age, gender, cigarette smoking in the 1960s, alcohol consumption in the 1960s, and diabetes	Kauppinen et al, 1995, Finland (109) *Talibova et al. 2018, (39)*
		0.2	0.1	Styrene	Standardized incidence ratios based on age-, gender-, and period-specific incidence rates of cancer in the general population	Anttila et al, 1998, Finland (318)
Liver and intra-hepatic bile ducts (C22)	4.3	3.5	5.3	Combined risk factors	.	.
		3.1	0.3	Inorganic dusts (mainly silica)	Frequency-matched for age and gender; stratified by alcohol consumption; hepatitis B infection and the use of oral contraceptives and other hormone preparations were rare	Kauppinen et al, 1992, Finland (319) *Brandi et al, 2020 (41)* *Farioli et al, 2018 (42)*
		0.0	2.1	Milk maids (aflatoxins from crops used by the livestock feed-processing industry)	Frequency-matched for age and gender; stratified by alcohol consumption; hepatitis B infection and the use of oral contraceptives and other hormone preparations were rare	Kauppinen et al, 1992, Finland (319)
		.	3.0	Chlorinated hydrocarbon solvents	Estimate for women; control for confounders as in Kauppinen et al (319)	Hernberg et al, 1988, Finland (320)
		0.4	.	Chlorinated hydrocarbon solvents	Estimate for men; adjusted for age and social class	Kauppinen et al, 1998, Finland (14) *Kubo et al, 2018 (40)*
Gallbladder (C23)	0.1	0.2	0.4	Hydrocarbon solvents	Restricted to men; adjusted for age and social class	Kauppinen et al, 1998, Finland (14) *Brandi et al, 2020 (41)*
Pancreas (C25)	8.0	13.4	3.5	Combined risk factors	.	.
		.	.	Gardener	Restricted to decedents; adjusted for age, gender, cigarette smoking in the 1960s, alcohol consumption in the 1960s, and diabetes	Partanen et al, 1994, Finland (108)
		.	.	Transport inspectors and supervisors (polycyclic aromatic hydrocarbons in engine exhaust)	Restricted to decedents; adjusted for age, gender, cigarette smoking in the 1960s, alcohol consumption in the 1960s, and diabetes	Partanen et al, 1994, Finland (108)
		.	.	Warehousemen (polycyclic aromatic hydrocarbons)	Restricted to decedents; adjusted for age, gender, cigarette smoking in the 1960s, alcohol consumption in the 1960s, and diabetes	Partanen et al, 1994, Finland (108)
		2.1	0.4	Organic solvents (including aliphatic and aromatic hydrocarbons)	Adjusted for age, gender, cigarette smoking in the 1960s, alcohol consumption in the 1960s, and diabetes	Kauppinen et al, 1995, Finland (109) *Andreotti et al, 2012 (43)* *Bagga et al, 2012 (45)*
		1.5	0.6	Inorganic dust containing crystalline silica	Adjusted for age, gender, cigarette smoking in the 1960s, alcohol consumption in the 1960s, and diabetes	Kauppinen et al, 1995, Finland (109)
		1.4	0.0	Rubber chemicals including acrylonitrile	Adjusted for age, gender, cigarette smoking in the 1960s, alcohol consumption in the 1960s, and diabetes	Kauppinen et al, 1995, Finland (109)
		0.8	1.9	Ionizing radiation	Adjusted for age, gender, cigarette smoking in the 1960s, alcohol consumption in the 1960s, and diabetes	Kauppinen et al, 1995, Finland (109) *Santibañes et al, 2010, (44)*
		2.5	0.3	Pesticides (herbicides and insecticides)	Adjusted for age, gender, cigarette smoking in the 1960s, alcohol consumption in the 1960s, and diabetes	Kauppinen et al, 1995, Finland (109)
		3.1	0.2	Nickel	Risk estimates from a meta-analysis; estimates adjusted for at least known risk factors for pancreatic cancer (age, gender, tobacco smoking, and social class), if there was a choice	Ojajärvi et al, 2000, international (111)

(continued)

Cause of death [a]	Attributable fraction [b] (%)			Potentially implicated exposure or work environment [c]	Study design and data analysis [d]	Primary source of quantitative estimate [e]
	Total	Men	Women			
Pancreas (continued)		2.8	0.1	Polycyclic aromatic hydrocarbons	Risk estimates from a meta-analysis; estimates adjusted for at least known risk factors for pancreatic cancer (age, gender, tobacco smoking, and social class), if there was a choice	Ojajärvi et al, 2000, international (111) *Santibañes et al, 2010, (44)*
Nose and nasal sinuses (C30-C31)	12.5	24.0	6.7	Combined risk factors	.	.
		15.7	2.4	Hardwood dust or softwood dust or both	Matched for country, gender, age at diagnosis, and survival status; confounding by exposure to formaldehyde unlikely	Hernberg et al, 1983, Denmark, Finland, Sweden (261) *Siew S. 2018 (46)*
		2.8	4.4	Leather dust (shoe and boot manufacturing)	Pooled analysis of data from 8 studies; adjusted for age, gender, study center, and smoking status, as well as for the remaining occupational exposures examined	t'Mannetje et al, 1999, France, Germany, Italy, Sweden, The Netherlands (321)
		7.3	0.0	Welding, flame-cutting and soldering (hexavalent chromium, nickel)	Pooled analysis of data from 8 studies; adjusted for age, gender, study center, and smoking status, as well as for the remaining occupational exposures examined	t'Mannetje et al, 1999, France, Germany, Italy, Sweden, The Netherlands (321) *Siew S. 2018, (46)*
Larynx (C32)	9.1	9.3	0.5	Combined risk factors	.	
		8.3	0.3	Asbestos	Adjusted for age, geographic region, and alcohol and tobacco consumption	Gustavsson et al, 1998, Sweden (317) *GBD Collaborators,2020 (47)*
		1.1	0.2	Welding fumes (metal dust, irritant gases, polycyclic aromatic hydrocarbons)	Adjusted for age, geographic region, and alcohol and tobacco consumption	Gustavsson et al, 1998, Sweden (317)
Bronchus and lung (C34)	24.0	29.0	5.3	Combined risk factors	.	.
		.	.	Industries [f]: metal production and processing workers, transportation workers and freight handlers, rubber and plastics industry, engine and vehicle construction, installation	Results for men; matched for age, gender, and region of residence; adjusted for smoking and asbestos exposure	Jöckel et al, 1998, Germany (51) *GBD Collaborators, 2020 (47)* *ILO , 2021, (65)*
		.	.	Industries [f]: chemical and oil processing, production of pottery and glass, engine, engine and vehicle construction, paper, wood, printing, cleaning services, hairdressing, housekeeping, and waste disposal	Results for women; matched for age, gender, and region; adjusted for smoking and time since quitting, asbestos, and education	Jahn et al, 1999, Germany (53) *GBD Collaborators,2020 (47)*
		.	.	Occupations [f]: metal production and processing worker (polycyclic aromatic hydrocarbons, chromates, arsenic compounds), transporta-tion workers and freight handlers (diesel engine exhaust)	Results for men; matched for age, gender, and region of residence; adjusted for smoking and asbestos exposure	Jöckel et al, 1998, Germany (51) *GBD Collaborators,2020 (47)*

(continued)

Cause of death [a]	Attributable fraction [b] (%)			Potentially implicated exposure or work environment [c]	Study design and data analysis [d]	Primary source of quantitative estimate [e]
	Total	Men	Women			
Bronchus and lung (continued)		.	.	Occupations [f]: assemblers, metal workers (soldering fumes containing cadmiun and lead), stock clerks, etc (diesel engine exhaust), restaurant and hotel service workers, waitresses (environmental tobacco smokers), ceramic and pottery workers (arsenic, asbestos, lead, silica), laudry workers and dry cleaners (chlorinated solvents and other industrial solvents)	Results for women; matched for age, gender, and region; adjusted for smoking and time since quitting, asbestos, and education	Jahn et al, 1999, Germany (53) *European Commission, 2021, (66)*
		14.0	0.6	Asbestos (and its synergistic joint action with smoking)	Restricted to male surgical patients from a general urban population; reference subjects were deceased men from the same metropolitan area; adjusted for age and smoking	Karjalainen et al, 1994, Finland (49) *GBD Collaborators,2020 (47)* *Furuya et al, 2018, (26)*
		2.7	0.2	Crystalline silica (quartz dust)	Standardized mortality ratio<br>Matched for age, gender, and region; adjusted for smoking and asbestos	Koskela et al, 1994, Finland (47)<br>Brüske-Hohlfeld et al, 2000, Germany (54) *GBD Collaborators,2020 (47)*
		2.5	0.1	Diesel engine exhaust	Matched for age, gender, and region; adjusted for smoking and asbestos	Brüske-Hohlfeld et al, 2000, Germany (54) *GBD Collaborators,2020 (47)*
		0.5	0.1	Welding fumes (mild steel welding, chromium and nickel welders excluded)	Restricted to men; matched for age, gender, and region; adjusted for smoking and asbestos	Jöckel et al. (52), 1998, Germany
		0.9	0.2	Arsenic (and its synergistic joint action with smoking)	Matched for year of birth and survival time	Järup & Pershagen, 1991, Sweden (29) *GBD Collaborators,2020 (47)*
		0.2	0.0	Cadmium	Restricted to men; standardized mortality ratio estimates based on gender and calendar year specific rates in the whole population; confounding by nickel hydroxide possible	Elinder et al, 1985, Sweden, United Kingdom, United States (48) *GBD Collaborators,2020 (47)*
		1.6	0.1	Chromium (hexavalent)	Adjusted for age, educational level, marital and socioeconomic status, smoking history, and other carcinogens under study	Droste et al, 2000, Belgium (56) *GBD Collaborators,2020 (47)*
		1.4	0.2	Lead	Restricted to men; matched for gender, year of birth, vital status, and, age at the end of follow-up	Anttila et al, 1995, Finland (46)
		1.5	0.0	Nickel sulfate	Restricted to men; matched for gender, year of birth, age at the end of follow-up, and vital status; adjusted for smoking, asbestos, chromates, cadmium, solvents, and gasoline	Anttila et al, 1998, Finland (43)
		4.5	1.2	Radon progeny	Standardized mortality ratio estimates based on national and regional cancer rates; silica dust and exhaust gases probably had co-carcinogenic or synergistic effects; smoking alone did not explain excess risk	Ahlman et al, 1991, Finland (44)
		3.0	2.0	Environmental tobacco smoke (tar and nitrosamines)	Restricted to subjects who had not smoked more than 400 cigarettes in their lifetime; multicenter case-referent study; matched for age and gender; adjusted in addition for educational level, proportion of life spent in urban areas, occupational exposure to lung carcinogens, intake of vegetables, beta-carotene, total carotenoids, and retinol	Boffetta et al, 1998, western European countries (87) *Stayner et al, 2007 (48) global*

(continued)

Cause of death [a]	Attributable fraction [b] (%)			Potentially implicated exposure or work environment [c]	Study design and data analysis [d]	Primary source of quantitative estimate [e]
	Total	Men	Women			
Bronchus and lung (continued)		0.0	0.7	Hairdressing (hair dyes and colorants)	Standardized incidence ratio estimates	Pukkala et al, 1992, Finland (45) *IARC Monographs,2018, (49)*
Bone (C40-C41)	0.6	0.6	0.6	Air carrier personnel (ionizing radiation)	Standardized incidence ratio estimates based on national cancer incidences.	Pukkala et al. (127), 1995, Finland
Skin melanoma (C43)	2.7	4.3	0.4	Combined risk factors	·	*John et al, 2020 (50)*
		2.4	0.3	Warehouse clerks (indoor work, diesel engine exhaust)	Adjusted for age, years of schooling, and ethnicity	Fritschi & Siemiatycki, 1996, Canada (322)
		1.9	0.0	Seafarer's outdoor work (repeated sunburns)	Standardized incidence ratio based on national cancer incidences	Pukkala & Saarni, 1996, Finland (79)
		0.1	0.1	Airline pilots' work (cosmic radiation)	Restricted to men; standardized incidence ratio based on national cancer incidence; proportion of current smokers among the pilots was similar to that expected in the general polulation	Haldorsen et al, 2000, Norway (150)
Skin non-melanoma (C44)	8.3	13.1	3.8	Ultraviolet radiation (outdoor work)	Standardized incidence ratio based on national cancer incidences	Pukkala & Saarni, 1996, Finland (79) *John et al, 2020 (50)*
Mesothelioma (all sites) (C45)	71.3	90.0	25.0	Asbestos	Case series study	Spirtas et al, 1994, United States (323)
		·	·	Asbestos	Case series study	Yates et al, 1997, United Kingdom (324) *Odgerel et al, 2017 (51)*
Female breast (C50)	1.7	·	1.7	Combined risk factors	·	·
·		·	1.7	Ionizing radiation	Restricted to postmenopausal women; adjusted for mean number of children, mean age at first delivery, and turnover rate for each job title	Weiderpass et al, 1999, Finland (126)
		·	0.1	Hairdressing (hair dyes)	Standardized incidence ratio estimates	Pukkala et al, 1992, Finland (45)
Cervix uteri (C53)	5.9	·	5.9	Aromatic hydrocarbon solvents (co-exposure with aliphatic and alicyclic, and chlorinated hydrocarbons)	Standardized for birth cohort, follow-up period, and social status; adjusted for mean parity, mean age at birth of first child, and turnover rate	Weiderpass et al, 2001, Finland (134)
Corpus uteri (endometrium) (C54)	1.1	·	1.1	Sedentary work (industrial sewers, low level of occupational physical activity)	Standardized for birth cohort, follow-up period, and social status; adjusted for mean parity, mean age at birth of first child, and turnover rate	Weiderpass et al, 2001, Finland (134)
Ovary (C56)	2.1	·	2.1	Combined risk factors	·	*GBD Collaborators,2020 (47)*
		·	0.7	Aromatic hydrocarbon solvents (co-exposure with aliphatic and alicyclic, and chlorinated hydrocarbons)	Stratified by birth cohort, follow-up period, and socio economic status; adjusted for mean number of children and turnover rate for each job title	Vasama-Neuvonen et al, 1999, Finland (132)
		·	0.3	Asbestos (co-exposure with silica dust and man-made vitreous fibers)	Stratified by birth cohort, follow-up period, and socio economic status; adjusted for mean number of children and turnover rate for each job title	Vasama-Neuvonen et al, 1999, Finland (132)
		·	0.4	Leather dust	Stratified by birth cohort, follow-up period, and socio economic status; adjusted for mean number of children and turnover rate for each job title	Vasama-Neuvonen et al, 1999, Finland (132)
		·	0.4	Diesel or gasoline engine exhaust	Stratified by birth cohort, follow-up period, and socio economic status; adjusted for mean number of children and turnover rate for each job title	Vasama-Neuvonen et al, 1999, Finland (132)
		·	0.3	Hairdressers (hair dyes and colorants)	Stratified by birth cohort, follow-up period, and socio economic status; adjusted for mean number of children and turnover rate for each job title	Vasama-Neuvonen et al, 1999, Finland (132)

(continued)

Cause of death [a]	Attributable fraction [b] (%)			Potentially implicated exposure or work environment [c]	Study design and data analysis [d]	Primary source of quantitative estimate [e]
	Total	Men	Women			
Prostate (C61)		6.0	·	Combined risk factors	·	·
		3.5	·	Metal dust, especially cadmium dust	Adjusted for age, ethnicity, socioeconomic status, Quatelet index, self versus proxy status of the respondent, and core substances	Aronsen et al, 1996, Canada (106)
		2.7	·	Herbicides	Adjusted for age, calendar period, and other farm exposures. Income, education, ethnic origin, and immigrant status examined as potential confounders	Morrison et al, 1993, Canada (103)
Kidney (renal cell carcinoma) (C64-C65)	3.1	4.7	0.8	Combined risk factors	·	*Michalek, 2019 Finland (52)*
		·	·	Oil refinery workers [f] (petroleum refining, constituents of gasoline: benzene, etc)	Standardized incidence ratio estimates based on national cancer incidences; adjusted for social class	Pukkala, 1998, Finland (325)
		·	·	Printers, printing industry [f]	Adjusted for age, cigarette-years, body mass index, and respondent status (self versus proxy); potential occupational confounders were chosen independently for each substance under study	Parent et al, 2000, Canada (326)
		·	·	Aviation gasoline[f]	Adjusted for age, cigarette-years, body mass index, and respondent status (self versus proxy); potential occupational confounders were chosen independently for each substance under study	Parent et al, 2000, Canada (326)
		·	·	Printing press operators used gasoline as as a cleaning agent	Restricted to subjects with reported occupational history; matched for year of birth, gender, and survival status at the time of data collection; adjusted for smoking, obesity, and coffee consumption	Partanen et al, 1991, Finland (327)
		·	·	Nonchlorinated hydrocarbon solvents [f]	Restricted to subjects with reported occupational history; matched for year of birth, gender, and survival status at the time of data collection; adjusted for smoking, obesity, and coffee consumption	Partanen et al, 1991, Finland (327)
		·	·	Iron and steel industry [f]	Multicenter population-based case-referent study	Mandel et al, 1995, Australia, Denmark, Germany, Sweden, United States (328)
		·	·	Dry-cleaning solvents [f]	Multicenter population-based case-referent study	Mandel et al, 1995, Australia, Denmark, Germany, Sweden, United States (328)
		·	·	Gasoline and other petroleum products [f]	Multicenter population-based case-referent study	Mandel et al, 1995, Australia, Denmark, Germany, Sweden, United States (328)
		1.1	0.0	Gasoline (tetraethyl lead used as additive)	Multicenter population-based case-referent study	Mandel et al, 1995, Australia, Denmark, Germany, Sweden, United States (328)
		0.4	0.2	Solvents	Matched for gender, age, and region; adjusted for smoking	Pesch et al, 2000 Germany (329)
		3.4	0.6	Heavy metals (cadmium and lead)	Matched for gender, age, and region; adjusted for smoking	Pesch et al, 2000 Germany (329)
					·	·

(continued)

Cause of death [a]	Attributable fraction [b] (%)			Potentially implicated exposure or work environment [c]	Study design and data analysis [d]	Primary source of quantitative estimate [e]
	Total	Men	Women			
Urinary bladder (C67)	10.3	14.2	0.7	Combined risk factors	·	*Hadkhale, 2018, Finland (53)*
		4.0	0.0	Textile dyes, paints, and pigments (2-naphthyl-amine)	Pair-matched for age, gender, and hospital	Tola et al, 1980, Finland (330)
		5.4	0.0	Leather and rubber (aromatic amines)	Pair-matched for age, gender, and hospital	Tola et al, 1980, Finland (330)
		2.8	0.4	Lead	Restricted to men; matched for gender, year of birth, age at the end of follow-up, and vital status; adjusted for smoking and vital status	Anttila et al, 1995, Finland (43)
		0.9	0.3	Chlorinated hydrocarbon solvents	Restricted to men; adjusted for age and social class; the male population of Finland used as the reference	Kauppinen et al, 1998, Finland (14)
		1.9	0.0	Polycyclic aromatic hydrocarbons	Matched for age, gender, and region; adjusted for smoking	Pesch et al, 2000, Germany (329)
Brain (C71)	6.4	10.6	1.3	Combined risk factors	·	·
		6.8	0.1	Precision metal workers, including machinists, tool and die makers, and sheet metal workers (metal dusts and fumes, lubricating oils, and solvents)	Restricted to men; adjusted for age at death and marital status	Thomas et al, 1986, United States (331)
		2.8	0.7	Lead	Restricted to men; matched for birth-year, age at diagnosis, and vital status; adjusted for year of first exposure measurement, smoking, exposure to gasoline and cadmium	Anttila et al, 1996, Finland (332)
		1.3	0.5	Aromatic hydrocarbon solvents	Standardized incidence ratios based on age-, gender-, and period-specific incidence rates of cancer in the general population	Anttila et al, 1998, Finland (318)
Hodgkin's disease (C81)	2.2	3.9	0.0	Farmers (pesticides and other chemicals used in cultivating grain, etc) [f]	Adjusted for age, gender, geographic region of farm, and their interactions using Poisson regression modeling	Pukkala & Notkola, 1997, Finland (137)
		·	·	Farmers (no specific exposure identified) [f]	The meta-analysis estimate of risk ratio was calculated using a random-effects model	Khuder et al, 1997, mostly European countries and United States (136)
Non-Hodgkin's lymphoma (C82-C85)	4.7	13.5	3.1	Combined risk factors	·	·
		1.5	0.5	Halogenated hydrocar-bon solvents (trichlo-roethylene, tetrachlo-roethylene, 1,1,1-trichloroethane)	Based on age-, gender-, and calendar-period-specific rates for the general Finnish population	Anttila et al, 1995, Finland (133)
		5.2	0.9	Herbicides (phenoxyacetic acids)	Restricted to men; matched for age, county, and vital status	Hardell & Eriksson, 1999, Sweden (138)
		7.4	1.7	Fungicides	Restricted to men; matched for age, county, and vital status	Hardell & Eriksson, 1999, Sweden (138)
Leukemia (C91-C95)	10.9	18.5	2.5	Combined risk factors	·	*Polychronatis et al, 2013 (54)*
		0.7	0.2	Benzene	Evaluation of 4 independent epidemiologic studies	Tomatis et al, 1991, international (120)
		17.8	2.3	Electrical occupations (low-frequency magnetic fields)	Matched for age, gender, parish, and proximity to a power line; adjusted for age, gender, motor fuel or exhaust fumes, benzene, oil products, solvents, and welding fumes	Feychting et al, 1997, Sweden (142)

(continued)

Cause of death [a]	Attributable fraction [b] (%)			Potentially implicated exposure or work environment [c]	Study design and data analysis [d]	Primary source of quantitative estimate [e]
	Total	Men	Women			
Mental disorders (all F codes)	3.5	7.3	1.8			
Vascular and unspecified dementia (F01, F03)	3.9	10.0	1.8	Pesticides (herbicides and insecticides) and fertilizers	Restricted to cases with symptoms occurring not more than 3 years before diagnosis (control for survival bias); adjusted for gender, education, and residence in community or institution	Lindsay et al, 1997, Canada (215)
Depressive episode (F32)	11.3	14.6	9.8	Job strain (high levels of psychological demands, low levels of decision latitude, and low levels of social support at work)	Adjusted for personal and occupational characteristics, including age, marital status, number of children, educational level, occupation, previous absenteeism for mental disorders, and stressful personal and occupational events during the previous 12 months	Niedhammer et al, 1998, France (333) *Niedhammer et al, 2021 (55)* *Niedhammer , Chastang, 2021 (60)*
Diseases of the nervous system and (all G andH codes)	3.1	5.1	1.7			
Spinal muscular atrophy (G12)	0.7	1.2	0.2	Electrical work (low-frequency magnetic fields)	Restricted to men; adjusted for age, calendar year, social class, and race	Sawitz al, 1998, the United States (334)
Parkinson's disease (G20-G21)	9.2	16.0	4.9	Pesticides (herbicides and insecticides)	Frequency-matched for age, gender, and race; adjusted for age, gender, race, smoking status, and farming	Gorell et al, 1998, the United States (216) *Lai et al, 2002 (58)*
		·	·	Pesticides (herbicides and insecticides)	A review of recent evidence	Checkoway and Nelson, 1999, Canada, Germany, Taiwan, the United States (335)
Alzheimer's disease (G30)	2.3	3.4	1.8	Electrical work (low-frequency magnetic fields)	Adjusted for education or social class, and age at onset or age at examination; age and gender not considered confounders	Sobel et al, 1995, Finland, United States (219) *Tyas et al, 2021 (59)*
Diseases of the circulatory system (all I codes)	12.4	14.4	6.7			
Ischemic heart disease (I21-I25)	16.9	18.9	9.1	Combined risk factors	·	*Pega et al, 2021 (56)* *Niedhammer et al, 2021 (55),* *Kivimäki et al, 2012 (61)*
		10.7	·	Shift work (involves work strain)	Restricted to men (shift workers versus day workers); total risk from ischemic heart disease adjusted for age, smoking, total cholesterol, systolic blood pressure, body mass index, spare time physical activity, and alcohol intake	*Takala et al, 2021,(63)* *ILO , 2021, (65)* *Eur.Commission, 2021, (66)* Tenkanen et al,1997 Finland (30)
		·	5.5	Shift work (involving work strain)	Restricted to women; mortality risk adjusted for age, cigarette smoking, body mass index, history of hypertension, diabetes mellitus and hypercholesterolemia, menopausal status, current use of postmenopausal hormones, past use of oral contraceptives, alcohol intake, level of physical activity, quintiles of vitamin E intake, average aspirin use, and parental history of myocardial infarction before 60 years of age; stratified by spouse's educational status	Kawachi et al, 1995, United States (34)
		6.2	2.3	Noise	Review and quantitative estimation of risk ratio	Olsen & Kristensen, 1991, Denmark (155) *Wu et al, 2017 (57)*
		1.0	0.0	Engine exhausts (including carbon monoxide)	Adjusted for calendar time and duration of employment; similar distributions of smoking habits and social class among the exposed and reference subjects	Stern, 1988, United States (16)

(continued)

Cause of death [a]	Attributable fraction [b] (%)			Potentially implicated exposure or work environment [c]	Study design and data analysis [d]	Primary source of quantitative estimate [e]
	Total	Men	Women			
Ischemic heart disease (continued)		2.2	1.5	Environmental tobacco smoke (carbon dioxide and nicotine)	Restricted to women; adjusted for age, alcohol intake, body mass index, history of hypertension, diabetes and hypercholesterolemia, current use of postmenopausal hormones, past use of oral contraceptives, vigorous fat intake, vitamin E intake, exercise, quintiles of saturated average aspirin use, and parental history of myocardial infarction before 60 years of age	Kawachi et al, 1997, United States (200)
Cerebrovascular diseases (I60-I69)	10.5	12.1	7.8	Combined risk factors	·	*Pega et al, 2021 (56)* *Niedhammer et al, 2021 (55)*
		4.8	4.8	Shift work	Longitudinal register study; adjustment by a Poisson regression analysis of the impact of occupational and socioeconomic variates on mortality	*Fransson et al, 2015 (62)* Virtanen & Notkola, 2001, Finland (205)
		7.6	3.2	Environmental tobacco smoke (including carbon monoxide)	Adjusted for age, gender, history of hypertension, heart disease, and diabetes	Bonita et al, 1999, New Zealand (207)
Diseases of the respiratory system (all J codes)	4.1	6.8	1.1			
Pneumonia (J12, J13, J15, J17)	1.1	1.4	0.3	Welders (metallic component of the welding fumes, oxides of nitrogen, and ozone)	Standardized mortality ratios were calculated based on age-, gender, and period-specific death rates of pneumonia in the general population; the fact that the excess mortality among men was confined to men younger than 65 years makes confounding by nonoccupational causes improbable	Coggon et al, 1994, United Kingdom (336)
Chronic obstructive pulmonary diseases (J41-J44, J47)	11.7	14.0	3.8	Combined risk factors	·	*Collegium Ramazzini, 2016 (22)* *GBD Collaborators, 2020 (47)*
	18.3	9.6	2.6	Dusty work such as manufacturing industry, construction work, and farming (organic dust, microbial dusts, endotoxins)	Stratified by gender, history of smoking, and social status	Isoaho et al, 1994, Finland (230)
		3.6	0.6	Welding fumes (chromium and nickel dust in stainless steel welding)	Adjusted for age, cigarette pack-years, duration of occupational exposures, and demographic variables	Hunting & Welch 1993, United States (231)
		1.2	0.9	Environmental tobacco smoke	Adjusted for age, gender, race, education, occupational exposure, and past smoking history	Euler et al1, 1987, United States (232)
Asthma (J45)	18.2	17.8	18.4	Combined risk factors	·	
				Occupations [f]: bakers, food manufacturing workers, painters and lacquerers, floor layers, farmers, animal husbandry workers, welders, plastic product workers	Incidence rates based on register data	Karjalainen et al, 2000, Finland (237)
		8.6	5.9	Farmers and agricultural workers, animal husbandry workers (grain dust, hay dust, animal epithelia, hairs or secretions, fodders)	Adjusted for age, gender, smoking status, and country (study center)	Kogevinas et al, 1999, Australia, New Zealand, United States, western Europe (226)
		1.2	0.1	Spray painters and lacquerers (epoxy resins or paints, isocyanates)	Adjusted for age, gender, smoking status, and country (study center)	Kogevinas et al, 1999, Australia, New Zealand, United States, western Europe (226)

(continued)

Cause of death [a]	Attributable fraction [b] (%)			Potentially implicated exposure or work environment [c]	Study design and data analysis [d]	Primary source of quantitative estimate [e]
	Total	Men	Women			
Asthma (continued)		0.3	4.7	Cleaners (chlorine and acids, solvents, dusts)	Adjusted for age, gender, smoking status, and country (study center)	Kogevinas et al, 1999, Australia, New Zealand, United States, western Europe (226)
		0.3	0.2	Plastics and rubber workers (eg, epoxides)	Adjusted for age, gender, smoking status, and country (study center)	Kogevinas et al, 1999, Australia, New Zealand, United States, western Europe (226)
		0.6	1.2	Bakers and pastry makers (flour dust)	·	·
		2.0	0.4	Welding fumes	Adjusted for age, gender, smoking, and formaldehyde exposure	Torén et al, 1999, Sweden (241)
		0.4	3.2	Textile dust	·	Torén et al, 1999, Sweden (241)
		0.0	0.2	Hairdressers and beauticians, etc (hair, acrylic monomers, resin, phthalates, persulfate salts, hair dyes and colorants)	Adjusted for age, smoking and atopy	Leino et al, 1997, Finland (240)
		5.8	3.9	Environmental tobacco smoke	Adjusted for age, gender, air pollution at workplace, occupational contact with animals, atopy, and ever-smoking	Flodin et al, 1995, Sweden (244)
Pneumoconioses (J60-J64, J67)	100	100	100	Asbestos dust, silica dust, organic dust	Based on the rarity of nonoccupational cases of pneumoconiosis	Karjalainen, 1998, Finland (11)
Cryptogenic fibrosing alveolitis (J84)	15.4	25.3	3.2	Metal dust, wood dust	Matched for age, gender, area of residence, and general practitioner; adjusted for smoking status and exposure to wood or metal dust	Hubbard et al, 1996, United Kingdom (26)
Diseases of the digestive system (all K codes)	2.1	2.3	1.5			
Gastric and duode nal ulcer (K25-K26)	29.0	29.0	29.0	Shift work (involving work strain)	Restricted to men; age-standardized hospitalization ratios for non-daytime workers; occupational groups with similar employment status but with daytime workers used only as a comparison group	Tüchsen et al, 1994, Denmark (337)
Diseases of the genitourinary system (all N codes)	1.3	3.0	0.4			
Chronic renal failure and nephritic syndrome (N03, N11, N18, N19, N28)	2.6	17.6	2.3	Combined risk factors	·	·
		3.6	0.6	Lead	Matched for age, gender, and region of residence (urban or rural area)	Nuyts et al, 1995, Belgium (338)
		5.7	0.3	Chromium	Matched for age, gender, and region of residence (urban or rural area)	Nuyts et al, 1995, Belgium (338)
		7.3	0.7	Quartz	Matched for age, gender, and region of residence (urban or rural area)	Nuyts et al, 1995, Belgium (338)
		2.2	0.7	Oxygenated hydrocarbons	Matched for age, gender, and region of residence (urban or rural area)	Nuyts et al, 1995, Belgium (338)

(continued)

Cause of death [a]	Attributable fraction [b] (%)			Potentially implicated exposure or work environment [c]	Study design and data analysis [d]	Primary source of quantitative estimate [e]
	Total	Men	Women			
Accidents and violent incidents (all V, W, X, Y codes)	3.1	3.7	0.4			
Accidents including accidental poisonings) (V01-X59, Y85-Y86)	5.4	6.5	0.4	Annual rate 3.6 per 100 000 workers	Based on statistics	Federation of Finnish Insurance Companies, 1999, Finland (9), Farmer's Social Insurance Institution, 1999, Finland (10),*and updates until 2022* *ILOSTAT, EUROSTAT, 1998-2021 (1,2,3,4,5,6,7)*
Homecide and injury purposely inflicted by other people (X85-Y09, Y87.1)	1.1	1.3	0.7	Policemen and guards	Based on statistics	*Takala, 2019, (64)* *European Commission, 2021, (66)* *Betti G et al, 2021, (72)* *Statistics China, 2014, (29)*
Suicides (X60-X84, Y87.0)	0.4	0.4	0.3	Combined risk factors	.	-------------
		0.2	0.2	Physicians (work stress)	Age-standardized death rates with other professionals (high social status, all or most members had an academic degree) and economically active population used as a comparison group	Isotalus & Saarela, 1999, Finland (339) Rimpelä et al, 1987, Finland (340)
		0.2	0.1	Lawyers (work stress)	Age-standardized death rates with other professionals (high social status, all or most members had an academic degree) and economically active population used as a comparison group	Rimpelä et al, 1987, Finland (340)
All items and general				*All risk factors and occupations*		*Dhungel et al, 2022, Japan (67)* *Takala, 2015, (68)* *Tran et al, 2022, (69)* *Hämäläinen et al, 2012-17,(11,30)* *Takala et al. 2017, (8)* *ILO, 2014, (70)* *ILO Takala et al, 2005, (71)*

[a] Codes of the International classification of diseases, 10th revision, in parentheses.
[b] Estimates are given separately by gender for those causes of death for which there was evidence of occupational etiology. [c] Agents, occupation or industry.
[d] Control of confounding variables.
[e] Authors, year, country, reference number (in parentheses as listed in the body of the text of Nurminen et al 2001).
[f] The implicated industries or occupations were not explicitly used as a basis for the estimates of attribured fraction.

Note: Updated new 2023 references to this Supplementary Annex are in ***italics*** *here above and the complete references are located below. Main article references are numbered 1- 28, and the Supplementary Annex references are numbered from 29 to 71*

View publication stats

References of this Supplementary Table S 3. include the main paper references of "Global-, Regional- and Country-level Estimates of the Work-related Burden of Diseases and Accidents in 2019 " numbered 1-29, supplementary references are numbered 30 -72. The original 340 references of Nurminen, M. and Karjalainen, A. are fully listed in Ref. 7 below.

1. Ervasti J, Pentti J, Nyberg ST, Shipley MJ, Leineweber C, Sørensen JK, Alfredsson L, Bjorner JB, Borritz M, Burr H, Knutsson A. Long working hours and risk of 50 health conditions and mortality outcomes: a multicohort study in four European countries. The Lancet Regional Health-Europe. 2021 Dec 1; 11:100212.
2. Peters SE, Dennerlein JT, Wagner GR, Sorensen G. Work and worker health in the post-pandemic world: a public health perspective. The Lancet Public Health. 2022 Feb 1;7(2):e188-94.
3. Takala, J. Global Estimates of Fatal Occupational Accidents. Epidemiology September 1999, Vol. 10 No. 5, 640-646. Available online: https://www.who.int/quantifying_ehimpacts/methods/en/takala.pdf (accessed on 10 November 2021).
4. Global Health Estimates. World Health Organization (WHO), available at https://www.who.int/data/global-health-estimates (accessed on 21 November, 2021).
5. International Labour Organisation. ILO List of Occupational Diseases (revised 2010), ILO Publications, Geneva. Available online: https://www.ilo.org/safework/info/publications/WCMS_125137/lang--en/index.htm (accessed on 21 November 2021)
6. IARC, 2022. Accessed through https://monographs.iarc.who.int/agents-classified-by-the-iarc/ (Accessed 24 Dec. 2022).
7. Nurminen, M. and Karjalainen, A. Epidemiologic estimate of the proportion of fatalities related to occupational factors in Finland, Scand. J. Work Environ. Health, Vol. 27, pp 161–21. https://www.sjweh.fi/article/605
8. GBD 2019 Risk Factors Collaborators. Global burden of 87 risk factors in 204 countries and territories, 1990–2019: a systematic analysis for the Global Burden of Disease Study 2019. The Lancet. 2020 Oct 17;396(10258):1223-49. https://doi.org/10.1016/S0140-6736(20)30752-2 and https://vizhub.healthdata.org/gbd-compare/
9. Takala, J.; Hämäläinen, P.; Nenonen, N.; Takahashi, K.; Chimed-Ochir, O.; Rantanen, J. Comparative Analysis of the Burden of Injury and Illness at Work in Selected Countries and Regions. Cent. Eur. J.Occup. Environ. Med. **2017**, [, 7–31 Available online: https://www.nnk.gov.hu/cejoem/Volume23/Vol23No1-2/23_1-2_Article_01-.pdf Supplementary excel files: https://www.nnk.gov.hu/cejoem/database.html (accessed on 10 November 2022)
10. Hämäläinen P, 2010. "Global Estimates of Occupational Accidents and Fatal Work-Related Diseases". Doctoral dissertation, Publication 917, Tampere University of Technology, Finland. Available online: https://trepo.tuni.fi/bitstream/handle/10024/115087/hamalainen.pdf?sequence=1&isAllowed=yhttps://trepo.tuni.fi/bitstream/handle/10024/115087/hamalainen.pdf?sequence=1&isAllowed=y (accessed on 10 November 2021)
11. Hämäläinen P, Takala J, Tan BK. Global estimates of occupational injuries and work-related illnesses 2017. Workplace Safety and Health Institute, Ministry of Manpower, Singapore. Available online: https://www.mom.gov.sg/-/media/mom/files/pdf/wshi-past-reports/global-estimates-for-occupational-accidents-and-workrelated-diseases-nov-2017.pdf (accessed on 3 October 2023).
12. Elsler, D,; Takala, J.; Remes, J.; An International Comparison of the Cost of Work-related Accidents and Illnesses. European Agency for Safety and Health at Work. Available online: https://osha.europa.eu/sites/default/files/international_comparison-of_costs_work_related_accidents.pdf (accessed on 21 November 2021)
13. Hämäläinen P, Takala J, Saarela KL, 2006. Global Estimates of occupational accidents, Safety Science, Vol. 44,pp. 137-156.Available online: http://www.osha.mddsz.gov.si/resources/files/pdf/occupational_acc_article_final.pdf doi:10.1016/j.ssci.2005.08.017 (accessed on 10 November 2022)
14. Steenland K.: Attributable fraction: example, cancers due to occupation in the US, Presentation at OCRC Research Day: Assessing the Human and Economic Burden of Occupational Cancer, Toronto, Ontario, 23 March 2011 [Online]. Available online: http://www.occupationalcancer.ca/wp-content/uploads/2011/03/Steenland.pdf (accessed on 10 November 2022)
15. Rushton, L.; Hutchings, S. J.; Fortunato, L.; Young, C.; Evans, G. S.; Brown, T.; Bevan, R.; Slack, R.; Holmes, P.; Bagga, S.; Cherrie, J. W.; Van Tongeren, M. 2012. "Occupational cancer burden in Great Britain" in Br J Cancer, Vol. 107, pp S3–S7,
16. Driscoll, T.; Takala, J.; Steenland, K.; Corvalan, C.; Fingerhut. M. 2005. "Review of estimates of the global burden of injury and illness due to occupational exposures" in Am. J. Ind. Med., Vol. 48, pp 491–502, doi: 10.1002/ajim.
17. Morrell S, Kerr C, Driscoll T, Taylor R, Salkeld G, Corbett S. Best estimate of the magnitude of mortality due to occupational exposure to hazardous substances. Occupational and Environmental Medicine 1988; 55: 634-631.
18. Collegium Ramazzini: A New Approach to the Control of Chronic Obstructive Pulmonary Disease (COPD), 19th statement, published on 19 July 2016 [Online]. Available online: http://www.collegiumramazzini.org/news1.asp?id=144 and http://www.collegiumramazzini.org/download/19_NineteenthCRStatement(2016).pdf
19. European Agency for Safety and Health at Work. The economics of occupational safety and health – the value of OSH to society. Web-site available online: https://visualisation.osha.europa.eu/osh-costs#!/ (accessed on 21 November 2022)
20. Niedhammer I, Sultan-Taïeb H, Parent-Thirion A, Chastang JF. Update of the fractions of cardiovascular diseases and mental disorders attributable to psychosocial work factors in Europe. International Archives of Occupational and Environmental Health. 2022; 95(1): 233–47.
21. Boschetto P, Quintavalle S, Miotto D, Lo Cascio N, Zeni E, Mapp CE. Chronic obstructive pulmonary disease (COPD) and occupational exposures. Journal of Occupational Medicine and Toxicology. 2006 Dec;1(1):1-6.
22. Li X, Cao X, Guo M, Xie M, Liu X. Trends and risk factors of mortality and disability adjusted life years for chronic respiratory diseases from 1990 to 2017: systematic analysis for the Global Burden of Disease Study 2017. BMJ. 2020 Feb 19;368.

23. GBD 2016 Occupational Risk Factors Collaborators. Global and regional burden of disease and injury in 2016 arising from occupational exposures: a systematic analysis for the Global Burden of Disease Study 2016. Occupational and environmental medicine. 2020 Mar 1 1;77(3):133-41.
24. Pega F, Náfrádi B, Momen NC, Ujita Y, Streicher KN, Prüss-Üstün AM, Group TA, Descatha A, Driscoll T, Fischer FM, Godderis L. Global, regional, and national burdens of ischemic heart disease and stroke attributable to exposure to long working hours for 194 countries, 2000–2016: A systematic analysis from the WHO/ILO Joint Estimates of the Work-related Burden of Disease and Injury. Environment International. 2021; 154:106595.
25. Odgerel, C.-O.; Takahashi, K.; Sorahan, T.; Driscoll, T.; Fitzmaurice, C.; Makoto, Y.; Sawanyawisuth, K.; Furuya, S.; Tanaka, F.; Horie, S.; van Zandwijk, N.; Takala J. Estimation of the Global Burden of Mesothelioma Deaths from Incomplete national Mortality Data. J. Occup. Environ. Med. 2017, 74, 851–858 Available online: https://www.ncbi.nlm.nih.gov/pmc/articles/PMC5740549/pdf/oemed-2017-104298.pdf (accessed 29 November 2022)
26. Furuya S, Chimed-Ochir O, Takahashi K, David A, Takala J. Global Asbestos Disaster. Int J Environ Res Public Health 2018, 15(5), 1000. Supplementary tables available (accessed on 10 November 2022)
27. Work Environment Authority, Sweden. Work-related Mortality in Sweden, Scientific review 2019:3 – report 1, in Swedish, (Arbetsmiljöverket, Arbetsrelaterad dödlighet - delrapport 1, Kunskapssammanställning 2019:3). Accessible online: https://www.av.se/globalassets/filer/publikationer/kunskapssammanstallningar/arbetsrelaterad-dodlighet-rap-2019-3-del-1. pdf?hl=Research%20report%20%20mortality (accessed on 21 November 2022)
28. WHO/ILO. WHO/ILO joint estimates of the work-related burden of disease and injury, 2000-2016: global monitoring report: Geneva: World Health Organization and the International Labour Organization, 2021. Available online: https://www.who.int/teams/environment-climate-change-and-health/monitoring/who-ilo-joint-estimates Available online:(accessed on 21 November 2021)
29. Statistical Communiqué of the People's Republic of China on the 2013 National Economic and Social Development, National Bureau of Statistics of China, 2014, November. Available online: http://www.stats.gov.cn/english/PressRelease/201402/t20140224_515103.html (accessed on 18 June 2022).

Numbers below 30 - 72 are new references to above Supplement Table S 3.

30. Takala, J., Hämäläinen, P., Saarela, K.L., Loke, Y.Y., Manickam, K., Tan, W.J., et al. Global Estimates of the Burden of Injury and Illness at Work in 2012. JOEH 11:326-337, 14 April 2014. Available online: https://www.tandfonline.com/doi/full/10.1080/15459624.2013.863131 (accessed on 10 November 2021)
31. Alele, F.O.; Franklin, T.; Leggat, P.; Occupational tuberculosis in healthcare workers in sub-Saharan Africa: A systematic review. Archives of Environmental & Occupational Health. Volume 74, 2019 - Issue 3, https://doi.org/10.1080/19338244.2018.1461600
32. NIOSH/CDC. Tuberculosis, Overview. National Institute for Safety and Health. Available online: https://www.cdc.gov/niosh/topics/tb/default.html (accessed on 26 November 2021)
33. Torén, K.;Blanc P.; Naidoo R. ; Murgia, N. ; Qvarfordt, I.; Aspevall, O.; Dahlman-Höglund, A.; Schioler, L. Occupational exposure to dust and to fumes, work as a welder and invasive pneumococcal disease risk. Occupational and Environmental Medicine, Vol 77, issue 2. Available online: https://oem.bmj.com/content/oemed/77/2/57.full.pdf (accessed on 26 November 2021)
34. Paget-Bailly, S.; Cyr, D.; Luce, D. Occupational exposures to asbestos, polycyclic hydrocarbons and solvents and cancers of the oral cavity an pharynx: a quantitative literature review. Int Archives of Occupational and Environmental Health 85, 341- 351 (2012), https://doi.org/10.1007/s00420-011-0683-y
35. Jansson, C.;, Oh, JK:; Martinsen, J.; Lagergren, J.; Plato, N.; Kjaerheim,K.; Pukkala, E.; Sparén,P.; Tryggvadottir, L.; Weiderpass, E. Occupation and risk of oesophageal adenocarcinoma and squamous-cell carcinoma: The Nordic Occupational Cancer Study. International Journal of Cancer. (2014). Available online: https://onlinelibrary.wiley.com/doi/10.1002/ijc.29409 (accessed 27 November 2021)
36. Oddone, E.; Modonesi, C.; Gatta, G. Occupational exposures and colorectal cancers: A quantitative overview of epidemiological evidence. World Journal of Gastroenterology. 2014 September 21; 20(35): 12431-12444 Available online: DOI 10.3748/wjg.v20.i35.12431 (accessed 27 November 2021)
37. Awan, K.; Hedge, R.; Cheever, V.; Carroll, W.; Khan, S.; Patil, S.; Warnakulasuriya, S. Oral and pharyngeal cancer risk associated with occupational carcinogenic substances. Head and Neck, Volume40, Issue12 (2018) https://doi.org/10.1002/hed.25486
38. Santibañez, M.; Alguacil, J.; García de la Hera, M. ; Navarrete-Muñoz, E.; Llorca, J.; Aragonés, N.; Kauppinen, T.; Vioque, J.Occupational exposures and risk of stomach cancer by histological type. Occupational & Environmental Medicine, BMJ. Volume 69, Issue 4 (2011). http://dx.doi.org/10.1136/oemed-2011-100071

39. Talibova, M.; Sormunen, J.; Hansen, J.; Kjaerheime, K.; Martinsen, Jl.; Sparen, P.; Tryggvadottir, L.; Weiderpass, E.; Pukkala, E. Benzene exposure at workplace and risk of colorectal cancer in four Nordic countries. Cancer Epidemiology, Volume 55, August 2018, Pages 156-161. https://doi.org/10.1016/j.canep.2018.06.011 Available online: https://helda.helsinki.fi//bitstream/handle/10138/305071/1_s2.0_S187778211830300X_main.pdf?sequence=1 (accessed 27 November 2021)
40. Kubo, S.; Takemura, S.; Tanaka, S.; Shinkawa, H.; Kinoshita, M.; Hamano, G.; Ito, T.; Koda, M.; Aota, T. Occupational cholangiocarcinoma caused by exposure to 1,2- dichloropropane and/or dichloromethane, Ann Gastroenterol Surg. 2018 Mar; 2(2): 99–105, doi: 10.1002/ags3.12051 . Available online: https://www.ncbi.nlm.nih.gov/pmc/articles/PMC5881298/pdf/AGS3-2-99.pdf (accessed 27 November 2021)
41. Brandi, G.; Tavolari, S. Asbestos and Intrahepatic Cholangiocarcinoma, Cells. 2020 Feb; Available online: doi: 10.3390/cells9020421 and https://www.ncbi.nlm.nih.gov/pmc/articles/PMC7072580/pdf/cells-09-00421.pdf (accessed 27 November 2021)
42. Farioli, A.; Straif, K.; Brandi, G.; Curti, S.; Kjaerheim, K.; Martinsen, J.I.; Sparen,P.; Tryggvadottir, L.; Weiderpass, E.; Biasco, G.; Violante, F.S.; Mattioli, S.; Pukkala, E. Occupational exposure to asbestos and risk of cholangiocarcinoma: a population-based case– control study in four Nordic countries Occup Environ Med 2018;75:191–198 Available online: https://oem.bmj.com/content/oemed/75/3/191.full.pdf (accessed 27 November 2021)
43. Andreotti, G.; Silverman, D.T. Occupational Risk Factors and Pancreatic Cancer: A Review of Recent Findings. Mol Carcinog. 2012 Jan; 51(1): 98–108. doi: 10.1002/mc.20779. Available online: https://www.ncbi.nlm.nih.gov/pmc/articles/PMC6394840/ (accessed 27 November 2021)
44. Santibañez, M.; Vioque, J.; Alguacil, J.; García de la Hera, M.; Moreno-Osset, E; Carrato, A. ; Porta, M. Occupational exposures and risk of pancreatic cancer. European Journal of Epidemiology, Springer Verlag, 2010, 25 (10), pp.721- 730. 10.1007/s10654-010-9490-0 .
45. Bagga, S.; Holmes, P.; Cherrie, J.; Van Tongeren, M.; Fortunato, L.; Hutchings, S.; Rushton, L. The burden of occupational cancer in Great Britain, Pancreatic cancer. RR 932, Research Report, Health and Safety Executive 2012. Available online: https://www.hse.gov.uk/research/rrpdf/rr932.pdf (accessed 29 November 2021)
46. Siew, S. Occupational Exposures (Wood Dust, Iron and Welding Fumes and Risk in Cancers of Lung and Nose among Men in Nordic Countries. Doctoral dissertation, Publication 2376, Tampere University, 2018, Finland. Available online: https://trepo.tuni.fi/bitstream/handle/10024/103347/978-952-03-0735-6.pdf?sequence=1&isAllowed=y (accessed 29 November 2021)
47. GBD 2019 Risk Factors Collaborators*. Global burden of 87 risk factors in 204 countries and territories, 1990–2019: a systematic analysis for the Global Burden of Disease Study 2019. Lancet 2020; 396: 1223–49 (*For the list of Collaborators see Viewpoint Lancet 2020; **396:** 1135–59). Available online: https://www.thelancet.com/journals/lancet/article/PIIS0140-6736(20)30752-2/fulltext and https://vizhub.healthdata.org/gbd-compare/ (accessed 29 November 2021)
48. Stayner, L.; Bena, J.; Sasco, A.J.; Smith, R.; Steenland, K.; Kreuzer, M.; Straif, K. Lung Cancer Risk and Workplace Exposure to Environmental Tobacco Smoke. American Journal of Public Health **97**, 545_551, 2007. Available online https://doi.org/10.2105/AJPH.2004.061275 (accessed 29 November 2021)
49. International Agency for Research on Cancer, IARC. Occupational Exposures of Hairdressers and Barbers and Personal Use of Hair Colourants, IARC Monographs, 2018/06, Available online: https://monographs.iarc.fr/wp-content/uploads/2018/06/mono99-17.pdf (accessed 29 November 2021)
50. John, S.M.;Garbe, C.; French, L.E.; Takala, J.; Yared, W.; Cardone, A.; Gehring, R.;Spahn, A.; Stratigos, A. Improved protection of outdoor workers from solar ultraviolet radiation: position statement. JEADV, 21 November 2020 https://doi.org/10.1111/jdv.17011 Available online: https://onlinelibrary.wiley.com/doi/epdf/10.1111/jdv.17011 (accessed 29 November 2021)
51. Odgerel, C.-O.; Takahashi, K.; Sorahan, T.; Driscoll, T.; Fitzmaurice, C.; Makoto, Y.; Sawanyawisuth, K.; Furuya, S.; Tanaka, F.; Horie, S.; van Zandwijk, N.; Takala J. Estimation of the Global Burden of Mesothelioma Deaths from Incomplete national Mortality Data. J. Occup. Environ. Med. 2017, 74, 851–858 Available online: https://www.ncbi.nlm.nih.gov/pmc/articles/PMC5740549/pdf/oemed-2017-104298.pdf (accessed 29 November 2021)
52. Michalek, I. Occupational Exposure and Risk of Kidney and Renal Pelvis Cancer in the Nordic Countries. Doctoral dissertation, Tampere University Dissertation 126, 2019, Finland. Available online: https://trepo.tuni.fi/bitstream/handle/10024/117385/978-952-03-1239-8.pdf?sequence=2&isAllowed=y (accessed 29 November 2021)
53. Hadkhale, K. Occupational Exposure and Risk of Bladder Cancer, Population based studies in the Nordic countries and Canada, Doctoral dissertation, Acta Universitatis Tamperensis 2435, Tampere, Finland. Available online: https://trepo.tuni.fi/bitstream/handle/10024/104597/978-952-03-0896-4.pdf?sequence=1&isAllowed=y (accessed 29 November 2021)

54. Polychronakis, I.; Dounias, G.; Makropoulos, V.; Riza, E.; Athena, L. Work-related leukemia: a systematic review. J Occup Med Toxicol. 2013; 8: 14. doi: 10.1186/1745-6673-8-14. Available online: https://www.ncbi.nlm.nih.gov/pmc/articles/PMC3668148/pdf/1745-6673-8-14.pdf (accessed 29 November 2021)
55. Niedhammer, I. ; Sultan-Taïeb, H. ; Parent-Thirion, A. ; Chastang, JF. Update of the fractions of cardiovascular diseases and mental disorders attributable to psychosocial work factors in Europe. Int Arch Occup Environ Health (2021). Available online: https://doi.org/10.1007/s00420-021-01737-4 (accessed 29 November 2021)
56. Pega, F.; Náfrádi, B.; Momen, N.C.; Ujita, Y.; Streicher, K.N.; Prüss-Üstün, A.M.; Descatha, A.; Driscoll, T.; Fischer, F.M.; Godderis, L.; Kiiver, H.M.; Li, J.; Magnusson Hanson, L.L.; Rugulies, R.; Sørensen, K.; Woodruff, T.J. Global, regional, and national burdens of ischemic heart disease and stroke attributable to exposure to long working hours for 194 countries, 2000–2016: A systematic analysis from the WHO/ILO Joint Estimates of the Work-related Burden of Disease and Injury, Environment International, Volume 154, 2021, https://doi.org/10.1016/j.envint.2021.106595. Available online: https://www.sciencedirect.com/science/article/pii/S0160412021002208 (accessed 29 November 2021)
57. Wu, X.; Yang, D.; Fan, W.; Fan, C.; Wu, G. Cardiovascular Risk Factors in Noise-Exposed Workers in China: Small Area Study, Noise Health. 2017 Nov-Dec; 19(91): 245–253. doi: 10.4103/nah.NAH_56_16 Available online: https://www.ncbi.nlm.nih.gov/pmc/articles/PMC5771056/ (accessed 29 November 2021)
58. Lai, B.C.L.; Marion, S.A.; Teschke, K.; Tsui J.K.C. Occupational and environmental risk factors for Parkinson's disease. Parkinsonism & Related Disorders 8 (2002) 297-309. Available online: https://www.sciencedirect.com/science/article/pii/S1353802001000542 (accessed 29 November 2021)
59. Tyas, L.S.; Manfreda, J.; Strain, L.A.; Montgomery, P.R. Risk factors for Alzheimer's disease, Int J Epidemiol, Vol. 30, Issue 3, June 2001, pp 590-597, Available online: DOI: 10.1093/ije/30.3.590 (accessed 29 December 2021).
60. Niedhammer, I.; Chastang, J-F. Overall fraction of disease attributable to multiple dependent risk factors: a new formula. The Lancet Neurology (IF44.182), Pub Date : 2021-12-01,DOI: 10.1016/s1474-4422(21)00366-5
61. Kivimäki M, et al. Job strain as a risk factor for coronary heart disease: a collaborative meta-analysis of individual participant data. The Lancet, 2012;380(9852):1491-7.
62. Fransson EI, et al. Job strain and the risk of stroke: an individual-participant data meta-analysis. Stroke, 2015;46(2):557-9
63. Takala, J.; Iavicoli, S.; Kang, SK.; Nogueira, C.; Gagliardi, D.; Kocks, D.; Rantanen, J. Work-related injuries and diseases, and COVID-19. International Journal of Labour Research, ILO, 2021 / Vol. 10 / Issue 1–2, pp. 27-47. Available online: https://www.ilo.org/wcmsp5/groups/public/---ed_dialogue/---actrav/documents/publication/wcms_806895.pdf
64. Takala, J. Burden of Injury due to Occupational Exposures. In book: U.Bültmann,J.Siegrist(eds.), Handbook of Disability, Work and Health, 2019 Springer Verlag, Available online: https://www.researchgate.net/publication/337305370_Burden_of_Injury_due_to_Occupational_Exposures
65. International Labour Office, Enhancing social dialogue towards a culture of safety and health – What have we learned from the Covid-19 Crisis? World Day Report, ILO Geneva 2022, 62 pages, Available online: https://www.ilo.org/wcmsp5/groups/public/---ed_protect/---protrav/---safework/documents/publication/wcms_842505.pdf
66. European Commission, EU strategic framework on health and safety at work 2021-2027, Occupational safety and health in a changing world of work. Brussels 28.06.2021, Available online: https://eur-lex.europa.eu/legal-content/EN/TXT/PDF/?uri=CELEX:52021DC0323&from=EN
67. Dhungel, B.; Murakami, T.; Gilmour, S.; Ikeda, S.; Wada,K.; Occupation- and industry-specific cancer mortality among Japanese women from 1980 to 2015. BMC Public Health (2022) 22:2003 https://doi.org/10.1186/s12889-022-14304-4
68. Takala J. Eliminating occupational cancer. Ind Health. 2015;53:307–9. https://doi.org/10.2486/indhealth.53-307 https://www.jstage.jst.go.jp/article/indhealth/53/4/53_53-307/_pdf/-char/en
69. Tran KB, Lang JJ, Compton K, Xu R, Acheson AR, Henrikson HJ, et al. The global burden of cancer attributable to risk factors, 2010–19: a systematic analysis for the Global Burden of Disease Study 2019. The Lancet. 2022;400:563–91 https://www.thelancet.com/pdfs/journals/lancet/PIIS0140-6736(22)01438-6.pdf
70. International Labour Office (ILO). 2014. Safety and Health at Work: a vision for sustainable prevention, Report, XX World Congress on Safety and Health at Work 2014: Global Forum for Prevention, Frankfurt, 2014. Available online: http://www.ilo.org/safework/info/publications/WCMS_301214/lang--en/index.htm (accessed on 10 November 2021).
71. Takala, J. ILO Introductory Report: Decent Work - Safe Work. XVII World Congress on Safety and Health at Work, Orlando, Florida, International Labour Organisation, Geneva, 2005, 49 p. Available online: http://goo.gl/ZC6OQD and http://ilo.org.mt/Portals/0/docs/intrep_05.pdf, and https://www.ilo.org/wcmsp5/groups/public/---europe/---ro-geneva/---sro-moscow/documents/genericdocument/wcms_312093.pdf Accessed 9 September, 2023
72. Betti,G, Vanadzins, I,Vlachou,H,Takala,J.; Methodological study on under-reporting of occupational accidents in European Union. EUROSTAT Project Report, Luxembourg, 2021, 39 p.,private file Research Gate, https://www.researchgate.net/publication/342128040_Methodological_study_on_under-reporting_of_occupational_accidents_in_European_Union

Supplementary Annex: Data Table covering consolidated global, regional and country data on work-related diseases, injuries and calculation methods. Excel formatted data converted to delimited pdf-file. Pages 21 - 118 .Global,regional and country data(converted from excel.file, available on request)

AFRO	Country "United Nation Country Classification
(Geographical Region)"	"United Nation Country Classification
(Geographical Sub-region)"	EU28	Arab	Labour force	Total employment
Labour force by sector (%) Agriculture	Labour force by sector (%) Industry
Labour force by sector (%) Service	"Labour force by sector
Agriculture"	"Labour force by sector
Industry"	"Labour force by sector
Service"	"Occupational injuries in 2019 reported to ILO
Fatal"	"Occupational injuries in 2019 reported to ILO
Non-fatal"	"Accidents at work in 2019 reported to Eurostat
Fatal"	"Accidents at work in 2019 reported to Eurostat
Non-fatal"	"Global estimates of occupational accidents (Fatal)
Agriculture"	"Global estimates of occupational accidents (Fatal)
Industry"	"Global estimates of occupational accidents (Fatal)
Service"	"Global estimates of occupational accidents (Fatal)
Total"	"Global estimates of occupational accidents
(At least four days absence)
Lower limit (Eq. j) (0.11) "	"Global estimates of occupational accidents
(At least four days absence)
Upper limit (Eq. j) (0.06)"	"Global estimates of occupational accidents
(At least four days absence) Average"	Average and the best estimate	Fatal
work-related diseases 2019	ILO Total mortality	IHME Deaths (All)
IHME DALY (All) IHME YLD (All)	IHME Deaths (Injury)	IHME DALY (Injury)
IHME YLD (Injury)	IHME Deaths (Disease)	DALY (Disease)	YLD (Disease)
"Disease Multiplier YLD/YLL
(Country)"	"Disease Multiplier
YLD/YLL
(WHO Division)" "Nominal GDP World Bank
(Millions USD)" *Employment (for purpose of burden estimation)	(1a) Fatal
Occupational Accidents	(1b) Fatal occupational accidents, EUROSTAT	(1c)
Fatal Occupational Accidents per 100,000 workers	(2) Fatal work-related
Diseases	(2a) Fatal Work-related Diseases per 100,000 workers	(3)
Non-fatal Occupational Accidents	(4) YLL-Injuries (Years per case)
(5) YLL-Diseases (Years per case)	(6) YLL-Fatal Injuries (Years)	(7)
YLL-Fatal Diseases (Years)	(8) YLD-Non-Fatal Injuries and Diseases (8.1)
YLD-Non-Fatal Injuries and Diseases (WHO multiplier)	(8.2) YLD-Non-Fatal
Injuries and Diseases (using YLD GBD Disease)	(8) CHOOSE	(9) DALY
(YLL+YLD)	(9.1) DALY (YLL+YLD)	(9.2) DALY (YLL+YLD)	(9) CHOOSE
(9a) DALY per 100,000 workers	"(10a) Total Costs
(Millions USD)" "(10b) Total Costs contribution to GDP loss
(%)"	"(11b) YLL contribution to GDP loss
(%)"	"(12a) Cost of YLD
(Millions USD)" "(12b) YLD contribution to GDP loss
(%)"	"(13a) Injury Contribution to GDP loss
(%)"	"(13b) Disease contribution to GDP loss
(%)"	"GDP per employed person
(US$)"	IHME Deaths B.1 Neoplasm (occupational) IHME DALY Neoplasm
(occupational)	IHME YLD Neoplasm (occupational)	IHME YLL Neoplasm
(occupational)	IHME YLL/Deaths Neoplasm (occupational) YLLnewILO(coeff)
YLLnewILO	YLDnew	DALYcancer	13(%)	13($)	DALY MSD B.9.3
(occupational)	14(%)	14($)	Deaths:IHD B.2.1 (male-15-49)	IHD(male-50-69)
IHD(male70+)	Stroke (male-15-49) B.2.3	CVB(male-50-69) CVB(male-70+)
IHD(female-15-49)	IHD(female-50-69)	IHD(female-70+)
CVB(female-15-49)	CVB(female-50-69)	CVB(female-70+) DEATHS
CARDIO-MALE	DEATHS CARDIO-FEMALE	Amale	Afemale Cmale	Cfemale
Deaths-male adjusted	Deaths-female adjusted	Deaths-male-work related

Deaths-female-work related Deaths total Circulatory diseases
DALY-IHD(male-15-49) DALY-IHD(male-50-69) DALY-IHD(male70+)
DALY-CVB(male-15-49) DALY-CVB(male-50-69) DALY-CVB(male-70+)
DALY-IHD(female-15-49) DALY-IHD(female-50-69) DALY-IHD(female-70+)
DALY-CVB(female-15-49) DALY-CVB(female-50-69) DALY-CVB(female-70+)
DALY-male DALY-female DALY-male-adjusted DALY-female-adjusted
DALY-male-work related DALY-female-work related DALY total
15-Cardio(%) 16(%) DALY(cancer) DALY(MSD) DALY(cardio)
DALY(injury) DALY(other) CHOOSE cancer1(%) MSD1(%) cardio1(%)
injury1(%) other1(%) Deaths(cancer) Death Circulatory disease (*)
Deaths(circular) Deaths(injury) Total death Other disese (New)
Deaths(cancer)(%) Deaths(circular)(%) Deaths(injury)(%)
Deaths(other)(%) Multiplier psychosocial/job strain (depression) IHME,
Depression DALY (15-49 Men) 50-69 Men 70+ Men IHME, Depression DALY
(15-49, women) 50-69 women 70+ women DALY, Depression (Men) DALY,
Depression (Women) DALY Depressive disorders (Psychosocial factors/job
strain) DALY (depression + all other) Total cost contribution to GDP (incl.
Psycho factors) Multiplier psychosocial/job strain (CVD) DALY, CVD
(psychosocial/job strain) DALY total (incl. Depression + CVD) Total
cost contribution to GDP (incl. Depression + CVD) DALY, psychosocial
DALY, Cancer, % DALY, MSD, % DALY, Cardio, % DALY, Injury, % DALY,
psychosocial, % DALY, others, % Total
AFRO Algeria Africa Northern Africa 10858275,00
10858275,00 9,60 30,42 59,99 1042394,40 3303087,26
6513879,17 187,63 716,77 1179,01 2083,41
1894011,87 3472355,09 2683183,48 2683183,00 6195,34 8278,75
2504,90 207475,04 101223,97 1749,38 112103,40 22916,44
755,52 95371,64 78307,53 4,59 3,69 145164,00
10858275,00 2083,41 2083,41 19,19 6195,34 57,06 2683183,48 50,98
22,59 106216,43 139927,94 669424,08 544287,98
105599,66 544287,98 915568,45 790432,35 351744,03
790432,35 8431,99 12240,21 8,43 2,27 8949,51 6,17 14,58
85,42 13368,97 207,90 6033,17 66,12 5967,04 28,70 1098,82 31537,77
349,47 31887,23 0,29 0,43 54168,58 0,50 0,72 2724,72
9875,99 18713,19 729,05 2881,38 8057,59 1719,55 6320,90 19337,72
926,60 2741,63 9395,26 25134,72 21286,34 7785,96 10975,25
0,31 0,52 7785,96 10975,25 1121,18 735,34 1856,52 133597,36
293070,66 236801,84 45055,26 96024,48 109334,66
84091,36 184369,17 235681,52 58199,98 98675,87
128256,11 683126,59 546648,92 211611,42 281852,54
30472,04 18884,12 49356,16 0,45 1,03 31887,23
54168,58 49356,16 112103,40 542916,97 3,48 5,92
5,39 12,24 72,97 1098,82 2271,61 1856,52 2083,41 8693,84 3240,00 12,64
26,13 23,96 37,27 0,17 8605,07 1193,32 161,79 28299,75 6736,48
947,41 9852,32 35352,03 7503,92 797936,27 7,35 0,58 28527,86
826464,13 7,61 36031,78 3,86 6,55 5,97 13,56 4,36
65,69 100,00
AFRO Angola Africa Middle Africa 13155275,00
12243086,00 50,73 6,80 42,47 6210917,53 832529,85
5199638,62 1117,97 180,66 941,13 2239,76
2036144,29 3732931,21 2884537,75 6985,00 9224,76 2739,42
209541,34 83208,96 2021,37 126129,57 19125,35 718,05
83411,77 64083,61 3,32 3,69 62307,00 12243086,00
2239,76 2239,74 18,29 6985,00 57,05 2884537,75 52,94 26,92
118564,23 188018,23 644576,52 715868,00 85275,13
715868,00 951158,98 1022450,47 391857,60 1022450,47

7768,95 4840,60 7,77 2,50 3280,35 5,26 14,69 85,31 5089,16 187,12
5571,48 54,40 5517,08 29,48 1238,96 36529,66 360,19 36889,85
0,30 0,19 42938,89 0,35 0,22 816,16 2486,85 1598,43 751,90
2485,06 1838,93 312,62 1644,20 2442,21 517,99 1791,26 2374,31 7685,75 5871,59
9433,03 13297,00 1,23 2,26 9433,03 13297,00 1358,36 890,90
2249,25 40140,13 74240,68 24696,59 41136,20 77222,45
28978,52 15331,42 47156,58 34573,13 31290,26
57918,82 36875,96 250631,16 175513,44 307609,60
397473,73 44295,78 26630,74 70926,52 0,58 1,03
36889,85 42938,89 70926,52 126129,57 745565,63
3,88 4,51 7,46 13,26 70,89 1238,96 2561,32 2249,25 2239,74 9536,81
3496,79 12,99 26,86 23,49 36,67 0,17 9008,78 887,99 115,36 28708,62
4205,61 577,27 9935,22 33106,65 7144,95 1029595,42 8,41 0,58
40995,53 1070590,95 8,74 48140,48 3,45 4,01 6,62
11,78 4,50 69,64 100,00
AFRO Benin Africa Western Africa 4826125,00
4714406,00 38,27 18,31 43,42 1804203,18 863207,74
2046995,09 324,76 187,32 370,51 882,58
802344,33 1470964,60 1136654,47 2689,87 3572,45 716,07
71932,86 43483,67 363,31 25515,69 6343,31 352,76 46417,17
37140,37 4,00 3,69 15652,00 4714406,00 882,58 882,58
18,72 2689,87 57,06 1136654,47 52,77 26,30 46574,47 70737,64
298612,86 276765,91 52549,84 276765,91 415924,98
394078,03 169861,95 394078,03 8822,43 1380,89 8,82 2,49
991,41 6,33 14,90 85,10 3320,04 45,99 1361,31 13,39 1347,92 29,31
477,08 13982,77 138,90 14121,68 0,30 0,05 25690,31
0,54 0,09 270,56 877,73 1101,81 285,24 880,59 1088,63 137,36 634,71
1409,91 212,03 698,33 1515,05 3044,27 2657,43 3460,59 4878,12 x x
x x x x 13414,61 26070,04 16116,77
16084,44 27444,25 16371,19 6780,06 18196,95 19097,81
12971,49 22409,74 16909,87 93842,66 72360,80
x x x x 0,54 14121,68 25690,31
0,00 25515,69 x x x x x x 477,08
986,28 0,00 882,58 4558,73 2212,79 10,47 21,63 19,36 48,54 0,17
2275,40 366,27 99,25 7040,06 1735,09 378,66 2674,76 8901,37 1921,64
395999,66 8,40 0,58 0,00 395999,66 8,40 1921,64 3,57
6,49 0,00 6,44 0,49 x 16,98
AFRO Botswana Africa Southern Africa 1076493,00
743967,00 19,90 17,62 62,49 148049,43 131086,99
464904,98 26,65 28,45 84,15 139,24
126584,16 232070,96 179327,56 424,48 563,72 220,40
13644,51 6430,52 66,07 3979,96 582,59 154,32 9664,54 5847,92 1,53
3,69 15782,00 743967,00 139,24 139,24 18,72 424,48 57,06
179327,56 51,42 24,73 7159,71 10497,88 17312,87 40014,58
7075,70 40014,58 34970,46 57672,17 24733,28 57672,17
4700,54 741,84 4,70 2,37 367,26 2,33 23,98 76,02 21213,31
74,16 1802,70 19,69 1783,01 24,04 75,29 1810,10 19,99 1830,09 0,25
0,04 3633,08 0,49 0,08 118,57 393,72 256,32 79,38 294,42 245,86
48,26 215,12 376,87 49,66 235,33 451,57 1053,48 824,52 771,90 1088,09
0,73 1,32 771,90 1088,09 111,15 72,90 184,06 5618,11 11765,14
3986,08 4313,39 8900,68 3952,68 2331,20 6261,19 5051,29 3031,00 7387,17 6558,59
33243,57 22880,52 24358,18 30194,62 3507,58 2023,04
5530,62 0,74 0,53 1830,09 3633,08 5530,62 3979,96 42698,42 5,23
10,39 15,82 11,38 57,18 75,29 155,64 184,06 139,24 535,31 165,14
14,06 29,08 26,01 30,85 0,17 501,81 29,36 4,25 1885,18 454,63
84,30 532,59 2367,91 481,48 58153,65 7,82 0,58 3196,70 61350,35

8,25   3678,18 2,98   5,92   9,01   6,49   6,00   69,60   100,00
AFRO   Burkina Faso   Africa Western Africa   7472712,00
4306498,00   26,21   25,16   48,64   1128733,13   1083514,90
2094680,63   339,00   203,17 235,12 379,14 817,43
743119,90   1362386,48   1052753,19   2457,13 3274,56 854,74
106648,43   72651,10   409,53 27434,57   5202,41 445,21 79213,86
67448,69   5,73   3,69   17309,00   4306498,00   817,43 817,43
18,98   2457,13 57,06   1052753,19   54,29   26,43   44375,78   64932,44
382635,95   250291,92   77832,80   250291,92   491944,18
359600,14   187141,03   359600,14   11423,30   1977,26 11,42
2,54   1537,92 8,89   11,13   88,87   4019,28 62,68   1860,05 18,60   1841,45
29,38   435,80 12803,27   129,32 12932,59   0,30   0,05   46093,67
1,07   0,19   591,09 2071,60 2641,93 523,67 1623,87 1503,21 307,62 1228,27
2633,87 413,62 1233,34 1784,80 6191,95 4655,75 5358,33 7553,22 0,87   1,62
5358,33 7553,22 771,60 506,07 1277,66 28914,57   61477,04   39188,81
28502,87   50423,32   22597,96   15418,57   35181,42
37325,62   24099,04   39561,32   25184,56   189913,39
135097,08   164345,50   219173,80   23665,75   14684,64
38350,40   0,89   0,64   12932,59   46093,67   38350,40
27434,57   234788,92   2,63   9,37   7,80   5,58   74,63   435,80
900,94 1277,66 817,43 2897,84 743,66 15,04   31,09   28,21   25,66   0,17
3813,37 727,49 215,48 9834,90 2760,40 501,75 4612,69 12762,55   2884,29
362484,43   8,42   0,58   22166,53   384650,96   8,93   25050,82
3,36   11,98   9,97   7,13   6,51   61,04   100,00
AFRO   Burundi Africa Eastern Africa   4987390,00
3678471,00   86,21   3,35   10,45   3171209,85   123228,78
384400,22   570,82 26,74   69,58   667,13
606486,23   1111891,43   859188,83   2098,80 2765,94 1368,48
115717,23   57047,52   856,12 62447,25   17472,74   512,37
53269,98   39574,78   2,89   3,69   3258,00 3678471,00   667,13
667,13 18,14   2098,80 57,06   859188,83   52,53   26,73   35046,65
56099,65   175725,82   220888,76   53190,51   220888,76
266872,13   312035,07   144336,81   312035,07   7254,97 236,37
7,25   2,48   155,64 4,78   18,23   81,77   885,69 49,47   1443,24 15,84
1427,40 28,85   372,25 10740,80   119,19 10859,99   0,30   0,01
27095,06   0,74   0,02   364,23 1190,54 875,74 370,17 1196,29 937,58
161,31 578,36 858,48 249,23 787,40 1022,58 3725,68 2403,32 3576,22 5041,12
0,96   2,10   3576,22 5041,12 514,98 337,76 852,73 18217,72   35668,06
13317,07   20433,35   36035,19   14462,43   8172,41 17027,09
11575,91   14963,64   24746,51   15241,29   119614,16
73848,71   114815,84   154902,42   16533,48   10378,46
26911,94   0,73   1,70   10859,99   27095,06   26911,94
62447,25   184720,81   4,07   10,15   10,08   23,40   52,29   372,25
769,56 852,73 667,13 2682,76 873,82 13,88   28,69   24,87   32,57   0,17
2350,44 494,84 78,66   5468,42 1301,63 196,29 2871,50 6835,47 1611,36
313646,42   8,53   0,58   15555,10   329201,53   8,95   17166,46
3,30   8,23   8,17   18,97   5,21   x   43,89
AFRO   Cameroon   Africa Middle Africa   11351501,00
10975051,00   43,49   14,42   42,09   4773049,68   1582602,35
4619398,97   859,15 343,42 836,11 2038,68
1853349,88   3397808,11   2625578,99   6261,97 8300,65 1495,09
167494,62   110933,90   608,94 40990,71   8459,53 886,15
126503,90   102474,37   4,26   3,69   39802,00   10975051,00
2038,68 2038,68 18,58   6261,97 57,06   2625578,99   53,42   27,12
108911,98   169803,48   752452,00   655699,77   130796,25
655699,77   1031167,46   934415,23   409511,71   934415,23

9395,56 3739,62 9,40 2,54 2728,83 6,86 13,31 86,69 3626,59 158,47
4557,68 45,45 4512,24 28,47 1110,64 31624,04 318,54 31942,57
0,29 0,12 73141,91 0,67 0,27 737,21 2430,93 2725,98 938,96
2323,57 2287,13 331,50 1558,50 2834,73 645,53 1670,00 2723,45 8101,71 6058,26
8139,63 11473,79 1,00 1,89 8139,63 11473,79 1172,11 768,74
1940,85 36509,01 71781,63 40167,68 51705,19 73489,41
34713,58 16527,79 44660,26 38803,79 38182,29
54397,03 39006,61 258445,66 179704,17 259655,27
340343,13 37390,36 22802,99 60193,35 0,55 0,37
31942,57 73141,91 60193,35 40990,71 728146,69
3,10 7,09 5,84 3,98 80,00 1110,64 2296,04 1940,85 2038,68 8655,84
3210,48 12,83 26,53 23,55 37,09 0,17 6949,35 977,73 243,41 21630,51
5240,17 1092,62 8008,21 27234,88 5850,35 940265,59 8,57 0,58
34791,76 975057,34 8,88 40642,11 3,28 7,50 6,17
4,20 4,17 x 25,32
AFRO Cape Verde Africa Western Africa 237728,00
206345,00 10,60 21,80 67,60 21872,57 44983,21
139489,22 3,94 9,76 25,25 38,95
35405,43 64909,95 50157,69 117,73 156,68 25,07
2813,16 1983,71 9,84 719,47 218,86 15,24 2093,69 1764,85 5,37 3,69
1703,00 206345,00 38,95 38,95 18,87 117,73 57,06 50157,69
50,89 21,58 1982,08 2541,09 14504,45 10255,16 2631,38 10255,16
19027,62 14778,33 7154,55 14778,33 9221,26 157,04 9,22
2,19 119,71 7,03 14,97 85,03 8253,17 3,25 90,46 0,92 89,53
27,55 20,88 575,23 5,91 581,15 0,28 0,00 1208,32 0,59 0,01
16,01 84,98 172,16 15,14 61,11 107,25 5,41 51,78 238,53 5,96
26,69 119,34 270,38 209,13 170,46 240,29 0,63 1,15 170,46 240,29
24,55 16,10 40,65 787,00 2507,49 2104,49 867,45 1949,05 1358,71 264,11
1469,47 2656,15 424,54 943,95 1426,26 7265,39 4462,87 4580,50 5127,84 659,59
343,57 1003,16 0,49 0,35 581,15 1208,32 1003,16 719,47 11266,24
3,05 6,35 5,27 3,78 81,54 20,88 43,17 40,65 38,95 159,20
56,21 13,12 27,12 24,46 35,30 0,17 218,39 36,30 8,57 389,01
172,32 41,37 257,55 575,13 138,22 14916,55 7,23 0,58 579,82
15496,38 7,51 718,05 3,75 7,80 6,47 4,64 4,63 72,70
100,00
AFRO Central African Republic Africa Middle Africa
1910933,00 1833805,00 69,85 6,26 23,89 1280912,79
114796,19 438096,01 230,56 24,91
79,30 334,77 304336,78 557950,76 431143,77 1046,30
1381,07 925,83 58719,13 17200,20 668,89 37536,34 3443,70
256,94 21182,79 13756,50 1,85 3,69 2303,00 1833805,00
334,77 334,77 18,26 1046,30 57,06 431143,77 50,97 28,90 17062,91
30240,65 57741,52 113454,55 15480,03 113454,55
105045,08 160758,11 62783,59 160758,11 5728,26 131,92
5,73 2,58 72,52 3,15 17,88 82,12 1255,86 36,94 1140,35 10,78
1129,57 30,58 185,57 5674,60 54,16 5728,75 0,31 0,01 9562,05 0,52
0,01 305,06 810,36 275,72 277,49 848,91 335,89 109,49 480,85 543,30
184,64 573,82 593,91 2445,68 1727,87 1370,24 1931,52 0,56 1,12 1370,24
1931,52 197,31 129,41 326,73 14494,18 24818,35 4556,17 14282,65
26424,35 5555,56 5255,95 14008,74 8045,42 10158,33 18070,60
9303,70 83390,11 53276,65 46720,87 59556,04 6727,81
3990,25 10718,06 0,58 2,05 5728,75 9562,05 10718,06 37536,34
97212,91 5,45 9,10 10,20 35,73 39,51 185,57 383,64 326,73
334,77 1437,99 534,00 12,91 26,68 23,28 37,14 0,17 1677,96 193,54
18,47 4682,75 674,50 91,57 1877,65 5387,77 1206,06 161964,18 8,83
0,58 6195,04 168159,21 9,17 7401,10 3,41 5,69 6,37 22,32

4,40 57,81 100,00
AFRO Chad Africa Middle Africa 5986315,00
4296936,00 75,06 1,88 23,06 3225280,16 80782,40
990873,44 580,55 17,53 179,35 777,43
706753,00 1295713,84 1001233,42 2451,68 3229,10 1057,65
98007,01 53380,11 613,93 42029,48 9351,00 443,72 55977,53
44029,11 3,68 3,69 10093,00 4296936,00 777,43 777,43
18,09 2451,68 57,06 1001233,42 53,23 26,93 41381,14 66019,02
255116,74 255763,71 55870,39 255763,71 362516,90
363163,87 163270,55 363163,87 8436,64 851,51 8,44 2,50
599,24 5,94 14,68 85,32 2348,88 45,17 1322,78 12,77 1310,01 29,00
434,84 12610,99 122,93 12733,92 0,30 0,03 32011,89
0,74 0,08 315,61 1197,63 1598,92 407,15 1216,64 1340,15 174,37 773,67
1307,52 328,22 885,21 1248,05 4116,71 3013,32 4292,50 6050,81 1,04 2,01
4292,50 6050,81 618,12 405,40 1023,52 15732,33 35334,28 23540,06
22704,73 37822,38 20343,09 8592,77 22384,39 18085,33
19297,27 28486,42 18019,43 126221,43 90795,77
131611,26 182319,95 18952,02 12215,44 31167,46
0,73 0,98 12733,92 32011,89 31167,46 42029,48
245221,12 3,51 8,83 8,60 11,59 67,47 434,84 898,94 1023,52
777,43 3104,52 993,32 14,01 28,96 25,04 32,00 0,17 3285,21 582,23
173,10 9639,98 2120,20 392,38 3925,14 11890,98 2625,48 365789,34
8,51 0,58 18014,79 383804,13 8,93 20640,27 3,32
8,34 8,12 10,95 5,38 63,89 100,00
AFRO Comoros Africa Eastern Africa 235265,00
217136,00 34,38 18,83 46,79 74651,36 40886,71
101597,93 13,44 8,87 18,39 40,70
36998,99 67831,48 52415,23 123,89 164,59 67,00
6190,76 3684,10 34,01 2585,75 834,82 32,99 3605,01 2849,28 3,77 3,69
1219,00 217136,00 40,70 40,70 18,74 123,89 57,06 52415,23
51,48 22,90 2095,30 2837,65 11697,60 11483,36 3848,29 11483,36
16630,55 16416,31 8781,23 16416,31 7659,05 93,36 7,66
2,27 65,67 5,39 18,61 81,39 5613,99 5,63 144,25 1,71 142,54
25,32 21,97 556,32 6,67 563,00 0,26 0,00 1994,00 0,92 0,01
21,82 73,68 116,00 17,63 61,77 105,89 13,18 59,77 158,71 17,74
73,30 172,52 248,87 274,40 168,70 237,80 0,68 0,87 168,70 237,80
24,29 15,93 40,22 1083,69 2232,48 1656,82 1003,47 1916,29 1566,47 648,50
1738,06 2109,13 1062,93 2355,08 2603,11 7310,37 7375,31 4955,37 6391,46 713,57
428,23 1141,80 0,53 1,19 563,00 1994,00 1141,80 2585,75 10131,76
3,39 11,99 6,87 15,55 62,21 21,97 45,43 40,22 40,70 169,79
61,69 12,94 26,75 23,97 36,33 0,17 147,66 35,43 9,28 200,00
52,08 12,15 186,19 256,14 73,43 16489,73 7,59 0,58 659,96
17149,70 7,90 733,39 3,28 11,63 6,66 15,08 4,28 59,08
100,00
AFRO Congo, Democratic Republic of the Africa Middle Africa
29801894,00 28571000,00 64,30 9,84 25,86 18371153,00
2811386,40 7388460,60 3306,81 610,07
1337,31 5254,19 4776536,14 8756982,93 6766759,54 16301,00
21555,19 12301,06 846742,87 298974,24 8898,72
537143,51 75336,05 3402,34 309599,36 223638,19 2,60
3,69 49869,00 28571000,00 5254,19 5254,14 18,39 16301,00
57,05 6766759,54 51,90 25,27 272668,59 411849,42
1115956,45 1566153,39 268119,45 1566153,39 1800474,46
2250671,40 952637,46 2250671,40 6301,76 3142,62 6,30 2,40
1947,84 3,91 17,61 82,39 1745,44 478,11 14021,17 139,39 13881,78
29,03 2891,29 83947,62 842,94 84790,56 0,30 0,15

151654,75 0,53 0,26 2375,84 7376,81 4873,99 2099,09 7277,42 5649,26
1038,11 5984,72 10145,07 1598,39 6420,36 9794,41 22636,91 21688,07
21369,53 30122,96 0,94 1,39 21369,53 30122,96
3077,21 2018,24 5095,45 116265,55 116265,55 116265,55
114222,92 223323,48 86476,54 50497,30 171474,17
139773,86 93047,81 200694,28 144868,72 637658,19
610594,43 601957,48 848065,87 86681,88 56820,41
143502,29 0,50 1,88 84790,56 151654,75 143502,29
537143,51 1333580,29 4,71 8,42 7,97 29,83 49,06 2891,29
5977,16 5095,45 5254,14 22436,84 8314,26 12,89 26,64 23,42 37,06
0,17 29232,69 2675,05 357,95 80525,13 11903,90 2142,67
32027,06 93143,25 20778,27 2271449,67 7,95 0,58
82944,32 2354394,00 8,24 103722,60 3,60 6,44 6,10
22,81 4,41 56,64 100,00
AFRO Congo, Republic of Africa Middle Africa
2182726,00 1973000,00 33,53 21,47 44,99 661546,90
423603,10 887652,70 119,08 91,92
160,67 371,67 337877,68 619442,42 478660,05 1126,00
1497,67 375,47 27150,02 12212,59 219,85 12864,43 1888,76
155,62 14285,59 10323,83 2,61 3,69 10885,00
1973000,00 371,67 371,71 18,84 1126,00 57,07 478660,05 49,92
25,46 18557,37 28665,79 77892,62 109105,81 13517,30
109105,81 125115,79 156328,97 60740,46 156328,97
6341,40 690,26 6,34 2,39 429,73 3,95 17,38 82,62 5516,98 46,62
1294,87 13,24 1281,64 27,49 199,66 5488,91 56,70 5545,62 0,28 0,03
6669,86 0,34 0,04 202,33 617,24 491,03 151,89 518,18 492,83 124,30
526,45 835,43 161,11 486,96 721,83 1817,60 1817,92 1565,13 2206,24 0,86
1,21 1565,13 2206,24 225,38 147,82 373,20 9695,40 18573,02 7445,63
8154,05 16148,44 7645,03 5897,71 15163,86 11694,93 9079,38
15753,21 11046,06 57601,12 53474,50 49600,13
64897,13 7142,42 4348,11 11490,53 0,58 0,65 5545,62 6669,86
11490,53 12864,43 119758,54 4,43 5,33 9,18 10,28
70,77 199,66 412,61 373,20 371,71 1537,12 553,14 12,99 26,84 24,18
35,99 0,17 1742,88 204,70 31,68 5528,43 837,92 143,65 1958,14 6414,24
1389,82 157718,78 7,99 0,58 6641,52 164360,31 8,33 8031,34
3,37 4,06 6,99 7,83 4,89 72,86 100,00
AFRO Côte d'Ivoire Africa Western Africa 8047578,00
7704281,00 40,15 12,86 46,99 3093268,82 990770,54
3620241,64 556,79 215,00 655,26 1427,05
1297317,57 2378415,55 1837866,56 4395,78 5822,83 1323,57
142933,16 89481,76 674,80 47957,99 12480,08 648,77
94975,17 77001,67 4,28 3,69 61349,00 7704281,00
1427,05 1427,05 18,52 4395,78 57,06 1837866,56 52,58 27,70 75027,91
121780,51 548122,10 476338,64 103394,29 476338,64
744930,52 673147,06 300202,71 673147,06 9669,05 5931,86
9,67 2,55 4364,68 7,11 13,61 86,39 7962,98 111,41 3295,56 32,06
3263,50 29,29 779,65 22837,95 224,36 23062,31 0,30 0,18
52459,19 0,68 0,42 838,99 2418,58 2482,38 852,70 2019,97 1872,49
335,15 1321,98 2252,51 495,76 1243,15 1905,88 7581,87 4782,18 5770,54 8134,28
0,76 1,70 5770,54 8134,28 830,96 545,00 1375,95 41112,45 72364,19
36419,79 47765,89 64847,41 28623,91 16571,35
38316,15 30551,60 30767,17 41624,70 27502,77
247771,17 146630,83 188577,87 249412,71 27155,21
16710,65 43865,86 0,57 0,62 23062,31 52459,19
43865,86 47957,99 505801,71 3,10 7,04 5,89 6,44
77,54 779,65 1611,78 1375,95 1427,05 6058,66 2240,18 12,87 26,60 23,55

36,97 0,17 4527,69 772,94 176,54 13510,88 3299,96 573,72 5359,48
17002,08 3712,02 676859,08 8,79 0,58 25354,47
702213,55 9,11 29066,49 3,28 7,47 6,25 6,83 4,14
72,03 100,00
AFRO Equatorial Guinea Africa Middle Africa
487562,00 487562,00 39,51 19,35 41,14 192635,75 94343,25
200583,01 34,67 20,47 36,31 91,45
83138,58 152420,74 117779,66 278,19 369,64 73,88
6044,68 2860,16 47,45 3142,16 570,65 26,43 2902,53 2289,51 3,73 3,69
10022,00 487562,00 91,45 91,45 18,76 278,19 57,06
117779,66 54,19 23,19 4956,08 6451,43 25194,78 24936,11
3389,34 24936,11 36602,29 36343,62 14796,85 36343,62
7507,21 752,37 7,51 2,34 517,89 5,17 16,55 83,45 20555,33
7,64 203,49 2,12 201,38 26,36 49,34 1300,52 13,69 1314,22 0,27
0,03 1451,28 0,30 0,03 22,33 57,59 67,90 19,59 54,45 74,92
10,49 55,57 135,29 16,16 58,07 122,89 201,57 226,35 349,61 492,81
1,73 2,18 349,61 492,81 50,34 33,02 83,36 1153,02 1695,98 989,50
1215,27 1692,70 1127,88 518,57 1571,50 1811,54 1059,70 1905,58 1845,72 6462,76
6274,45 11209,02 13660,97 1614,10 915,29 2529,38 0,52 0,64
1314,22 1451,28 2529,38 3142,16 27906,58 3,59 3,96 6,91 8,58
76,95 49,34 102,00 83,36 91,45 388,28 145,48 12,71 26,27 23,55
37,47 0,17 576,61 31,43 5,72 1393,08 176,88 30,65 609,95 1580,18
363,56 36707,18 7,53 0,58 1461,98 38169,16 7,83 1825,55
3,44 3,80 6,63 8,23 4,78 73,11 100,00
AFRO Eritrea Africa Eastern Africa 1612622,00
1510458,00 63,12 7,29 29,59 953401,09 110112,39
446944,52 171,61 23,89 80,90 276,40
251275,95 460672,57 355974,26 861,81 1138,22 551,24
46214,56 21653,15 351,25 24875,44 5840,82 199,99 21339,12
15812,32 2,86 3,69 2065,00 1510458,00 276,40 276,40 18,30
861,81 57,06 355974,26 54,19 27,64 14978,69 23816,38
72735,63 92591,34 20408,57 92591,34 111530,70
131386,40 59203,64 131386,40 7383,90 152,48 7,38 2,57
99,44 4,82 17,55 82,45 1367,14 38,90 1172,60 12,54 1160,06 29,82
152,85 4558,32 49,27 4607,60 0,31 0,01 9375,98 0,62 0,01 293,28
655,41 310,93 286,93 645,66 336,67 92,65 370,42 549,87 170,93 594,66
717,08 2097,15 1650,97 1156,34 1630,00 0,55 0,99 1156,34 1630,00 166,51
109,21 275,72 14486,11 20061,30 4927,71 15425,23 19975,20
5413,33 4532,70 10853,95 7736,55 9638,77 18698,82 11103,25
73394,85 50004,17 40468,85 49368,94 5827,51 3307,72
9135,23 0,60 1,65 4607,60 9375,98 9135,23 24875,44 83392,15
4,13 8,41 8,19 22,30 56,97 152,85 316,00 275,72 276,40 1178,49
433,24 12,97 26,81 23,45 36,76 0,17 1516,88 265,02 30,12 3246,48
893,33 136,89 1791,94 4185,44 992,25 132378,65 8,76 0,58 5280,16
137658,81 9,11 6272,41 3,35 6,81 6,64 18,07 4,56 60,58
100,00
AFRO Ethiopia Africa Eastern Africa 53021347,00
51939000,00 66,63 9,32 24,05 34606955,70 4840714,80
12491329,50 6229,25 1050,44 2260,93 9540,62
8673288,89 15901029,63 12287159,26 29634,00 39174,62
8679,85 793473,40 397694,27 6111,18 493546,60 157576,05
2568,67 299926,79 240118,22 4,01 3,69 107645,00
51939000,00 9540,62 9540,62 18,37 29634,00 57,06 12287159,26
54,98 23,28 524508,94 689993,30 3016174,67 2795342,29
486122,06 2795342,29 4230676,92 4009844,54 1700624,30
4009844,54 8145,47 8768,19 8,15 2,34 6251,10 5,81 18,21 81,79

2072,53 413,06 11389,91 129,13 11260,78 27,26 5256,05
143289,56 1643,13 144932,70 0,28 0,30 148684,11 0,29
0,31 1940,92 5634,09 8282,11 1830,44 5422,97 9055,69 962,69 3455,39 8133,93
1262,44 4187,85 8723,84 20607,68 15487,63 38019,10 53592,56
1,84 3,46 38019,10 53592,56 5474,75 3590,70 9065,45 99404,13
168958,15 122018,49 107681,10 162988,00 131623,67
49300,44 100596,81 111336,40 82478,94 132022,36
127767,89 623578,76 444099,98 1150440,44 1536739,85
165663,42 102961,57 268624,99 0,52 0,95 144932,70
148684,11 268624,99 493546,60 2954056,14 3,43 3,51
6,35 11,67 75,04 5256,05 10865,92 9065,45 9540,62 40975,09
15312,50 12,83 26,52 23,28 37,37 0,17 24395,12 4932,56
1197,71 44981,96 11631,57 2944,22 29726,92 57594,94
14495,43 4024339,97 7,75 0,58 155265,25 4179605,21
8,05 169760,68 3,47 3,56 6,43 11,81 4,06 70,68 100,00
AFRO Eswatini Africa Southern Africa 291000,00
291000,00 12,15 23,39 64,46 35356,50 68064,90
187578,60 6,36 14,77 33,95 55,09
50078,16 91809,97 70944,07 198,00 253,09 95,42
5107,19 2073,34 23,80 1375,68 154,14 71,62 3731,51 1919,21 1,06 3,69
3962,00 291000,00 55,09 55,09 18,93 198,00 68,04 70944,07
51,32 25,31 2827,22 5010,58 5662,89 18869,46 2275,95 18869,46
13500,68 26707,26 10113,74 26707,26 4639,41 183,81
4,64 2,69 77,10 1,95 23,58 76,42 13615,12 32,87 831,58
8,81 822,78 25,03 29,45 737,13 7,89 745,02 0,26 0,01 973,52
0,33 0,01 53,43 148,41 87,40 39,09 132,36 98,12 14,86 76,31
168,41 18,25 93,09 215,84 435,14 330,59 208,66 294,13 0,48 0,89
208,66 294,13 30,05 19,71 49,75 2546,76 4484,96 1408,91 2131,21 4000,24
1607,20 729,78 2212,60 2358,11 1154,59 2876,03 3259,78 14168,54 8845,62
6794,23 7870,17 978,37 527,30 1505,67 0,52 0,47 745,02 973,52 1505,67
1375,68 22107,37 5,52 7,21 11,15 10,19 65,93 29,45 60,89
49,75 55,09 264,22 118,80 11,15 23,05 20,85 44,96 0,17 168,94
4,11 0,73 617,11 133,74 30,52 173,29 761,03 155,10 26862,36
9,23 0,58 870,28 27732,63 9,53 1025,37 2,69 3,51 5,43
4,96 3,70 79,72 100,00
AFRO Gabon Africa Middle Africa 721991,00
580155,00 29,96 10,74 59,30 173814,44 62308,65
344031,92 31,29 13,52 62,27 107,08
97343,05 178462,25 137902,65 331,02 438,09 105,26
7409,88 3450,83 56,96 3410,65 590,42 48,30 3999,23 2860,40 2,51 3,69
15593,00 580155,00 107,08 107,08 18,46 331,02 57,06
137902,65 49,51 23,58 5301,71 7804,27 20711,95 29944,58
3970,33 29944,58 33817,93 43050,56 17076,31 43050,56
5829,12 908,93 5,83 2,26 556,68 3,57 18,96 81,04 26877,30
20,79 523,46 5,57 517,89 24,91 58,71 1462,49 15,73 1478,22 0,25
0,04 1847,48 0,32 0,05 60,93 260,60 188,83 45,50 218,15 188,45
19,86 116,56 339,92 25,24 105,31 272,81 710,95 471,22 517,71 729,77
0,73 1,55 517,71 729,77 74,55 48,89 123,44 2952,91 7665,98 2834,93
2489,28 6688,13 2905,18 953,01 3323,48 4300,18 1613,98 3504,96 3896,13 21709,68
12127,53 15808,85 18781,67 2276,47 1258,37 3534,85 0,61
0,59 1478,22 1847,48 3534,85 3410,65 32779,36 4,37 5,46 10,45
10,09 69,63 58,71 121,37 123,44 107,08 436,02 148,86 13,46 27,84
24,56 34,14 0,17 697,47 88,49 13,06 1970,12 355,98 77,75 790,31
2352,02 521,63 43572,18 7,51 0,58 2043,14 45615,32 7,86
2564,77 3,24 4,05 7,75 7,48 5,62 71,86 100,00
AFRO Gambia Africa Western Africa 777591,00

444972,00 27,03 15,01 57,96 120275,93 66790,30
257905,77 21,65 14,49 46,68 82,82
75294,64 138040,18 106667,41 253,88 336,71 112,68
10427,07 6052,81 54,45 3902,02 976,72 58,23 6525,05 5076,09 3,50
3,69 1902,00 444972,00 82,82 82,82 18,61 253,88 57,06
106667,41 53,72 24,88 4449,63 6317,87 23618,81 24828,48
6561,77 24828,48 34386,31 35595,97 17329,26 35595,97
7727,75 146,98 7,73 2,42 100,96 5,31 17,26 82,74 4274,43 6,24
175,19 1,85 173,34 27,78 45,03 1250,87 13,35 1264,22 0,28 0,01
3350,62 0,75 0,01 60,76 228,41 324,02 62,13 169,75 223,53 34,85
164,01 393,61 45,76 136,07 306,79 703,57 614,16 557,57 785,97 0,79
1,28 557,57 785,97 80,29 52,66 132,95 3014,05 6733,63 4659,55 3562,94
5322,47 3324,04 1684,44 4702,16 5193,60 2798,51 4428,25 4277,52 21294,28
16770,40 16875,52 21461,70 2430,07 1437,93 3868,01 0,87
0,88 1264,22 3350,62 3868,01 3902,02 23211,11 3,68 9,74 11,25
11,35 63,98 45,03 93,09 132,95 82,82 296,85 75,90 15,17 31,36
27,90 25,57 0,17 392,93 58,35 19,21 2073,58 462,39 111,50 457,68
2573,14 503,12 36099,09 8,11 0,58 2235,71 38334,80 8,62
2738,82 3,30 8,74 10,09 10,18 7,14 60,55 100,00
AFRO Ghana Africa Western Africa 12919340,00
9580143,00 29,75 21,05 49,21 2850092,54 2016620,10
4714388,37 513,02 437,61 853,30 1803,93
1639934,10 3006545,86 2323239,98 5466,08 7270,01 1723,50
159467,57 100530,40 536,41 37209,86 9008,42 1187,09
122257,71 91521,99 2,98 3,69 72354,00 9580143,00
1803,93 1803,93 18,83 5466,08 57,06 2323239,98 52,57 25,89 94840,49
141525,66 451717,01 553194,01 121816,99 553194,01
688083,17 789560,17 358183,15 789560,17 7182,39 5196,75
7,18 2,47 3411,59 4,72 18,19 81,81 7552,50 145,72 4379,44 44,27
4335,17 29,75 969,48 28841,95 294,53 29136,48 0,30 0,22
58612,12 0,61 0,44 847,57 2859,91 3256,32 996,83 2713,62 2729,61
636,54 2753,88 5534,05 1108,39 3511,78 6017,64 9413,25 11861,16 9263,85
13058,52 0,98 1,10 9263,85 13058,52 1333,99 874,92 2208,92
41287,80 84560,94 47745,52 55780,28 85975,10
41342,31 31047,35 78903,26 74370,60 62431,73
113815,17 85777,60 297300,06 339580,25 292581,51
373860,39 42131,74 25048,65 67180,38 0,70 0,39
29136,48 58612,12 67180,38 37209,86 597421,33
4,23 8,52 9,76 5,41 72,08 969,48 2004,22 2208,92 1803,93 7065,31
2287,69 13,72 28,37 25,53 32,38 0,17 8532,30 1155,62 310,44 26154,44
6498,19 1466,02 9791,41 33141,30 7126,83 796687,00 8,32 0,58
38830,26 835517,26 8,72 45957,09 3,49 7,02 8,04
4,45 5,50 71,50 100,00
AFRO Guinea Africa Western Africa 4433672,00
4250460,00 60,65 5,79 33,56 2577903,99 246101,63
1426454,38 464,02 53,40 258,19 775,62
705104,56 1292691,69 998898,13 2425,16 3200,77 1017,58
89546,33 47652,56 574,78 39073,91 8829,39 442,80 50472,42
38823,17 3,33 3,69 15681,00 4250460,00 775,62 775,62
18,25 2425,16 57,06 998898,13 52,62 26,31 40812,16 63801,09
224542,65 247642,21 50737,60 247642,21 329155,91
352255,46 155350,86 352255,46 7744,01 1214,34 7,74 2,46
828,39 5,28 16,02 83,98 3689,25 44,50 1277,00 12,84 1264,15 28,41
430,13 12219,13 124,11 12343,24 0,29 0,05 27677,98
0,65 0,10 275,33 1127,72 1606,08 325,18 1085,16 1346,41 202,50 893,98
1720,51 327,94 925,79 1632,93 3797,55 3468,01 3179,18 4481,44 0,84 1,29

3179,18 4481,44 457,80 300,26 758,06 13671,74 32852,40 22764,04
18084,25 33492,19 19694,47 9973,06 25654,14 22483,74
19002,13 29407,36 22085,77 112253,42 98893,20
93974,75 127791,80 13532,36 8562,05 22094,41 0,52
0,92 12343,24 27677,98 22094,41 39073,91
251065,92 3,75 8,41 6,71 11,87 69,26 430,13 889,22 758,06
775,62 3331,94 1236,97 12,91 26,69 23,28 37,12 0,17 2000,91 373,20
123,29 7360,16 1867,75 400,15 2415,20 9361,30 1954,90 354210,36 8,33
0,58 12770,57 366980,93 8,63 14725,47 3,36 7,54
6,02 10,65 4,01 68,41 100,00
AFRO Guinea-Bissau Africa Western Africa 800827,00
778444,00 60,48 8,61 30,91 470802,93 67024,03
240617,04 84,74 14,54 43,55 142,84
129854,93 238067,38 183961,15 444,15 586,99 111,48
10276,16 5901,63 44,71 2880,50 478,30 66,77 7395,66 5423,34 2,75
3,69 1432,00 778444,00 142,84 142,84 18,35 444,15 57,06
183961,15 53,73 29,54 7675,44 13119,13 37602,18 49999,88
6951,58 49999,88 58396,75 70794,46 27746,15 70794,46
7501,73 107,42 7,50 2,67 69,17 4,83 15,76 84,24 1839,57 9,32
280,42 2,77 277,65 29,79 78,78 2346,79 23,41 2370,21 0,30 0,00
3728,15 0,48 0,01 77,88 233,98 175,92 83,24 201,31 137,40 50,12
168,25 230,22 76,39 161,43 197,39 700,84 598,73 574,24 809,45 0,82
1,35 574,24 809,45 82,69 54,23 136,92 3817,39 6960,61 2709,23 4538,73
6299,02 2178,84 2434,55 4888,74 3263,11 4326,27 5248,06 2921,23 23245,12
18959,07 19045,82 25631,87 2742,60 1717,34 4459,93 0,57
0,37 2370,21 3728,15 4459,93 2880,50 57355,67 4,06 6,38 7,64
4,93 76,99 78,78 162,85 136,92 142,84 612,92 228,45 12,85 26,57
23,30 37,27 0,17 360,44 48,97 10,88 1261,86 289,29 52,14 413,04
1568,53 328,94 71123,40 9,14 0,58 2577,84 73701,24 9,47
2906,78 3,22 5,06 6,05 3,91 3,94 77,82 100,00
AFRO Kenya Africa Eastern Africa 23733381,00
23116127,00 54,34 6,22 39,44 12561303,41 1437823,10
9117000,49 2261,03 312,01 1650,18 4223,22
3839290,29 7038698,86 5438994,57 13189,22 17412,44
3711,29 381758,76 227489,78 2075,78 164689,45 51858,68
1635,52 217069,32 175631,10 4,24 3,69 98843,00
23116127,00 4223,22 4223,22 18,27 13189,22 57,06 5438994,57
54,36 25,34 229557,12 334167,79 1521839,37 1340167,37
281138,94 1340167,37 2085564,28 1903892,27 844863,85
1903892,27 9022,12 8917,73 9,02 2,44 6507,28 6,58 16,07 83,93
4275,93 247,29 6942,39 78,15 6864,24 27,76 2339,27 64933,17 739,27
65672,44 0,28 0,28 107525,36 0,47 0,46 1052,93 3362,24
3158,72 1267,67 4005,39 4044,83 463,31 1657,79 4021,67 827,39 2582,52 5122,36
12089,40 8579,02 17018,09 23989,07 1,41 2,80 17018,09
23989,07 2450,60 1607,27 4057,87 53781,03 103432,40 49940,17
73100,45 122651,83 63332,73 23551,64 49956,76
54121,62 53132,39 84203,37 76146,47 390723,34
254266,86 550016,02 710993,24 79202,31 47636,55
126838,85 0,55 0,71 65672,44 107525,36 126838,85
164689,45 1439166,17 3,15 5,16 6,08 7,90 77,72 2339,27
4836,02 4057,87 4223,22 18190,59 6792,08 12,86 26,59 23,22 37,34
0,17 16609,10 3359,55 595,70 33817,99 8155,12 1676,04 20167,21
42531,79 10408,03 1914300,31 8,28 0,58 73312,86
1987613,17 8,60 83720,89 3,30 5,41 6,38 8,29 4,21
72,41 100,00
AFRO Lesotho Africa Southern Africa 974540,00

742055,00 44,30 13,31 42,39 328730,37 98767,52
314557,11 59,17 21,43 56,93 137,54
125035,32 229231,43 177133,37 423,39 560,93 333,34
15664,23 5100,42 78,81 4489,68 422,11 254,53 11174,54 4678,30
0,72 3,69 1845,00 742055,00 137,54 137,54 18,53 423,39 57,06
177133,37 51,61 25,52 7098,94 10805,96 8518,66 40661,78
5415,00 40661,78 26423,56 58566,68 23319,90 58566,68
3560,86 65,70 3,56 2,41 21,18 1,15 29,65 70,35 2486,34 100,05
2647,21 26,97 2620,24 26,19 75,09 1966,64 20,24 1986,89 0,27 0,00
2429,99 0,33 0,01 109,51 354,02 182,08 101,13 398,45 259,83 34,77
211,11 378,94 51,15 324,81 618,40 1110,41 954,29 698,80 985,04 0,63
1,03 698,80 985,04 100,63 66,00 166,62 5186,10 10640,09 2930,65
5370,97 11934,27 4200,38 1683,79 6101,36 5241,02 2958,77 9736,50 8994,11
35508,43 25225,46 22346,03 26038,32 3217,83 1744,57
4962,40 0,67 0,61 1986,89 2429,99 4962,40 4489,68 44697,72 7,52
9,20 18,78 16,99 47,51 75,09 155,24 166,62 137,54 549,55 181,67
13,66 28,25 25,03 33,06 0,17 508,70 32,62 4,90 1893,46 479,97
103,33 542,96 2407,87 489,84 59056,52 7,96 0,58 2868,26 61924,78
8,35 3358,10 3,21 3,92 8,01 7,25 5,42 72,18 100,00
AFRO Liberia Africa Western Africa 2226993,00
2162710,00 42,62 10,15 47,23 921747,00 219515,07
1021447,93 165,91 47,63 184,88 398,43
362210,28 664052,18 513131,23 1233,96 1632,40 254,09
28223,13 17061,88 165,66 11730,52 2910,16 88,44 16492,62
14151,72 6,05 3,69 2950,00 2162710,00 398,43 398,43 18,42
1233,96 57,06 513131,23 53,24 26,47 21214,40 32662,42
204457,80 127678,20 21151,14 127678,20 258334,61
181555,02 75027,96 181555,02 11944,95 352,38 11,94
2,49 278,89 9,45 10,92 89,08 1364,03 15,27 467,96 4,62 463,34
30,34 218,86 6640,87 66,22 6707,08 0,31 0,01 9912,15 0,46 0,01
126,33 339,78 491,90 127,42 272,07 365,68 86,23 303,94 540,05 116,01
256,87 437,40 1151,46 1088,86 1596,87 2250,98 1,39 2,07 1596,87 2250,98
229,95 150,82 380,77 6171,41 10207,82 6672,31 7134,36 8699,07 5101,75
4164,49 8825,22 7250,98 6848,85 8392,36 6127,14 36137,35 32690,29
50116,19 67580,11 7216,73 4527,87 11744,60 0,54 0,54
6707,08 9912,15 11744,60 11730,52 141460,67 2,60 3,84
4,55 4,54 84,48 218,86 452,45 380,77 398,43 1704,08 634,34 12,84
26,55 23,38 37,22 0,17 745,89 109,58 30,28 4347,81 953,30 204,93
865,56 5369,42 1035,01 182590,03 8,44 0,58 6788,38 189378,40
8,76 7823,38 3,54 5,23 6,20 6,19 4,13 74,70 100,00
AFRO Madagascar Africa Eastern Africa 13818212,00
13587810,00 64,12 8,73 27,15 8712503,77 1186215,81
3689090,42 1568,25 257,41 667,73 2493,38
2266713,52 4155641,46 3211177,49 7752,71 10246,10
2752,45 261423,15 142064,02 1543,65 120936,72 36577,89
1208,80 140486,44 105486,12 3,01 3,69 13721,00
13587810,00 2493,38 2493,38 18,35 7752,71 57,06 3211177,49 54,65
28,95 136260,92 224476,69 735624,35 888463,21
164568,71 888463,21 1096361,96 1249200,82 525306,32
1249200,82 8068,72 1107,11 8,07 2,65 742,84 5,41 17,82 82,18
1009,80 91,00 2797,80 30,06 2767,74 30,41 1375,04 41821,44 454,22
42275,66 0,31 0,04 64397,18 0,47 0,07 1269,83 3009,23
2344,01 1573,34 3455,05 2339,91 457,26 1552,76 2080,89 1433,03 3453,15 3345,28
10868,75 8704,93 9908,39 13967,08 0,91 1,60 9908,39 13967,08
1426,81 935,79 2362,60 63214,08 91527,75 34214,76 86667,23
107689,00 36722,97 22265,11 46256,40 28136,68

78727,54 110895,08 52100,26 372743,97 284889,78
339808,28 457106,38 48932,39 30626,13 79558,52
0,59 0,89 42275,66 64397,18 79558,52 120936,72
942032,74 3,86 5,87 7,26 11,03 71,98 1375,04 2842,65 2362,60
2493,38 10726,14 4015,07 12,82 26,50 23,25 37,43 0,17 6313,65
1186,85 173,57 13577,38 3358,41 473,15 7558,36 17093,50 4092,21
1253293,03 9,22 0,58 45984,82 1299277,85 9,56 50077,03
3,25 4,96 6,12 9,31 3,85 72,50 100,00
AFRO Malawi Africa Eastern Africa 8040477,00
3524411,00 76,36 5,37 18,27 2691240,24 189260,87
643909,89 484,42 41,07 116,55 642,04
583673,22 1070067,57 826870,40 2010,90 2652,94 1447,38
139672,59 73209,25 994,58 77586,07 22724,61 452,79
62086,52 50484,65 4,35 3,69 11962,00 3524411,00
642,04 642,04 18,22 2010,90 57,06 826870,40 55,16 25,62 35415,12
51525,00 238876,54 205040,50 65154,23 205040,50
325816,65 291980,61 152094,34 291980,61 9244,57 1105,84
9,24 2,47 810,76 6,78 15,37 84,63 3394,04 63,98 1741,39 20,04
1721,35 26,90 356,66 9595,71 111,71 9707,42 0,28 0,03 33545,97
0,95 0,11 543,34 1379,13 1167,27 515,69 1415,55 1433,70 170,72 662,98
1547,26 239,77 891,69 2059,88 4720,69 3167,54 5765,45 8127,10 1,22 2,57
5765,45 8127,10 830,22 544,52 1374,74 27514,72 41435,90 18646,19
28950,98 42651,52 22512,50 8908,40 19332,70 20997,03
16008,62 27994,65 30231,94 154272,69 89320,70
188415,31 229174,43 27131,80 15354,69 42486,49
1,21 2,20 9707,42 33545,97 42486,49 77586,07
128654,66 2,98 10,30 13,04 23,81 49,87 356,66 737,33 1374,74
642,04 2015,53 279,50 17,70 36,58 31,85 13,87 0,17 3418,23 471,02
94,22 8204,88 1728,16 399,86 3920,65 10066,33 2321,84 294302,45
8,35 0,58 24557,19 318859,64 9,05 26879,03 3,04
10,52 13,32 24,33 8,43 40,35 100,00
AFRO Mali Africa Western Africa 7179722,00
5948937,00 62,44 7,56 30,00 3714516,26 449739,64
1784681,10 668,61 97,59 323,03 1089,23
990212,46 1815389,51 1402800,99 3394,25 4483,48 1624,44
142918,83 73865,05 887,45 64132,64 16008,17 736,99
78786,19 57856,89 2,76 3,69 17394,00 5948937,00
1089,23 1089,23 18,31 3394,25 57,06 1402800,99 54,23 28,40 59066,73
96391,04 286110,97 375786,81 77504,90 375786,81
441568,73 531244,58 232962,66 531244,58 7422,65 1291,10
7,42 2,61 836,56 4,81 17,83 82,17 2923,88 44,08 1325,95 13,75
1312,20 29,77 602,01 17921,05 187,79 18108,84 0,30 0,05
37989,06 0,64 0,11 240,90 1055,01 2316,63 334,84 1091,69 1979,42
316,19 1308,21 2358,91 578,82 1372,56 2112,64 4154,45 5066,30 5148,24 7257,07
x x x x x x 12347,21 30766,76
31657,05 19843,01 34024,35 27840,10 15778,26
37178,98 32689,28 33711,81 43968,12 30540,53
116813,72 151713,78 x x x x
1,08 18108,84 37989,06 0,00 64132,64 x x
x x x x 602,01 1244,55 0,00 1089,23 5728,03 2792,24
10,51 21,73 19,02 48,75 0,17 2542,92 451,19 138,74 8108,56 2025,93
372,79 3040,36 10258,75 2207,65 533452,23 8,97 0,58 0,00
533452,23 8,97 2207,65 3,39 7,12 0,00 12,02 0,41 x
22,95
AFRO Mauritania Africa Western Africa 1234266,00
764637,00 30,83 17,61 51,56 235737,59 134652,58

394246,84						42,43	29,22	71,36	143,01
130010,05		238351,75		184180,90			436,27	579,29	126,44
14653,09		10036,27		59,38	4068,58	989,23	67,06	10584,50	
9047,04	5,88	3,69	7779,00	764637,00		143,01	143,01	18,70	436,27
57,06	184180,90		51,86	22,93	7415,92	10002,55		61241,10	
39339,04		11429,37		39339,04		78659,57		56757,50	
28847,84		56757,50		10287,18		800,24	10,29	2,28	623,03
8,01	12,46	87,54	10173,45		12,77	338,23	3,62	334,61	26,20
77,38	2027,54	21,94	2049,47	0,27	0,02	6159,24	0,81	0,06	52,09
245,67	553,92	54,33	195,80	376,03	49,35	297,26	628,35	65,28	245,77
473,09	857,89	1024,81	885,03	1247,56	1,03	1,22	885,03	1247,56	127,44
83,59	211,03	2622,16	7218,18	7377,79	3344,77	6344,37	5336,42	2386,04	8494,32
8303,97	4147,39	8198,23	6727,36	23767,55		28236,43		24519,62	
34374,05		3530,83	2303,06	5833,89	0,76	0,53	2049,47	6159,24	5833,89
4068,58	38646,32		2,61	7,83	7,42	5,17	76,98	77,38	159,97
211,03	143,01	528,22	147,86	14,65	30,28	27,07	27,99	0,17	665,74
136,17	50,30	1608,54	487,55	103,85	818,68	2130,70	489,60	57247,10	
7,49	0,58	3371,99	60619,09		7,93	3861,58	3,38	10,16	9,62
6,71	6,37	63,75	100,00						
AFRO	Mayotte	Africa	Eastern	Africa			44560,00		44560,00
			0,00	0,00	0,00				
0,00	0,00	0,00	0,00	0,00	0,00	0,00		25,42	25,42
									3,69
44560,00		0,00	0,00	0,00	25,42	57,06	0,00	x	x
	x	x	x	x	x	x	x		
x		x	x	x	x	x	x	x	x
x	x	x	4,51						
	x	x	x	x	x	x	x	x	x
x	x	x	x	x	31,95	45,04	x	x	x
x	x	x		x	x	x	x	x	x
x	x	x	x	x	x	x	x	x	x
x	x			x	0,00	0,00	0,00	0,00	x
x	x	x	x	x	4,51	9,32	0,00	0,00	34,75
20,92	12,98	26,83	0,00	60,19	0,17				
	0,00	0,00	0,00	0,00	0,00	0,58	0,00	0,00	x
0,00	x	x	x	x	x	x	x		
AFRO	Mozambique		Africa	Eastern	Africa			13188550,00	
12768231,00		70,22	8,59	21,19	8965851,81		1096791,04		
2705588,15						1613,85	238,00	489,71	2341,57
2128698,58		3902614,06		3015656,32			7285,09	9626,66	2437,88
239841,94		131490,09		1586,79	119541,83		33938,85		851,08
120300,10		97551,24		4,29	3,69	14021,00		12768231,00	
2341,57	2341,57	18,34	7285,09	57,06	3015656,32		53,95	26,73	
126320,91		194725,74		885101,45		769541,09		147633,45	
769541,09		1206148,10		1090587,73		468680,09		1090587,73	
9446,48	1324,49	9,45	2,51	971,94	6,93	14,63	85,37	1098,12	140,72
3767,41	43,23	3724,18	26,47	1292,10	34195,68		396,94	34592,62	
0,27	0,04	69242,46		0,54	0,08	774,03	2688,20	1891,95	1351,59
4798,67	2938,67	281,52	1089,98	2210,69	547,03	2172,06	4100,82	11222,69	
6194,43	9456,89	13330,63		0,84	2,15	9456,89	13330,63		1361,79
893,15	2254,94	38745,24		79873,73		30281,13		71267,28	
144865,05		48125,57		14211,47		31855,92		29104,70	
33326,65		68695,52		62997,40		360886,88		178790,26	
304104,14		384763,23		43791,00		25779,14		69570,13	
0,54	0,94	34592,62		69242,46		69570,13		119541,83	
797640,69		2,87	5,74	5,77	9,91	75,71	1292,10	2671,19	2254,94

2341,57 10042,90 3738,04 12,87 26,60 23,32 37,22 0,17 6557,77
1080,45 182,82 12662,40 2887,65 572,82 7699,16 15740,99 3891,06
1094478,80 8,57 0,58 40211,54 1134690,33 8,89 44102,60
3,05 6,10 6,13 10,54 3,89 70,30 100,00
AFRO Namibia Africa Southern Africa 722712,00
722712,00 21,85 16,40 61,75 157912,57 118524,77
446274,66 28,42 25,72 80,78 134,92
122654,41 224866,42 173760,41 412,35 547,27 171,75
10845,96 6171,76 30,48 1870,05 321,53 141,27 8975,91 5850,24 1,87
3,69 10700,00 722712,00 134,92 134,92 18,67 412,35 57,06
173760,41 50,80 22,13 6854,25 9123,56 18499,50 35132,27
7273,41 35132,27 34477,31 51110,09 23251,23 51110,09
4770,55 510,45 4,77 2,21 273,89 2,56 24,01 75,99 14805,34
50,43 1142,08 15,55 1126,53 22,34 73,14 1633,75 22,55 1656,30 0,23
0,02 3951,36 0,55 0,06 76,06 320,74 361,29 56,04 272,47 378,78
21,30 137,12 457,27 26,38 178,61 596,08 972,00 714,52 518,22 730,50
0,53 1,02 518,22 730,50 74,62 48,94 123,57 3606,73 9536,85 5289,77
3102,91 8169,95 5706,68 1031,51 3942,08 5777,30 1813,55 5520,69 8127,23 28081,92
16942,68 14971,88 17321,66 2155,95 1160,55 3316,50 0,46
0,26 1656,30 3951,36 3316,50 1870,05 40315,88 4,80 11,46 9,62
5,42 68,69 73,14 151,20 123,57 134,92 574,90 215,65 12,72 26,30
23,47 37,51 0,17 437,81 22,11 4,71 1025,83 207,26 49,49 461,49
1249,59 284,04 51394,13 7,11 0,58 1916,94 53311,07 7,38
2200,98 3,11 7,41 6,22 3,51 4,13 75,62 100,00
AFRO Niger Africa Western Africa 8435887,00
8396232,00 72,54 6,71 20,75 6090626,69 563387,17
1742218,14 1096,31 122,26 315,34 1533,91
1394463,00 2556515,51 1975489,25 4790,59 6324,49 1981,53
183044,74 89404,87 1393,98 99996,80 22822,67 587,55
83047,94 66582,20 4,04 3,69 13678,00 8396232,00
1533,91 1533,91 18,27 4790,59 57,06 1975489,25 55,36 28,02 84920,85
134253,46 567991,74 521143,80 91695,80 521143,80
787166,05 740318,11 310870,11 740318,11 9375,23 1282,34
9,38 2,61 925,29 6,76 13,98 86,02 1629,06 51,89 1569,82 15,52
1554,30 29,95 849,67 25450,79 254,13 25704,92
46390,19 0,55 0,08 345,99 1372,34 1701,41 422,13 1313,07 1406,04
223,24 1290,88 1822,41 426,38 1448,04 1717,22 4489,35 4568,41 6048,98 8526,77
x x x x x x 17468,15 40454,71
25119,34 24210,94 41015,76 21337,60 11014,81
36948,05 26398,59 25058,94 46077,80 25768,85
138635,21 136488,76 x x x x x
1,19 25704,92 46390,19 0,00 99996,80 x x
x x x x 849,67 1756,54 0,00 1533,91 8081,03 3940,92
10,51 21,74 18,98 48,77 0,17 2849,97 535,44 138,25 10443,84
2944,30 492,42 3431,50 13552,28 2819,31 743137,42 8,85 0,58
0,00 743137,42 8,85 2819,31 3,46 6,24 0,00 13,46 0,38
x 23,54
AFRO Nigeria Africa Western Africa 63226720,00
41587553,00 34,97 12,00 53,03 14543167,28 4990506,36
22053879,36 2617,77 1082,94 3991,75 7692,46
6993147,41 12820770,26 9906958,84 23728,35 31420,81
7275,32 948398,10 677402,09 3468,61 267781,62 85830,79
3806,71 680616,48 591571,30 6,64 3,69 432294,00
41587553,00 7692,46 7692,46 18,50 23728,35 57,06 9906958,84
52,46 23,39 403519,06 555045,22 3877791,21 2241091,93
781921,41 2241091,93 4836355,49 3199656,21 1740485,69

3199656,21 11629,33 50272,91 11,63 2,30 40308,84
9,32 12,28 87,72 10394,79 383,46 11217,37 122,75 11094,62
28,93 4208,52 121764,69 1347,19 123111,88 0,30 1,28
400530,86 0,96 4,16 3886,95 11897,84 20053,75 3244,72
10233,90 16182,02 2015,11 10426,28 21301,57 2580,13
10734,01 18658,48 41342,00 39075,55 45336,90
63907,87 1,10 1,64 45336,90 63907,87 6528,51 4281,83
10810,34 192865,42 357581,88 275850,16 188492,54
321733,44 234126,37 98285,39 301410,28 281248,27
170482,55 353212,59 267380,81 1230665,45 1106267,17
1349585,28 1809294,79 194340,28 121222,75 315563,03
0,76 0,64 123111,88 400530,86 315563,03 267781,62
2092668,82 2,55 8,28 6,52 5,54 77,11 4208,52 8700,35 10810,34
7692,46 29310,82 8709,49 14,36 29,68 26,24 29,71 0,17 32164,64
6294,76 1983,31 95490,13 26279,13 4183,96 39120,51
123163,91 26939,21 3226595,43 7,76 0,58 182395,43
3408990,86 8,20 209334,65 3,61 11,75 9,26 7,86 6,14
61,39 100,00
AFRO Réunion Africa Eastern Africa 247700,00
247700,00 13,00 12,00 75,00 32201,00 29724,00
185775,00 5,80 6,45 33,63 45,87
41701,42 76452,61 59077,01 141,33 187,20
3,69
247700,00 45,87 0,00 18,52 141,33 57,06 59077,01
x 0,00 x x x x x x x
x x x x x x x x x
x x x x 25,07 x x
x x x x x x x x
x x x x x x 177,61 250,37 x x
x x x x x x x x x
x x x x x x x x x x
x x x 0,00 0,00 0,00 0,00
x x x x x x 25,07 51,82 0,00 0,00
193,15 116,26 12,98 26,83 0,00 60,19 0,17
0,00 0,00 0,00 0,00 0,00 0,58 0,00 0,00
0,00 0,00 x x x x x x x
AFRO Rwanda Africa Eastern Africa 6354567,00
3297189,00 62,29 8,63 29,08 2053819,03 284547,41
958822,56 369,69 61,75 173,55 604,98
549982,82 1008301,83 779142,32 1881,26 2486,24 1183,15
114720,56 67491,20 615,60 46172,92 13162,28 567,54
68547,63 54328,92 3,82 3,69 10334,00 3297189,00
604,98 604,98 18,35 1881,26 57,06 779142,32 53,62 25,05 32441,02
47131,17 193020,82 187072,07 67264,08 187072,07
272593,01 266644,26 146836,27 266644,26 8267,44 854,36
8,27 2,41 604,96 5,85 16,65 83,35 3134,18 76,59 2024,99 23,70
201,29 2,63 333,66 876,92 103,25 980,17 0,03 0,00 36550,78
1,11 0,11 231,27 825,42 750,65 305,29 1069,55 979,82 100,65 572,68
1289,17 220,40 988,90 1753,59 3008,35 2896,88 4556,56 6423,03 1,51 2,22
4556,56 6423,03 656,14 430,34 1086,49 11778,04 24546,54 11126,36
17178,50 32103,08 14789,49 5117,93 16434,06 17209,20
13406,61 30660,90 25785,57 94244,77 79951,09
142746,49 177269,21 20555,49 11877,04 32432,53
0,98 1,40 980,17 36550,78 32432,53 46172,92
150507,86 0,36 13,41 11,90 16,94 57,40 333,66 689,79 1086,49
604,98 2089,54 461,10 15,97 33,01 28,95 22,07 0,17 3095,76 539,57

86,98	8377,46	2440,67	446,19	3664,33	10966,86		2428,78	269073,04	
8,16	0,58	18746,00		287819,04		8,73	21174,78		0,34
12,70	11,27	16,04	7,36	52,29	100,00				
AFRO	Saint Helena, Ascension, and Tristan Da Cunha						Africa	Western Africa	
	2486,00	2486,00	6,00	48,00	46,00	149,16	1193,28	1143,56	
		0,03	0,26	0,21	0,49	447,98	821,29	634,63	
1,42	1,91								
	3,69		2486,00	0,49	0,49	19,82	1,42	57,06	634,63
x	0,00		x	x	x	x	x	x	x
x	x		x	x	x	x	x	x	x
x	x	x	x	0,25	x	x			
		x	x	x	x	x	x	x	x
x	x	x	x	x	x	1,78	2,51	x	x
x	x	x	x		x	x	x	x	x
x	x	x	x	x	x	x	x	x	x
x	x	x				0,00	0,00	0,00	0,00
x	x	x	x	x	x	0,25	0,52	0,00	0,49
2,43	1,17	10,35	21,39	20,27	47,99	0,17			
		0,00	0,00	0,00	0,00	0,00	0,58	0,00	0,00
0,00	0,00	x	x	x	x	x	x	x	
AFRO	Sao Tome and Principe			Africa	Middle Africa				71873,00
62497,00		19,14	17,94	62,92	11961,93		11211,96		39323,11
				2,15	2,43	7,12	11,70	10639,66	
19506,04		15072,85			35,66	47,36	11,11	749,07	417,26
2,57	196,26	63,03	8,54	552,81	354,23	1,78	3,69	473,00	62497,00
11,70	11,70	18,73	35,66	57,06	15072,85		51,83	23,26	606,61
829,52	1766,71	3351,89	641,25	3351,89	3202,85	4788,03	2077,39	4788,03	5124,81
24,24	5,12	2,30	13,37	2,83	27,90	72,10	7568,36	1,10	30,99
0,30	30,69	27,90	6,32	176,45	1,72	178,18	0,29	0,00	202,71
0,32	0,00	5,84	21,73	34,95	6,55	16,80	22,36	3,75	17,82
40,68	6,55	17,69	33,39	70,03	70,51	51,54	72,65	0,74	1,03
51,54	72,65	7,42	4,87	12,29	286,44	645,36	461,03	387,28	547,50
322,67	177,13	515,57	515,80	394,16	601,87	471,72	2127,81	2017,89	1565,99
2078,99	225,50	139,29	364,79	0,58	0,31	178,18	202,71	364,79	196,26
3846,09	5,56	6,33	11,39	6,13	70,59	6,32	13,07	12,29	11,70
48,15	17,05	13,14	27,16	24,31	35,40	0,17	37,66	5,14	1,30
116,45	28,19	5,35	43,24	146,43	31,49	4819,52	7,71	0,58	210,85
5030,37	8,05	242,34	3,54	4,03	7,25	3,90	4,82	76,46	100,00
AFRO	Senegal	Africa	Western	Africa			4252308,00		
3890161,00		30,10	13,12	56,78	1170938,46		510389,12		
2208833,42						210,77	110,75	399,80	721,32
655747,46		1202203,68		928975,57			2219,58	2940,91	779,74
71664,44		42579,55		351,38	25064,12		6522,24	428,36	46600,32
36057,31		3,42	3,69	24911,00		3890161,00		721,32	721,32
18,54	2219,58	57,06	928975,57		52,77	24,61	38062,86		54630,00
200224,56		215231,95		49446,20		215231,95		292917,42	
307924,81		142139,06		307924,81		7529,70	1875,72	7,53	2,38
1282,16	5,15	17,57	82,43	6403,59	76,87	2035,54	20,91	2014,63	26,21
393,67	10317,43		107,09	10424,52		0,27	0,07	23822,69	
0,61	0,15	313,98	1231,49	2006,78	348,99	1071,60	1447,57	184,09	1084,44
2310,17	258,96	993,75	1816,87	4117,51	3896,92	3049,13	4298,12	0,74	1,10
3049,13	4298,12	439,07	287,97	727,05	15794,29		36571,36		28229,08
20027,31		33643,45		20955,65		8966,35	31155,90		30693,51
16140,79		32575,43		25558,04		122431,32		107588,98	
90663,74		118665,45		13055,58		7950,59	21006,16		0,54
0,64	10424,52		23822,69		21006,16		25064,12		

227607,32 3,56 8,13 7,17 8,56 72,58 393,67 813,84 727,05
721,32 3027,70 1098,87 13,00 26,88 23,82 36,29 0,17 2658,48 542,27
176,58 7208,94 2290,07 467,81 3259,60 9654,95 2143,82 310068,62 7,97
0,58 12141,56 322210,19 8,28 14285,38 3,24 7,39
6,52 7,78 4,43 70,64 100,00
AFRO Sierra Leone Africa Western Africa 2681078,00
2564249,00 54,49 6,76 38,76 1397259,28 173343,23
993902,91 251,51 37,62 179,90 469,02
426380,53 781697,63 604039,08 1463,07 1932,09 472,26
45136,56 26635,79 246,49 16578,12 3895,05 225,77 28558,44
22740,74 3,91 3,69 3865,00 2564249,00 469,02 469,02 18,29
1463,07 57,06 604039,08 51,46 25,77 24133,54 37700,98
154780,41 146706,46 30152,30 146706,46 216614,92
208540,97 91986,81 208540,97 8447,50 326,50 8,45 2,41
233,29 6,04 14,56 85,44 1507,26 28,03 820,17 8,06 812,11 28,97
259,49 7518,26 74,62 7592,88 0,30 0,01 16107,80 0,63 0,02
214,75 702,20 993,11 239,54 600,67 739,95 157,06 642,21 1210,92 252,87
574,61 1017,62 2334,85 2369,59 1922,47 2709,96 0,82 1,14 1922,47 2709,96
276,84 181,57 458,40 10685,00 20888,56 14177,64 13647,50
19137,65 11001,98 7761,76 18502,52 16614,78 14973,63
18836,40 14706,21 72751,91 70514,63 59902,65
80643,36 8625,98 5403,10 14029,09 0,55 0,65 7592,88 16107,80
14029,09 16578,12 154233,08 3,51 7,44 6,48 7,65
74,93 259,49 536,46 458,40 469,02 2010,14 745,17 12,91 26,69 23,33
37,07 0,17 1599,50 246,27 75,78 6258,51 1378,15 347,88 1871,03 7752,62
1597,53 210138,50 8,19 0,58 8108,81 218247,31 8,51 9706,34
3,48 7,38 6,43 7,60 4,45 70,67 100,00
AFRO South Africa Africa Southern Africa 23326430,00
16570981,00 5,28 22,31 72,41 874947,80 3696985,86
11999047,34 157,49 802,25 2171,83 3131,56
2846876,46 5219273,51 4033074,98 9454,80 12586,36
7108,52 382848,33 134057,68 3325,89 191742,45 26693,19
3782,63 191105,87 107364,49 1,28 3,69 301924,00
16570981,00 3131,56 3131,56 18,90 9454,80 57,06 4033074,98 49,63
22,14 155405,83 209314,28 293494,56 798493,17
132498,07 798493,17 658214,67 1163213,28 497218,18
1163213,28 3972,09 11992,70 3,97 2,20 5347,48 1,77 27,43
72,57 18220,04 2004,95 44441,66 510,96 43930,70 21,91
1676,93 36743,32 427,36 37170,69 0,22 0,68 46770,87
0,28 0,85 1701,13 6678,33 6975,08 1179,84 4926,90 6008,90 572,87 3714,92
10827,78 662,89 4167,27 12794,22 18814,19 16991,95
16726,28 23577,73 0,89 1,39 16726,28 23577,73
2408,58 1579,71 3988,29 83421,24 200100,11 98717,90 69311,16
153519,91 90284,48 28577,81 107618,96 130556,15
47504,01 136288,70 174707,77 569353,22 421744,13
506169,15 585204,73 72888,36 39208,72 112097,07
0,68 1,16 37170,69 46770,87 112097,07 191742,45
775432,20 5,65 7,11 17,03 29,13 41,09 1676,93 3466,74 3988,29
3131,56 12064,81 3789,58 13,90 28,73 25,96 31,41 0,17 13410,49
2160,01 386,31 38284,55 11308,14 2689,15 15699,28 50489,07
10987,27 1174200,55 7,09 0,58 64792,11 1238992,65
7,48 75779,37 3,00 3,77 9,05 15,48 6,12 62,59 100,00
AFRO South Sudan Africa Southern Africa 4671395,00
4110370,00 60,38 16,39 23,23 2481841,41 673689,64
954838,95 446,73 146,19 172,83 765,75
696134,51 1276246,59 986190,55 2345,23 3110,98 643,96

66472,59 39348,27 400,23 32996,06 11889,22 243,73
33476,53 27459,05 4,56 3,69 11998,00 4110370,00
765,75 765,75 18,63 2345,23 57,06 986190,55 52,74 24,69 40383,24
57902,00 0,00
35,06 979,89
10,14 969,75 27,66 415,96 11505,21 120,30 11625,51 0,28
0,03 18983,71 0,46 0,06 166,58 613,25 741,92 172,12 632,83
794,18 81,57 287,73 600,31 142,74 442,87 742,40 2096,81 1402,47 3349,64
4721,72 8342,49 18689,28
10844,56 9864,83 19911,36 12071,07 4129,54 8615,46 8067,65
9110,03 14795,89 11369,24
18983,71
415,96 859,91 0,00 765,75 3970,89 1929,27 10,48
21,66 19,28 48,59 0,17 1567,36 398,08 74,48 4144,51 1085,68 162,80
1990,26 5284,47 1207,61 1207,61 0,58 0,00 1207,61 0,03 1207,61
0,00 x x x x x x
AFRO Tanzania, United Republic of Africa Eastern Africa
27172946,00 26639212,00 65,09 6,47 28,44 17339463,09
1723557,02 7576191,89 3121,10 374,01
1371,29 4866,41 4424005,42 8110676,60 6267341,01 15199,37
20065,77 4209,22 417258,05 235755,93 2684,64 215505,17
70973,19 1524,58 201752,88 164782,75 4,46 3,69 62410,00
26639212,00 4866,41 4866,41 18,27 15199,37 57,06 6267341,01
53,84 24,25 261990,52 368576,47 1771465,66 1490442,09
293434,56 1490442,09 2402032,65 2121009,08 924001,54
2121009,08 9016,91 5627,45 9,02 2,37 4150,17 6,65 16,26 83,74
2342,79 333,90 8955,83 100,33 8855,49 26,52 2695,80 71496,24 810,03
72306,27 0,27 0,17 109874,16 0,41 0,26 1135,50 4350,69
5973,68 1212,64 3897,27 5435,92 469,46 1640,54 5082,30 959,40 3628,54 7396,32
14399,31 10857,47 19484,44 27465,69 1,35 2,53
19484,44 27465,69 2805,76 1840,20 4645,96 57583,42
129034,88 85861,99 69964,38 118460,34 83302,47
24464,38 48261,11 65965,58 59354,02 114125,00
109560,79 431431,17 304713,31 583791,49 770820,14
84065,98 51644,95 135710,92 0,51 0,81 72306,27
109874,16 135710,92 215505,17 1587612,55 3,01 4,57
5,65 8,97 77,79 2695,80 5573,07 4645,96 4866,41 20992,89 7857,61
12,84 26,55 23,18 37,43 0,17 8360,14 1733,29 372,42 30400,16
8290,33 1670,54 10217,58 39247,33 8211,17 2129220,25 7,99
0,58 78440,91 2207661,17 8,29 86652,09 3,28 4,98
6,15 9,76 3,93 71,91 100,00
AFRO Togo Africa Western Africa 2804487,00
2114627,00 32,38 19,21 48,41 684716,22 406219,85
1023690,93 123,25 88,15 185,29 396,69
360624,26 661144,48 510884,37 1206,53 1603,22 572,38
54507,95 30293,34 328,34 22104,55 4608,01 244,04 32403,41
25685,33 3,82 3,69 7575,00 2114627,00 396,69 396,69 18,76
1206,53 57,06 510884,37 53,29 27,53 21138,77 33213,52
132553,00 128282,22 31252,58 128282,22 186905,29
182634,51 85604,87 182634,51 8838,69 669,53 8,84 2,57
474,83 6,27 14,29 85,71 3582,19 35,27 1054,86 10,45 1044,41 29,61
213,99 6336,73 63,40 6400,13 0,30 0,02 16757,47 0,79 0,06
284,66 916,30 715,53 274,42 734,64 497,28 133,30 601,20 1058,23 200,62
572,17 869,25 2614,29 2149,78 2010,97 2834,70 0,77 1,32 2010,97 2834,70
289,58 189,92 479,50 13806,09 27427,16 10701,95 15022,63
23502,15 7768,52 6483,14 17283,72 14878,45 11842,50

18714,35 12963,30 85914,85 63604,29 66087,56
83868,71 9516,61 5619,20 15135,81 0,72 1,05 6400,13 16757,47
15135,81 22104,55 122236,56 3,42 8,97 8,10 11,83
67,69 213,99 442,39 479,50 396,69 1566,10 513,03 13,66 28,25 25,33
32,76 0,17 1789,09 283,97 52,09 4612,37 1365,25 256,48 2090,42 6063,12
1353,49 183988,00 8,70 0,58 8748,50 192736,50 9,11 10101,99
3,32 8,69 7,85 11,47 5,24 63,42 100,00
AFRO Uganda Africa Eastern Africa 16588429,00
16303226,00 72,13 6,51 21,36 11759516,91 1061340,01
3482369,07 2116,71 230,31 630,31 2977,33
2706666,03 4962221,05 3834443,54 9302,03 12279,36
2870,68 286301,68 159415,16 1776,64 140691,96 41798,66
1094,04 145609,73 117616,51 4,20 3,69 37372,00
16303226,00 2977,33 2977,33 18,26 9302,03 57,06 3834443,54 55,66
25,59 165727,69 238010,24 1070072,64 949430,59
187663,67 949430,59 1473810,58 1353168,53 591401,60
1353168,53 9039,99 3378,43 9,04 2,48 2452,94 6,56 16,00 84,00
2292,31 145,06 4009,66 46,56 3963,11 27,32 1649,83 45074,16 529,55
45603,71 0,28 0,10 86828,95 0,53 0,20 820,01 2137,92
2179,36 957,50 2437,74 2513,46 275,28 1102,36 3075,35 509,46 1720,84 3807,68
7917,44 5902,28 11894,78 16767,14 1,50 2,84 11894,78
16767,14 1712,85 1123,40 2836,25 41926,32 64590,55 32909,32
54342,36 75147,17 39052,22 14370,08 32131,76
40611,68 33174,14 55024,65 56672,15 259993,58
167128,57 390601,82 474777,10 56246,66 31810,07
88056,73 0,54 0,86 45603,71 86828,95 88056,73
140691,96 991987,18 3,09 5,89 5,97 9,55 75,49 1649,83
3410,73 2836,25 2977,33 12853,84 4815,95 12,84 26,53 23,16 37,47
0,17 14090,90 1806,60 329,29 38104,92 8598,03 1880,43 16007,26
47329,76 10513,95 1363682,47 8,36 0,58 50896,79
1414579,26 8,68 61410,74 3,22 6,14 6,22 9,95 4,34
70,13 100,00
AFRO Zambia Africa Eastern Africa 7390080,00
2786321,00 49,64 10,53 39,83 1383129,74 293399,60
1109791,65 248,96 63,67 200,87 513,50
466821,23 855838,93 661330,08 1589,77 2103,28 1075,47
105054,80 61602,94 562,54 39209,78 9514,09 512,93 65845,02
52088,85 3,79 3,69 19320,00 2786321,00 513,50 513,50
18,43 1589,77 57,06 661330,08 52,79 26,82 27107,17 42635,96
170129,16 166213,08 60773,61 166213,08 239872,28
235956,20 130516,73 235956,20 8608,92 1663,24 8,61 2,50
1179,65 6,11 14,92 85,08 6933,87 110,68 3127,30 33,78 3093,00 27,95
281,97 7879,66 86,06 7965,72 0,29 0,06 37752,05 1,35 0,26
527,27 1205,51 1179,92 697,46 2019,21 2018,74 249,32 622,06 1048,97 381,42
1199,97 2310,87 5515,67 3572,72 5299,08 7469,69 0,96 2,09 5299,08 7469,69
763,07 500,47 1263,54 26402,66 36708,72 17716,17 36874,45
60846,77 30672,30 12719,92 18438,12 14599,67
22721,20 37267,61 34701,35 176962,09 107580,52
170012,97 224924,92 24481,87 15069,97 39551,84
1,42 1,41 7965,72 37752,05 39551,84 39209,78
111476,82 3,32 15,74 16,49 16,35 48,11 281,97 582,91 1263,54
513,50 1422,65 44,27 19,82 40,97 36,09 3,11 0,17 4796,74 563,22
101,69 10112,97 1799,14 392,90 5393,86 12043,08 2894,53
238850,73 8,57 0,58 22860,96 261711,70 9,39 25755,49
3,04 14,43 15,11 14,98 9,84 42,60 100,00
AFRO Zimbabwe Africa Eastern Africa 7037039,00

2888597,00 66,19 6,57 27,24 1911962,35 189780,82
786853,82 344,15 41,18 142,42 527,76
479778,37 879593,67 679686,02 1648,13 2175,88 1204,53
105407,36 57320,98 580,78 36167,80 4602,16 623,75 69239,56
52718,82 3,19 3,69 16769,00 2888597,00 527,76 527,76
18,27 1648,13 57,06 679686,02 54,35 26,49 28683,66 43652,83
143481,20 165467,38 56900,80 165467,38 215817,69
237803,87 129237,29 237803,87 7471,37 1252,87 7,47 2,50
832,94 4,97 15,23 84,77 5805,24 112,15 2993,70 33,45 2960,25 26,40
292,32 7715,81 87,19 7803,00 0,27 0,05 37361,09 1,29 0,22
441,05 1912,70 1497,96 310,10 1000,03 939,99 230,22 1921,11 2775,98 240,80
980,52 1756,81 4476,53 4883,58 5045,93 7112,85 1,13 1,46 5045,93 7112,85
726,61 476,56 1203,17 21330,60 56709,05 22997,31 18205,64
30401,09 14615,80 10947,60 55080,17 38436,95
15402,53 31573,83 26120,44 139184,07 134523,26
156887,91 195930,76 22591,86 13127,36 35719,22
1,24 1,25 7803,00 37361,09 35719,22 36167,80
120752,76 3,62 17,31 16,55 16,76 45,76 292,32 604,31 1203,17
527,76 1577,02 152,64 18,54 38,32 33,47 9,68 0,17 1902,99 109,87
25,87 5446,14 1214,54 287,64 2021,48 6756,56 1457,16 239261,03 8,28
0,58 20645,71 259906,73 9,00 22102,86 3,00 14,37
13,74 13,92 8,50 46,46 100,00
AFRO TOTAL 422623000,00
357028042,00 187013725,04 0,00
339,00 0,00 0,00 33662,47 8579,99 23608,49 65850,94
59864493,81 109751571,98 84808032,89 203737,94
269588,88 80692,57 7219542,89 3847979,11 48156,01
3392108,08 835806,38 32536,56 3827434,81 3012172,73
3,69 3,69 1789489,00 357028042,00 65850,94 65805,05
18,44 203737,94 57,06 84808032,89 53,08 25,06 3509300,05
5108567,49 21436773,58 19918456,81 4242328,67 19918456,81
30054641,13 28438039,11 12860196,21 28536324,35 8418,01
150639,29 8,42 2,41 6,00
36130,00 1011285,48 0,28
5,07 1997108,90 0,56 10,01 27650,62 89732,48
109495,02 25941,74 80191,96 93156,54 13419,36
60579,03 129248,57 19585,46 67588,04 125570,87
291067,32 246111,71 303043,00 427176,00 1,04 1,74
303043,00 427176,00 43638,19 28620,79 72258,98
1372794,89 2578730,16 1598043,88 1463451,62 2485403,28
1390376,96 664550,96 1754453,58 1705298,17 1201320,43
2182290,84 1813496,59 8832073,73 6932417,51 9195460,80
12032594,54 1324146,36 806183,83 2031069,60 0,57 0,95
999659,97 1997108,90 2031069,60 3359112,02 20051088,62
36130,00 74692,26 67914,81
65805,05 276320,44 99693,12 13,08 27,03 23,81 36,08
0,17 239101,83 38202,99 8484,55 650650,21 153109,01
29665,48 280133,00 813647,71 181567,60 28717891,95
8,04 0,58 1173958,23 29891850,18 8,37 1453811,07 3,34
6,68 6,79 11,24 4,86 67,08 100,00

AMRO	Anguilla		America	Caribbean				6049,00	6049,00
4,00	21,00	75,00	241,96	1270,29	4536,75				
0,02	0,14	0,26	0,42	383,56	703,20	543,38	543,00	4,00	4,42
									2,43
6049,00	0,42	0,42	6,98	4,00	66,13	543,38	x	x	x
x	x	x		x	x	x		x	x
x	x	x	x	x	x	x	x	x	x
x	1,44	x	x						
x	x	x	x	x	x	x	x	x	x
x	x	x	x	7,88	5,43	x	x	x	x
x	x		x	x	x	x	x	x	x
x	x	x	x	x	x	x	x	x	x
x				0,00	0,00	0,00	0,00	x	x
x	x	x	x	1,44	1,20		0,42	5,62	2,56
25,67	21,33	7,51	45,50	0,17					
0,00	0,00	0,00		0,00	0,58	0,00	0,00	0,00	0,00
x	x	x	x	x	x	x			
AMRO	Argentina		America	South America				20877041,00	
12041135,00		0,06	21,84	78,10	7224,68	2629783,88		9404126,44	
			0,63	294,54	536,04	831,20	755635,95		
1385332,58		1070484,27		1070484,00		7568,00	8400,00	7211,45	
414585,13		200671,60		2417,62	200658,18		82342,49		4793,83
213926,96		118329,12		1,24	2,43	383067,00		12041135,00	
831,20	831,20	6,90	7568,00	62,85	1070484,27		48,94	19,94	40678,05
150919,86		215115,74		394919,44		146639,24		394919,44	
406713,65		586517,36		338237,15		586517,36		3377,70	12938,86
3,38	1,59	6843,52	1,79	16,96	83,04	31813,20		2764,95	61398,71
752,45	60646,26		21,93	2871,86	62991,21		781,54	63772,76	
0,53	2,03	71709,73		0,60	2,28	1325,07	7874,51	14786,42	
839,69	3831,68	7946,09	397,28	2866,07	18121,83		639,93	2364,27	10414,31
21448,46		15779,60		27182,40		18755,87		1,27	1,19
27182,40		18755,87		3914,27	1256,64	5170,91	62869,76		
226412,82		194850,13		46440,18		117506,04		112345,72	
19293,69		80356,70		195352,26		39849,04		79317,75	
133828,16		555627,41		328544,00		704166,45		390512,28	
101399,97		26164,32		127564,29		1,06	1,67	63772,76	
71709,73		127564,29		200658,18		122812,40		15,68	17,63
31,36	49,34	-14,01	2871,86	2386,89	5170,91	831,20	5615,18	-474,77	51,14
42,51	14,80	-8,46	0,17	4758,26	499,48	132,51	14979,08		2757,41
629,10	5301,91	17946,19		3859,18	590376,54		4,90	0,58	73732,16
664108,70		5,52	77591,34		9,60	10,80	19,21	30,21	11,68
18,49	100,00								
AMRO	Aruba	America	Caribbean				51610,00		51610,00
0,60	14,50	84,90	309,66	7483,45	43816,89				
0,03	0,84	2,50	3,36	3056,95	5604,42	4330,69	4331,00	32,00	36,00
									2,43

3202,00	51610,00		3,36	3,36	6,52	32,00	62,00	4330,69	x
x			x	x	x		x	x	x
x	x		x	x	x	x	x	x	x
x	x	x	x	12,31	x	x			
		x	x	x	x	x	x	x	x
x	x	x	x	x	x	67,20	46,37	x	x
x	x	x	x		x	x	x	x	x
x	x	x	x	x	x	x	x	x	x
x	x	x				0,00	0,00	0,00	0,00
x	x	x	x	x	x	12,31	10,23		3,36
45,59	19,69	27,00	22,44	7,38	43,19	0,17			
		0,00	0,00	0,00		0,00	0,58	0,00	0,00
0,00	0,00	x	x	x	x	x	x	x	
AMRO	Belize	America	Central	America			178991,00		
149157,00		16,80	15,59	67,61	25058,38		23253,58		
100845,05						2,18	2,60	5,75	10,53
9575,13	17554,41		13564,77		13565,00		94,00	104,00	30,03
2302,95	1185,65	13,69	942,99	229,18	16,34	1359,96	956,47	2,37	2,43
1764,00	149157,00		10,53	10,53	7,06	94,00	63,02	13564,77	
52,16	24,69	549,38	2321,17	5678,74	5814,90	1132,86	5814,90	8549,29	8685,45
4003,41	8685,45	5731,74	101,11	5,73	1,92	67,16	3,81	8,49	91,51
11826,46		8,22	221,05	2,52	218,53	26,59	35,57	945,76	10,91
956,66	0,64	0,01	603,90	0,40	0,01	12,65	48,92	65,95	8,70
27,80	40,87	5,04	23,88	53,64	5,58	17,06	37,75	133,67	82,02
233,05	160,80	1,74	1,96	233,05	160,80	33,56	10,77	44,33	624,60
1486,04	883,70	475,82	857,18	555,45	248,41	721,21	637,39	344,56	560,48
472,58	3923,36	2244,65	6840,06	4401,01	984,97	294,87	1279,84	0,86	0,63
956,66	603,90	1279,84	942,99	4902,07	11,19	7,06	14,97	11,03	55,75
35,57	29,57	44,33	10,53	89,77	14,09	39,63	32,94	11,73	15,70
0,17	48,50	4,09	0,80	172,70	25,38	3,77	52,86	199,33	41,86
8727,32	5,85	0,58	739,75	9467,06	6,35	781,61	10,11	6,38	13,52
9,96	8,26	51,78	100,00						
AMRO	Bermuda	America	North	America			36387,00		33480,00
2,00	15,00	83,00	669,60	5022,00	27788,40				
0,06	0,56	1,58	2,20	2004,23	3674,43	2839,33	2839,00	21,00	23,20
9,80	467,51	272,84	0,74	89,92	57,65	9,06	377,58	215,19	1,33
2,43	7484,00	33480,00		2,20	2,20	6,59	21,00	62,72	2839,33
43,33	17,93	95,53	376,62	669,73	1085,55	385,87	1085,55	1141,88	1557,70
858,02	1557,70	3410,63	255,25	3,41	1,41	149,71	2,00	23,31	76,69
223536,44		7,44	138,91	2,17	136,74	18,38	7,99	146,76	2,33
149,09	0,45	0,03	112,90	0,34	0,03	1,73	16,44	47,21	0,60
5,50	17,98	0,33	3,77	40,20	0,26	2,15	18,77	45,99	26,16
47,38	32,69	x	x	x	x	x	x		80,77
483,93	598,14	34,08	172,03	237,92	16,38	115,68	408,20	22,24	83,81
214,71	1049,50	445,75	x	x	x	x			0,27
149,09	112,90	0,00	89,92	x	x	x	x	x	x
7,99	6,64		2,20	29,84	13,01	26,76	22,24	7,39	43,61
0,17	3,95	1,32	0,40	27,06	16,88	5,65	5,40	45,82	8,50
1566,20	4,68	0,58	0,00	1566,20	4,68	8,50	9,52	7,21	0,00
5,74	0,54	x	23,01						
AMRO	Bolivia	America	South	America			5758760,00		
5443620,00		30,54	19,41	50,05	1662481,55		1056606,64		
2724531,81						144,64	118,34	155,30	418,27
380249,23		697123,59		538686,41		538686,00		3422,00	3840,27
916,34	69700,45		42941,63		287,62	19246,44		4776,87	628,72
50454,01		38164,76		3,11	2,43	36689,00		5443620,00	

418,27 418,27 7,68 3422,00 62,86 538686,41 50,31 19,55 21042,75
66887,72 214669,39 169428,23 45111,65 169428,23
302599,86 257358,71 133042,13 257358,71 5558,80 2039,47
5,56 1,62 1446,83 3,94 9,25 90,75 6739,82 277,74 6268,51 72,37
6196,14 22,31 1298,33 28964,52 338,30 29302,83 0,54 0,20
27187,58 0,50 0,18 254,48 1311,75 2500,81 222,67 777,98 1402,49
159,35 846,64 2641,35 281,37 829,11 1743,46 3867,98 3578,08 7498,04 5173,65
x x x x x x 12812,57 38051,21
34448,89 12777,39 23470,98 19842,04 7933,58 24114,29
34286,29 15972,68 25771,36 24582,40 105209,12
93414,80 x x x x 0,35 29302,83
27187,58 0,00 19246,44 x x x x x
x 1298,33 1079,08 418,27 4919,35 2123,67 26,39 21,94 8,50
43,17 0,17 1164,78 220,35 43,28 7300,40 2253,01 427,60 1399,55 9695,94
1841,85 259200,56 4,76 0,58 0,00 259200,56 4,76 1841,85
11,31 10,49 0,00 7,43 0,71 x 29,93
AMRO Brazil America South America 107461083,00
92603010,00 9,08 19,99 70,94 8408353,31 18511341,70
65692575,29 731,53 2073,27 3744,48 6549,27
5953885,27 10915456,33 8434670,80 9675063,57 58205,00
64755,00 23741,34 1608922,87 874647,72 8392,58
566689,94 145478,05 15348,75 1042232,94 729169,68
2,33 2,43 1444733,00 92603010,00 6549,27 6549,27 7,07 58205,00
62,85 8434670,80 50,19 20,40 328698,87 1187187,39
2878657,31 2997400,54 842695,60 2997400,54 4394543,57
4513286,79 2358581,86 4513286,79 4745,57 68560,86 4,75
1,64 44910,97 3,11 10,06 89,94 15601,36 6469,42
148718,28 1852,63 146865,66 22,70 22086,20 501390,18
6324,76 507714,95 0,55 7,92 479368,95 0,52 7,48 9018,19
39360,77 47659,72 4766,61 21756,57 39451,29 3813,42
20768,99 50625,27 4544,19 15520,84 44780,08
103939,14 76449,21 139916,86 96542,72 1,35 1,26
139916,86 96542,72 20148,03 6468,36 26616,39
437992,99 1188364,71 679374,39 255374,11 668861,78
556961,56 182088,53 607233,29 625969,55 260801,15
510780,68 588612,14 2962705,57 1965764,21 3988223,07
2482435,50 574304,12 166323,18 740627,30 0,80 0,61
507714,95 479368,95 740627,30 566689,94 2218885,66
11,55 10,91 16,85 12,90 47,79 22086,20 18356,49 26616,39
6549,27 56494,37 9502,41 39,09 32,49 11,59 16,82 0,17 21555,62
3383,99 691,58 101371,37 27349,79 4438,07 25170,14
130200,52 25791,53 4539078,32 4,90 0,58 428082,58
4967160,90 5,36 453874,11 10,22 9,65 14,91 11,41 9,14
44,67 100,00
AMRO Cayman Islands America Caribbean 39000,00
39000,00 1,90 19,10 79,00 741,00 7449,00 30810,00
0,06 0,83 1,76 2,65 2413,57 4424,88 3419,22 4425,00
25,00 27,00
2,43 5936,00 39000,00 2,65 2,65 6,81 25,00 64,10
3419,22 x x x x x x
x x x x x x x x
x x x x x x x 9,30 x x
x x x x x
x x x x x x x x x 50,78
35,04 x x x x x x x x
x x x x x x x x x x

x x x x x x 0,00
0,00 0,00 0,00 x x x x x x 9,30
7,73 2,65 35,39 15,70 26,29 21,85 7,50 44,36 0,17
0,00 0,00 0,00 0,00
0,58 0,00 0,00 0,00 0,00 x x x x x
x x
AMRO Colombia America South America 26413532,00
22157441,00 15,77 20,12 64,11 3494228,45 4458077,13
14205135,43 304,00 499,30 809,69 1613,00
1466359,30 2688325,39 2077342,34 2077342,00 13927,00
15540,00 3570,74 257427,46 162531,13 945,02 69950,09
22408,86 2625,72 187477,37 140122,26 2,96 2,43
271347,00 22157441,00 1613,00 1613,00 7,28 13927,00 62,85
2077342,34 50,31 18,04 81145,14 251175,01 781467,20
648393,95 178370,55 648393,95 1113787,35 980714,10
510690,70 980714,10 5026,70 13639,79 5,03 1,50 9570,09
3,53 10,72 89,28 12246,31 774,98 18959,65 242,88 18716,77
24,15 5284,64 127630,90 1656,21 129287,11 0,58 1,58 89658,00
0,40 1,10 1061,99 5999,95 14920,27 485,99 1912,65 5320,95 508,98
3557,20 15660,74 521,75 1976,16 6675,08 16207,65 14009,36
34391,04 23729,84 2,12 1,69 34391,04 23729,84
4952,31 1589,90 6542,21 52998,75 175917,29 185084,15 28885,52
60290,10 68013,16 25255,39 103888,49 174537,94
33470,85 67348,97 85491,91 402457,43 316640,33
853975,18 536343,25 122972,43 35935,00 158907,42
0,72 0,32 129287,11 89658,00 158907,42 69950,09
532911,48 11,61 8,05 14,27 6,28 59,79 5284,64 4392,22 6542,21
1613,00 13390,01 2100,15 39,47 32,80 12,05 15,68 0,17 4247,21
896,02 281,91 12808,19 4821,29 1094,55 5237,20 17994,33 3856,43
984570,54 4,44 0,58 91848,49 1076419,03 4,86 95704,92
12,01 8,33 14,76 6,50 8,89 49,51 100,00
AMRO Costa Rica America Central America 2568229,00
2175098,00 11,97 18,80 69,22 260359,23 408918,42
1505602,84 22,65 45,80 85,82 154,27
140244,98 257115,80 198680,39 198680,00 1367,00 1521,27
315,78 24369,89 14930,95 123,07 8623,27 2535,56 192,71 15746,63
12395,39 3,70 2,43 61521,00 2175098,00 154,27 154,27
7,09 1367,00 62,85 198680,39 49,47 17,39 7631,11 23772,12
91105,63 60924,81 15573,78 60924,81 122508,86
92328,04 46977,01 92328,04 5632,34 3465,07 5,63 1,44
2576,85 4,19 8,82 91,18 28284,24 51,92 1225,26 17,17 1208,09
23,27 518,77 12070,89 171,56 12242,45 0,56 0,35 7164,45
0,33 0,20 122,77 735,04 1319,44 43,05 169,25 500,52 38,53 290,37
1202,02 34,24 117,69 603,80 1676,77 1082,77 3343,89 2307,29 1,99 2,13
3343,89 2307,29 481,52 154,59 636,11 5967,03 21710,42 16873,06
2600,27 5464,26 6362,75 1920,89 8538,15 13341,65 2516,86 4391,38 7255,66
43487,24 24233,05 86724,22 51638,69 12488,29
3459,79 15948,08 0,73 0,40 12242,45 7164,45 15948,08
8623,27 48349,79 9,99 5,85 13,02 7,04 64,10 518,77 431,16
636,11 154,27 1316,33 212,12 39,41 32,76 11,72 16,11 0,17 716,55
233,75 58,04 2958,86 1097,82 217,76 969,65 4129,26 846,42 93174,45
4,28 0,58 9217,99 102392,45 4,71 10064,41 11,96 7,00
15,58 8,42 9,83 47,22 100,00
AMRO Cuba America Caribbean 5092603,00
5006980,00 17,40 17,11 65,49 871214,52 856694,28
3279071,20 75,80 95,95 186,91 358,65

326047,71 597754,13 461900,92 461901,00 3147,00 3505,65
2294,87 156074,00 78772,86 1179,51 99983,84 46393,82
1115,36 56090,16 32379,03 1,37 2,43 103131,00
5006980,00 358,65 358,65 7,16 3147,00 62,85 461900,92 45,43
21,26 16295,09 66901,28 105464,86 176621,24 46485,98
176621,24 188661,24 259817,61 129682,36 259817,61
3767,96 3885,94 3,77 1,66 2172,31 2,11 16,11 83,89 20597,45
591,38 14299,49 186,79 14112,70 23,86 1194,19 28498,05
377,19 28875,24 0,58 0,59 15078,85 0,30 0,31 410,81
3242,99 8118,12 190,74 1360,03 3767,26 136,68 1712,63 8650,76 122,25 915,72
4477,81 9166,36 7263,46 6630,69 4575,18 0,72 0,63 6630,69 4575,18 954,82
306,54 1261,36 19224,91 95978,71 102678,31 10126,49
42043,82 49457,05 6415,57 49199,56 97066,24 7526,35
29944,63 54419,80 218085,71 143581,46 157757,12
90440,46 22717,02 6059,51 28776,54 0,57 2,00 28875,24
15078,85 28776,54 99983,84 87103,14 15,31 7,99
15,25 53,00 8,45 1194,19 992,52 1261,36 358,65 3236,82 691,46 36,89
30,66 11,08 21,36 0,17 1115,30 313,54 96,82 4886,32 2911,52 767,70
1461,11 8053,74 1579,47 261397,08 5,22 0,58 16632,84
278029,92 5,55 18212,30 10,39 5,42 10,35 35,96 6,55
31,33 100,00
AMRO Dominica America Caribbean 5199072,00
5199072,00 40,00 32,00 28,00 2079628,80 1663703,04
1455740,16 180,93 186,33 82,98 450,24
409308,76 750399,39 579854,08 579854,00 3268,00 3718,24
6,19 365,94 218,06 0,79 59,30 21,53 5,40 306,64 196,52 1,78
2,43 470,00 5199072,00 450,24 450,24 8,66 3268,00 62,86
579854,08 47,87 20,38 21550,96 66608,71 131162,24
174090,95 12483,90 174090,95 219321,90 262250,61
100643,57 262250,61 4218,48 19,83 4,22 1,70 11,86 2,52
15,43 84,57 90,40 3,16 68,04 0,81 67,24 21,28 1240,00 26385,31
317,85 26703,16 0,51 0,00 119,40 0,00 0,00 1,41 12,91
23,70 1,11 9,37 19,79 0,79 6,09 36,27 1,03 5,25 30,72
39,29 35,50 6769,31 4670,83 172,28 131,57 6769,31 4670,83 974,78 312,95
1287,73 69,01 383,74 322,17 60,86 282,75 272,87 38,23 173,34 414,13
60,67 164,45 371,42 994,70 698,53 171371,61 91907,79 24677,51
6157,82 30835,33 0,59 0,00 26703,16 119,40 30835,33
59,30 204533,41 12,18 0,05 14,06 0,03 73,68 1240,00 1030,60
1287,73 450,24 3461,11 740,27 35,83 29,78 13,01 21,39 0,17 6,05
1,03 0,25 27,93 8,99 2,17 7,16 37,65 7,44 262258,05
5,04 0,58 17822,82 280080,87 5,39 17830,26 9,53
0,04 11,01 0,02 6,37 73,03 100,00
AMRO Dominican Republic America Caribbean
5199103,00 4663190,00 8,78 18,82 72,40 409428,08
877612,36 3376149,56 35,62 98,29
192,44 326,35 296684,87 543922,25 420303,56 420304,00
2931,00 3257,35 867,21 72378,26 38108,91 510,56 38139,65
12414,84 356,64 34238,61 25694,07 3,01 2,43 78845,00
4663190,00 326,35 326,35 7,00 2931,00 62,85 420303,56 50,39
23,96 16443,43 70221,18 219096,14 178514,48 33629,70
178514,48 305760,75 265179,09 120294,30 265179,09
6556,90 5169,79 6,56 1,86 3704,47 4,70 7,97 92,03 16907,95
155,31 4195,67 44,86 4150,81 26,73 1112,19 29724,35 321,25 30045,60
0,64 0,51 16441,54 0,35 0,28 977,13 3250,83 4596,67 416,05
1600,79 2299,99 468,80 1562,87 4449,85 334,37 992,27 2474,66 8543,69 5666,48
6769,35 4670,86 0,79 0,82 6769,35 4670,86 974,79 312,95 1287,73 47363,42

96811,32 61368,21 21443,21 48591,81 32397,37
23395,99 45864,18 53246,01 18372,00 31609,91
31939,04 245464,96 147637,10 194487,33 121696,74
28006,18 8153,68 36159,86 0,78 0,82 30045,60 16441,54
36159,86 38139,65 144392,44 9,83 5,38 11,83 12,47
60,50 1112,19 924,37 1287,73 326,35 2893,99 531,08 38,43 31,94 11,28
18,35 0,17 1180,36 158,48 37,64 5661,46 1270,41 286,77 1351,39 7027,46
1390,89 266569,98 5,72 0,58 20900,40 287470,38 6,16
22291,29 10,45 5,72 12,58 13,27 7,75 50,23 100,00
AMRO Ecuador America South America 8519410,00
7853175,00 29,74 17,21 53,04 2335534,25 1351531,42
4165324,02 203,19 151,37 237,42 591,99
538169,52 986644,11 762406,81 762407,00 4936,00 5527,99
1385,96 106981,11 55955,41 818,72 50961,65 9917,15 567,24
56019,47 46038,26 4,61 2,43 98808,00 7853175,00
591,99 591,99 7,54 4936,00 62,85 762406,81 50,13 17,60 29677,88
86853,85 407783,90 218153,13 53209,02 218153,13
524315,63 334684,85 169740,74 334684,85 6676,48 6596,90
6,68 1,48 5130,70 5,19 7,03 92,97 12581,92 218,21 5057,49
61,19 4996,31 22,90 1873,01 42886,03 525,23 43411,25 0,55
0,55 29643,55 0,38 0,37 614,97 1809,63 3718,97 370,16 823,33
1793,60 229,27 899,86 3411,28 288,72 728,52 2063,89 5455,61 3971,42 11092,47
7653,81 2,03 1,93 11092,47 7653,81 1597,32 512,81 2110,12 31634,03
54274,41 47532,73 21479,66 26220,59 24218,12
11627,85 26721,69 39198,39 17671,41 24547,56
26903,71 157525,65 102602,54 320284,81 197737,75
46121,01 13248,43 59369,44 0,76 0,65 43411,25
29643,55 59369,44 50961,65 151298,96 8,28 5,65
11,32 9,72 65,02 1873,01 1556,72 2110,12 591,99 4974,58 952,86 37,65
31,29 11,90 19,15 0,17 2224,35 400,95 89,64 9418,52 2218,18 450,01
2655,18 11786,71 2397,35 337082,21 4,29 0,58 34315,54
371397,74 4,73 36712,89 11,69 7,98 15,99 13,72 9,89
40,74 100,00
AMRO El Salvador America Central America 2790598,00
2908120,00 16,29 22,51 61,20 473732,75 654617,81
1779769,44 41,21 73,32 101,45 215,98
196344,37 359964,67 278154,52 278155,00 1828,00 2043,98
336,48 29811,82 18426,37 154,20 10240,45 2290,70 182,28
19571,37 16135,67 4,70 2,43 24639,00 2908120,00
215,98 215,98 7,43 1828,00 62,86 278154,52 51,56 18,85 11134,86
34454,39 165022,45 86903,89 19344,14 86903,89
210611,70 132493,14 64933,39 132493,14 7242,19 1784,40
7,24 1,57 1398,15 5,67 6,81 93,19 8472,48 48,49 1375,10 15,90
1359,20 28,03 693,60 19441,93 227,43 19669,36 0,68 0,17
10510,79 0,36 0,09 230,37 768,86 2206,43 83,37 212,78 684,75
109,32 561,94 2605,05 73,28 227,56 903,18 2259,11 2141,51 3633,42 2507,07
1,61 1,17 3633,42 2507,07 523,21 167,97 691,19 11570,80 22724,08
26825,80 4816,75 6603,48 8759,43 5426,03 16396,41 28546,23
4675,93 7655,88 11282,88 57576,85 47430,62 92603,47
55527,01 13334,90 3720,31 17055,21 0,59 0,35 19669,36
10510,79 17055,21 10240,45 75017,33 9,34 4,99
8,10 4,86 72,71 693,60 576,47 691,19 215,98 1929,26 443,22 35,95
29,88 11,19 22,97 0,17 594,80 111,99 38,23 2932,32 689,50 159,08
719,53 3674,85 729,47 133222,61 4,58 0,58 9857,91 143080,52
4,92 10587,38 13,75 7,35 11,92 7,16 7,40 52,43 100,00
AMRO Falkland Islands (Malvinas) America South America

1850,00	95,00	2,00	3,00	1757,50	37,00	55,50			
0,15	0,00	0,00	0,16	145,65	267,02	206,33	206,00	0,92	1,08
									2,43
1850,00	0,16	0,16	8,66	0,92	49,55	206,33	x	x	
x	x	x		x	x	x		x	x
x	x	x	x	x	x	x	x	x	x
x	0,44	x	x						
x	x	x	x	x	x	x	x	x	x
x	x	x	x	0,00	0,00	x	x	x	x
x	x		x	x	x	x	x	x	x
x	x	x	x	x	x	x	x	x	x
x				0,00	0,00	0,00	0,00	x	x
x	x	x	x	0,44	0,37		0,16	1,44	0,48
x	25,40	11,10	32,93	0,17					
0,00	0,00	0,00		0,00	0,58	0,00	0,00	0,00	0,00
x	x	x	x	x	x	x			
AMRO	French Guiana		America	South America				4433672,00	
102295,70		18,00	21,00	61,00	18413,23		21482,10		62400,38
				1,60	2,41	3,56	7,56	6877,06	12607,95
9742,50	9743,00	64,00	71,56						
			2,43		102295,70		7,56	7,56	7,40
64,00	62,56	9742,50	x	x			x	x	x
x	x	x		x	x		x	x	x
x	x	x	x	x	x	x	x	24,40	x
x							x	x	x
x	x	x	x	x	x	x	x	x	x
x	5772,75	3983,20	x	x	x	x	x	x	
x	x	x	x	x	x	x	x	x	x
x	x	x	x	x	x	x	x		
0,00	0,00	0,00	0,00	x	x	x	x	x	x
24,40	20,28		7,56	91,84	39,60	26,56	22,08	8,24	43,12
0,17							0,00	0,00	0,00
0,00	0,58	0,00	0,00	0,00	0,00	x	x	x	x
x	x	x							
AMRO	Grenada	America	Caribbean				59900,00		55270,00
11,00	20,00	69,00	6079,70	11054,00		38136,30			
	0,53	1,24	2,17	3,94	3582,50	6567,92	5075,21	5075,00	35,00
38,94	6,10	548,61	358,02	1,75	143,93	57,90	4,35	404,68	300,12
2,87	2,43	1089,00	55270,00		3,94	3,94	7,13	35,00	63,33
5075,21	49,16	24,05	193,72	841,59	2545,99	2174,75	430,50	2174,75	3581,31
3210,06	1465,82	3210,06	6479,66	70,56	6,48	1,87	50,16	4,61	9,05
90,95	19703,27		2,35	62,03	0,71	61,32	26,09	13,18	343,97
3,98	347,95	0,63	0,01	182,29	0,33	0,00	3,36	26,44	27,47
2,64	17,13	20,76	1,23	11,62	35,09	2,10	11,88	36,41	65,63
50,66	77,99	53,81	1,19	1,06	77,99	53,81	11,23	3,61	14,84
159,44	776,37	432,54	138,62	514,58	325,43	59,83	337,33	442,10	119,68
368,60	474,30	1841,67	1190,90	2188,56	1265,14	315,15	84,76	399,92	0,72
0,26	347,95	182,29	399,92	143,93	2135,97	9,72	5,09	11,17	4,02
70,01	13,18	10,96	14,84	3,94	35,06	6,98	37,60	31,25	11,24
19,91	0,17	13,69	1,67	0,28	45,65	13,13	2,58	15,46	59,64
12,47	3222,53	5,83	0,58	231,15	3453,68	6,25	243,62	10,07	5,28
11,58	4,17	7,05	61,85	100,00					
AMRO	Guadeloupe		America	Caribbean				215923,40	
215923,40		7,00	5,00	88,00	15114,64		10796,17		
190012,59						1,31	1,21	10,83	13,35
12140,78		22258,10		17199,44		17199,00		136,00	149,35

2,43
215923,40 13,35 13,35 6,19 136,00 62,99 17199,44 x
x x x x x x x
x x x x x x x x x
x x x x 51,50 x x
x x x x x x x x
x x x x x x 281,14 193,98 x x
x x x x x x x x x
x x x x x x x x x x
x x x 0,00 0,00 0,00 0,00
x x x x x x 51,50 42,80 13,35
192,16 84,50 26,80 22,27 6,95 43,98 0,17
0,00 0,00 0,00 0,00 0,58 0,00 0,00
0,00 0,00 x x x x x x x
AMRO Guatemala America Central America 6879134,00
7104989,00 31,30 18,73 49,98 2223861,56 1330764,44
3551073,50 193,48 149,05 202,41 544,93
495393,42 908221,27 701807,35 701807,00 4466,00 5010,93
1608,03 135404,40 60681,73 1240,08 83797,62 16566,80
367,95 51606,77 44114,93 5,89 2,43 77605,00
7104989,00 544,93 544,93 7,67 4466,00 62,86 701807,35 54,22
20,36 29543,54 90932,34 542725,66 228169,71 51394,95
228169,71 663201,54 348645,59 171870,82 348645,59
9334,31 7243,89 9,33 1,70 5927,98 7,64 5,55 94,45 10922,61
89,19 2602,07 29,01 2573,06 28,85 1694,57 48886,95 551,18 49438,13
0,70 0,54 30946,46 0,44 0,34 584,93 1106,24 3047,53 308,07
486,69 1418,19 331,39 981,70 3356,68 315,33 608,67 1569,74 3974,49 3879,23
8956,79 6180,19 2,25 1,59 8956,79 6180,19 1289,78 414,07 1703,85 30473,79
33067,06 38220,31 17916,21 15084,32 18736,09
17331,35 28747,23 39097,42 19360,43 19780,12
20680,40 115526,85 105145,08 260347,64 167511,97
37490,06 11223,30 48713,36 0,69 1,18 49438,13
30946,46 48713,36 83797,62 135750,01 7,45 4,67
7,35 12,64 67,90 1694,57 1408,41 1703,85 544,93 4715,49 1067,58 35,94
29,87 11,56 22,64 0,17 2010,62 262,94 73,77 7602,22 1432,04 286,38
2298,15 9129,72 1897,03 350542,61 4,93 0,58 28156,32
378698,94 5,33 30053,35 13,05 8,17 12,86 22,13 7,94
35,85 100,00
AMRO Guyana America South America 309040,00
251594,00 15,44 24,97 59,60 38846,11 62823,02
149950,02 3,38 7,04 8,55 18,96
17239,04 31604,90 24421,97 24422,00 158,00 176,96
42,38 3632,35 2012,64 19,08 1317,44 326,61 23,30 2314,91 1686,04 2,68
2,43 5471,00 251594,00 18,96 18,96 7,54 158,00 62,80 24421,97
51,94 26,99 984,98 4264,37 11757,69 10683,54 2010,71 10683,54
17007,04 15932,89 7260,06 15932,89 6759,72 369,82 6,76
2,09 255,68 4,67 7,70 92,30 21745,35 10,08 297,92 3,48
294,44 29,21 60,01 1752,80 20,72 1773,52 0,70 0,04 995,32 0,40
0,02 275,33 1127,72 1606,08 325,18 1085,16 1346,41 202,50 893,98 1720,51
327,94 925,79 1632,93 3797,55 3468,01 402,38 277,64 0,11 0,08 402,38
277,64 57,94 18,60 76,54 2882,59 9311,29 3241,04 1964,91 5827,37 2713,31
1267,46 4780,74 3353,16 1810,90 4674,50 3193,29 21970,94 14715,76
2327,98 1178,11 335,23 78,93 414,16 0,16 0,52 1773,52 995,32 414,16
1317,44 11432,44 10,43 5,85 2,44 7,75 73,54 60,01 49,87
76,54 18,96 150,29 21,45 39,93 33,18 12,62 14,27 0,17 195,39
14,95 2,04 694,12 122,92 18,25 211,02 823,13 171,67 16104,55

6,40 0,58 239,39 16343,94 6,50 411,05 10,85 6,09 2,53
8,06 2,52 69,95 100,00
AMRO Haiti America Caribbean 5152876,00
4458195,00 29,03 6,66 64,31 1294214,01 296915,79
2867065,20 112,60 33,25 163,42 309,27
281158,09 515456,51 398307,30 398307,00 2802,00 3111,27
760,75 60237,44 34305,90 279,34 21195,45 7159,55 481,42
39041,99 27146,34 2,28 2,43 13418,00 4458195,00
309,27 309,27 6,94 2802,00 62,85 398307,30 50,25 24,71 15540,21
69236,60 165927,59 176114,04 35073,23 176114,04
250704,41 260890,85 119850,04 260890,85 5623,45 754,55
5,62 1,90 499,40 3,72 9,36 90,64 3009,74 139,18 3766,92 41,73
3725,19 26,77 1063,30 28459,45 318,81 28778,25 0,65 0,09
19186,10 0,43 0,06 620,33 2452,89 2400,88 469,09 1593,64 1844,80
549,80 2250,94 2873,85 905,13 2185,84 2796,06 6551,18 7781,68 6709,17 4629,33
1,02 0,59 6709,17 4629,33 966,12 310,17 1276,28 29716,60 72391,53
34605,02 24086,09 47408,08 26774,66 26703,24
65269,30 40165,35 48113,55 66503,27 40273,31
194062,19 233402,24 198742,14 138851,29 28618,87
9303,04 37921,90 0,85 0,48 28778,25 19186,10 37921,90
21195,45 153809,14 11,48 7,65 15,13 8,45 57,29 1063,30
883,74 1276,28 309,27 2718,73 462,42 39,11 32,51 11,38 17,01 0,17
1204,29 98,56 16,77 6699,45 956,64 131,05 1308,44 7699,77 1495,36
262386,22 5,89 0,58 21918,86 284305,08 6,38 23414,22
10,12 6,75 13,34 7,46 8,24 54,10 100,00
AMRO Honduras America Central America 4627392,00
3833404,00 29,49 21,39 49,12 1130470,84 819965,12
1882968,04 98,35 91,84 107,33 297,52
270469,30 495860,39 383164,85 383165,00 2409,00 2706,52
1108,54 74459,87 33972,52 518,82 38377,53 10790,90
589,72 36082,34 23181,62 1,80 2,43 23828,00
3833404,00 297,52 297,52 7,76 2409,00 62,84 383164,85 53,17
21,88 15819,52 52699,00 100884,15 134202,61 29369,65
134202,61 169402,67 202721,12 97888,16 202721,12
4419,12 1052,99 4,42 1,79 627,08 2,63 12,99 87,01 6215,89 202,77
5167,22 53,88 5113,34 25,22 914,28 23055,86 242,94 23298,81
0,61 0,14 15697,79 0,41 0,10 220,45 1368,65 2650,46 176,43
832,99 1564,44 182,77 919,34 2264,83 312,92 853,23 1731,87 4003,49 3600,49
6024,97 4157,24 1,50 1,15 6024,97 4157,24 867,60 278,53 1146,13 10624,53
39532,14 36025,04 9559,32 24243,46 21980,36 8930,41
26438,98 29898,82 17038,24 25922,67 24204,63
103294,58 96364,78 155451,12 111265,72 22384,96
7454,80 29839,77 0,78 1,00 23298,81 15697,79 29839,77
38377,53 95507,23 13,75 9,27 17,61 22,65 36,71 914,28
759,89 1146,13 297,52 2320,27 348,59 39,40 32,75 12,82 15,02 0,17
778,77 144,61 40,30 3828,56 854,09 164,52 936,82 4737,50 941,94
203663,06 5,31 0,58 17247,38 220910,44 5,76 18189,32
10,55 7,11 13,51 17,37 8,23 43,23 100,00
AMRO Jamaica America Caribbean 1294078,00
1244925,00 15,22 16,18 68,60 189477,59 201428,87
854018,55 16,48 22,56 48,68 87,72
79748,76 146206,07 112977,42 112977,00 782,00 869,72
205,11 16044,64 10347,40 45,25 4385,10 2188,26 159,86 11659,54
8159,14 2,33 2,43 13812,00 1244925,00 87,72 87,72 7,05
782,00 62,82 112977,42 48,55 21,90 4259,28 17122,87 44154,68
45836,91 12401,78 45836,91 65536,83 67219,06

33783,92		67219,06		5264,32	727,11	5,26	1,72	489,88	3,55
12,97	87,03	11094,64		76,17	1909,02	20,82	1888,20	24,79	296,92
7360,43	81,16	7441,59	0,60	0,08	5113,60	0,41	0,06	51,74	312,71
688,65	58,17	339,64	877,75	41,46	191,14	823,66	70,10	287,25	1221,55
1284,39	1271,69	1684,92	1162,60	1,31	0,91	1684,92	1162,60	242,63	77,89
320,52	2539,01	9538,62	8835,55	3134,23	10171,37		11620,88		2019,82
5710,81	8689,25	3947,64	8860,60	13884,19		32202,04		28063,36	
42244,01		25655,86		6083,14	1718,94	7802,08	0,63	0,35	7441,59
5113,60	7802,08	4385,10	42476,69		11,35	7,80	11,90	6,69	62,25
296,92	246,78	320,52	87,72	795,98	164,56	37,30	31,00	11,02	20,67
0,17	349,51	30,28	7,68	1403,54	308,46	70,53	382,35	1735,50	351,56
67570,63		5,43	0,58	4509,60	72080,23		5,79	4861,17	10,32
7,09	10,82	6,08	6,74	58,93	100,00				
AMRO	Martinique		America	Caribbean				171421,60	
130800,00		10,00	17,00	73,00	13080,00		22236,00		95484,00
				1,14	2,49	5,44	9,07	8246,35	15118,30
11682,32		11682,00		82,00	91,07				
					2,43		130800,00		9,07
9,07	6,94	82,00	62,69	11682,32		x	x		
x	x	x		x	x	x		x	x
x	x	x	x	x	x	x	x	x	x
x	31,20	x	x						
x	x	x	x	x	x	x	x	x	x
x	x	x	x	223,19	154,00	x	x	x	x
x	x		x	x	x	x	x	x	x
x	x	x	x	x	x	x	x	x	x
x				0,00	0,00	0,00	0,00	x	x
x	x	x	x	31,20	25,93		9,07	117,00	50,80
26,66	22,16	7,75	43,42	0,17					
0,00	0,00	0,00		0,00	0,58	0,00	0,00	0,00	0,00
x	x	x	x	x	x	x			
AMRO	Mexico	America	Central	America			57839046,00		
54993594,00		12,48	25,55	61,97	6863200,53		14050863,27		
34079530,20						597,10	1573,70	1942,53	4113,33
3739389,41		6855547,26		5297468,33		5297468,00		34566,00	
38679,33		11155,80		757670,70		411049,02		4357,86	
302336,83		82980,44		6797,94	455333,87		328068,58		2,58
2,43	1076163,00		54993594,00		4113,33	4113,33	7,48	34566,00	
62,85	5297468,33		50,34	18,72	207047,46		647115,70		
1746479,82		1650275,20		406392,67		1650275,20		2600642,98	
2504438,36		1260555,83		2504438,36		4728,99	50891,67		4,73
1,55	34176,65		3,18	10,97	89,03	19568,88		2112,21	50636,76
649,22	49987,54		23,67	13116,20		310407,77		4031,46	
314439,23		0,57	6,15	191873,85		0,35	3,75	5419,91	18250,15
35645,39		1700,76	5341,71	11470,93		1681,30	8735,68	36578,63	
1239,85	4203,55	13770,50		46417,97		32643,43		75307,80	
51962,43		1,62	1,59	75307,80		51962,43		10844,32	
3481,48	14325,81		267777,97		544695,82		452665,23		97607,74
169746,50		156614,30		84088,91		257214,67		415416,99	
85491,26		147145,81		185228,87		1282921,20		774155,94	
2081391,80		1232316,16		299720,42		82565,18		382285,60	
0,70	0,55	314439,23		191873,85		382285,60		302336,83	
1313502,85		12,09	7,38	14,70	11,63	54,21	13116,20		10901,26
14325,81		4113,33	35254,78		7124,00	37,20	30,92	11,67	20,21
0,17	10093,36		1833,48	470,72	84029,28		21019,11		3612,70
12083,74		106252,63		19643,84		2524082,20		4,59	0,58

220961,08 2745043,28 4,99 240604,92 11,45 6,99 13,93
11,01 8,77 47,85 100,00
AMRO Montserrat America Caribbean 4521,00
4,00 6,00 90,00 180,84 271,26 4068,90
0,02 0,03 0,23 0,28 252,77 463,40 358,08 358,00 3,00 3,28
2,43
4521,00 0,28 0,28 6,15 3,00 66,36 358,08 x x
x x x x x x x x
x x x x x x x x x x
x 1,08 x x
x x x x x x x x x x
x x x x 0,00 0,00 x x x x
x x x x x x x x x
x x x x x x x x x x
x 0,00 0,00 0,00 0,00 x x
x x x x 1,08 0,90 0,28 4,17 1,92
x 21,47 6,66 46,04 0,17
0,00 0,00 0,00 0,00 0,58 x x x x
x x x x x x x
AMRO Netherlands Antilles America North America
105592,80 62000,00 1,10 17,60 81,30 682,00 10912,00
50406,00 0,06 1,22 2,87 4,15
3776,93 6924,37 5350,65 5351,00 39,00 43,15
2,43 62000,00 4,15
4,15 6,70 39,00 62,90 5350,65 x x x
x x x x x x x
x x x x x x x x x x
x 14,79 x x
x x x x x x x x x x
x x x x 137,48 94,86 x x x x
x x x x x x x x x
x x x x x x x x x x
x 0,00 0,00 0,00 0,00 x x
x x x x 14,79 12,29 4,15 55,44 24,21
26,67 22,17 7,49 43,67 0,17
0,00 0,00 0,00 0,00 0,58 x x x x
x x x x x x x
AMRO Nicaragua America Central America 3060596,00
2903210,00 30,60 16,24 53,16 888382,26 471481,30
1543346,44 77,29 52,81 87,97 218,07
198241,74 363443,18 280842,46 280842,00 1825,00 2043,07
392,36 37049,54 22893,58 201,08 15951,97 5349,71 191,29
21097,57 17543,87 4,94 2,43 12621,00 2903210,00
218,07 218,07 7,51 1825,00 62,86 280842,46 52,73 18,58 11498,12
33904,56 173181,56 88161,53 23345,62 88161,53
218584,24 133564,21 68748,30 133564,21 7529,05 950,24
7,53 1,56 752,86 5,97 7,91 92,09 4347,26 39,17 1038,06 12,86
1025,21 26,17 692,43 18123,14 227,33 18350,48 0,63 0,08
12218,51 0,42 0,05 142,77 775,45 1817,47 48,58 285,94 617,45
95,73 460,79 1798,84 56,99 214,80 711,11 2064,39 1664,97 3984,97 2749,63
1,93 1,65 3984,97 2749,63 573,84 184,23 758,06 7235,44 22338,15
24386,02 3041,14 8582,66 8683,69 4760,19 13321,61 21773,20
3881,37 7113,94 9564,45 52220,63 39523,00 100803,62 65270,71
14515,72 4373,14 18888,86 0,65 0,55 18350,48 12218,51
18888,86 15951,97 68154,39 8,40 5,59 8,64 7,30
70,08 692,43 575,50 758,06 218,07 1860,50 374,51 37,22 30,93 11,72

20,13 0,17 1387,50 93,97 19,30 3936,38 580,45 91,04 1487,90 4547,17
1001,82 134566,03 4,64 0,58 10917,76 145483,79 5,01
11919,58 12,61 8,40 12,98 10,96 8,19 46,85 100,00
AMRO Paraguay America South America 3625051,00
3385793,00 18,72 18,14 63,14 633820,45 614182,85
2137789,70 55,14 68,79 121,85 245,78
223440,79 409641,45 316541,12 316541,00 2128,00 2373,78
585,84 51171,22 29111,03 308,69 20983,92 5078,43 277,15
30187,30 24032,59 3,90 2,43 35304,00 3385793,00
245,78 245,78 7,26 2128,00 62,85 316541,12 51,53 22,21 12664,08
47257,11 188570,67 118838,82 28076,08 118838,82
248491,85 178760,00 87997,27 178760,00 7339,25 2591,05
7,34 1,77 1966,25 5,57 6,72 93,28 10427,10 119,72 3010.50
33,44 2977,06 24,87 807,53 20080,62 225,56 20306,18 0,60
0,21 16873,83 0,50 0,18 238,71 1080,81 1495,57 136,29 564,75
1018,87 86,36 463,92 1387,39 105,58 393,47 1103,11 2858,71 1879,49 4719,90
3256,74 1,65 1,73 4719,90 3256,74 679,67 218,20 897,87 11760,55
32347,79 20267,04 7531,39 17412,76 14102,62 4200,09
13559,33 15970,99 6261,13 12772,87 14044,49 80509,04
46798,58 132925,48 81091,49 19141,27 5433,13 24574,40
0,73 0,62 20306,18 16873,83 24574,40 20983,92
96021,68 8,17 6,79 9,89 8,44 66,70 807,53 671,16 897,87
245,78 2147,08 422,61 37,61 31,26 11,45 19,68 0,17 642,34 49,74
9,97 3009,77 496,11 76,75 695,40 3531,47 701,66 179461,66 5,30
0,58 14204,00 193665,67 5,72 14905,66 10,49 8,71
12,69 10,84 7,70 49,58 100,00
AMRO Peru America South America 18920065,00
18049074,00 27,37 15,20 57,43 4940031,55 2743459,25
10365583,20 429,78 307,27 590,84 1327,89
1207171,29 2213147,37 1710159,33 1710159,00 11345,00
12672,89 1608,74 158090,35 106206,91 658,99 44139,91
11217,50 949,75 113950,44 94989,40 5,01 2,43
202014,00 18049074,00 1327,89 1327,89 7,36 11345,00 62,86
1710159,33 49,96 19,96 66339,70 226494,40 1157276,35
572796,00 117593,03 572796,00 1450110,45 865630,10
410427,13 865630,10 8034,27 16230,34 8,03 1,62 12952,80
6,41 6,13 93,87 11192,49 481,86 11510,24 140,10 11370,15
23,60 4304,78 101577,16 1251,61 102828,76 0,57 1,15 62198,06
0,34 0,70 677,49 1979,97 6311,38 538,77 994,89 2940,62 262,18 1072,08
5744,50 414,23 918,86 2987,78 7275,13 5578,10 24634,37 16997,73
3,39 3,05 24634,37 16997,73 3547,35 1138,85 4686,20 35167,63
60293,20 75843,17 32465,05 32624,99 36757,64
13605,05 32195,72 62431,93 26570,88 32232,45
37653,41 198084,47 137965,89 670735,66 420412,93
96585,93 28167,67 124753,60 0,69 0,24 102828,76
62198,06 124753,60 44139,91 531709,77 7,09 4,29
8,60 3,04 76,97 4304,78 3577,83 4686,20 1327,89 11564,52 2354,02
37,22 30,94 11,48 20,36 0,17 1872,05 388,07 104,72 9979,59 3421,32
848,26 2295,02 13683,66 2652,46 868282,57 4,81 0,58 72107,58
940390,15 5,21 74760,04 10,93 6,61 13,27 4,69 7,95
56,54 100,00
AMRO Saint Kitts and Nevis America Caribbean 18170,00
18170,00 46,00 24,00 30,00 8358,20 4360,80 5451,00
0,73 0,49 0,31 1,53 1387,53 2543,80 1965,66 1966,00 11,00
12,53 4,03 324,67 210,61 0,72 74,10 39,34 3,31 250,57 171,28
2,16 2,43 927,00 18170,00 1,53 1,53 8,40 11,00 60,54

1965,66	x	x			x	x	x		x
x	x		x	x		x	x	x	x
x	x	2,10	x	x	x	x	4,33	x	x
0,00	0,00	95,00			1,96	16,35	13,80	2,08	16,15
14,31	0,52	6,43	16,12	1,14	8,98	23,14	45,92	30,16	23,66
16,32	x	x	x	x	x	x		92,15	484,16
207,37	105,89	482,98	215,22	25,03	186,99	191,35	66,13	277,91	281,63
x	x	x	x	x	x				0,00
95,00	0,00	74,10	x	x	x	x	x	x	4,33
3,60		1,53	16,13	6,67	26,87	22,33	9,46	41,33	0,17
7,43	1,41	0,19	34,40	12,46	1,55	8,89	47,38	9,34	9,34
0,05	0,58	0,00	9,34	0,05	9,34	x	x	x	x
x	x	x							
AMRO	Saint Lucia		America Caribbean					108318,00	
83724,00		9,97	14,69	75,34	8347,28	12299,06		63077,66	
			0,73	1,38	3,60	5,70	5181,03	9498,56	7339,79
73400,00		53,00	58,00	12,80	987,71	637,28	2,58	198,68	72,80
10,22	789,03	564,48	2,51	2,43	1703,00	83724,00		5,70	5,70
6,81	53,00	63,30	7339,79	48,73	21,97	277,74	1164,56	3088,00	2989,54
725,12	2989,54	4530,29	4431,84	2167,41	4431,84	5410,98	x	5,41	1,72
x	3,69	9,68	90,32	x	3,24	84,33	1,01	83,32	25,72
19,97	513,51	6,22	519,74	0,62	0,01	339,87	0,41		4,49
25,46	44,24	5,18	25,80	53,13	1,60	10,81	47,15	3,48	16,10
68,62	93,38	70,58	141,03	97,31	1,51	1,38	141,03	97,31	20,31
6,52	26,83	216,04	776,41	609,83	268,25	788,55	724,99	77,76	326,08
531,71	200,39	520,90	821,87	2494,19	1576,32	3766,99	2173,45	542,45	145,62
688,07	0,82	0,24	519,74	339,87	688,07	198,68	2685,48	11,47	7,50
15,19	4,39	61,45	19,97	16,60	26,83	5,70	48,47	6,20	41,20
34,24	11,76	12,80	0,17	18,14	2,93	0,65	80,22	24,24	5,18
21,29	106,19	21,16	4453,00	5,32	0,58	397,70	4850,70	5,79	418,87
10,71	7,01	14,18	4,10	8,64	55,36	100,00			
AMRO	Saint Pierre and Miquelon				America	North America			
3194,00	3194,00	18,00	41,00	41,00	574,92	1309,54	1309,54		
	0,05	0,15	0,07	0,27	246,66	452,22	349,44	349,00	2,00
2,27									
2,43		3194,00	0,27	0,27	8,50	2,00	62,62	349,44	x
x			x	x	x		x	x	x
x	x		x	x	x	x	x	x	x
x	x	x	x	0,76	x	x			
		x	x	x	x	x	x	x	x
x	x	x	x	x	x	4,16	2,87	x	x
x	x	x	x		x	x	x	x	x
x	x	x	x	x	x	x	x	x	x
x	x	x				0,00	0,00	0,00	0,00
x	x	x	x	x	x	0,76	0,63		0,27
2,90	1,24	26,23	21,80	9,34	42,63	0,17			
		0,00	0,00	0,00		0,00	0,58	0,00	0,00
0,00	0,00	x	x	x	x	x	x	x	
AMRO	Saint Vincent and the Grenadines					America Caribbean			
57953,00		47167,00		10,08	19,88	70,04	4754,43	9376,80	33035,77
				0,41	1,05	1,88	3,35	3042,61	5578,13
4310,37	4310,00	30,00	33,35	4,80	434,71	311,78	0,53	53,94	28,02
4,28	380,77	283,76	2,92	2,43		47167,00		3,35	3,35
7,10	30,00	63,60	4310,37	49,32	22,68	165,06	680,29	2168,32	1831,05
462,26	1831,05	3013,67	2676,40	1307,62	2676,40	6389,37	x	6,39	1,79
x	4,60	11,40	88,60	x	1,95	50,09	0,62	49,47	25,37

11,25	285,39	3,58	288,97			162,73	0,35		4,87
26,69	52,05	3,38	16,72	33,44	2,36	13,82	59,68	2,80	11,57
40,83	80,16	64,05	75,46	52,06	0,94	0,81	75,46	52,06	10,87
3,49	14,35	228,85	796,68	697,36	173,08	513,97	467,34	112,58	401,03
706,79	154,85	367,05	525,16	2100,81	1446,16	1977,53	1175,55	284,76	78,76
363,53	0,77	0,11	288,97	162,73	363,53	53,94	1807,24	9,59	5,40
12,06	1,79	71,16	11,25	9,35	14,35	3,35	28,34	4,40	39,69
32,99	11,81	15,51	0,17	16,27	2,13	0,48	48,48	14,51	2,84
18,55	63,93	13,69	2690,09	5,70	0,58	210,12	2900,21	6,15	223,81
9,96	5,61	12,53	1,86	7,72	62,31	100,00			
AMRO	Suriname		America	South America				219507,00	
204277,00		8,08	24,33	67,59	16505,58		49700,59		
138070,82						1,44	5,57	7,87	14,87
13520,44		24787,48		19153,96		19154,00		128,00	142,87
30,23	2455,60	1493,53	9,10	651,77	206,71	21,13	1803,83	1286,82	2,49
2,43	3808,00	204277,00		14,87	14,87	7,28	128,00	62,66	19153,96
48,92	24,46	727,49	3131,44	8131,92	7944,68	1624,70	7944,68	11990,85	
11803,62		5483,64	11803,62		5869,90	223,53	5,87	1,89	151,59
3,98	8,88	91,12	18641,35		10,83	292,96	3,06	289,90	26,77
48,72	1304,17	13,77	1317,94	0,65	0,02	761,03	0,37	0,01	31,72
160,58	156,25	23,88	120,66	140,49	11,67	73,52	173,81	17,12	79,28
191,01	435,75	303,20	285,80	197,20	0,66	0,65	285,80	197,20	41,16
13,21	54,37	1503,95	4884,83	2180,58	1215,73	3665,03	2008,19	563,35	2177,95
2187,21	942,81	2472,06	2542,70	12665,81		7732,81	8307,41	5029,45	1196,27
336,97	1533,24	0,75	0,32	1317,94	761,03	1533,24	651,77	7539,64	10,99
6,35	12,79	5,44	64,44	48,72	40,49	54,37	14,87	129,00	24,91
37,77	31,39	11,53	19,31	0,17	93,70	12,54	2,19	412,71	110,50
21,43	106,96	530,36	105,79	11909,41		5,83	0,58	886,21	12795,62
6,26	992,01	10,30	5,95	11,98	5,09	7,75	58,92	100,00	
AMRO	Turks and Caicos Islands				America	Caribbean			
4848,00	4848,00	20,00	23,70	56,30	969,60	1148,98	2729,42		
	0,08	0,13	0,16	0,37	335,11	614,36	474,73	475,00	3,00
3,37									
2,43	925,00	4848,00	0,37	0,37	7,60	3,00	61,88	474,73	x
x			x	x	x		x	x	x
x	x		x	x	x	x	x	x	x
x	x	x	x	1,16	x	x			
		x	x	x	x	x	x	x	x
x	x	x	x	x	x	6,31	4,36	x	x
x	x	x	x		x	x	x	x	x
x	x	x	x	x	x	x	x	x	x
x	x	x				0,00	0,00	0,00	0,00
x	x	x	x	x	x	1,16	0,96		0,37
4,33	1,84	26,71	22,20	8,51	42,58	0,17			
		0,00	0,00	0,00		0,00	0,58	0,00	0,00
0,00	0,00	x	x	x	x	x	x	x	
AMRO	Venezuela, Bolivarian Rep. of				America	South America			
11996396,00		14580285,00		7,86	15,34	76,81	1146010,40		
2236615,72		11199116,91						99,70	250,50
638,35	988,55	898685,03		1647589,22		1273137,12		1273137,00	
9164,00	10152,55		4612,27	317874,51		130355,55		3230,33	
216916,65		59791,02		1381,94	100957,86		70564,52		2,32
2,43	482359,00		14580285,00		988,55	988,55	6,78	9164,00	62,85
1273137,12		48,64	21,99	48083,92		201546,49		486229,95	
507887,15		88861,90		507887,15		735860,36		757517,56	
338492,32		757517,56		5046,95	24344,44		5,05	1,71	16085,93

3,33 9,02 90,98 33082,96 577,44 15602,77 169,11 15433,65
26,73 3477,46 92944,48 1018,41 93962,89 0,64 3,11 41419,93
0,28 1,37 1669,14 8788,14 11083,92 590,90 2842,09 4622,78 669,59
3581,12 10402,54 551,95 1813,01 5194,55 19125,83 11814,70
15619,59 10777,53 0,82 0,91 15619,59 10777,53
2249,22 722,09 2971,32 78407,23 257461,23 150495,30 31100,84
85829,91 64567,60 32938,55 103130,46 122183,23
32790,27 58796,54 66506,36 524486,85 290552,36
428335,36 265045,78 61680,29 17758,07 79438,36
0,54 1,49 93962,89 41419,93 79438,36 216916,65
325779,73 12,77 5,63 10,80 29,48 41,33 3477,46 2890,22 2971,32
988,55 10071,46 2715,23 34,53 28,70 9,82 26,96 0,17 3862,88
755,01 186,73 12333,91 4237,16 816,52 4680,14 16843,24 3572,88
761090,44 5,22 0,58 45915,37 807005,81 5,53 49488,25
11,64 5,13 9,84 26,88 6,13 40,37 100,00
AMRO Virgin Islands, U.S. America North America 51757,00
47700,00 1,00 19,00 80,00 477,00 9063,00 38160,00
0,04 0,34 2,18 2,56 2328,19 4268,36 3298,27
30,00 32,56 17,34 841,32 384,73 3,49 258,44 98,92 13,85 582,88
285,81 0,96 1,08 3984,00 47700,00 2,56 5,37 30,00
62,89 3298,27 45,67 21,45 0,00 643,60 619,19 694,82 285,81 694,82
1262,79 1338,42 929,41 1338,42 2647,36 105,47 2,65 1,35 x 1,30
0,00 100,00 x 11,07 245,67 3,03 2422,64 218,85 11,38 2489,75
3,11 2492,86 155,05 0,33 0,01 5,75 66,65 100,85
1,91 18,26 32,30 1,50 22,19 98,90 0,92 8,65 34,28 136,95
77,65 67,39 46,50 0,49 0,60 67,39 46,50 9,70 3,12 12,82
262,29 1901,70 1512,38 96,28 540,85 500,81 70,28 622,73 1255,55 58,32
279,91 469,10 3472,19 1606,12 1708,58 961,76 246,04 64,44 310,47 0,65
0,54 2492,86 155,05 310,47 258,44 -1878,41 197,41 12,28 24,59
20,47 -154,74 11,38 9,46 12,82 0,00 26,64 5,80 42,71 35,50
0,00 21,79 0,17 7,92 2,10 0,63 39,65 20,51 6,24 10,23
62,24 12,03 1350,45 2,83 0,58 179,45 1529,90 3,21 191,48 162,94
10,13 20,29 16,89 12,52 -122,78 100,00
AMRO TOTAL 309345398,80
272116940,10 39472816,43 55505911,91
177148662,93 0,00 0,00 0,00 0,00 3434,14 2109,22 10097,47
19747,60 17952363,90 32912667,15 25432515,52
171036,92 190784,79 62841,31 4360615,07 2332995,36
25721,79 1816369,01 530819,62 37105,67 2543663,17
1801889,93 2,43 2,43 4476667,00 272116940,10 19747,60
19745,04 7,26 171036,92 62,85 25429217,25 49,98 19,99
988087,85 3404070,63 8677044,78 8660156,65 2209883,92
8660156,65 13069203,26 13052315,12 6602042,39 13052315,12
4802,79 215004,88 4,80 1,61
64889,62 1555222,88
0,57 25,59 1145819,06 0,42 18,85 23984,52 101997,48
167105,67 11820,06 47069,93 91262,26 9989,74 52789,39
174880,43 11174,54 36237,51 107337,00 270994,63
204263,65 402775,00 277915,00 1,49 1,36 402775,00
277915,00 57999,60 18620,31 76619,91 1162256,68
3013195,67 2201063,46 634919,11 1423802,21 1246216,56
480414,45 1517743,99 2027299,32 648091,58 1170266,08
1385722,57 7381960,97 4953476,34 10667687,19 6541845,87
1536146,96 438303,67 1974140,16 0,73 0,67 1555222,88
1145664,01 1974140,16 1816110,58 6561177,50
64889,62 53941,13 73921,04 19745,04

170802,04 32226,25 37,99 31,58 11,56 18,87 0,17 60169,57
9919,37 2407,51 296722,16 79043,82 14638,07 70891,45
380645,33 74955,11 13127270,23 4,82 0,58 1141053,01
14268323,24 5,24 1216008,12 10,90 8,03 13,84 12,73 8,52
45,98 100,00

0,00 0,00

EMRO Afghanistan Asia Southern Asia 10701549,00
6396534,00 42,50 18,55 38,96 2718526,95 1186557,06
2492089,65 358,85 156,63 184,41 699,89
636259,75 1166476,20 901367,98 901368,00 5605,00 6304,89
3818,35 288396,82 97478,91 3001,45 189607,98 22582,19
816,90 98788,84 74896,73 3,13 2,49 19807,00
6396534,00 699,89 699,89 10,94 5605,00 87,63 901367,98 55,65
29,25 38947,52 163930,89 519154,45 412666,42 80162,50
412666,42 722032,86 615544,83 283040,91 615544,83
11287,88 2235,79 11,29 3,17 1607,57 8,12 6,12 93,88 3096,52
162,32 5560,89 52,09 5508,80 33,94 1106,83 37563,53 355,19 37918,72
0,59 0,12 56698,68 0,89 0,18 4282,82 6981,06 6270,86 1418,37
2147,69 3129,99 3492,04 6643,69 6957,32 2289,81 3460,24 4256,41 17963,55
19623,69 21034,21 15343,21 1,17 0,78 21034,21
15343,21 3028,93 1028,00 4056,92 201775,06 221155,12 89385,09
76638,42 70955,39 45571,43 166808,68 204517,87
100588,34 123543,54 114509,10 64300,80 615509,49
664342,23 720723,84 519430,39 103784,23 34801,84
138586,07 2,17 2,96 37918,72 56698,68 138586,07
189607,98 192733,38 5,25 7,85 19,19 26,26 41,44 1106,83
2860,89 4056,92 699,89 5108,86 441,25 21,66 56,00 13,70 8,64 0,17
10117,84 607,38 60,45 24759,70 3399,58 386,74 10745,37
28288,20 6479,57 622024,40 9,72 0,58 80102,75
702127,15 10,98 86582,32 5,40 8,08 19,74 27,00 12,33
27,45 100,00
EMRO Djibouti Africa Eastern Africa 414572,00
82735,00 24,55 13,44 62,00 20311,44 11119,58 51295,70
2,68 1,47 3,80 7,94 7222,52 13241,30
10231,91 10232,00 72,00 79,94 57,21 5687,42 3447,29 32,14
2145,55 559,60 25,07 3541,86 2887,69 4,41 2,49 3384,00 82735,00
7,94 7,94 9,60 72,00 87,02 10231,91 49,34 26,10 392,03
1878,97 8432,51 4807,93 3026,01 4807,93 10703,50 7078,92 5297,01 7078,92
12937,09 437,79 12,94 2,74 344,90 10,19 4,95 95,05 40901,67
7,42 202,10 2,23 199,87 26,94 14,32 385,63 4,30 389,93 0,47
0,02 2002,08 2,42 0,08 48,26 148,17 115,17 41,22 128,84 106,24

17,88 61,18 105,05 24,13 73,91 108,23 440,28 248,20 814,85 594,39
1,85 2,39 814,85 594,39 117,34 39,82 157,16 2389,80 4486,21 1811,03
2282,77 4023,45 1725,25 875,24 1805,13 1465,16 1480,68 2422,49 1714,55 14360,99
7643,45 26578,70 18304,72 3827,33 1226,42 5053,75 6,11 2,59
389,93 2002,08 5053,75 2145,55 -2512,39 3,64 18,70 47,22 20,05
10,39 14,32 37,00 157,16 7,94 x x x x x
x 0,17 253,03 67,53 9,93 555,96 148,71 23,00 323,87 712,34
172,01 7250,94 8,76 0,58 2921,07 10172,00 12,29 3093,08 3,83
19,68 49,68 21,09 30,41 -24,70 100,00
EMRO Egypt Africa Northern Africa 29776956,00
26133514,00 20,62 26,94 52,44 5388730,59 7040368,67
13704414,74 46,00 711,31 929,33 1014,13 2654,77
2413425,27 4424612,99 3419019,13 3419019,00 22898,00
25552,77 4103,39 381634,64 239539,88 1416,84 85782,42
14187,66 2686,55 295852,21 225352,22 3,20 2,49
363069,00 26133514,00 2654,77 2654,77 10,16 22898,00 87,62
3419019,13 50,53 26,24 134148,61 600885,91 1947306,93
1519903,94 251935,93 1519903,94 2682341,46 2254938,46
986970,45 2254938,46 10263,99 37265,37 10,26 2,81
27053,64 7,45 5,99 94,01 13892,85 503,42 15966,33
154,77 15811,56 31,41 4522,04 142029,58 1390,24 143419,82
0,55 1,99 162480,89 0,62 2,26 11509,19 49800,43
44281,94 3926,11 8751,85 9823,46 5553,01 31682,65 39057,35
2424,91 8577,07 10227,44 92022,71 64665,91 58527,49
42692,33 0,64 0,66 58527,49 42692,33 8427,96 2860,39
11288,34 570040,68 1468674,39 635385,49 239576,71
290994,92 159784,76 279699,89 899421,17 566716,59
157166,68 296445,53 173073,43 2834343,44 1879329,93
1802674,58 1240730,72 259585,14 83128,96 342714,10
1,31 0,33 143419,82 162480,89 342714,10 85782,42
1520541,23 5,35 6,06 12,78 3,20 72,62 4522,04 11688,39
11288,34 2654,77 25952,82 7087,61 17,42 45,04 10,23 27,31
0,17 28472,56 2322,24 294,06 61596,52 11965,22 1159,10
30892,82 73948,10 17403,59 2272342,05 8,70 0,58
198088,75 2470430,80 9,45 215492,34 5,81 6,58 13,87
3,47 8,72 61,55 100,00
EMRO Iran, Islamic Rep. Of Asia Southern Asia
28212245,00 24046846,00 17,37 31,39 51,24 4176937,15
7548304,96 12321603,89 551,36 996,38
911,80 2459,53 2235936,95 4099217,74 3167577,35 3167577,00
21070,00 23529,53 4398,20 412273,60 249817,79 2290,71
147898,37 32766,81 2107,49 264375,23 217050,98 4,59
2,49 191718,00 24046846,00 2459,53 2459,53 10,23 21070,00
87,62 3167577,35 50,26 22,46 123616,32 473133,28
2205190,37 1211011,31 252232,58 1211011,31 2801939,97
1807760,91 848982,18 1807760,91 11652,01 22338,99
11,65 2,48 17581,29 9,17 5,67 94,33 7972,69 574,91 16767,08
188,83 16578,25 28,84 4160,97 119986,85 1366,68 121353,53
0,50 0,97 158432,62 0,66 1,26 5384,63 18878,39 34056,97
1430,67 5218,11 13971,33 1843,31 9583,53 33051,96 945,42 4010,19
15245,55 46921,23 32481,62 55452,00 40448,95
1,18 1,25 55452,00 40448,95 7985,09 2710,08 10695,17
265589,25 568032,23 422977,40 89272,71 175179,90
186109,80 93751,76 277604,69 397469,05 70109,33
145797,63 207855,85 1301103,17 789038,38 1537657,24
982579,59 221422,64 65832,83 287255,47 1,19 0,62

121353,53 158432,62 287255,47 147898,37 1092820,91
4,33 5,65 10,25 5,28 74,48 4160,97 10755,12 10695,17
2459,53 23589,48 6213,86 17,64 45,59 10,43 26,34 0,17 29073,34
3366,97 372,88 84428,48 21941,62 3112,46 32564,61
107407,59 23235,38 1830996,29 7,61 0,58 166033,66
1997029,96 8,30 189269,05 6,08 7,93 14,38 7,41 9,48
54,72 100,00
EMRO Iraq Asia Western Asia Y 10469143,00
7103293,00 18,27 22,93 58,80 1297771,63 1628785,08
4176736,28 171,31 215,00 309,08 695,38
632167,25 1158973,29 895570,27 895570,00 6224,00 6919,38
2015,29 155657,36 71401,53 1212,67 78890,44 14533,69
802,62 76766,92 56867,84 2,86 2,49 167224,00
7103293,00 695,38 695,38 9,79 6224,00 87,62 895570,27 53,07
24,79 36904,10 154309,49 449321,61 391823,62 65201,90
391823,62 640535,20 583037,21 256415,48 583037,21
9017,44 15079,32 9,02 2,69 10577,82 6,33 7,06 92,94
23541,76 543,44 13842,01 150,14 13691,89 25,19 1229,13
30967,65 339,58 31307,23 0,44 0,74 39301,26 0,55
0,93 2747,02 11999,01 12747,38 1789,60 5103,71 7043,57 1347,94
6348,35 11658,23 1144,41 3658,79 7372,32 28236,32 18843,01
20577,41 15010,00 0,73 0,80 20577,41 15010,00
2963,15 1005,67 3968,82 129549,66 356468,08 182193,61
102020,42 160185,73 105496,26 65678,37 184829,97
152212,26 71210,97 122476,16 107092,07 844120,52
530630,25 615158,57 422690,36 88582,83 28320,25
116903,09 1,65 1,11 31307,23 39301,26 116903,09
78890,44 316635,19 4,89 6,14 18,25 12,32 58,41 1229,13
3177,00 3968,82 695,38 6127,56 1026,06 20,06 51,85 11,35 16,74 0,17
8271,10 721,77 69,10 16720,33 2794,62 297,05 9015,90 19613,96
4752,56 587789,76 8,27 0,58 67569,98 655359,75 9,23
72322,54 4,78 6,00 17,84 12,04 11,04 48,31 100,00
EMRO Jordan Asia Western Asia Y 2649637,00
2294531,00 2,47 24,45 73,09 56674,92 561012,83
1677072,71 7,48 74,05 124,10 205,64
186943,78 342730,27 264837,03 264837,00 2010,00 2215,64
329,98 30831,79 18516,35 156,12 11361,14 3084,19 173,87
19470,65 15432,16 3,82 2,49 43698,00 2294531,00
205,64 205,64 8,96 2010,00 87,60 264837,03 53,02 23,23 10902,58
46687,53 182468,40 120090,29 19494,72 120090,29
240058,50 177680,40 77084,83 177680,40 10462,20
4571,77 10,46 2,51 3475,00 7,95 6,23 93,77 19044,41 111,98
2706,47 30,74 2675,73 23,89 397,04 9487,08 108,99 9596,07 0,42 0,18
10266,90 0,45 0,20 570,92 1632,95 1678,79 103,61 503,99 1061,10
104,09 682,01 1442,19 78,03 426,37 1186,64 3724,77 2166,79 5207,94 3798,88
1,40 1,75 5207,94 3798,88 749,94 254,53 1004,47 27571,78 50498,55
24322,69 7948,60 17975,76 16417,90 5174,36 19948,94
19964,31 7323,14 16082,66 19022,64 117574,88 61524,74
164392,18 107867,03 23672,47 7227,09 30899,56 1,35
0,50 9596,07 10266,90 30899,56 11361,14 115556,72
4,00 4,28 12,87 4,73 74,12 397,04 1026,24 1004,47 205,64 2237,41
608,49 17,75 45,87 9,19 27,20 0,17 3051,04 226,07 24,91 6405,12
1136,13 130,41 3285,41 7584,72 1804,44 179484,84 7,82 0,58 17859,95
197344,79 8,60 19664,39 4,86 5,20 15,66 5,76 9,96
58,56 100,00
EMRO Lebanon Asia Western Asia Y 2423712,00

1590401,00 11,32 23,59 65,10 180033,39 375175,60
1035351,05 23,76 49,52 76,62 149,90
136275,97 249839,27 193057,62 193058,00 1394,00 1543,90
397,37 29182,99 14232,22 205,23 15357,66 4679,03 192,14
13825,33 9553,19 2,24 2,49 33383,00 1590401,00 149,90
149,90 9,43 1394,00 87,65 193057,62 52,03 22,23 7799,77 30995,01
72727,41 80446,33 12970,79 80446,33 111522,19
119241,11 51765,57 119241,11 7012,21 2340,88 7,01 2,44
1526,57 4,57 10,06 89,94 20990,30 84,04 2319,40 27,08 2292,32
27,28 275,20 7506,41 88,68 7595,09 0,48 0,16 5528,43 0,35 0,12
551,33 2106,34 4683,01 35,04 121,71 639,53 179,53 997,52 3733,69 34,23
125,88 805,61 4588,59 2850,26 4763,88 3474,97 1,04 1,22 4763,88 3474,97
686,00 232,82 918,82 27234,21 61218,25 61688,58 2725,95
4841,76 9341,56 8794,17 28594,37 45842,02 3319,35 6346,66 12279,86
119696,87 66428,51 124269,38 80987,95 17894,79
5426,19 23320,98 1,47 0,97 7595,09 5528,43 23320,98 15357,66
67438,95 6,81 4,96 20,91 13,77 53,55 275,20 711,32 918,82
149,90 1336,40 199,98 20,59 53,23 11,22 14,96 0,17 1060,71 161,11
34,02 3438,89 1118,82 232,76 1233,16 4635,29 974,16 120215,27 7,56
0,58 13479,53 133694,80 8,41 14453,69 5,68 4,14
17,44 11,49 10,81 50,44 100,00
EMRO Libya Africa Northern Africa 2422944,00
1978407,00 16,41 24,43 59,17 324656,59 483324,83
1170623,42 42,85 63,80 86,63 193,28
175708,80 322132,80 248920,80 248921,00 1733,00 1926,28
304,76 25788,84 14722,83 136,70 8193,44 1393,23 168,06 17595,40
13329,60 3,12 2,49 25418,00 1978407,00 193,28 193,28
9,77 1733,00 87,60 248920,80 49,75 25,38 9614,80 43988,93
139424,77 111291,06 15299,48 111291,06 193028,50
164894,78 68903,21 164894,78 9756,76 2479,97 9,76 2,71
1791,29 7,05 6,00 94,00 12847,71 73,58 2192,27 22,17 2170,09
29,49 342,34 10096,49 103,15 10199,64 0,52 0,13 8981,44
0,45 0,12 678,45 1463,58 2257,52 187,08 368,07 886,69 388,68 1082,89
1955,69 200,63 445,78 964,98 3745,25 3091,54 4762,37 3473,87 1,27 1,12
4762,37 3473,87 685,78 232,75 918,53 33483,45 44480,44 29870,28
11164,97 12537,55 12452,89 19052,87 31870,45
24401,18 12806,42 16314,34 13992,14 115774,13
92841,85 147215,37 104323,48 21199,01 6989,67 28188,69
1,42 0,41 10199,64 8981,44 28188,69 8193,44 109331,58
5,28 4,65 14,60 4,24 71,22 342,34 884,86 918,53 193,28 1892,61
472,13 18,09 46,75 10,21 24,95 0,17 1973,76 206,29 25,73 4848,36
1076,28 134,66 2188,63 5969,52 1354,25 166249,04 8,40 0,58 16293,06
182542,10 9,23 17647,31 5,59 4,92 15,44 4,49 9,67
59,89 100,00
EMRO Morocco Africa Northern Africa 12084530,00
10996311,00 33,25 23,09 43,66 3656273,41 2539048,21
4800989,38 482,63 335,15 355,27 1173,06
1066414,24 1955092,78 1510753,51 1732923,15 9635,00 10808,06
3025,46 254375,76 138077,42 1676,80 106874,64 23516,44
1348,66 147501,13 114560,98 3,48 2,49 112871,00
10996311,00 1173,06 1173,06 10,67 9635,00 87,62 1510753,51 49,71
24,42 58315,81 235328,94 834891,04 601290,58
131012,64 601290,58 1128535,80 894935,33 424657,39
894935,33 10262,86 11583,79 10,26 2,67 8569,69 7,59
6,63 93,37 10264,44 458,00 13541,70 131,40 13410,30
29,28 1902,76 55713,03 545,90 56258,93 0,51 0,58 6862,46

0,06   0,07   2702,87 16635,75   21105,52   699,80 4597,04 8829,39
1872,34 10767,19   18927,85   985,20 4509,20 9342,34 34613,76
27557,33   23752,50   17326,04   0,69   0,63   23752,50
17326,04   3420,36 1160,84 4581,20 129307,57   485114,96
285842,25   42173,74   144478,17   123982,90   89604,87
312411,24   248741,67   60560,00   152942,38   134679,62
937682,83   743325,58   643452,54   467348,92   92657,17
31312,38   123969,54   1,13   0,97   56258,93   6862,46
123969,54   106874,64   600969,77   4,99   0,61   10,98   9,47
73,95   1902,76 4918,18 4581,20 1173,06 11145,03   3151,04 17,07   44,13
10,53   28,27   0,17   6639,61 1622,33 179,44 25837,70   9554,72 1092,66
8321,75 35756,64   7317,01 902252,35   8,21   0,58   71654,40
973906,74   8,86   78971,41   5,78   0,70   12,73   10,97   8,11
61,71   100,00
EMRO   Occupied Palestinian
1013500,00   6,05   30,37   63,59   61317,00   307800,00
644484,65   8,09   40,63   47,69   96,42
87650,28   160692,18   124171,23   142431,71   888,00 984,42
200,80 18362,09   9547,26 132,01 10244,99   3232,63 68,79   8117,10
6314,63 3,50   2,49   96,42   96,42   888,00
124171,23   0,00

        x   175,37
                                229,65 994,10 918,11 53,16   307,50 537,42
77,06   481,79 1109,11 38,61   249,65 826,13 2069,59 1492,18 0,00   0,00
                                                11154,61   29846,01
13224,77   3426,43 9705,96 7755,19 3910,32 13921,04   14647,76
3150,28 8347,74 11672,80   61126,33   38102,90   0,00

                                453,29   96,42   1437,71 888,00 x
31,53   6,71   61,76   0,17
0,00   0,00   0,00   0,58   x   x   x   x
x   x   x   x   x   x   x
EMRO   Pakistan   Asia   Southern Asia   73855153,00
59793793,00   36,92   24,96   38,13   22075868,38   14924530,73
22799373,27   2308567,00   2914,01 1970,04 1687,15
6571,21 5973823,91   10952010,51   8462917,21   8462917,00   52391,00
58962,21   22690,12   1534293,82   694511,05   10057,46
765958,56   229027,57   12632,66   768335,26   465483,48
1,54   2,49   263687,00   59793793,00   6571,21 6571,21 10,99   52391,00
87,62   8462917,21   53,39   23,97   350812,59   1256007,23
2080123,23   3271065,05   615122,36   3271065,05   3686943,05
4877884,87   2221942,19   4877884,87   6166,10 16259,20   6,17
2,69   9173,22 3,48   13,57   86,43   4409,94 2556,95 75926,38   771,19
75155,20   29,39   10346,48   304109,25   3120,56 307229,80
0,51   1,35   292880,85   0,49   1,29   24071,76   50658,55
40262,66   6743,34 21661,97   21131,29   12377,93   26331,16
29707,38   6818,94 21030,32   26783,82   123600,27   85388,75
145164,48   105888,89   1,17   1,24   145164,48   105888,89
20903,69   7094,56 27998,24   1199848,06   1534043,73
591197,40   377257,73   666925,77   323435,34   613327,34
782489,14   424791,00   397483,18   654233,63   399977,40
4082952,88   2722456,09   4795294,78   3376063,72   690522,45
226196,27   916718,72   1,53   1,28   307229,80   292880,85
916718,72   765958,56   2595096,94   8,33   7,94   24,86   20,77
38,08   10346,48   26743,18   27998,24   6571,21 57707,15

14046,27 17,93 46,34 11,39 24,34 0,17 37490,06 4642,81
879,35 143155,41 37180,37 5421,10 42425,99 182142,81
37278,42 4915163,29 8,22 0,58 529863,42 5445026,71
9,11 567141,84 5,64 5,38 16,84 14,07 10,42 47,66 100,00
EMRO Somalia Africa Eastern Africa 3953141,00
3447437,00 80,28 2,41 17,31 2767602,42 83083,23
596751,34 365,32 10,97 44,16 420,45
382227,37 700750,18 541488,77 541489,00 3021,00 3441,45
3455,25 267629,96 98112,62 2685,34 182585,55 35926,10
769,91 85044,40 62186,52 2,72 2,49 4918,00 3447437,00
420,45 420,45 12,20 3021,00 87,63 541488,77 54,61 29,69 22962,82
89690,43 249634,30 228523,46 67811,55 228523,46
362287,55 341176,71 180464,80 341176,71 10508,90
516,83 10,51 3,27 356,12 7,24 7,89 92,11 1426,57 66,55 2044,04
21,21 2022,83 30,40 596,53 18131,94 190,12 18322,06 0,53
0,03 43662,78 1,27 0,06 834,98 1771,62 935,29 862,09 1828,56
1036,05 344,85 1138,85 1364,43 568,14 1664,59 1787,58 5954,35 4767,10 7770,02
5667,77 1,30 1,19 7770,02 5667,77 1118,88 379,74 1498,62 41082,10
52552,37 15372,34 46252,67 54357,54 17093,52
17061,64 32690,87 20119,01 31999,34 50935,14
28562,39 205066,63 148914,12 267597,68 177048,89
38534,07 11862,28 50396,34 1,46 5,30 18322,06
43662,78 50396,34 182585,55 46209,97 5,06 12,05
13,91 50,40 18,58 596,53 1541,89 1498,62 420,45 3484,72 925,85 17,12
44,25 12,07 26,57 0,17 3997,88 523,57 77,04 9047,90 2121,98 350,50
4547,14 11286,72 2628,42 343805,13 9,97 0,58 29129,09
372934,22 10,82 31757,51 4,91 11,71 13,51 48,96 8,52
12,39 100,00
EMRO Sudan, The Republic of Africa Northern Africa
12434007,00 10349605,00 38,37 16,82 44,81 3971143,44
1740803,56 4637658,00 524,19 229,79
343,19 1097,16 997421,54 1828606,16 1413013,85 1413014,00
9068,00 10165,16 2573,17 203474,10 96367,86 1635,99
102735,16 18172,61 937,18 100738,93 78195,25 3,47
2,49 26111,00 10349605,00 1097,16 1097,16 10,60 9068,00 87,62
1413013,85 51,69 24,05 56711,28 218129,40 768793,26
554281,98 90382,58 554281,98 1043633,94 829122,66
365223,26 829122,66 10083,80 2632,98 10,08 2,66 1939,59
7,43 6,60 93,40 2522,90 137,85 4424,76 46,08 4378,67 31,76 1790,86
56884,76 598,64 57483,40 0,56 0,15 56800,47 0,55
0,14 3388,23 9782,07 12929,86 1060,66 2965,43 5768,34 1921,75 5335,72
9829,72 1236,04 2648,58 5560,66 23429,12 16272,23 24439,41
17827,10 1,04 1,10 24439,41 17827,10 3519,27 1194,42
4713,69 167603,17 291134,65 179442,45 62683,57 95339,70
83220,02 96155,79 156923,69 128234,24 75848,26
90033,68 78973,81 704315,24 488030,77 734685,92
534663,93 105794,77 35822,48 141617,26 1,37 0,99
57483,40 56800,47 141617,26 102735,16 470486,37
5,51 5,44 13,57 9,84 65,64 1790,86 4628,93 4713,69 1097,16 10080,40
2563,45 17,77 45,92 10,88 25,43 0,17 10240,96 828,38 105,58
28558,42 4035,68 533,63 11104,54 32771,97 7283,50
836406,16 8,08 0,58 81854,77 918260,94 8,87 89138,27
6,26 6,19 15,42 11,19 9,71 51,24 100,00
EMRO Syrian Arab Republic Asia Western Asia Y
5224030,00 4797872,00 10,13 23,19 66,69 486024,43
1112626,52 3199700,84 64,16 146,87

236,78 447,80 407090,72 746332,98 576711,85 576712,00
4204,00 4651,80 834,35 68228,53 36344,27 448,42 31586,21
9420,41 385,92 36642,32 26923,86 2,77 2,49 40405,00
4797872,00 447,80 447,80 9,33 4204,00 87,62 576711,85 49,43
25,18 22135,05 105866,36 302697,76 272506,18 36331,21
272506,18 430699,18 400507,60 164332,63 400507,60
8976,88 x 8,98 2,67 x 6,31 7,32 92,68 x 117,48
3586,99 38,34 3548,65 30,21 830,21 25077,52 270,94 25348,46
0,53 0,21 17687,04 0,37 0,15 1816,47 8653,45 9373,19 473,93
1805,79 2530,11 1083,88 4432,56 8181,99 349,79 1282,63 2639,88 16717,41
10756,15 10267,99 7489,89 0,61 0,70 10267,99 7489,89
1478,59 501,82 1980,41 87654,79 256377,67 128448,36 27581,68
58301,29 37261,13 54219,31 128682,66 109636,26
22649,96 43883,77 39571,05 485151,92 299171,47
297984,79 208323,70 42909,81 13957,69 56867,50
1,19 0,66 25348,46 17687,04 56867,50 31586,21
269018,39 5,89 4,11 13,20 7,33 69,47 830,21 2145,88 1980,41
447,80 4817,27 1393,38 17,23 44,55 9,30 28,92 0,17 2851,74 485,26
54,26 8463,99 2586,43 288,32 3355,09 11146,52 2407,27 402914,86
8,40 0,58 32869,41 435784,28 9,08 35276,68 5,82
4,06 13,05 7,25 8,09 61,73 100,00
EMRO Tunisia Africa Middle Africa 4115302,00
3539575,00 13,80 33,46 52,75 488461,35 1184341,80
1867125,81 64,48 156,33 138,17 358,98
326343,02 598295,54 462319,28 462319,00 3101,00 3459,98
777,60 58764,70 32391,49 334,43 20906,14 4639,86 443,17
37858,56 27751,62 2,75 2,49 39236,00 3539575,00
358,98 358,98 10,14 3101,00 87,61 462319,28 48,64 22,81 17460,09
70722,18 199169,30 180738,99 32732,02 180738,99
287351,57 268921,25 120914,28 268921,25 8118,25 3185,28
8,12 2,49 2207,78 5,63 7,81 92,19 11084,95 189,24 5328,56
54,98 5273,57 27,87 612,47 17067,88 177,94 17245,82 0,49
0,19 18096,06 0,51 0,20 764,07 4071,19 7644,37 189,30 1096,63
3034,49 296,33 1985,97 6695,11 173,37 894,27 3314,52 9680,80 6686,47 8088,75
5900,26 0,84 0,88 8088,75 5900,26 1164,78 395,32 1560,10 36818,01
118750,50 100307,65 11256,69 35163,01 41619,87
14509,22 56674,42 81628,03 11340,49 31148,64
45190,94 249297,37 155945,76 208299,20 137609,35
29995,09 9219,83 39214,91 1,11 0,59 17245,82 18096,06
39214,91 20906,14 173458,32 6,00 6,30 13,65 7,28
66,78 612,47 1583,10 1560,10 358,98 3482,98 928,43 17,58 45,45 10,31
26,66 0,17 3002,89 588,80 103,16 7905,65 3505,39 634,27 3626,08 11622,47
2531,26 271452,51 7,67 0,58 22666,22 294118,73 8,31
25197,48 5,86 6,15 13,33 7,11 8,57 58,98 100,00
EMRO West Bank & Gaza Strip Asia Western Asia Y
1275969,00 1240000,00 16,10 28,40 55,50 199640,00
352160,00 688200,00 26,35 46,49
50,93 123,76 112513,09 206274,00 159393,55 159394,00
1086,00 1209,76
8145,29 -1,00 2,49 15561,00 1240000,00 123,76 123,76 9,98
1086,00 87,58 159393,55 0,00 0,00 0,00 0,00 0,00 0,00
0,00 0,00 0,00 0,00 0,00 0,00 0,00 0,00 0,00 0,00
0,00 0,00 #DIV/0! #DIV/0! 12549,19 x x x x
x 214,56 x x
x x x x x x x x x x
x x x x 2507,95 1829,40 x x x x

x x x x x x x x x
x x x x x x x x x x
x 0,00 0,00 0,00 0,00 0,00 x x
x x x x 214,56 554,60 123,76 1764,36 871,44
12,16 31,43 7,01 49,39 0,17
0,00 0,00 0,00 0,00 0,00 0,58 x x x x
x x x x x x x
EMRO Yemen Asia Western Asia Y 6793468,00
5917244,00 27,55 10,20 62,25 1630200,72 603558,89
3683484,39 215,19 79,67 272,58 567,43
515849,19 945723,52 730786,36 730786,00 5185,00 5752,43
2336,73 169658,29 62396,79 1709,34 103278,49 12066,83
627,39 66379,80 50329,97 3,14 2,49 23486,00
5917244,00 567,43 567,43 9,59 5185,00 87,63 730786,36 53,36
25,58 30278,69 132642,09 419952,19 333647,51 54335,68
333647,51 582872,97 496568,29 217256,46 496568,29
9850,41 2313,47 9,85 2,75 1666,82 7,10 5,88 94,12 3969,08 111,01
3474,78 34,02 3440,76 31,00 1023,90 31735,73 313,78 32049,51
0,54 0,13 36823,05 0,62 0,15 2923,47 7844,38 7863,66 848,59
2370,70 3559,40 1508,53 5000,09 7164,62 949,24 2429,92 4015,59 17794,82
13614,52 13352,76 9740,05 0,75 0,72 13352,76 9740,05
1922,80 652,58 2575,38 141821,58 232026,40 114550,65 48314,28
73820,54 53488,72 73646,05 145135,86 99030,53
55308,75 78923,82 59608,01 551995,92 405894,00
414203,14 290383,10 59645,25 19455,67 79100,92
1,34 1,75 32049,51 36823,05 79100,92 103278,49
245316,32 5,50 6,32 13,57 17,72 56,89 1023,90 2646,53 2575,38
567,43 5823,58 1585,72 17,58 45,45 9,74 27,23 0,17 9634,79 541,16
64,13 22240,04 3184,43 385,52 10197,33 25552,98 5934,55
502502,84 8,49 0,58 45720,33 548223,17 9,26 51654,88
5,85 6,72 14,43 18,84 9,42 44,75 100,00
EMRO TOTAL 206806358,00
170721598,00 49500173,81 41682601,55
79546955,13 46,00 2308567,00 0,00 0,00 6534,02 5502,10 5886,47
17922,60 16293273,66 29871001,71 23082137,69
149585,00 167507,60 51318,02 3904240,69 1876905,56
27131,66 1863406,74 429788,84 24186,36 2040833,95
1455262,00 2,49 2,49 1373976,00 170721598,00 17922,60
17922,60 10,50 149585,00 87,62 23082137,69 52,84 24,21
921002,06 3624196,62 10379287,53 9294094,65 1728051,96
9294094,65 14924486,21 13839293,34 6273250,65 13839293,34
120113,02 8,74 2,66
29541,00 875718,03 0,51
7,05 916505,01 0,54 7,38
431502,82 310505,76
406484,00 296506,00 0,94 0,95 406484,00 296506,00
58533,70 19865,90 78399,60 3072923,78 5774859,57
2876020,05 1150577,33 1874786,43 1224756,55 1602269,87
3277521,51 2435487,38 1105300,38 1830843,35 1397567,35
13240072,64 9093620,03 12500187,92 8668355,85 1800027,06
580779,84 2380806,90 1,39 1,09 875718,03 916505,01
2380806,90 1853161,75 7813101,64
29365,63 77415,08 77915,88 17922,60 167006,79
42303,49 17,58 46,35 10,73 25,33 0,17 156131,32 16911,68
2354,04 447962,47 105749,97 14182,16 173827,68
558439,83 121556,41 13960849,74 8,18 0,58 1376106,39

15336956,13 8,98 1497662,79 5,71 5,98 15,52 12,08 9,77
50,94 100,00

EURO Albania Europe Southern Europe 1550679,00
1264670,00 36,42 20,15 43,43 460592,81 254831,01
549246,18 78,30 34,15 19,22 131,67
119701,59 219452,92 169577,25 169577,00 1352,17 1483,85
216,62 26809,69 20920,82 60,73 8960,94 6147,90 155,89 17848,75
14772,92 4,80 1,78 14800,00 1264670,00 131,67 131,67
10,41 1352,17 106,92 169577,25 46,32 19,73 6099,23 26679,50
141468,90 60761,73 28102,79 60761,73 174247,63
93540,46 60881,52 93540,46 13778,11 2039,16 13,78
2,59 1655,56 11,19 11,15 88,85 11702,66 77,69 1795,48 21,17
1774,31 22,84 560,22 12794,59 152,66 12947,24 1,02 0,15
12212,91 0,97 0,14 135,61 800,19 2436,73 57,72 422,17 2295,87
38,27 362,05 2235,29 32,23 307,65 2475,40 2993,22 2310,43 2106,07 1713,51
0,70 0,74 2106,07 1713,51 303,27 114,80 418,08 6992,60 23133,18
32071,13 3473,06 12873,03 30047,94 1947,67 10300,94
26732,54 2383,17 10182,12 30488,72 67178,23 43887,64
47267,48 32548,77 6806,52 2180,77 8987,29 0,71 0,71 12947,24
12212,91 8987,29 8960,94 50432,08 7,43 7,01 5,16 5,14
75,26 560,22 531,86 418,08 131,67 1597,62 373,87 35,07 33,29 8,24
23,40 0,17 115,77 28,18 11,39 500,38 231,59 71,80 147,75 755,91
150,01 93690,46 7,41 0,58 5194,65 98885,12 7,82 5344,66
13,09 12,35 9,09 9,06 5,40 51,00 100,00
EURO Armenia Asia Western Asia 1486252,00
1074582,00 24,05 24,75 51,20 258436,97 265959,05
550185,98 3,00 24,00 43,93 35,64 19,26 98,83
89844,82 164715,51 127280,17 127280,00 1148,93 1247,76
467,79 27638,07 16453,68 60,92 5372,07 2519,95 406,87 22266,00
13933,73 1,67 1,78 12645,00 1074582,00 98,83 98,83
9,20 1148,93 106,92 127280,17 46,82 20,48 4627,09 23528,72
43434,37 45918,49 18021,94 45918,49 71590,18
74074,30 46177,76 74074,30 6662,14 842,43 6,66 2,62
511,11 4,04 12,17 87,83 11767,37 251,97 5949,16 67,90 5881,26
23,34 476,02 11110,81 128,28 11239,09 1,05 0,13 10589,30
0,99 0,12 252,33 1654,41 2761,52 44,11 379,68 730,73 35,66 598,83
3952,64 19,51 260,02 1232,63 3494,62 2642,43 2018,57 1642,31 0,58 0,62
2018,57 1642,31 290,67 110,04 400,71 11845,71 48146,92 34951,44
2634,09 12141,66 9781,19 1729,12 16727,61 45061,51 1820,86
9212,37 15889,02 89679,26 49806,81 51800,70 30955,71

7459,30 2074,03 9533,33 0,89 0,50 11239,09 10589,30 9533,33
5372,07 37340,51 15,70 14,79 13,32 7,50 48,69 476,02 451,91
400,71 98,83 1298,97 272,21 36,65 34,79 7,61 20,96 0,17 102,09
22,62 6,20 397,54 345,03 93,59 126,78 773,76 149,49 74223,79
6,91 0,58 5510,27 79734,06 7,42 5659,76 14,10 13,28 11,96
6,74 7,10 46,83 100,00
EURO Azerbaijan Asia Western Asia 5103475,00
4938542,00 36,00 14,79 49,22 1777875,12 730410,36
2430750,37 63,00 239,00 302,24 97,87 85,08 485,19
441081,84 808650,04 624865,94 716757,99 5280,25 5765,44
659,99 71378,32 48798,59 227,38 19671,41 8350,48 432,61
51706,91 40448,11 3,59 1,78 42607,00 4938542,00
485,19 485,19 9,82 5280,25 106,92 624865,94 49,79 26,03 24156,66
137420,72 511513,15 262130,15 58266,39 262130,15
673090,53 423707,53 219843,77 423707,53 13629,34
5807,05 13,63 3,27 4413,05 10,36 6,24 93,76 8627,45 204,33 6348,44
60,56 6287,88 30,77 2187,68 67321,77 648,39 67970,16 1,38
0,59 30295,62 0,61 0,26 1110,79 6616,06 6824,72 254,18 2149,36
2438,51 287,30 3297,76 9072,04 174,51 1844,62 3859,64 13218,15 9914,75
6931,34 5639,36 0,52 0,57 6931,34 5639,36 998,11 377,84 1375,95 52692,30
194192,69 92948,19 14377,03 67136,99 34411,44
14334,17 93073,34 116082,44 11730,19 59595,96
52988,09 370852,22 235090,50 194467,75 133715,95
28003,36 8958,97 36962,32 0,75 0,40 67970,16 30295,62
36962,32 19671,41 268808,01 10,10 4,50 5,49 2,92
76,99 2187,68 2076,90 1375,95 485,19 6466,39 1716,62 33,83 32,12 7,50
26,55 0,17 312,25 54,73 7,42 1780,49 1113,20 150,34 369,45 2943,80
550,00 424257,53 8,59 0,58 21364,22 445621,75 9,02
21914,22 15,25 6,80 8,29 4,41 4,92 60,32 100,00
EURO Belarus Europe Eastern Europe 5006274,00
4909111,00 11,06 30,38 58,56 542947,68 1491387,92
2874775,40 95,00 1698,00 92,30 199,85 100,62 392,76
357058,39 654607,04 505832,71 580219,88 5248,78 5641,55
831,84 62161,56 37576,76 227,45 16958,53 6082,95 604,39
45203,03 31493,81 2,30 1,78 60258,00 4909111,00
392,76 392,76 8,00 5248,78 106,92 505832,71 47,82 22,68 18780,49
119056,08 284008,57 222166,83 41998,14 222166,83
421845,14 360003,40 179834,72 360003,40 8593,11 5178,03
8,59 2,81 3486,13 5,79 6,94 93,06 12274,73 335,81 8727,55
106,64 8620,91 25,67 2174,64 55827,35 690,58 56517,93 1,15
0,69 21632,81 0,44 0,27 1116,86 9400,95 13732,75 336,32
2713,45 3584,21 186,34 3755,32 26425,64 145,93 1651,43 8474,62 19339,90
17372,43 6799,33 5531,95 0,35 0,32 6799,33 5531,95 979,10 370,64
1349,74 51645,97 266552,39 183105,53 17775,78 84725,13
51206,04 8768,10 102745,81 297138,33 10088,61 56525,84
105300,05 498803,12 312274,49 175364,12 99438,46
25252,43 6662,38 31914,81 0,65 0,35 56517,93 21632,81
31914,81 16958,53 232979,32 13,40 5,13 7,57 4,02
69,89 2174,64 2064,52 1349,74 392,76 6356,32 1724,40 34,21 32,48 6,18
27,13 0,17 1083,03 261,47 56,23 4340,22 2741,34 910,86 1363,25 7385,18
1452,24 361455,64 7,36 0,58 18446,76 379902,40 7,74
19899,00 14,88 5,69 8,40 4,46 5,24 61,33 100,00
EURO Bosnia and Herzegovina Europe Southern Europe
1489352,00 802856,00 17,96 31,71 50,33 144192,94
254585,64 404077,42 24,51 34,11
14,14 72,77 66154,53 121283,31 93718,92 107501,11

858,41 931,18 353,23 24490,95 15520,80 54,80 8061,41 5514,51
298,43 16429,54 10006,29 1,56 1,78 19788,00
802856,00 72,77 72,77 9,06 858,41 106,92 93718,92 46,48
21,52 3382,01 18476,09 36105,17 40170,18 17328,97
40170,18 57963,27 62028,28 39187,07 62028,28
7219,63 1428,62 7,22 2,72 889,88 4,50 18,47 81,53 24647,01
205,50 4870,88 51,48 4819,39 23,45 355,65 8340,70 89,09 8429,80 1,05
0,21 7320,02 0,91 0,18 150,82 1300,47 2797,86 63,69 712,88 2372,58
37,47 574,24 3956,60 40,96 521,23 3820,10 3951,35 3766,12 2022,78 1645,74
0,51 0,44 2022,78 1645,74 291,28 110,26 401,54 7101,93 38168,19
36981,04 3820,48 22612,65 33247,71 1833,35 16215,58
46947,21 3162,43 18245,79 50870,04 95112,83 72062,90
48690,26 31490,42 7011,40 2109,86 9121,26 1,14 1,00 8429,80
7320,02 9121,26 8061,41 29095,80 14,54 12,63 15,74 13,91 43,18
355,65 337,64 401,54 72,77 867,27 101,21 41,01 38,93 8,39 11,67
0,17 168,20 49,47 18,26 763,73 521,95 181,50 223,76 1346,18 260,61
62288,89 7,76 0,58 5272,09 67560,97 8,42 5532,70 12,48
10,83 13,50 11,93 8,19 43,07 100,00
EURO Georgia Asia Western Asia 2312198,00
1690170,00 38,15 14,26 47,59 644799,86 241018,24
804351,90 45,00 168,00 109,62 32,30 28,15 170,06
154604,31 283441,23 219022,77 251232,00 1807,12 1977,18
529,66 31000,60 17142,97 73,94 5386,30 2049,08 455,72 25614,30
15093,89 1,43 1,78 15892,00 1690170,00 170,06 170,06
10,06 1807,12 106,92 219022,77 45,13 23,09 7675,50 41718,16
64566,90 78880,97 19806,71 78880,97 113960,56
128274,64 69200,37 128274,64 6742,55 1071,53 6,74 2,92
607,10 3,82 10,87 89,13 9402,60 299,54 7792,88 79,33 7713,55 25,75
748,71 19280,33 198,29 19478,62 1,15 0,18 10335,61
0,61 0,10 405,77 2224,22 4263,85 230,38 1403,66 3150,00 53,67 811,29
6158,03 61,88 762,30 4712,66 6735,31 5312,71 3140,34 2554,99 0,47 0,48
3140,34 2554,99 452,21 171,18 623,39 19077,46 64829,45 54962,87
11883,16 42370,89 41741,86 2647,11 22621,67 69008,12
4093,37 24180,78 57579,12 170395,86 95738,67 79447,09
46042,64 11440,38 3084,86 14525,24 0,86 0,32 19478,62
10335,61 14525,24 5386,30 78548,86 17,09 9,07 12,75
4,73 56,37 748,71 710,80 623,39 170,06 2064,59 435,01 36,26 34,43
8,24 21,07 0,17 143,49 41,36 14,02 264,44 222,50 75,60 189,52
512,14 116,48 128391,11 7,60 0,58 8395,59 136786,70 8,09
8512,06 14,24 7,56 10,62 3,94 6,22 57,42 100,00
EURO Gibraltar Europe Southern Europe 22910,00
24420,00 0,00 40,00 60,00 0,00 9768,00 14652,00
0,00 1,31 0,51 1,82 1656,12 3036,22 2346,17 2691,20
26,11 27,93
1,78 24420,00 1,82 1,82 7,46 26,11 106,92
2346,17 x x x x x x
0,00 x x x x x x x
x x x x x x x 10,82 x x
x x x x x
x x x x x x x x x 31,12
25,32 x x x x x x x x
x x x x x x x x x x
x x x x x x 0,00
0,00 0,00 0,00 x x x x x x 10,82
10,27 1,82 38,20 15,29 28,32 26,88 4,77 40,03 0,17
0,00 0,00 0,00 0,00

0,58 0,00 0,00 0,00 0,00 x x x x x
x x
EURO Kazakhstan Asia Central Asia 8951102,00
8585200,00 14,86 20,98 64,16 1275760,72 1801174,96
5508264,32 216,88 241,36 192,79 651,03
591841,83 1085043,36 838442,60 961742,98 9179,23 9830,25
2481,09 144714,12 72803,18 570,05 40962,11 12373,82
1911,04 103752,01 60429,36 1,39 1,78 169835,00
8585200,00 651,03 651,03 7,58 9179,23 106,92 838442,60 50,15
22,67 32649,37 208089,68 304389,17 384081,43 74560,93
384081,43 545128,22 624820,48 315299,97 624820,48
6349,63 10783,89 6,35 2,80 6021,52 3,55 8,58 91,42 19782,30
550,82 14942,03 158,31 14783,72 26,84 3803,08 102072,59
1093,03 103165,62 1,20 2,04 40539,98 0,47 0,80 1314,44
6690,18 7790,75 853,76 4538,74 5007,72 301,87 3018,62 13931,31 441,89
3152,13 9362,56 17663,27 14679,13 12157,04 9891,00 0,69
0,67 12157,04 9891,00 1750,61 662,70 2413,31 61942,29
196166,56 106875,05 44984,76 140293,05 72422,77
14631,18 84630,85 167335,68 27603,60 102351,17
123098,19 503152,60 326028,10 346303,10 219682,35
49867,65 14718,72 64586,36 0,75 0,48 103165,62
40539,98 64586,36 40962,11 375566,41 18,93 7,44
11,85 7,51 54,28 3803,08 3610,50 2413,31 651,03 11027,44 2962,84
34,49 32,74 5,90 26,87 0,17 982,92 123,88 18,22 3623,51 1753,29
391,36 1112,87 5507,25 1098,94 625919,42 7,29 0,58 37330,92
663250,34 7,73 38429,86 15,55 6,11 9,74 6,18 5,79
56,63 100,00
EURO Kyrgyzstan Asia Central Asia 2649315,00
2381859,00 19,32 25,36 55,33 460175,16 604039,44
1317882,58 78,23 80,94 46,13 205,30
186633,59 342161,59 264397,59 303279,59 2546,66 2751,96
404,69 36552,87 23557,19 135,25 9875,39 2849,81 269,45 26677,48
20707,38 3,47 1,78 7736,00 2381859,00 205,30 205,30 8,62
2546,66 106,92 264397,59 51,95 22,16 10664,56 56426,17
200040,89 104642,53 25033,28 104642,53 267131,61
171733,26 92124,01 171733,26 11215,26 867,61 11,22
2,82 649,71 8,40 5,61 94,39 3247,88 60,40 1787,62 17,59 1770,03
29,31 1055,12 30920,35 307,28 31227,63 1,31 0,10 14787,84
0,62 0,05 411,78 2149,34 3117,89 264,91 1267,09 893,80 102,17 1147,99
4553,77 124,15 800,12 1238,55 5430,34 4105,21 3598,20 2927,50 0,66 0,71
3598,20 2927,50 518,14 196,14 714,28 19623,98 62330,03 39174,87
13843,04 39328,29 12540,26 4947,55 31989,60 52150,55
7681,28 25941,82 16381,50 152363,73 93404,27
100957,64 66608,42 14537,90 4462,76 19000,66 0,80
0,41 31227,63 14787,84 19000,66 9875,39 96841,74
11,69 5,54 7,11 3,70 71,96 1055,12 1001,69 714,28 205,30 3039,37
777,26 34,72 32,96 6,75 25,57 0,17 327,83 37,87 5,48 1591,04
733,41 136,16 367,52 2369,83 454,40 172187,66 7,23 0,58 10982,38
183170,05 7,69 11436,79 17,05 8,07 10,37 5,39 6,24
52,87 100,00
EURO Moldova, Republic of Europe Eastern Europe
1228000,00 872394,00 20,96 21,72 57,32 182853,78
189483,98 500056,24 36,00 457,00 31,09 25,39
17,50 73,98 67252,69 123296,61 95274,65 109285,63
932,76 1006,73 218,18 21037,55 14064,49 89,71 6723,27 2602,73
128,47 14314,28 11461,76 4,02 1,78 11914,00

872394,00		73,98	73,98	8,48	932,76	106,92	95274,65		45,93
22,20	3397,87	20710,58		85363,95		38966,33		13608,02	
38966,33		109472,40		63074,78		37716,47		63074,78	
12548,50		1495,03	12,55	2,76	1165,79	9,79	5,06	94,94	13656,67
47,55	1365,24	14,47	1350,77	28,41	386,45	10978,13		117,60	11095,73
1,27	0,15	8727,32	1,00	0,12	308,89	2349,05	4391,62	110,85	1077,13
1385,61	59,14	1296,12	6961,76	47,17	791,10	2211,10	5771,66	5251,15	1667,82
1356,94	0,29	0,26	1667,82	1356,94	240,17	90,92	331,08	14475,58	
66113,65		54997,25		5949,45	32253,45		19383,98		2812,68
35098,48		79513,30		3399,46	24855,50		29407,73		
143585,87		102473,13		41491,61		26479,97		5974,79	1774,16
7748,95	0,89	0,77	11095,73		8727,32	7748,95	6723,27	28779,51	
10,14	7,97	7,08	6,14	68,67	386,45	366,88	331,08	73,98	1042,54
215,22	37,07	35,19	7,10	20,64	0,17	313,55	84,66	18,58	1355,06
907,39	242,18	404,40	2343,18	456,10	63530,88		7,28	0,58	4478,89
68009,77		7,80	4934,99	16,31	12,83	11,39	9,89	7,26	42,32
100,00									
EUR	Montenegro		Europe	Eastern Europe				243831,00	
243831,00		7,15	19,41	73,44	17433,92		47327,60		
179069,49						2,96	6,34	6,27	15,57
14157,36		25955,16		20056,26			260,70	276,28	45,95
3727,48	2447,97	8,75	1047,01	633,91	37,19	2680,47	1814,06	2,09	
4779,00	243831,00		15,57	15,57	6,39				
0,00					0,00		0,00		
							19599,64		29,63
747,27	8,64	738,63	24,93	108,01	2692,58	31,50	2724,08		
1413,79	0,58	0,03	30,06	305,65	523,82	13,27	202,77	577,56	6,45
111,21	511,05	13,72	152,53	922,13	918,88	761,63	331,16	269,43	
					1411,03	8759,17	7054,67	751,58	5937,36
7770,67	316,46	3128,13	6386,33	865,71	4682,56	11722,22		21800,92	
15029,05		0,00	0,00	0,00	0,00	0,00	0,00		
1413,79									
102,54		15,57	378,82	260,70	0,00	27,07	4,11	68,82	0,17
35,93	9,33	3,20	152,83	88,35	30,00	46,33	251,18	49,39	49,39
0,02	0,58	x	x	x	x	x	x	x	x
x	x	x							
EURO	North Macedonia		Europe	Southern Europe				963271,00	
797628,00		13,90	31,10	5,50	110870,29		248062,31		43869,54
				18,85	33,24	1,54	53,62	48748,85	
89372,89		69060,87		79216,88		852,82	906,44	149,01	13461,85
9052,82	38,73	5016,54	3191,94	110,28	8445,31	5860,88	2,27	1,78	12267,00
797628,00		53,62	53,62	6,72	852,82	106,92	69060,87		47,12
23,43	2526,51	19985,26		49741,77		39950,41		10280,73	
39950,41		72253,53		62462,17		32792,50		62462,17	
9058,55	1111,21	9,06	2,82	765,00	6,24	9,61	90,39	15379,35	
62,43	1728,53	18,21	1710,32	27,40	353,33	9679,86	103,06	9782,92	1,23
0,15	4241,45	0,53	0,07	148,05	956,03	1726,54	71,17	766,83	2290,71
42,71	431,34	1851,13	51,07	604,53	3124,84	3281,17	2788,30	1308,28	1064,42
0,40	0,38	1308,28	1064,42	188,39	71,32	259,71	6953,50	27775,88	
23904,08		3885,14	22851,37		32541,59		2045,19	12114,19	
23813,42		3303,89	18580,72		42902,93		80281,12		58282,78
32009,91		22249,14		4609,43	1490,69	6100,12	0,76	0,63	9782,92
4241,45	6100,12	5016,54	37321,14		13,54	5,87	8,44	6,94	65,20
353,33	335,44	259,71	53,62	982,17	239,78	35,97	34,15	5,46	24,41
0,17	113,31	28,14	9,95	449,50	255,99	78,84	144,77	731,77	145,51
62607,68		7,85	0,58	3525,87	66133,55		8,29	3671,37	14,79

6,41	9,22	7,59	5,55	56,43	100,00
EURO	Russian Federation		Europe	Eastern Europe
75654100,00		71933081,00		5,83	26,79	67,38	4193698,62
19270872,40		48468509,98							712,93	2582,30
1696,40	4991,62	4537839,56		8319372,53		6428606,04		7373989,29
76910,26		81901,89		17143,13		1207103,83		651498,91
6828,30	522492,42		195771,95		10314,84		684611,41
455726,96		1,99	1,78	1483498,00		71933081,00		4991,62	4991,62
6,94	76910,26		106,92	6428606,04		47,85	22,19	238839,34
1706625,18		3541138,34		3177217,47		598840,26		3177217,47
5486602,86		5122681,99		2544304,78		5122681,99		7627,37
113151,89		7,63	2,70	73029,98		4,92	6,96	93,04	20623,31
6028,62	146875,77		1788,96	145086,82		24,07	31864,96
766872,90		9455,75	776328,65		1,08	16,01	313119,36		0,44
6,46	16106,27		104051,98		129015,12		6804,09	44455,20
71362,14		3356,82	45789,10		264289,72		3008,19	28910,88
173315,57		238209,97		226933,42		102750,44		83598,09
0,43	0,37	102750,44		83598,09		14796,06		5601,07	20397,14
756155,58		2986480,61		1733115,73		355767,22		1362052,62
990581,50		160092,41		1273510,42		3025951,31		198863,36
964333,48		2102252,08		6368355,11		4306200,80		2746951,82
1586324,98		395561,06		106283,77		501844,84		0,70	0,73
776328,65		313119,36		501844,84		522492,42		3008896,72
14,15	5,71	9,15	9,52	61,47	31864,96		30251,39		20397,14
4991,62	91756,14		24648,17		34,73	32,97	5,44	26,86	0,17
13757,83		3166,31	677,37	53926,73		27911,46		9583,11	17149,94
85032,56		16962,29		5139644,29		7,15	0,58	290066,31
5429710,60		7,55	307028,61		14,30	5,77	9,24	9,62	5,65
55,42	100,00
EURO	Serbia	Europe	Southern Europe			2900000,00
2899964,00		15,61	27,43	56,96	452684,38		795460,13
1651819,49						76,96	106,59	57,81	241,36
219419,71		402269,47		310844,59		310845,00		3100,62	3341,98
1220,82	78632,99		48156,16		144,13	18093,82		11422,91
1076,68	60539,18		36733,24		1,54	1,78	52960,00
2899964,00		241,36	241,36	8,32	3100,62	106,92	310844,59		46,28
22,11	11170,95		68556,07		124912,55		141010,19		55861,79
141010,19		204639,57		220737,21		135588,81		220737,21
7056,62	3737,19	7,06	2,75	2281,19	4,31	14,81	85,19	18262,30
716,05	17583,13		201,64	17381,49		24,27	1284,63	31183,22
361,75	31544,97		1,09	0,58	28059,56		0,97	0,51	371,82
3827,81	9586,27	171,11	2333,83	8269,41	94,43	1683,97	12801,08		122,44
1658,00	13211,20		12656,46		12229,60		3938,67	3204,51	0,31
0,26	3938,67	3204,51	567,17	214,70	781,87	17397,42		108739,22
123913,11		9703,91	69925,84		112982,95		4542,42	46321,79
151844,15		8775,87	53712,95		171776,07		284731,74
221226,44		88607,99		57967,80		12759,55		3883,84	16643,39
0,57	0,62	31544,97		28059,56		16643,39		18093,82
126395,47		15,41	13,71	8,13	8,84	53,90	1284,63	1219,58	781,87
241,36	3779,69	1034,12	33,99	32,27	6,39	27,36	0,17	499,92	143,20
67,32	1603,88	999,74	414,45	665,57	2741,77	565,62	221302,82		7,63
0,58	9619,88	230922,71		7,96	10185,50		13,66	12,15	7,21
7,84	4,41	54,73	100,00
EURO	Tajikistan		Asia	Central Asia				3124212,00
2172398,00		44,72	15,79	39,49	971496,39		343021,64
857879,97						165,15	45,96	30,03	241,15

219222,80 401908,47 310565,64 310566,00 2322,71 2563,86
421,92 46079,25 29111,59 234,23 18705,13 6702,12 187,69
27374,11 22409,47 4,51 1,78 8194,00 2172398,00 241,15
241,15 11,10 2322,71 106,92 310565,64 51,24 26,45 12357,33
61439,90 284228,35 116130,19 29309,42 116130,19
358025,58 189927,42 103106,65 189927,42 16480,66
1350,43 16,48 3,40 1072,07 13,08 5,38 94,62 3771,87 53,77 1788,72
17,20 1771,52 32,95 962,33 31705,17 307,83 32013,00 1,47
0,12 17316,36 0,80 0,07 648,41 3274,29 3503,59 200,09 1497,58
1355,88 307,10 2233,63 3545,91 178,80 1262,10 1409,56 7240,19 5633,45 4243,18
3452,27 0,59 0,61 4243,18 3452,27 611,02 231,30 842,32 31088,17
95484,51 49882,34 11037,83 44915,23 20567,31
14976,32 63097,58 46501,30 10862,50 38329,60
20316,47 206008,96 149538,60 120733,58 91639,67
17385,64 6139,86 23525,49 1,08 0,86 32013,00 17316,36
23525,49 18705,13 98367,43 8,94 4,84 6,57 5,22
74,43 962,33 913,60 842,32 241,15 2635,14 518,06 36,52 34,67 9,15
19,66 0,17 213,22 32,31 3,60 1599,37 645,68 68,71 246,73 2267,95
417,44 190344,85 8,76 0,58 13597,74 203942,59 9,39
14015,17 15,70 8,49 11,54 9,17 6,87 48,23 100,00
EURO Turkey Asia Western Asia 28300520,00
28080178,00 18,11 25,32 56,57 5085320,24 7109901,07
15884956,69 864,50 952,73 555,97 2373,20
2157458,79 3955341,11 3056399,95 3056400,00 30023,10
32396,30 15155,79 791922,96 368086,27 3353,69
256478,65 85392,79 11802,10 535444,31 282693,48
1,12 1,78 720101,00 28080178,00 2373,20 2373,20 8,45 30023,10
106,92 3056399,95 51,01 21,42 121067,06 642967,22
779564,94 1203519,23 343120,76 1203519,23 1543599,21
1967553,51 1107155,04 1967553,51 5497,11 39584,77 5,50
2,72 19991,52 2,78 11,76 88,24 25644,46 7682,80
181250,36 1956,06 179294,30 23,34 12438,97 290289,61
3166,99 293456,60 1,05 7,53 188040,92 0,67 4,82 3738,64
18761,92 31753,26 1217,90 5678,06 15110,82 925,34 7635,45
36231,77 792,69 4251,11 21845,99 45017,88 32963,85
38436,66 31272,19 0,85 0,95 38436,66 31272,19
5534,88 2095,24 7630,12 177165,46 557722,21 414840,17 75282,38
188161,60 210177,43 45787,26 220993,49 430535,18
62071,05 155561,26 295320,35 1206670,84 726364,90
1030266,26 689088,93 148358,34 46168,96 194527,30
0,69 0,91 293456,60 188040,92 194527,30 256478,65
1035050,03 19,01 12,18 12,60 16,62 39,59 12438,97 11809,09
7630,12 2373,20 36575,28 9954,01 34,01 32,29 6,49 27,22 0,17
22090,44 2142,63 320,20 61276,97 17945,29 3251,33 24339,80
80306,03 17371,21 1984924,71 7,07 0,58 112436,78
2097361,49 7,47 129807,99 13,99 8,97 9,27 12,23 6,19
49,35 100,00
EURO Turkmenistan Asia Central Asia 2337132,00
2291584,00 20,68 39,83 39,49 473899,57 912737,91
904946,52 80,56 122,31 31,67 234,54
213220,85 390904,89 302062,87 302063,00 2450,14 2684,69
187,60 25007,88 17517,70 95,75 8620,08 3772,90 91,85 16387,80
13744,80 5,20 1,78 45231,00 2291584,00 234,54 234,54
10,23 2450,14 106,92 302062,87 50,62 28,78 11873,36 70503,45
375891,38 134585,64 22986,69 134585,64 458268,19
216962,45 105363,50 216962,45 19997,88 9045,24 20,00

3,59   7419,30 16,40   4,61   95,39   19737,88   46,56   1564,62 15,94
1548,68 33,26   1015,13 33765,19   347,53 34112,72   1,49   0,67
10339,52   0,45   0,20   637,21 2623,56 2747,42 383,39 1480,22 926,73
198,17 1534,89 3564,04 179,60 1040,79 1247,91 6349,09 4557,44 3174,20 2582,54
0,50   0,57   3174,20 2582,54 457,08 173,03 630,12 30259,25   77542,35
37013,35   20212,56   46646,22   13985,33   9689,08 43586,31
43249,78   10414,50   33333,42   17713,21   191659,95
117344,30   95819,54   66494,80   13798,01   4455,15 18253,17
0,80   0,38   34112,72   10339,52   18253,17   8620,08
145636,96   7,44   2,26   3,98   1,88   84,44   1015,13 963,72 630,12
234,54 3018,29 804,90 33,63   31,93   7,77   26,67   0,17   236,08 24,88
3,77   759,09 393,16 67,27   262,21 1174,67 238,52 217200,97   9,48
0,58   10550,33   227751,30   9,94   10788,85   14,98   4,54
8,01   3,78   4,74   63,95   100,00
EURO   Ukraine Europe Eastern Europe   22788270,00
16667984,00   13,82   24,96   61,22   2303515,39   4160328,81
10204139,80   410,00 3984,00   391,60 557,48 357,14 1306,23
1187478,70   2177044,28   1682261,49   1682261,00   17821,27
19127,50   4574,44 308641,57   167777,19   1270,61 93981,21
32920,44   3303,83 214660,36   134856,75   1,69   1,78
155582,00   16667984,00   1306,23 1306,23 7,84   17821,27   106,92
1682261,49   48,06   24,15   62772,46   430470,27   761276,80
799149,96   168700,04   799149,96   1254519,53   1292392,68
661942,77   1292392,68   7526,52 11709,91   7,53   2,96   7105,90
4,57   7,70   92,30   9334,18 2034,73 54626,93   717,25 53909,68
26,49   7383,59 195626,51   2602,74 198229,26   1,19   1,85   89629,63
0,54   0,84   8001,01 52365,23   86824,60   2851,64 15073,26
21441,97   1451,85 25082,30   152148,56   1014,09 9285,47 44191,82
114380,00   102280,50   30950,14   25181,13   0,27   0,25
30950,14   25181,13   4456,82 1687,14 6143,96 379750,60
1478629,58   1165183,44   147780,38   464214,99   302979,23
69375,05   680973,88   1808224,72   67514,18   306593,16
566291,81   2959763,11   1915961,78   800883,69   471703,65
115327,25   31604,14   146931,40   0,88   0,56   198229,26
89629,63   146931,40   93981,21   763621,19   15,80   7,14
11,71   7,49   57,85   7383,59 7009,71 6143,96 1306,23 19993,25   4293,72
36,93   35,06   6,53   21,48   0,17   5105,25 1393,77 356,42 17956,95
9827,16 3414,69 6617,82 28922,34   5899,67 1298292,35   7,79   0,58
84926,35   1383218,70   8,30   90826,01   14,33   6,48   10,62
6,79   6,57   55,21   100,00
EURO   Uzbekistan   Asia   Central Asia   12841390,00
13541087,00   25,71   23,02   51,27   3481413,47   3117158,23
6942515,30   65,00   237,00   591,84 417,70 242,99 1252,53
1138661,39   2087545,88   1613103,63   1613104,00   14478,02
15730,55   1850,05 217456,70   141371,37   1012,97 74883,15
22963,94   837,08 142573,55   118407,43   4,90   1,78   57707,00
13541087,00   1252,53 1252,53 9,25   14478,02   106,92 1613103,63
51,25   28,87   64197,72   417975,13   2076359,54   771487,08
146802,18   771487,08   2558532,39   1253659,93   628975,03
1253659,93   18894,59   10903,50   18,89   3,56   8848,66 15,33
3,62   96,38   4261,62 332,07 10757,53   106,51 10651,02   32,07
5998,44 192397,65   1923,97 194321,62   1,44   0,83   87809,26
0,65   0,37   4378,94 20508,42   15727,23   1376,56 7790,94 4178,88
1847,60 12271,76   20766,70   796,00 5528,65 5344,97 40690,23
29147,90   17440,67   14189,79   0,43   0,49   17440,67
14189,79   2511,46 950,72 3462,17 211697,46   598855,63

239310,65 73588,17 241187,86 67327,40 91701,92
345551,45 295635,21 49224,28 176535,19 82801,61
1227541,80 789158,45 526149,82 384178,36 75765,57
25739,95 101505,52 0,75 0,55 194321,62 87809,26
101505,52 74883,15 795140,38 7,60 3,43 3,97 2,93
82,08 5998,44 5694,69 3462,17 1252,53 17963,07 5017,41 33,39 31,70
6,97 27,93 0,17 1636,66 177,19 14,48 5748,20 2389,96 243,11 1818,68
8219,20 1666,29 1255326,22 9,27 0,58 58670,19 1313996,41
9,70 60336,48 14,79 6,68 7,72 5,70 4,59 60,51 100,00
EURO TOTAL 178952283,00
165171539,00 22837967,30 41847528,68
100091949,21 717,00 6807,00 0,00 0,00 3882,45 5607,57 3503,22 12993,24
11812037,73 21655402,51 16733720,12 176600,06
189593,30 46911,79 3137818,24 1701858,46 14487,38
1121289,45 411264,13 32424,41 2016528,79 1290594,33
1,78 1,78 2895794,00 165171539,00 12993,24 12993,24
7,87 176600,06 106,92 16713663,86 49,01 22,39 636237,51
4070628,19 7605444,43 7605444,43 1672629,05 7605444,43
12312310,12 12312310,12 6379494,74 12312310,12 7454,26
215859,91 7,45 2,85
73167,78 1894585,65 1,15
33,22 896411,26 0,54 15,72
547341,69 482650,44
243046,00 197743,00 0,44 0,41 243046,00 197743,00
34946,46 13248,78 48195,24 1857276,28 6899622,23
4430284,91 816750,03 2899628,22 2063696,60 452177,06
3102681,11 6732111,10 483858,31 2082753,69 3793099,21
14637937,26 9629873,60 6499947,27 3945381,42 935992,41
264340,56 1200332,96 0,73 0,68 1891861,57 896411,26
1211711,45 1120242,44 7207407,76
73059,77 70822,44 48175,49 12993,24 212240,25
55364,80 34,42 33,37 6,12 26,09 0,17 47237,76 7822,01
1612,13 158089,92 69026,50 19404,89 55597,15
233584,72 48004,19 12360314,31 7,48 0,58 693792,45
13054106,76 7,90 726472,28 14,49 6,87 9,28 8,58 5,57
55,21 100,00

HIGH Andorra Europe Southern Europe 39750,00 39750,00
0,40 4,70 94,90 159,00 1868,25 37722,75
0,03 0,09 0,72 0,83 756,80 1387,47 1072,14 1072,00 34,14 34,97
24,34 813,53 332,81 0,98 101,14 56,18 23,36 712,38 276,63 0,63

1,08 3155,00 39750,00 0,83 0,83 2,09 34,14 85,89 1072,14
46,03 18,65 38,32 636,86 452,18 735,42 324,51 735,42 1127,35 1410,60
999,68 1410,60 2836,11 89,48 2,84 1,70 35,89 1,14 7,65 92,35
79371,07 20,12 40,15 0,40 386,79 19,22 15,93 306,20 0,32
306,51 199,31 0,50 0,02 1,73 8,90 29,05 0,49
2,51 14,45 0,38 3,24 32,45 0,35 1,60 17,07 28,13 22,07
56,41 43,81 2,01 1,98 56,41 43,81 69298,96 2,93 69301,90
80,69 271,10 338,62 32,60 93,06 182,89 18,52 96,70 315,74 29,58
68,44 198,19 651,28 384,55 x x x x
0,25 306,51 199,31 0,00 101,14 x x x x x
x 15,93 9,19 0,83 44,16 18,21 36,07 20,81 1,89
41,24 0,17 9,17 2,59 0,60 34,59 13,51 2,51 11,96 48,93
10,11 1420,71 3,57 0,58 0,00 1420,71 3,57 10,11 21,57 14,03
0,00 7,12 0,71 x x
HIGH Antigua and Barbuda America Caribbean 39600,00
30000,00 7,00 11,00 82,00 2100,00 3300,00 24600,00
0,34 0,16 0,47 0,97 882,00 1617,00 1249,50 1250,00
25,77 26,74 3,24 388,00 285,18 1,15 101,45 45,89 2,08 286,54
239,29 5,06 2,43 1415,00 30000,00 0,97 0,97 3,23 25,77
85,89 1249,50 48,27 22,67 46,83 584,04 2995,92 1457,42 277,97 1457,42
3626,79 2088,29 908,84 2088,29 12089,30 171,06 12,09 2,10 141,31
9,99 2,36 97,64 47166,67 1,04 27,03 0,34 26,69 25,66
12,02 308,50 3,93 312,43 1,04 0,01 136,50 0,46 0,01 1,81
13,29 23,70 1,58 9,97 17,52 0,93 8,88 29,58 1,37 8,89
25,04 40,39 38,28 56,20 43,64 1,39 1,14 56,20 43,64 8,09
2,92 11,02 89,07 400,19 321,14 86,77 303,69 238,16 44,95 256,91
351,82 81,63 278,68 319,86 1066,17 886,06 1483,40 1010,22 213,61 67,68
281,30 0,94 0,34 312,43 136,50 281,30 101,45 1256,61 8,61 3,76
7,76 2,80 77,07 12,02 6,94 11,02 0,97 22,66 2,73 53,06
30,61 4,28 12,05 0,17 6,38 1,04 0,21 38,99 12,70 2,17
7,49 52,41 9,94 2098,23 6,99 0,58 162,59 2260,82 7,54 172,53
13,82 6,04 12,44 4,49 7,63 55,58 100,00
HIGH Australia Oceania Australia and New Zealand
13500080,00 12874061,00 2,56 19,06 78,38 329575,96
2453796,03 10090689,01 54,05 117,78
191,72 363,56 330505,23 605926,26 468215,75 468216,00
11057,10 11420,66 5809,90 223276,19 116372,47 311,37
54462,43 39377,54 5498,53 168813,75 76994,94 0,84
1,08 1330901,00 12874061,00 363,56 363,56 2,82 11057,10
85,89 468215,75 48,45 16,70 17613,25 184640,34
200808,24 245314,49 122972,48 245314,49 403061,84
447568,09 325226,07 447568,09 3130,81 41667,92 3,13
1,57 20759,25 1,56 15,78 84,22 103378,49 4574,30 79121,95
1637,42 77484,53 16,94 5158,62 87382,36 1846,58 89228,95
0,69 9,22 49671,83 0,39 5,13 483,52 2749,18 11269,32
106,32 587,78 4662,19 110,13 754,13 11266,98 94,98 418,80 6890,74
9237,31 7430,61 19157,73 14877,49 2,07 2,00 19157,73
14877,49 2758,71 996,79 3755,51 22823,97 81382,85
132333,08 7325,06 21132,19 57066,98 5306,34 22734,28
107923,33 8694,07 19140,87 74904,45 195797,43
116818,15 406074,34 233892,13 58474,71 15670,77
74145,48 0,58 0,42 89228,95 49671,83 74145,48
54462,43 180059,39 22,14 12,32 18,40 13,51 33,63 5158,62
2976,15 3755,51 363,56 10641,31 2142,98 48,48 27,97 3,42 20,14
0,17 3721,92 551,66 137,90 15639,19 3588,57 800,91 4319,56 19494,73
3953,17 451521,26 3,51 0,58 42856,09 494377,34 3,84

46809,26 18,05 10,05 15,00 11,02 9,47 36,42 100,00
HIGH Austria Europe Western Europe Y 4622075,00
4354863,00 3,66 25,36 70,99 159387,99 1104393,26
3091517,24 106,00 60909,00 106,00 60909,00 26,14 53,01
58,74 137,89 125353,94 229815,56 177584,75 136515,00
3740,25 3878,14 1486,16 67616,07 36045,88 151,99 18263,40
10967,38 1334,16 49352,67 25078,49 1,03 1,08
430947,00 4354863,00 106,00 106,00 2,43 3740,25 85,89
177584,75 48,00 18,19 5088,29 68051,17 77954,73 81116,49
32727,22 81116,49 151094,19 154255,96 105866,69
154255,96 3469,55 14951,93 3,47 1,68 7714,22 1,79 8,43
91,57 98957,65 1044,19 19834,31 340,90 19493,40 18,67
1744,99 32576,20 569,69 33145,89 0,76 3,28 17541,03
0,40 1,74 171,68 1525,41 7078,39 35,41 286,00 1884,64 34,21 434,54
8842,79 29,62 184,30 2953,57 5006,17 4614,79 6559,11 5093,67 1,31 1,10
6559,11 5093,67 944,51 341,28 1285,79 7978,13 44642,07 82943,80
2661,19 10578,72 25286,31 1668,31 12579,95 83584,14
2839,90 8574,37 35361,62 101936,81 65311,12 133558,13
72088,48 19232,37 4829,93 24062,30 0,55 0,42 33145,89
17541,03 24062,30 18263,40 61243,34 21,94 11,61
15,93 12,09 38,44 1744,99 1006,73 1285,79 106,00 3567,19 709,47 48,92
28,22 2,97 19,89 0,17 885,44 176,23 56,91 2582,23 933,07 272,06
1080,64 3605,98 777,98 155033,93 3,56 0,58 13908,01
168941,94 3,88 14685,99 19,62 10,38 14,24 10,81 8,69
36,25 100,00
HIGH Bahamas America Caribbean 229479,00
206281,00 2,20 14,48 83,31 4538,18 29869,49 171852,70
0,74 1,43 3,27 5,44 4948,36 9072,00 7010,18
7010,00 177,17 182,61 15,90 1476,65 972,38 3,75 256,07 66,58 12,14
1220,57 905,79 2,88 1,08 11250,00 206281,00 5,44 5,44
2,64 177,17 85,89 7010,18 50,49 25,92 274,81 4592,50 13311,67
5054,61 1002,35 5054,61 18178,99 9921,92 5869,67 9921,92 8812,73 991,43
8,81 2,36 725,98 6,45 2,04 97,96 54537,26 8,12 221,37
2,51 218,86 26,95 82,66 2227,86 25,55 2253,41 1,09 0,12 516,04
0,25 0,03 22,55 87,00 81,14 13,08 42,61 51,35 6,57 38,85
90,17 8,90 30,59 71,27 209,40 138,71 325,65 252,89 1,56 1,82
325,65 252,89 46,89 16,94 63,84 1066,65 2644,58 1121,85 673,15 1327,01
719,35 317,20 1149,03 1097,90 496,82 999,43 935,69 6325,11 3640,34 9836,50
6636,84 1416,46 444,67 1861,13 0,90 0,12 2253,41 516,04 1861,13 256,07
5035,27 12,40 2,84 10,24 1,41 73,12 82,66 47,69 63,84 5,44
166,46 30,67 49,66 28,65 3,27 18,43 0,17 28,37 4,19 0,73
168,48 50,76 8,09 32,80 221,94 42,29 9964,20 4,83 0,58 1075,73
11039,93 5,35 1118,02 20,41 4,67 16,86 2,32 10,13 45,61
100,00
HIGH Bahrain Asia Western Asia Y 981661,00
966510,00 0,94 35,25 63,81 9085,19 340694,78 616730,03
1,49 16,35 11,72 29,56 26873,81 49268,65
38071,23 38071,00 830,10 859,66 96,09 9447,13 5972,34 50,74
4085,76 1650,84 45,34 5361,37 4321,50 4,16 1,08 38475,00
966510,00 29,56 29,56 3,06 830,10 85,89 38071,23 47,99
22,93 1418,52 19036,45 80073,82 21513,40 5283,23 21513,40
100528,79 41968,37 25738,20 41968,37 10401,22
4001,87 10,40 2,12 3187,59 8,28 2,37 97,63 39808,18 29,67
689,87 7,87 682,00 22,99 387,28 8902,07 102,73 9004,80 0,93 0,36
2953,59 0,31 0,12 104,52 286,51 175,23 24,55 48,86 55,20 16,07
111,74 169,28 11,01 36,39 68,25 541,25 254,38 1393,06 1081,82 2,57

4,25   1393,06 1081,82 200,60 72,48   273,08 5126,58 8972,33 2605,08 1694,08
1906,35 858,76 794,07 3285,35 2363,21 932,80 1436,17 1063,79 18853,96
7590,72 48525,52   32281,50   6987,68 2162,86 9150,54 0,95   0,42
9004,80 2953,59 9150,54 4085,76 16773,68   8,96   2,94   9,10   4,06
74,94   387,28 223,43 273,08 29,56   810,01 169,74 47,81   27,58   3,65
20,96   0,17   537,83 83,25   3,23   871,88 231,53 15,09   622,16 1108,44
287,28 42255,64   4,37   0,58   5289,01 47544,65   4,92   5576,29
18,94   6,21   19,25   8,59   11,73   35,28   100,00
HIGH   Barbados   America Caribbean   161909,40
124956,00   2,65   18,93   78,42   3311,33 23654,17   97990,50
                        0,54   1,14   1,86   3,54   3218,43 5900,46 4559,45
4559,00 107,32 110,86 15,76   1448,05 1030,96 3,57   313,28 148,12 12,19
1134,77 882,84 3,50   2,43   4366,00 124956,00   3,54   3,54   2,83
107,32 85,89   4559,45 46,24   20,67   163,69 2218,58 7921,54 5536,10 1029,64
5536,10 10303,81   7918,36 3411,91 7918,36 8245,95 360,02 8,25   1,91
276,78 6,34   3,01   96,99   34940,30   6,68   150,69 2,08   148,62
22,25   50,07   1113,98 15,59   1129,57 0,90   0,04   487,00 0,39   0,02
6,41   51,74   107,25 5,83   38,51   94,81   2,60   32,01   142,33 4,27
30,09   152,26 169,84 167,17 229,76 178,43 1,35   1,07   229,76 178,43
33,09   11,95   45,04   309,17 1548,34 1461,80 307,81 1154,17 1271,05 127,69
921,07 1683,31 255,29 955,41 1871,11 4230,44 3444,27 5722,95 3676,33 824,11
246,31 1070,42 0,86   0,25   1129,57 487,00 1070,42 313,28 4918,10 10,96
4,73   10,39   3,04   70,88   50,07   28,89   45,04   3,54   94,71   12,21
52,87   30,50   3,74   12,89   0,17   15,29   5,16   1,45   104,28 53,38
13,25   20,93   162,07 30,38   7948,74 6,36   0,58   618,70 8567,45 6,86
649,08 13,18   5,68   12,49   3,66   7,58   57,40   100,00
HIGH   Belgium Europe Western Europe Y   5137174,00
4832024,00   0,92   20,84   78,24   44454,62   1006993,80
3780575,58   77,00   72059,00   77,00   72059,00   7,29   48,34
71,83   127,46 115870,18   212428,66   164149,42   99167,00
4150,07 4277,52 3799,02 105766,35   37007,86   126,37 13473,51
7548,26 3672,65 92292,84   29459,60   0,47   1,08   515333,00
4832024,00   77,00   77,00   1,59   4150,07 85,89   164149,42   46,89
17,11   3610,24 71001,16   37888,30   81251,70   34058,74
81251,70   112499,70   155863,09   108670,14   155863,09
2328,21 11998,04   2,33   1,54   4040,77 0,78   7,30   92,70
106649,51   3097,14 54861,92   869,14 53992,78   17,43   1936,19
33753,73   543,35 34297,08   0,71   3,66   19618,06   0,41
2,09   171,22 1604,00 6036,59 58,23   514,13 3200,25 49,06   506,94 6777,88
56,04   327,50 5168,23 5426,53 4921,57 7290,07 5661,32 1,34   1,15   7290,07
5661,32 1049,77 379,31 1429,08 8077,81 47454,77   72709,57   3817,81
17015,51   39807,72   2398,50 15282,64   67399,70   4282,57
12723,32   56776,74   113871,67   76079,18   152976,87
87514,41   22028,67   5863,47 27892,14   0,58   0,28   34297,08
19618,06   27892,14   13473,51   60582,31   30,49   17,44
24,79   11,98   15,31   1936,19 1117,04 1429,08 77,00   3915,03 784,80 49,46
28,53   1,97   20,05   0,17   1587,47 303,68 89,49   5060,60 1895,19 492,84
1920,97 7120,07 1500,81 157363,91   3,26   0,58   16121,65
173485,56   3,59   17622,47   19,77   11,31   16,08   7,77   10,16
34,92   100,00
HIGH   Brunei Darussalam   Asia   South-Eastern Asia
217006,00   221711,00   1,95   20,76   77,28   4323,36 46027,20
171338,26   0,71   2,21   3,26   6,17
5612,51 10289,61   7951,06 7951,00 190,42 196,59 42,63   3355,03 1778,24
23,32   1910,91 741,77 19,31   1444,12 1036,47 2,54   1,08   12016,00
221711,00   6,17   6,17   2,78   190,42 85,89   7951,06 50,14   21,11

309,54 4020,47 10418,57 4536,87 1232,86 4536,87 14748,57 8866,87
5562,86 8866,87 6652,16 799,32 6,65 1,95 564,65 4,70 3,43 96,57
54196,68 13,32 297,01 3,90 293,11 22,01 88,84 1954,93 26,01
1980,94 0,89 0,11 775,92 0,35 0,04 36,29 71,00 60,43 9,34
28,07 34,59 6,29 31,03 56,79 5,56 24,41 40,31 176,38 99,66
307,95 239,15 1,75 2,40 307,95 239,15 44,34 16,02 60,37 1732,91
2140,52 901,07 549,36 1006,47 578,76 313,07 883,21 745,04 392,33 932,55
665,62 5922,54 2991,37 10340,28 7178,45 1489,00 480,96 1969,96 0,89
0,86 1980,94 775,92 1969,96 1910,91 2229,14 13,43 5,26 13,36 12,96
54,99 88,84 51,25 60,37 6,17 187,48 41,21 47,39 27,34 3,29
21,98 0,17 33,64 2,96 0,30 93,77 20,36 2,25 36,70 114,88
25,16 8892,04 4,01 0,58 1138,63 10030,67 4,52 1163,80 19,75
7,74 19,64 19,05 11,60 22,22 100,00
HIGH Bulgaria Europe Eastern Europe Y 3420262,00
3233139,00 6,62 30,02 63,36 214033,80 970588,33
2048516,87 85,00 2162,00 85,00 2162,00 36,39 130,06 71,70 238,14
216493,34 396904,45 306698,90 351801,68 3456,85 3694,99
775,65 53922,05 33691,63 125,74 13673,01 7942,65 649,91
40249,04 25748,98 1,78 1,78 69105,00 3233139,00
238,14 238,00 7,37 3456,85 106,92 306698,90 45,57 22,31 10846,41
77125,50 151992,10 152150,52 40782,80 152150,52
239964,01 240122,43 128754,71 240122,43 7422,01 5128,98
7,42 2,72 3248,67 4,70 10,79 89,21 21373,97 318,30 8634,55
93,12 8541,43 26,83 1432,22 38432,89 419,00 38851,89 1,20
0,83 17735,71 0,48 0,33 754,49 4808,24 11384,56 338,16
3119,91 9242,92 173,03 1844,02 15485,99 174,48 1780,46 14000,55
15896,63 13800,83 4853,63 3769,23 0,31 0,27 4853,63 3769,23
698,92 252,54 951,46 34975,35 138706,29 147860,47 17425,39
93467,85 127630,95 8093,32 51577,99 178420,08 10687,80
57467,51 179403,77 376405,37 247101,23 114925,91
67487,36 16549,33 4521,65 21070,98 0,65 0,42 38851,89
15428,41 21070,98 13673,01 151098,13 16,19 6,43
8,78 5,70 62,90 1432,22 1359,69 951,46 238,00 4103,08 1073,17 34,91
33,14 5,80 26,16 0,17 434,32 117,18 58,90 1739,72 1229,85 738,67
571,13 3215,79 628,63 240751,06 7,45 0,58 12179,03
252930,08 7,82 12807,66 15,36 6,10 8,33 5,41 5,06
59,74 100,00
HIGH Canada America North America 20743970,00
19055731,00 1,51 19,25 79,24 287741,54 3668228,22
15099761,24 47,19 176,07 286,90 510,16
463781,85 850266,72 657024,28 657024,00 16366,34
16876,50 9730,53 374534,95 174354,55 1260,44 123193,07
63663,78 8470,09 251341,88 110690,76 0,79 1,08
1644037,00 19055731,00 510,16 510,16 2,68 16366,34 85,89
657024,28 47,23 16,61 24094,37 271773,13 239650,04
319172,96 136458,56 319172,96 535517,55 615040,46
432326,07 615040,46 2810,27 46201,88 2,81 1,55 20675,86
1,26 9,31 90,69 86275,20 6969,20 119712,84 2283,29
117429,55 16,85 7635,61 128658,34 2501,62 131159,96 0,69
11,32 71118,47 0,37 6,14 709,09 6794,12 19175,20 150,89
1134,46 6320,20 170,71 2171,42 19753,52 135,93 852,31 9878,63 17287,03
13207,75 29437,41 22860,48 1,70 1,73 29437,41
22860,48 4238,99 1531,65 5770,64 33496,26 197936,14
234594,93 12341,74 46658,00 94843,10 8441,37 63478,39
197136,18 12561,42 40588,34 120585,63 400244,81
230976,80 681561,18 399783,52 98144,81 26785,50

124930,30 0,66 0,65 131159,96 71118,47 124930,30
123193,07 164638,65 24,49 13,28 23,33 23,00 15,89 7635,61
4405,20 5770,64 510,16 15511,05 2960,09 49,23 28,40 3,29 19,08
0,17 4446,62 443,36 116,30 15848,90 3234,52 588,05 4928,74 19279,44
4018,56 619059,02 3,25 0,58 72209,72 691268,74 3,63
76228,28 18,97 10,29 18,07 17,82 11,03 23,82 100,00
HIGH Chile America South America 9514561,00
8501401,00 8,98 22,25 68,78 763425,81 1891561,72
5847263,61 125,20 90,79 111,10 327,09
297358,91 545158,01 421258,46 421258,00 7301,57 7628,67
1824,08 123174,60 73167,20 586,34 49614,85 22161,18
1237,74 73559,75 51006,02 2,26 2,43 252940,00
8501401,00 327,09 327,09 3,85 7301,57 85,89 421258,46 46,82
18,22 15315,26 133046,95 313253,05 335555,85 63368,82
335555,85 461615,26 483918,05 211731,03 483918,05
5429,87 13734,32 5,43 1,75 9320,14 3,68 6,00 94,00 29752,74
644,69 13589,14 186,13 13403,01 20,79 3406,50 70820,63
983,50 71804,13 0,84 2,14 33053,45 0,39 0,98 371,74
2277,04 4127,78 209,79 1250,85 3548,98 79,37 827,68 4302,78 170,63 848,52
4512,16 6668,34 4864,51 13501,95 10485,33 2,02 2,16 13501,95
10485,33 1944,28 702,52 2646,80 17675,11 67260,63 54797,80
12382,75 39806,54 47930,12 3747,47 23933,87 47404,69
11882,38 30379,44 55801,11 171367,67 104345,10
346982,55 224913,37 49965,49 15069,20 65034,68
0,76 0,58 71804,13 33053,45 65034,68 49614,85
264410,94 15,55 7,16 14,09 10,75 52,45 3406,50 1965,31 2646,80
327,09 6947,17 1248,27 49,03 28,29 4,71 17,97 0,17 3214,06 364,48
82,87 15979,57 4538,92 787,02 3606,16 20780,83 4048,24
487966,29 5,74 0,58 37590,05 525556,34 6,18 41638,29
13,66 6,29 12,37 9,44 7,92 50,31 100,00
HIGH Croatia Europe Southern Europe Y 1960869,00
1679460,00 6,19 27,67 66,14 103958,57 464706,58
1110794,84 43,00 10373,00 43,00 10373,00 17,05 22,31
21,11 60,46 54963,84 100767,04 77865,44 55379,00
1442,43 1502,89 1067,43 41283,15 18957,55 43,66 5815,10 3760,42
1023,77 35468,05 15197,13 0,75 1,78 55967,00
1679460,00 43,00 43,00 2,56 1442,43 85,89 77865,44 47,06
19,80 2023,60 28560,54 25115,40 54479,56 18900,67
18900,67 55699,54 85063,69 49484,80 49484,80
3316,51 1856,15 3,32 1,82 836,96 1,50 10,28 89,72 33324,40
873,15 18154,83 266,24 17888,59 20,49 672,96 13787,16
205,20 13992,36 0,83 0,47 10859,77 0,48 0,27 159,10
1426,66 4252,65 49,65 670,81 2438,75 24,52 465,93 6868,59 26,98 337,02
4033,54 4536,68 4488,48 2782,64 2160,94 0,61 0,48 2782,64 2160,94 400,70
144,78 545,48 7421,00 41636,88 53043,04 2954,26 20809,58
33580,93 1234,49 13417,41 72967,23 2365,43 12291,50
50334,07 101696,37 70409,26 62376,87 33897,86
8982,27 2271,16 11253,43 0,67 0,35 13992,36 8102,32 11253,43
5815,10 10321,60 25,12 14,55 20,20 10,44 29,69 672,96 388,25
545,48 43,00 1328,20 223,99 50,67 29,23 3,24 16,86 0,17 280,55
98,87 45,97 996,28 764,18 369,56 394,75 1883,65 378,21 49863,01
2,97 0,58 6504,48 56367,49 3,36 6882,69 24,82 14,37 19,96
10,32 12,21 18,31 100,00
HIGH Cyprus Asia Western Asia Y 636733,00
416478,00 2,41 18,44 79,16 10037,12 76798,54
329683,98 10,00 2158,00 10,00 2158,00 1,65 3,69 6,26 11,60

10542,19 19327,36 14934,77 12879,00 357,70 369,30
199,25 9117,81 5043,69 22,00 2012,86 948,04 177,26 7104,96 4095,65 1,36
1,08 23804,00 416478,00 10,00 10,00 2,40 357,70 85,89
14934,77 48,41 16,98 484,12 6072,72 8695,97 6987,11 4526,67 6987,11
15252,81 13543,95 11083,51 13543,95 3662,33 871,78
3,66 1,57 497,02 2,09 6,00 94,00 57155,48 138,68 2502,41
40,96 2461,46 17,75 166,88 2962,03 49,29 3011,32 0,72 0,17 3058,94
0,73 0,17 55,48 274,19 614,48 7,15 45,43 258,98 9,69 68,41
573,48 5,90 29,90 397,78 673,41 437,66 903,58 701,70 1,34 1,60
903,58 701,70 130,12 47,01 177,13 2642,03 8053,97 8354,21 493,84 1513,93
3559,56 469,85 2020,41 6398,31 510,22 1142,81 4754,66 16675,03 7860,94
22374,50 12603,47 3221,93 844,43 4066,36 0,98 0,48 3011,32
3058,94 4066,36 2012,86 1394,48 19,74 20,05 26,66 13,20 20,35 166,88
96,28 177,13 10,00 286,85 13,69 58,18 33,56 3,49 4,77 0,17
103,97 20,54 6,56 427,75 111,96 24,75 126,70 547,96 111,99 13655,94
3,28 0,58 2350,36 16006,30 3,84 2462,35 18,81 19,11 25,40
12,58 15,38 8,71 100,00
HIGH Czech Republic Europe Eastern Europe Y 5441332,00
5303100,00 2,66 37,25 60,09 141062,46 1975404,75
3186632,79 95,00 42321,00 95,00 42321,00 23,13 94,82
60,55 178,50 162272,45 297499,49 229885,97 122348,00
4554,66 4733,16 1652,92 112565,82 71412,81 319,41 21896,00
31117,04 1333,51 90669,82 40295,77 0,80 1,78
245349,00 5303100,00 95,00 95,00 1,79 4554,66 85,89
229885,97 -28,87 37,78 -2742,59 172054,11 146886,49
315139,51 49550,84 49550,84 316198,01 484451,03
218862,35 218862,35 5962,51 14628,97 5,96 3,19 6795,73
2,77 2,06 97,94 46265,20 837,09 17679,15 245,39 17433,76
20,83 2124,94 44255,43 622,92 44878,35 0,85 2,08 27213,36
0,49 1,19 341,61 3709,16 11776,51 94,94 948,27 3719,11 64,16
1149,21 15918,92 47,51 473,17 6246,16 10259,19 9122,42 7721,70
5996,51 0,75 0,66 7721,70 5996,51 1111,92 401,77 1513,69 15970,74
105611,87 151330,15 6341,74 31429,68 52716,22 3211,98
32916,38 167499,41 5504,53 20130,76 77784,93
227369,48 143525,10 171132,30 94344,51 24643,05
6321,08 30964,13 0,58 0,41 44878,35 25741,45 30964,13
21896,00 95382,41 14,19 8,14 9,79 6,92 60,95 2124,94
1225,94 1513,69 95,00 4361,91 916,02 48,72 28,11 2,18 21,00 0,17
574,89 177,08 79,44 3434,16 2301,39 1109,70 778,45 6105,45 1142,73
220005,08 4,15 0,58 17897,27 237902,35 4,49 19040,00
18,86 10,82 13,02 9,20 8,00 40,09 100,00
HIGH Denmark Europe Northern Europe Y 3023904,00
2877657,00 2,22 18,55 79,23 63883,99 533805,37
2279967,64 39,00 50179,00 39,00 50179,00 10,48 25,62
43,32 79,42 72199,11 132365,03 102282,07 50227,00
2471,52 2550,94 1852,55 60790,73 26882,13 64,32 8825,04 5818,71
1788,23 51965,69 21063,42 0,68 1,08 356085,00
2877657,00 39,00 39,00 1,36 2471,52 85,89 102282,07 46,74
17,28 1822,83 42710,28 32639,99 49637,90 24591,49
49637,90 77173,11 94171,01 69124,60 94171,01
2681,80 9549,50 2,68 1,55 4038,92 1,13 6,93 93,07 123741,29
1410,82 25401,47 425,27 24976,21 17,70 1153,07 20413,23
347,58 20760,81 0,72 2,57 15277,92 0,53 1,89 67,00
726,24 3030,44 30,39 304,43 1731,42 13,03 220,14 3112,90 24,39 181,61
2267,57 2715,35 2232,66 4291,17 3332,43 1,58 1,49 4291,17 3332,43 617,93
223,27 841,20 3101,32 21186,14 37407,74 1972,25 9994,51 23035,74

635,42 6457,46 30567,08 1947,43 6793,24 26140,12 56402,04
34735,96 89134,42 51846,25 12835,36 3473,70 16309,06
0,57 0,31 20760,81 15277,92 16309,06 8825,04 32998,19
26,90 19,80 21,13 11,44 20,73 1153,07 665,24 841,20 39,00 2334,56
477,25 49,39 28,50 1,67 20,44 0,17 434,77 113,49 39,75 2253,42
1059,76 319,33 561,51 3419,62 660,87 94831,88 3,30 0,58 9426,63
104258,52 3,62 10087,50 19,91 14,65 15,64 8,46 9,68
31,65 100,00
HIGH Estonia Europe Northern Europe Y 703864,00
671337,00 3,17 28,70 68,13 21281,38 192673,72
457381,90 15,00 6180,00 15,00 6180,00 3,49 9,25 8,69 21,43
19480,67 35714,57 27597,62 19318,00 576,59 598,02
125,50 8103,15 5152,31 21,07 1999,89 1018,43 104,43 6103,26 4133,88 2,10
1,78 30650,00 671337,00 15,00 15,00 2,23 576,59 85,89
27597,62 46,58 18,86 698,72 10873,14 23548,64 20055,72
4858,92 4858,92 35120,50 31627,58 16430,78 16430,78
5231,43 1603,43 5,23 1,72 1075,12 3,51 4,05 95,95 45655,16
71,79 1446,74 18,90 1427,84 19,89 269,00 5350,25 70,82 5421,07 0,81
0,25 2818,51 0,48 0,15 41,36 453,42 1287,49 12,89 123,61 309,64
6,12 142,54 2503,26 5,19 72,63 669,09 1163,66 1283,94 998,84 775,68
0,86 0,60 998,84 775,68 143,83 51,97 195,80 1928,44 12942,70
16050,73 835,14 4020,56 4322,84 311,39 4069,50 25534,70 614,55
2894,78 8372,58 26518,02 19192,64 22762,10 11595,04
3277,74 776,87 4054,61 0,60 0,30 5421,07 3233,27 4054,61 1999,89 1721,94
15,44 9,21 11,54 5,69 58,12 269,00 155,20 195,80 15,00 550,98
111,78 48,82 28,17 2,72 20,29 0,17 144,58 34,33 12,48 454,51
299,63 164,08 183,07 808,84 164,66 16595,44 2,47 0,58 2343,56
18939,00 2,82 2508,22 28,62 17,07 21,41 10,56 13,24 9,09
100,00
HIGH Faroe Islands Europe Northern Europe 34710,00
27540,00 10,70 18,90 70,40 2946,78 5205,06 19388,16
0,48 0,25 0,37 1,10 1001,35 1835,82 1418,59 1419,00
23,65 24,75
2,43 3126,00 27540,00 1,10 1,10 4,00 23,65 85,89
1418,59 x x x x x x
x x x x x x x x
x x x x x x x 11,04 x x
x x x x x
x x x x x x x x x 49,26
38,25 x x x x x x x x
x x x x x x x x x x
x x x x x x 0,00
0,00 0,00 0,00 x x x x x x 11,04
6,37 1,10 31,12 12,62 35,46 20,46 3,54 40,54 0,17
0,00 0,00 0,00 0,00
0,58 x x x x x x x x x
x x
HIGH Finland Europe Northern Europe Y 2748960,00
2565577,00 3,78 21,64 74,58 96978,81 555190,86
1913407,33 26,00 37632,00 15,90 26,65 36,35
78,91 71734,93 131514,04 101624,49 37632,00 2203,49
2282,40 1180,85 41792,35 20357,55 52,01 6738,66 4306,02 1128,84
35053,69 16051,53 0,84 1,08 269751,00 2565577,00
26,00 26,00 1,01 2203,49 85,89 101624,49 46,77 16,83 1216,12
37092,21 33485,24 42197,25 18204,19 42197,25
71793,57 80505,58 56512,52 80505,58 2798,34 7548,55

2,80	1,49	3520,72	1,31	4,69	95,31	105142,43		933,96	16267,76
307,34	15960,42		17,09	1028,02	17567,86		338,29	17906,16
0,70	1,88	11083,15		0,43	1,17	95,68	1439,99	6030,23	39,60
336,44	1863,18	14,12	312,51	6919,80	24,82	178,81	3107,57	4542,84	3872,71
3901,00	3029,44	0,86	0,78	3901,00	3029,44	561,74	202,97	764,72	4451,22
40668,59		73308,43		2478,11	11017,50		24525,55		703,62
8886,85	64633,53		2131,25	7305,01	34962,67		91226,75		52225,47
78337,71		40853,44		11280,63		2737,18	14017,81		0,55
0,26	17906,16		11083,15		14017,81		6738,66	30759,81
24,94	15,44	19,53	9,39	30,71	1028,02	593,10	764,72	26,00	2057,87
410,75	49,96	28,82	1,26	19,96	0,17	739,90	148,30	53,18	2685,76
1011,06	313,96	905,93	3801,48	781,43	81287,01		3,17	0,58	8102,29
89389,31		3,48	8883,72	20,03	12,40	15,68	7,54	9,94	34,41
100,00
HIGH	France	Europe	Western	Europe	Y		30385859,00
27176029,00		2,53	20,43	77,04	687553,53		5552062,72
20936412,74		803,00	778820,00		803,00	778820,00		112,76	266,50
397,79	777,05	706408,76		1295082,72		1000745,74		1034167,00
23340,59		24117,64		19297,13		680472,07		278160,49
1660,81	177897,81		99718,38		17636,32		502574,27
178442,11		0,55	1,08	2630318,00		27176029,00		803,00	803,00
2,95	23340,59		85,89	1000745,74		47,07	18,38	37799,59
428969,08		284370,84		511326,88		226655,73		511326,88
751139,51		978095,56		693424,41		978095,56		2763,98	72701,42
2,76	1,72	27523,73		1,05	11,45	88,55	96788,17		15812,35
303436,45		4971,49	298464,96		18,88	10889,40		205542,11
3423,69	208965,79		0,77	20,23	129802,81		0,48	12,56	989,01
6427,48	27236,67		408,37	2468,40	15337,81		201,28	1620,30	31850,14
287,10	1339,94	24958,47		24484,75		22384,83		43120,05
33486,13		1,76	1,50	43120,05		33486,13		6209,29	2243,57
8452,86	46425,63		192347,81		310239,62		25008,28		84403,62
186761,27		9907,94	50671,30		287482,37		22034,03		56434,95
260305,50		513852,30		321644,17		904944,24		481157,08
130311,97		32237,52		162549,49		0,60	0,65	208965,79
129802,81		162549,49		177897,81		298879,65		27,82	17,28
21,64	23,68	9,58	10889,40		6282,40	8452,86	803,00	21973,14
3998,34	49,56	28,59	3,65	18,20	0,17	6786,54	1061,42	373,49	27731,51
9816,23	2938,49	7972,45	38527,24		7718,95	985814,50		3,63	0,58
93953,61		1079768,11		3,97	101672,56		19,35	12,02	15,05
16,48	9,42	27,68	100,00
HIGH	Germany	Europe	Western	Europe	Y		41975210,00
42395665,00		1,21	27,18	71,61	512987,55		11523141,75
30359535,71		416,00	867533,00		416,00	867533,00		84,13	553,11
576,83	1214,07	1103701,76		2023453,23		1563577,50		867533,00
36412,23		37626,30		22584,55		778706,62		368836,98
942,08	120378,63		76996,03		21642,47		658328,00
291840,95		0,80	1,08	3846414,00		42395665,00		416,00	416,00
0,98	36412,23		85,89	1563577,50		46,05	16,93	19156,74
616593,84		525005,61		699671,80		325840,58		699671,80
1160756,19		1335422,38		961591,16		1335422,38		2737,91
105311,45		2,74	1,50	47631,97		1,24	4,58	95,42	90726,59
17440,40		305026,24		5379,28	299646,95		17,18	16987,89
291872,21		5239,71	297111,91		0,70	26,96	205227,97		0,48
18,62	1628,38	17276,88		74169,56		460,01	4232,17	25382,23
363,83	4967,10	85916,91		359,49	2457,73	38754,15		56781,38
49705,16		59566,29		46257,94		1,05	0,93	59566,29

46257,94		8577,55	3099,28	11676,83		75979,55		505485,28
887797,46		30215,68		145803,62		338614,04		17663,76
146318,06		844476,60		29160,72		101508,00		457159,04
1166287,96		728529,08		1223490,11		678003,14		176182,58
45426,21		221608,79		0,52	0,28	297111,91		205227,97
221608,79		120378,63		491095,08		25,60	17,68	19,09	10,37
27,26	16987,89		9800,79	11676,83		416,00	34952,20		7747,52
48,60	28,04	1,19	22,17	0,17	8511,63	1812,78	623,50	41004,91
19612,00		6392,50	10532,24		62747,74		12164,48
1347586,85		3,18	0,58	128089,88		1475676,73		3,48
140254,36		20,13	13,91	15,02	8,16	9,50	33,28	100,00
HIGH	Greece	Europe	Southern Europe	Y		4822612,00
3911030,00		11,60	15,34	73,06	453679,48		599952,00
2857398,52				35,00	5127,00	74,40	28,80	54,29	157,49
143174,28		262486,17		202830,22		45076,00		3359,05	3516,55
1951,51	76621,04		41008,67		129,40	11739,27		5855,19	1822,12
64881,78		35153,48		1,18	1,08	189410,00		3911030,00
35,00	35,00	0,89	3359,05	85,89	202830,22		45,47	16,32	1591,57
54803,81		66388,84		60749,72		36737,24		60749,72
122784,22		117145,10		93132,62		117145,10		3139,43	5946,40
3,14	1,44	3215,19	1,70	2,59	97,41	48429,70		1328,24	23905,24
365,50	23539,74		17,72	1567,14	27773,72		431,24	28204,97
0,72	1,37	24737,78		0,63	1,20	608,86	2906,66	9666,30	124,08
823,58	7140,14	119,08	826,68	12014,86		79,95	471,29	11775,36
10065,32		9427,08	6843,69	5314,66	0,68	0,56	6843,69	5314,66	985,49
356,08	1341,57	28187,96		86249,59		111770,19		6913,49	25896,65
81702,96		5631,88	24171,27		120226,18		5447,66	16609,99
124468,40		211738,73		133425,67		143966,94		75220,77
20731,24		5039,79	25771,03		0,66	0,30	28204,97		24737,78
25771,03		11739,27		26692,06		22,97	20,15	20,99	9,56
26,33	1567,14	904,13	1341,57	35,00	2956,61	450,34	53,00	30,58	1,18
15,23	0,17	1478,14	525,33	207,42	5939,89	2598,20	867,77	2072,61	8827,34
1809,39	118954,50		3,04	0,58	14895,66		133850,15		3,42
16705,05		21,07	18,48	19,25	8,77	12,48	19,94	100,00
HIGH	Greenland		America	North America				26840,00
26840,00		13,80	19,20	67,00	3703,92	5153,28	17982,80
		0,61	0,25	0,34	1,20	1087,70	1994,12	1540,91	1541,00
23,05	24,25	17,22	682,97	272,38	2,06	146,97	51,18	15,16	536,01
221,20	0,70	1,08	3052,00	26840,00		1,20	1,20	4,46	23,05
85,89	1540,91	46,44	20,77	55,56	478,71	366,06	546,50	250,89	546,50
900,33	1080,77	785,16	1080,77	3354,45	102,38	3,35	1,99	41,63	1,36
9,47	90,53	113710,88		12,59	265,92	2,92	262,99	20,89	10,75
224,65	2,49	227,15			159,63	0,59	0,02	1,76	19,22
17,24	1,08	9,96	14,06	0,46	4,78	12,61	0,89	4,32	12,37
42,45	18,78	38,09	29,58	x	x	x	x	x	x
79,44	574,76	249,72	61,61	336,67	224,19	21,81	141,21	152,19	54,94
160,83	172,51	1210,45	487,01	x	x	x	x
0,55	227,15	159,63	0,00	146,97	x	x	x	x	x
x	10,75	6,20		1,20	30,45	12,30	35,32	20,37	3,93
40,38	0,17	24,93	2,30	0,23	73,95	13,29	0,79	27,30	87,51
19,06	1099,83	4,10	0,58	0,00	1099,83	4,10	19,06	20,65	14,51
0,00	13,36	1,73	x	x
HIGH	Guernsey		Europe	Northern Europe				31470,00
31470,00				0,00	0,00	0,00
	0,00	0,00	0,00	0,00	0,00	0,00	0,00	0,00	27,03
27,03

1,08 31470,00 0,00 0,00 0,00 27,03 85,89 0,00
x 0,00 x x x x x x
x x x x x x x x x
x x x x 12,61 x
x x x x x x x x
x x x x x x 44,66 34,68 x x
x x x x x x x x x
x x x x x x x x x x
x x x x 0,00 0,00 0,00 0,00
x x x x x x 12,61 7,28 0,00
34,30 14,42 36,76 21,21 0,00 42,03 0,17
0,00 0,00 0,00 0,00 0,00 0,58 0,00 0,00
0,00 0,00 x x x x x x x
HIGH Hungary Europe Eastern Europe Y 4295107,00
4512124,00 4,72 32,09 63,19 212972,25 1447940,59
2851211,16 84,00 23802,00 84,00 23802,00 34,93 69,50
54,17 158,60 144183,28 264336,02 204259,65 108182,00
3875,31 4033,92 1619,49 84698,42 46308,80 118,94 14313,67
8721,71 1500,55 70384,74 37587,08 1,15 1,78 155013,00
4512124,00 84,00 84,00 1,86 3875,31 85,89 204259,65 47,02
21,86 3949,36 84703,06 103231,96 156748,14 43746,85
43746,85 191884,38 245400,56 132399,27 132399,27
4252,64 6592,14 4,25 1,96 3546,51 2,29 5,27 94,73 34354,77
909,11 21714,45 251,06 21463,39 23,61 1808,00 42685,54
499,30 43184,84 0,96 1,48 26010,22 0,47 0,73 535,87
4600,35 10720,02 170,15 1530,57 3677,68 135,41 1946,52 17992,39
94,97 862,16 6754,22 11636,16 11287,92 6095,11 4733,34 0,52
0,42 6095,11 4733,34 877,70 317,13 1194,83 24723,90 132005,06
137664,64 9706,79 49492,34 53918,14 6405,80 54487,45
194245,12 7780,16 32851,92 88134,95 279789,01
195652,02 146555,67 82042,27 21104,02 5496,83 26600,85
0,59 0,32 43184,84 21221,19 26600,85 14313,67
27078,73 22,51 11,06 13,86 7,46 45,11 1808,00 1043,09 1194,83
84,00 3807,57 872,48 47,48 27,40 2,21 22,91 0,17 514,85 186,72
73,48 1746,23 1217,97 534,60 726,06 3142,40 642,16 133041,44 2,95
0,58 15375,29 148416,73 3,29 16017,45 29,10 14,30
17,92 9,64 10,79 18,25 100,00
HIGH Iceland Europe Northern Europe To be included 199778,90
201016,00 4,04 17,47 78,49 8121,05 35117,50 157777,46
1,33 1,69 3,00 6,02 5468,42 10025,44
7746,93 7747,00 172,65 178,66 55,00 2890,48 1791,92 4,60 610,29 392,74
50,40 2280,19 1399,18 1,59 1,08 21718,00 201016,00 6,02
6,02 2,99 172,65 85,89 7746,93 47,31 17,48 284,58 3018,10 5306,95
3772,08 1912,92 3772,08 8609,64 7074,77 5215,61 7074,77 4283,06 930,20 4,28
1,64 573,37 2,64 9,27 90,73 108041,15 40,32 753,32 14,44
738,77 18,32 80,55 1475,83 28,85 1504,68 0,75 0,16 1037,23 0,52
0,11 7,07 54,98 209,57 1,32 8,88 61,41 1,09 8,46 155,13
0,92 4,10 71,76 162,57 90,19 283,50 220,16 1,74 2,44 283,50
220,16 40,82 14,75 55,58 334,89 1604,96 2397,02 96,80 322,15 784,42
55,27 259,83 1374,67 90,29 201,24 788,81 3419,28 1327,80 5962,72 3241,19
858,63 217,16 1075,79 0,54 0,30 1504,68 1037,23 1075,79 610,29 2846,78
17,48 12,05 12,50 7,09 50,89 80,55 46,47 55,58 6,02 169,56
36,52 47,50 27,41 3,55 21,54 0,17 18,19 3,32 0,97 64,94
20,67 5,03 21,83 87,29 18,11 7092,88 3,53 0,58 621,81 7714,69
3,84 639,92 19,50 13,44 13,94 7,91 8,29 36,90 100,00
HIGH Ireland Europe Northern Europe Y 2234085,00

2322488,00		4,43	18,77	76,80	102886,22		435931,00		
1783670,78		41,00	13252,00		41,00	13252,00		16,87	20,92
33,89	71,69	65170,70		119479,62		92325,16		52803,00	
1994,71	2066,40	740,60	36473,05		21914,48		69,41	10022,65	
6767,18	671,19	26450,41		15147,30		1,34	1,08	425889,00	
2322488,00		41,00	41,00	1,77	1994,71	85,89	92325,16		46,90
16,84	1923,00	33591,46		49013,29		40262,57		19144,67	
40262,57		84527,75		75777,03		54659,13		75777,03	
3639,53	15500,38		3,64	1,53	8987,87	2,11	7,00	93,00	
183376,19		500,89	8928,80	154,05	8774,75	17,52	930,62	16302,86	
286,21	16589,07		0,71	3,04	11247,06		0,48	2,06	99,07
751,00	2456,23	23,90	129,62	861,78	20,02	210,74	2272,64	19,31	96,21
1231,65	2109,59	1514,37	3170,35	2462,03	1,50	1,63	3170,35	2462,03	456,53
164,96	621,49	4577,45	21882,04		30878,82		1621,02	4528,66	11156,62
972,10	6176,67	23140,69		1681,13	3860,83	13892,26		46620,98	
25035,04		70063,18		40701,49		10089,10		2727,00	12816,10
0,55	0,43	16589,07		11247,06		12816,10		10022,65	
25102,16		19,63	13,31	15,16	11,86	40,05	930,62	536,90	621,49
41,00	1951,12	442,60	47,70	27,52	2,10	22,68	0,17	937,44	234,68
64,10	2061,56	545,95	125,28	1193,49	2649,28	637,90	76414,93		3,29
0,58	7407,70	83822,63		3,61	8045,60	19,79	13,42	15,29	11,96
9,60	29,95	100,00							
HIGH	Isle of Man		Europe	Northern Europe				41790,00	
41790,00		2,00	13,00	85,00	835,80	5432,70	35521,50		
		0,14	0,26	0,67	1,07	975,23	1787,92	1381,57	1382,00
35,89	36,96								
	1,08	7492,00	41790,00		1,07	1,07	2,57	35,89	85,89
1381,57	x	x			x	x	x		x
x	x		x	x		x	x	x	x
x	x	x	x	x	x	x	16,75	x	x
					x	x	x	x	x
x	x	x	x	x	x	x	x	x	59,30
46,05	x	x	x	x	x	x		x	x
x	x	x	x	x	x	x	x	x	x
x	x	x	x	x	x				0,00
0,00	0,00	0,00	x	x	x	x	x	x	16,75
9,66		1,07	46,63	19,15	35,91	20,72	2,30	41,07	0,17
					0,00	0,00	0,00	0,00	0,00
0,58	0,00	0,00	0,00	0,00	x	x	x	x	x
x	x								
HIGH	Israel	Asia	Western Asia				3846000,00		
3966920,00		0,92	17,23	81,86	36495,66		683500,32		
3247320,71		70,00	65640,00				5,99	32,81	61,70
100,49	91356,73		167487,33		129422,03		129422,00		3407,06
3507,55	656,81	44672,70		29470,00		101,42	13125,60		8183,15
555,39	31547,10		21286,86		2,07	1,08	401954,00		
3966920,00		100,49	100,49	2,53	3407,06	85,89	129422,03		48,73
18,47	4897,42	62941,79		138693,47		76060,26		29395,42	
76060,26		206532,67		143899,47		97234,63		143899,47	
5206,37	20927,23		5,21	1,71	14053,32		3,50	6,30	93,70
101326,47		424,88	8255,43	127,89	8127,53	19,13	1589,54	30406,30	
478,46	30884,76		0,78	3,13	16414,72		0,41	1,66	105,25
687,41	2538,37	37,26	266,07	1201,32	16,79	189,72	2628,40	18,72	140,72
1683,92	2342,56	1803,39	5457,79	4238,41	2,33	2,35	5457,79	4238,41	785,92
283,97	1069,90	5068,18	20479,47		29996,18		2661,71	8872,23	15795,02
885,57	5675,61	26338,19		2167,39	5737,00	19838,59		52345,33	

29857,83 121956,48 70173,37 17561,73 4701,62 22263,35
0,56 0,33 30884,76 16414,72 22263,35 13125,60
61211,04 14,95 7,95 10,78 6,36 59,96 1589,54 917,05 1069,90
100,49 3354,70 747,62 47,38 27,34 3,00 22,29 0,17 1924,27 349,13
110,25 5211,43 1509,04 444,32 2310,16 6868,58 1523,67 145423,14 3,67
0,58 12868,22 158291,35 3,99 14391,89 19,51 10,37
14,06 8,29 9,09 38,67 100,00
HIGH Italy Europe Southern Europe Y 25529690,00
23359867,00 3,89 25,87 70,24 908698,83 6043197,59
16407970,58 491,00 289283,00 491,00 289283,00 149,03 290,07
311,75 750,85 682592,30 1251419,22 967005,76 20063,02
20813,87 18598,91 570049,62 246331,73 997,85
117360,29 73255,54 17601,06 452689,33 173076,19
0,62 1,08 1886445,00 23359867,00 491,00 491,00 2,10 20063,02
85,89 967005,76 44,20 15,89 21702,02 318724,14
233331,14 380139,12 209122,03 380139,12 573757,30
720565,28 549548,19 720565,28 2456,17 46334,24 2,46
1,46 18842,85 1,00 10,06 89,94 80755,81 15008,37
250804,83 4285,89 246518,94 16,43 9360,27 153746,42 2672,98
156419,40 0,67 12,63 117428,85 0,50 9,48 1097,08 7708,57
40221,25 374,46 2438,63 23839,50 207,05 2262,03 49662,73
262,87 1562,35 38805,09 32972,33 33783,57 36228,74
28134,48 1,10 0,83 36228,74 28134,48 5216,94 1885,01
7101,95 51711,39 230294,11 467663,60 23216,90 81589,84
282840,65 10282,96 68779,73 467391,84 20327,48
60364,27 399660,78 636980,32 448771,97 699889,79
373731,00 100784,13 25039,98 125824,11 0,54 0,50
156419,40 117428,85 125824,11 117360,29 203532,63
27,26 20,47 21,93 20,45 9,89 9360,27 5400,20 7101,95 491,00 18852,27
3600,80 49,65 28,64 2,60 19,10 0,17 4979,56 1939,00 746,18 30575,27
17143,27 6133,79 7167,29 49763,14 9450,45 730015,73 3,13
0,58 72726,33 802742,06 3,44 82176,78 19,49 14,63
15,67 14,62 10,24 25,35 100,00
HIGH Japan Asia Eastern Asia 65093650,00
67240000,00 3,38 24,22 72,40 2272712,00 16285528,00
48681760,00 845,00 124766,00 372,72 781,71 924,95
2079,38 1890348,68 3465639,25 2677993,97 2677994,00 57750,21
59829,59 29138,40 1118806,33 661149,54 1668,92
230868,14 157942,48 27469,48 887938,19 503207,06
1,31 1,08 4975415,00 67240000,00 2079,38 2079,38 3,09 57750,21
85,89 2677993,97 43,70 14,01 90861,49 808835,95
1254700,65 1070003,87 699994,97 1070003,87 2154398,09
1969701,31 1599692,41 1969701,31 3204,04 159414,40 3,20
1,34 92841,41 1,87 13,35 86,65 73994,87 22478,52
331351,31 8687,17 322664,13 14,35 26942,98 386748,42
10412,53 397160,95 0,59 29,39 345160,09 0,51 25,54
2038,24 11631,34 55990,66 1802,93 8968,06 55311,26 421,01
3042,27 67464,73 812,58 3719,71 74031,45 61541,21 55160,96
92373,27 71735,15 1,50 1,30 92373,27 71735,15
13301,75 4806,26 18108,01 96680,99 340433,02
667044,44 109913,54 349655,97 774909,86 20574,76
89531,19 622105,15 68461,46 225188,24 933779,70
1377334,94 922383,93 2067377,75 1199532,34 297702,40
80368,67 378071,06 0,56 0,34 397160,95 345160,09
378071,06 230868,14 618441,07 18,43 16,02 17,55 10,72
37,28 26942,98 15544,17 18108,01 2079,38 57265,75

12699,22 47,05 27,14 3,63 22,18 0,17 8397,53 2828,15 1250,67
34604,05 17733,86 11646,82 11642,57 56220,17
11265,22 1980966,53 2,95 0,58 218525,07 2199491,60
3,27 229790,29 18,06 15,69 17,19 10,50 10,45 28,12 100,00
HIGH Jersey Europe Northern Europe 53380,00 59950,00
2,80 13,30 83,90 1678,60 7973,35 50298,05
0,28 0,38 0,96 1,61 1466,98 2689,46 2078,22 2078,00 51,49 53,10
1,08
59950,00 1,61 1,61 2,69 51,49 85,89 2078,22 x x
x x x x x x x
x x x x x x x x x
x x x 24,02 x x
x x x x x x x x x
x x x x x 75,75 58,83 x x x
x x x x x x x x x
x x x x x x x x x x
x x 0,00 0,00 0,00 0,00 x
x x x x x 24,02 13,86 1,61 66,96
27,47 35,87 20,70 2,41 41,02 0,17
0,00 0,00 0,00 0,00 0,00 0,58 0,00 0,00 0,00
0,00 x x x x x x x
HIGH Korea, Republic of Asia Eastern Asia
24994700,00 27231369,00 5,10 24,60 70,30 1388799,82
6698916,77 19143652,41 855,00 227,76 321,55
363,73 913,04 830036,88 1521734,29 1175885,58 23388,00
23388,12 24301,16 4731,41 328813,48 234953,74 518,75
55266,79 32441,35 4212,66 273546,69 202512,38 2,85
1,08 1630525,00 27231369,00 913,04 2348,18 3,35 23388,12
85,89 1175885,58 44,00 16,86 103322,81 394372,46
1271170,92 572613,60 349363,10 572613,60 1768866,18
1070308,86 847058,36 1070308,86 6495,69 105913,90 6,50
1,83 76113,54 4,67 14,14 85,86 59876,72 2557,04 47506,47
1027,59 46478,88 18,18 10911,57 198337,79 4385,00
202722,79 0,74 12,14 148736,79 0,55 8,91 742,91 3717,86
8682,63 793,53 3957,76 12029,26 115,30 1019,56 14002,44 331,36
1714,43 17675,34 16116,03 13739,92 35469,55 27544,91
2,20 2,00 35469,55 27544,91 5107,61 1845,51 6953,12 35364,39
112334,31 112999,90 46675,91 149323,64 180384,74
5814,55 30442,57 143609,32 27767,53 89355,84
240594,32 441493,14 281448,36 971676,41 564229,77
139921,40 37803,39 177724,80 0,65 0,20 202722,79
148736,79 177724,80 55266,79 485857,69 11,46 8,41
10,05 3,12 66,96 10911,57 6295,20 6953,12 2348,18 25078,37
5523,42 43,51 25,10 9,36 22,02 0,17 4046,75 1086,09 262,43 9331,06
4241,04 1196,13 5220,32 13970,81 3185,73 1073494,59 3,94 0,58
102724,93 1176219,52 4,32 105910,66 17,24 12,65 15,11
4,70 9,00 41,31 100,00
HIGH Kuwait Asia Western Asia Y 2473000,00
2370356,00 1,78 22,15 76,07 42192,34 525033,85
1803129,81 6,92 25,20 34,26 66,38
60346,03 110634,39 85490,21 85490,00 2035,82 2102,20
168,52 22778,91 16072,27 106,57 8535,43 3165,02 61,95 14243,47
12907,24 9,66 1,08 136197,00 2370356,00 66,38 66,38
2,80 2035,82 85,89 85490,21 50,40 21,57 3345,28 43909,94
426115,99 49376,52 14878,77 49376,52 473371,21
96631,74 62133,99 96631,74 19970,47 27199,18

19,97   1,99   24483,97   17,98   1,12   98,88   57458,46   42,56
955,03 13,12   941,91 22,13   949,80 21020,32   292,80 21313,11
0,90   1,22   9348,28 0,39   0,54   490,35 857,98 784,14 82,04   162,77
400,10 49,34   133,02 284,37 36,15   53,40   182,23 1987,88 427,44 3509,39
2725,32 1,77   6,38   3509,39 2725,32 505,35 182,60 687,95 23924,80
27437,75   10196,89   5311,47 5975,51 5441,08 2534,18 4233,01 3350,29
3372,80 2498,09 2429,39 67862,19   14564,63   119803,63   92863,66
17251,72   6221,87 23473,59   0,99   0,36   21313,11   9348,28
23473,59   8535,43 33961,33   4,50   1,97   4,96   1,80   86,76
949,80 547,97 687,95 66,38   1962,22 398,07 48,40   27,93   3,38   20,29
0,17   1080,79 132,88 11,39   2978,44 513,18 41,38   1217,47 3505,42 784,00
97415,74   4,11   0,58   13567,73   110983,47   4,68   14351,73
19,20   8,42   21,15   7,69   12,93   30,60   100,00
HIGH   Latvia Europe Northern Europe Y   1177735,00
910029,00   7,29   23,72   68,99   66341,11   215858,88
627829,01   29,00   2245,00 10,88   10,36   11,93   33,17
30154,47   55283,20   42718,84   33485,00   781,59 814,76
186,68 12764,94   7594,74 50,82   3894,61 1520,85 135,87 8870,33 6073,89
2,17   1,78   33505,00   910029,00   26,00   26,00   2,86   781,59
85,89   42718,84   46,71   20,58   1214,51 16087,14   35719,51
29378,46   6852,02 6852,02 53021,15   46680,10   24153,67
24153,67   5826,31 1952,11 5,83   1,90   1315,10 3,93   3,76   96,24
36817,51   98,19   2160,53 27,59   2132,94 21,72   364,65 7921,08 102,46
8023,54 0,88   0,30   4300,46 0,50   0,17   109,37 1089,38 2468,54 35,47
402,49 1155,79 17,99   440,67 4894,81 16,96   280,71 2984,01 2844,82 3382,60
1671,30 1297,90 0,59   0,38   1671,30 1297,90 240,67 86,96   327,63 5024,20
31301,70   31841,64   2008,66 12577,37   15600,23   857,41
12358,46   53001,64   1371,34 9720,35 35155,90   66725,89
53693,40   39200,78   20602,09   5644,91 1380,34 7025,25 0,77
0,43   8023,54 4577,71 7025,25 3894,61 632,56 15,13   8,63   13,25   7,35
55,64   364,65 210,38 327,63 26,00   690,34 89,32   52,82   30,47   3,77
12,94   0,17   232,05 54,47   19,01   818,01 596,95 317,45 292,86 1520,78
301,06 24454,73   2,69   0,58   4060,60 28515,32   3,13   4361,66
28,14   16,05   24,64   13,66   15,30   2,22   100,00
HIGH   Lithuania   Europe Northern Europe Y   1660967,00
1378419,00   6,44   25,70   67,86   88770,18   354253,68
935395,13   37,00   4666,00 37,00   4666,00 14,56   17,00   17,77   49,33
44849,99   82224,99   63537,49   47652,00   1183,88 1233,21
256,42 18373,37   11525,16   64,46   5035,28 2045,55 191,96 13338,09
9479,60 2,46   1,78   55887,00   1378419,00   37,00   37,00   2,68
1183,88 85,89   63537,49   46,38   20,10   1716,10 23797,08   59639,22
43481,51   10653,75   10653,75   85152,40   68994,68
36166,92   36166,92   6177,54 3452,44 6,18   1,85   2418,03 4,33
3,39   96,61   40544,28   118,09 2614,73 32,83   2581,90 21,86   552,33
12076,06   153,55 12229,62   0,89   0,50   6862,46 0,50   0,28
188,51 1613,17 4142,53 52,46   464,76 1215,51 29,65   553,72 8110,23 26,54
287,21 2839,44 4104,92 4547,01 2357,05 1830,44 0,57   0,40   2357,05 1830,44
339,42 122,64 462,05 8638,34 46467,12   50821,94   2972,81 14905,34
16476,99   1411,55 15585,02   83808,01   2135,64 10851,62
34623,46   95416,58   69460,99   54788,33   27962,05
7889,52 1873,46 9762,98 0,71   0,37   12229,62   6825,68 9762,98 5035,28
2313,37 14,36   8,02   11,47   5,91   60,24   552,33 318,66 462,05 37,00
1077,48 169,49 51,26   29,57   3,43   15,73   0,17   515,55 87,20   25,46
1303,67 802,80 349,80 611,24 2223,07 470,50 36637,42   2,66   0,58
5643,00 42280,42   3,07   6113,50 28,93   16,14   23,09   11,91   14,46
5,47   100,00

HIGH	Liechtenstein		Europe	Western	Europe			19199,00	
19199,00		0,80	39,40	59,80	153,59	7564,41	11481,00		
		0,03	0,36	0,22	0,61	551,29	1010,70	780,99	0,00
16,49	17,10								
	1,08	6839,00	19199,00		0,61		3,16	16,49	85,89
780,99		x	0,00			x			x
x	x		x	x		x	x	x	x
x	x	x	x	x	x	x	7,69	x	x
					x	x	x	x	x
x	x	x	x	x	x	x	x	x	27,24
21,16	x	x	x	x	x	x		x	x
x	x	x	x	x	x	x	x	x	x
x	x	x	x	x	x				0,00
0,00	0,00	0,00	x	x	x	x	x	x	7,69
4,44		0,00	20,93	8,80	36,76	21,21	0,00	42,03	0,17
					0,00	0,00	0,00	0,00	0,00
0,58	0,00	0,00	0,00	0,00	x	x	x	x	x
x	x								
HIGH	Luxembourg		Europe	Western	Europe	Y		254340,70	
289139,00		0,68	10,81	88,51	1966,15	31255,93		255916,93	
12,00	7270,00	12,00	7270,00	0,32	1,50	4,86	6,69	6077,41	11141,92
8609,67	15455,00		248,33	255,02	110,09	4313,28	2151,84	7,25	906,79
558,10	102,83	3406,50	1593,74	0,88	1,08	73264,00		289139,00	
12,00	12,00	4,15	248,33	85,89	8609,67	48,06	17,63	576,77	4377,72
4771,97	5649,34	2516,91	5649,34	9726,46	10603,83		7471,40	10603,83	
3363,94	2464,56	3,36	1,71	1209,15	1,65	15,42	84,58	253386,78	
88,31	1606,87	25,69	1581,18	17,90	115,86	2074,42	33,70	2108,12	0,73
0,53	1122,25	0,39	0,28	10,56	68,04	243,44	3,67	20,40	124,87
1,88	17,22	275,41	2,56	11,73	184,96	225,44	186,84	360,93	280,29
1,60	1,50	360,93	280,29	51,97	18,78	70,75	501,36	2043,80	3004,18
245,42	700,84	1595,08	96,42	536,24	2737,56	218,59	480,99	2001,48	5024,51
2911,93	8044,27	4368,39	1158,37	292,68	1451,06	0,50	0,31	2108,12	1122,25
1451,06	906,79	5015,62	21,67	11,54	14,92	9,32	42,55	115,86	66,84
70,75	12,00	256,42	61,72	45,18	26,07	4,68	24,07	0,17	66,12
13,40	3,69	232,09	67,69	17,27	80,75	305,54	64,12	10667,96	
3,69	0,58	838,71	11506,67		3,98	902,83	18,32	9,75	12,61
7,88	7,85	43,59	100,00						
HIGH	Malta	Europe	Southern	Europe	Y		184605,50		
253076,00		1,02	18,92	80,07	2581,38	47881,98		202637,95	
3,00	2202,00	3,00	2202,00	0,42	2,30	3,85	6,57	5974,37	10953,00
8463,68	3864,00	217,36	223,93	96,19	3762,77	1874,71	6,27	883,74	582,57
89,92	2879,03	1292,14	0,81	1,08	14647,00		253076,00		3,00
3,00	1,19	217,36	85,89	8463,68	48,06	17,65	144,18	3835,84	3402,26
2272,51	1571,04	2272,51	7382,27	6252,53	5551,06	6252,53	2917,02	427,26	2,92
1,57	196,91	1,34	5,73	94,27	57875,89		77,78	1422,01	22,67
1399,34	17,99	101,41	1824,42	29,56	1853,97	0,73	0,11	909,82	0,36
0,05	11,41	103,84	365,56	2,42	19,13	123,70	2,53	34,01	402,63
1,93	12,08	169,75	299,90	241,33	261,97	203,44	0,87	0,84	261,97
203,44	37,72	13,63	51,35	542,79	2950,21	4712,63	164,46	626,16	1651,14
124,73	953,83	4310,10	157,36	454,40	2027,53	6404,87	3802,87	5594,87	3205,77
805,66	214,79	1020,45	0,40	0,43	1853,97	909,82	1020,45	1082,59	1385,70
25,11	12,32	13,82	14,66	34,07	101,41	58,50	51,35	3,00	227,51
64,60	44,57	25,72	1,32	28,39	0,17	30,59	8,72	3,64	112,89
44,67	14,66	40,53	162,44	33,69	6286,23	2,48	0,58	589,82	6876,04
2,72	623,51	26,96	13,23	14,84	15,74	9,07	20,15	100,00	
HIGH	Monaco	Europe	Western	Europe			52000,00		52000,00

0,00	16,10	83,90	0,00	8372,00	43628,00				
0,00	0,40	0,83	1,23	1118,90	2051,31	1585,11	1585,00	44,66	45,89
20,62	494,85	131,44	0,33	38,97	24,44	20,29	455,89	107,00	0,31
1,08	7424,00	52000,00		1,23	1,23	2,37	44,66	85,89	1585,11
43,96	17,19	54,10	767,85	326,54	2272,51	198,05	2272,51	1148,49	x
x		x	x		x	x	x	x	x
x	18,54	329,16	5,43	323,73	17,46	20,84	363,83	6,10	369,93
0,71	0,05	67,35	0,13	0,01	0,85	6,86	33,21	0,23	1,79
14,78	0,15	1,97	36,65	0,20	1,38	25,31	25,72	24,35	73,79
57,31	x	x	x	x	x	x		39,43	202,57
384,18	13,75	62,23	185,15	7,55	57,43	353,12	14,58	53,25	283,25
x	x	x	x	x	x				369,93
67,35	0,00	38,97	x	x	x	x	x	x	20,84
12,02		1,23	57,91	23,82	35,98	20,76	2,13	41,14	0,17
3,82	1,45	0,53	14,71	8,13	2,57	5,45	23,70	4,84	4,84
0,01	0,58	x	x	x	x	x	x	x	x
x	x	x							
HIGH	Mauritius		Africa	Eastern	Africa			631687,60	
551379,00		5,97	23,68	70,35	32917,33		130566,55		
387895,13						5,40	6,27	7,37	19,04
17305,13		31726,07		24515,60		24516,00		473,56	492,60
72,80	6397,13	4576,26	9,18	659,15	219,56	63,62	5737,98	4356,70	3,15
3,69	10914,00		551379,00		19,04	19,04	3,45	473,56	85,89
24515,60		47,88	21,71	911,41	10281,13		32882,80		2272,51
4811,92	2272,51	44075,34		13465,06		16004,46		13465,06	
7993,66	872,43	7,99	2,03	650,88	5,96	3,10	96,90	19794,01	
17,19	468,93	5,27	463,65	26,97	220,94	5959,12	67,73	6026,86	1,09
0,12	2402,07	0,44	0,05	88,29	391,48	450,07	46,39	207,36	294,94
20,10	173,00	540,50	22,80	109,39	349,23	981,85	621,88	896,42	696,14
x	x	x	x	x	x		4234,21	11690,25	
6159,67	2608,67	6891,09	4479,12	967,19	4969,50	6409,90	1547,21	4156,25	4960,41
x	x	x	x	x	x			0,12	6026,86
2402,07	0,00	659,15	x	x	x	x	x	x	220,94
127,46		19,04	620,06	252,62	35,63	20,56	3,07	40,74	0,17
246,87	42,62	7,48	500,19	181,43	31,54	291,98	692,13	163,36	13628,42
2,47	0,58	0,00	13628,42		2,47	163,36	44,22	17,63	0,00
4,84	1,20	x	x						
HIGH	Netherlands		Europe	Western	Europe	Y		8955744,00	
8982405,00		2,08	16,11	81,81	186834,02		1447065,45		
7348505,53		37,00	92838,00		37,00	92838,00		30,64	69,46
139,62	239,72	217928,66		399535,88		308732,27		92838,00	
7714,69	7954,41	6238,54	174860,30		63138,16		124,16	18247,22	
12390,53		6114,38	156613,08		50747,63		0,48	1,08	
913865,00		8982405,00		37,00	37,00	0,41	7714,69	85,89	
308732,27		47,17	17,31	1745,28	133573,59		67722,14		
147897,83		54439,97		147897,83		203041,00		283216,70	
189758,83		283216,70		2260,43	20657,28		2,26	1,51	6890,01
0,75	2,68	97,32	101739,46		5440,64	97183,43		1659,22	95524,22
17,56	3599,24	63193,73		1097,65	64291,38		0,72	6,54	32488,08
0,36	3,31	223,74	1995,36	7269,88	77,47	710,23	4255,82	68,14	646,52
7438,50	81,73	511,81	6871,80	6848,70	6078,29	12708,94		9869,50	1,86
1,62	12708,94		9869,50	1830,09	661,26	2491,34	10551,93		59170,92
92830,15		5306,61	24367,98		56738,33		3301,60	19595,14	
77093,51		6343,96	19972,78		78091,22		149253,60		
100941,73		276965,61		163902,12		39883,05		10981,44	
50864,49		0,57	0,20	64291,38		32488,08		50864,49	

18247,22 117325,52 31,66 16,00 25,05 8,99 18,30 3599,24
2076,50 2491,34 37,00 7336,85 1624,11 49,06 28,30 0,50 22,14 0,17
1432,32 254,65 85,08 8475,17 3987,01 1115,18 1715,34 12833,91 2415,17
285631,87 3,18 0,58 29399,67 315031,54 3,51 31814,85
20,41 10,31 16,15 5,79 10,10 37,24 100,00
HIGH New Zealand Oceania Australia and New Zealand
2455038,00 2695100,00 5,84 19,29 74,87 157393,84
519884,79 2017821,37 25,81 24,95
38,34 89,11 81005,15 148509,44 114757,30 114757,00
2314,73 2403,84 978,43 44452,69 25338,93 72,31 12600,74
9223,60 906,12 31851,95 16115,34 1,02 1,08 210886,00
2695100,00 89,11 89,11 3,31 2314,73 85,89 114757,30 46,70
17,37 4161,51 40199,90 52533,21 54765,49 27481,18
54765,49 96894,63 99126,91 71842,59 99126,91
3595,21 7581,80 3,60 1,65 4110,62 1,95 16,02 83,98 78247,93
695,87 12588,30 235,50 12352,81 17,75 1079,92 19170,37
365,47 19535,84 0,72 1,53 10898,11 0,40 0,85 105,01
746,96 2736,99 23,86 145,20 981,52 24,68 225,61 2652,67 23,98 126,21
1509,44 2260,54 1787,86 3483,90 2705,53 1,54 1,51 3483,90 2705,53 501,68
181,27 682,95 4846,83 21764,81 32393,98 1513,12 4933,48 12106,08
1155,62 6540,48 26077,67 1954,72 5000,02 16534,28 47891,59
28854,82 73809,76 43665,37 10628,61 2925,58 13554,19
0,50 0,47 19535,84 10898,11 13554,19 12600,74
42538,04 20,16 11,25 13,99 13,00 41,60 1079,92 623,04 682,95
89,11 2343,92 551,86 46,07 26,58 3,80 23,54 0,17 899,47 241,45
46,83 3602,47 809,81 157,48 1156,53 4464,77 933,14 100060,05 3,71
0,58 7834,32 107894,36 4,00 8767,45 18,11 10,10 12,56 11,68
8,13 39,43 100,00
HIGH Norway Europe Northern Europe To be included 2705522,00
2715516,00 2,04 19,42 78,54 55396,53 527353,21
2132766,27 33,00 9943,00 33,00 9943,00 9,09 25,31 40,52 74,92
68109,58 124867,57 96488,58 42500,00 2332,27 2407,19
1069,82 41100,68 21788,73 45,15 6461,28 4358,15 1024,67 34639,41
17430,59 1,01 1,08 362522,00 2715516,00 33,00 33,00
1,22 2332,27 85,89 96488,58 46,58 16,79 1537,19 39169,25
42859,41 45472,36 20615,98 45472,36 83565,85
86178,80 61322,42 86178,80 3077,35 11156,06 3,08
1,50 5721,74 1,58 5,65 94,35 133500,23 790,26 13917,46
273,85 13643,61 17,26 1088,10 18785,79 377,06 19162,86
0,71 2,56 11702,91 0,43 1,56 74,16 634,57 2556,98 20,90
156,59 1196,65 13,50 164,96 2826,51 15,09 101,35 1819,69 2137,43 1843,63
3839,36 2981,57 1,80 1,62 3839,36 2981,57 552,87 199,76 752,63 3510,79
19032,85 31489,82 1788,70 6516,05 17061,37 679,75 4944,31
26737,09 1633,91 4865,16 21369,32 47032,12 28158,60
84481,46 45538,83 12165,33 3051,10 15216,43 0,56
0,24 19162,86 11702,91 15216,43 6461,28 33635,32
22,93 14,00 18,21 7,73 37,12 1088,10 627,76 752,63 33,00 2240,39
491,53 48,57 28,02 1,47 21,94 0,17 597,32 127,29 34,32 1996,34
562,01 126,26 736,05 2600,43 553,86 86732,66 3,19 0,58 8795,10
95527,76 3,52 9348,95 20,06 12,25 15,93 6,76 9,79 35,21
100,00
HIGH Oman Asia Western Asia Y 1349041,00
2367036,00 3,99 32,01 63,99 94444,74 757688,22
1514666,34 15,49 36,37 28,78 80,64
73306,03 134394,39 103850,21 103850,00 2032,97 2113,61
218,08 22945,69 13028,25 157,46 10222,81 1771,00 60,62

12722,88	11257,26	7,68	2,49	76332,00	2367036,00
80,64	80,64	3,41	2032,97	85,89	103850,21	53,68	24,18	4328,26
49152,50	378440,23	123060,62	12164,20	123060,62
431920,99	176541,38	65644,96	176541,38	18247,34
13928,56	18,25	2,26	12203,91	15,99	1,21	98,79	32247,93
34,71	953,89	11,68	942,21	27,15	948,47	25746,36	319,16	26065,52
1,10	0,84	8452,61	0,36	0,27	321,50	782,45	933,54	89,74	177,02
301,80	82,26	485,83	806,07	43,48	133,46	266,87	1782,49	1102,67	1914,40
1486,68	1,07	1,35	1914,40	1486,68	275,67	99,61	375,28	16309,92
23573,99	13902,23	6342,07	6024,12	4722,86	4197,58	13711,64
11518,09	3373,91	4469,68	4263,30	58458,46	31013,28	62784,59
41813,78	9040,98	2801,52	11842,51	0,50	0,43	26065,52
8452,61	11842,51	10222,81	119957,93	6,03	1,96	2,74
2,37	86,90	948,47	547,20	375,28	80,64	2285,52	709,22	41,50	23,94
3,53	31,03	0,17	1712,84	98,10	6,11	2327,00	315,01	32,98	1812,97
2653,01	741,35	177282,73	7,49	0,58	6844,97	184127,70	7,78
7586,32	14,16	4,59	6,43	5,55	4,12	65,15	100,00
HIGH	Panama	America	Central	America	1791921,00
1919877,00	14,41	17,72	67,88	276654,28	340202,20
1303212,51	14,00	11,00	45,37	16,33	24,76	86,46
78601,86	144103,41	111352,63	111353,00	1648,92	1735,38
181,63	18389,60	13168,93	57,07	4732,18	1865,10	124,56	13657,42
11303,84	4,80	2,43	52938,00	1919877,00	86,46	86,46
4,50	1648,92	85,89	111352,63	50,24	18,89	4343,60	31155,93
152462,01	78508,51	14129,43	78508,51	187961,54
114008,05	49628,97	114008,05	9790,29	5182,78	9,79	1,85
4203,93	7,94	3,81	96,19	27573,64	45,88	1131,90	14,07	1117,83
24,36	769,29	18743,20	235,92	18979,11	0,99	0,52	7392,92
0,39	0,20	76,52	418,98	939,47	49,75	218,05	695,10	28,84	170,53
876,60	38,76	128,48	672,90	1308,17	883,12	2542,88	1974,75	1,94	2,24
2542,88	1974,75	366,18	132,31	498,48	3734,85	12517,20	11899,97
2846,26	6809,42	8855,62	1452,33	5053,57	9392,20	2558,95	4460,30	8011,09	32826,26
19326,24	63809,38	43215,56	9188,55	2895,44	12083,99
0,63	0,25	18979,11	7392,92	12083,99	4732,18	70819,84
10,10	3,93	6,43	2,52	77,02	769,29	443,83	498,48	86,46	1680,72
381,14	45,77	26,41	5,14	22,68	0,17	418,68	88,26	27,67	1581,91
489,76	110,62	516,16	2108,54	435,70	114443,75	5,96	0,58	6984,55
121428,29	6,32	7420,25	15,63	6,09	9,95	3,90	6,11	58,32
100,00
HIGH	Poland	Europe	Eastern	Europe	Y	18180350,00
16460917,00	9,15	32,13	58,71	1506173,91	5288892,63
9664204,37	184,00	81302,00	184,00	81302,00	247,01	253,87
183,62	684,50	622272,05	1140832,08	881552,06	236970,00
14137,74	14822,24	7107,10	373791,60	205491,04	696,77
74769,41	41824,71	6410,33	299022,19	163666,33	1,21
1,78	549165,00	16460917,00	184,00	184,00	1,12	14137,74
85,89	881552,06	47,28	21,12	8699,89	298522,31	372004,89
541769,37	174711,21	174711,21	679227,08	848991,56
481933,41	481933,41	4126,30	22660,20	4,13	1,87	12410,73
2,26	2,91	97,09	33361,75	5223,37	115524,95	1232,15
114292,80	21,88	6595,87	144324,47	1555,91	145880,38	0,89
4,87	116034,46	0,50	2,74	1457,20	13721,97	31003,01
812,13	5779,56	11770,21	224,40	4057,59	46724,19	330,78	2802,31
23604,05	36028,61	30857,83	25799,42	20035,29
0,72	0,65	25799,42	20035,29	3715,12	1342,36	5057,48	67683,82
396578,84	389357,22	43998,07	180425,39	164660,10

11295,59   118042,72   484096,17   27599,96   105080,98
285676,64   873358,57   518610,20   625395,92   336721,87
90057,01   22560,37   112617,38   0,68   0,45   145880,38
82081,51   112617,38   74769,41   66584,73   21,48   12,08
16,58   11,01   38,85   6595,87 3805,34 5057,48 184,00 13069,60   2484,39
50,47   29,12   1,41   19,01   0,17   2656,42 767,47 197,96 4409,69 2287,70
758,90 3489,87 6950,36 1733,08 483666,49   2,94   0,58   65092,84
548759,33   3,33   66825,92   26,58   14,96   20,52   13,63   12,18
12,13   100,00
HIGH   Portugal   Europe Southern Europe Y   5673792,00
4913086,00   5,50   24,68   69,83   270219,73   1212549,62
3430807,95   104,00 131717,00   44,32   58,20   65,19
167,70 152457,97   279506,28   215982,13   133939,00   4219,69
4387,39 1729,15 87886,88   52710,84   221,44 18335,83   8358,11
1507,71 69551,05   44352,72   1,76   1,08   231227,00
4913086,00   104,00 104,00 2,12   4219,69 85,89   215982,13   45,06
16,71   4685,99 70523,53   128057,04   80062,26   48278,08
48278,08   203266,56   155271,79   123487,60   123487,60
4137,25 9566,43 4,14   1,53   6026,81 2,61   4,24   95,76   47063,50
805,97 15906,00   213,68 15692,32   19,47   1968,67 38330,15
521,94 38852,09   0,79   1,83   31856,77   0,65   1,50   225,05
1317,97 4688,81 113,20 883,88 6084,16 43,19   391,16 6323,77 63,19   474,06
9075,81 6131,09 6104,80 8051,58 6252,69 1,31   1,02   8051,58 6252,69 1159,43
418,93 1578,36 10421,48   39320,38   56900,99   6344,21 27696,67
74082,12   2088,56 11763,25   63470,14   4688,63 16751,96
98382,48   127443,78   89243,28   167364,09   91405,26
24100,43   6124,15 30224,58   0,62   0,37   38852,09   31856,77
30224,58   18335,83   4218,33 19,11   15,67   14,87   9,02   41,32
1968,67 1135,78 1578,36 104,00 3881,11 672,66 50,72   29,26   2,68   17,33
0,17   1155,39 374,34 154,86 4613,79 2055,35 758,26 1581,35 6921,90 1411,54
124899,14   2,54   0,58   17469,81   142368,95   2,90   18881,35
27,29   22,38   21,23   12,88   13,26   2,96   100,00
HIGH   Puerto Rico   America Caribbean   1356681,00
896515,00   1,09   15,21   83,70   9772,01 136359,93   750383,06
1,60   6,55   14,26   22,41   20368,33   37341,94
28855,14   28855,00   769,99 792,39 215,03 12712,13   7656,07
46,84   4275,76 2038,35 168,19 8436,37 5617,72 1,99   2,43   103138,00
896515,00   22,41   22,41   2,50   769,99 85,89   28855,14   47,76
16,76   1070,16 12903,88   26693,08   32320,60   6592,67 32320,60
40667,11   46294,64   20566,70   46294,64   4536,13 4678,48
4,54   1,56   3070,86 2,98   5,03   94,97   115043,25   86,32   1557,01
24,89   1532,12 17,75   359,23 6376,12 103,58 6479,71 0,72   0,75   3015,76
0,34   0,35   87,74   665,54 1780,50 26,19   171,16 676,11 27,92   307,51
1982,01 19,13   112,82 895,41 1769,51 1426,52 1925,24 1495,10 1,09   1,05
1925,24 1495,10 277,23 100,17 377,41 4193,37 19826,92   23099,06
1596,38 5490,44 8651,41 1374,42 9154,23 21745,67   1473,79 4352,86 10600,76
41690,60   27137,44   45359,87   28442,16   6531,82 1905,62
8437,45 0,94   0,48   6479,71 3015,76 8437,45 4275,76 24085,96   15,93
7,42   20,75   10,51   45,39   359,23 207,25 377,41 22,41   622,24 33,35
57,73   33,31   3,60   5,36   0,17   234,88 59,49   23,11   1115,08 524,25
179,53 302,08 1699,17 332,21 46626,84   5,20   0,58   4876,84 51503,69
5,74   5209,05 12,58   5,86   16,38   8,30   10,11   46,77   100,00
HIGH   Qatar   Asia   Western Asia   Y   1644000,00
2102667,00   1,17   53,70   45,14   24601,20   1129132,18
949143,88   4,03   54,20   18,03   76,27
69333,34   127111,13   98222,23   98222,00   1805,91 1882,18

152,47 19906,70   12380,49   124,21 8823,92 2168,52 28,26   11082,78
10211,98   11,73   1,08   146374,00   2102667,00   76,27   76,27
3,63   1805,91 85,89   98222,23   53,58   30,81   4086,45 55646,18
653895,61   61406,87   11543,46   61406,87   713628,24
121139,50   71276,08   121139,50   33939,19   49678,16
33,94   2,84   45519,96   31,10   0,76   99,24   69613,50   19,67
643,76 7,24   636,52 32,36   842,54 27264,41   310,11 27574,52
1,31   1,92   7592,59 0,36   0,53   130,40 308,06 181,66 46,11   46,52
31,50   17,90   90,59   101,39 15,29   18,13   22,10   602,16 183,07 2332,97
1811,74 3,87   9,90   2332,97 1811,74 335,95 121,39 457,33 6545,68 9798,08
2805,75 3629,06 2112,42 552,52 898,49 2600,16 1572,23 1358,45 799,54 389,63
23204,67   6310,60 89902,99   62452,62   12946,03   4184,33
17130,36   0,81   0,42   27574,52   7592,59 17130,36   8823,92
60018,11   3,86   1,06   2,40   1,24   91,44   842,54 486,08 457,33
76,27   1910,93 506,04 44,09   25,44   3,99   26,48   0,17   1780,84 96,67
2,42   1259,41 135,07 5,29   1878,32 1396,24 543,58 121683,07   5,79
0,58   9901,35 131584,42   6,26   10444,92   20,96   5,77   13,02
6,71   7,94   45,61   100,00
HIGH   San Marino   Europe Southern Europe   21960,00
21634,00   0,20   33,50   66,30   43,27   7247,39 14343,34
0,01   0,35   0,27   0,63   570,45 1045,82 808,14 457,00
18,58   19,21   8,25   279,63 133,88 0,34   43,04   26,80   7,91   236,58
107,08 0,83   1,08   1616,00 21634,00   0,63   0,35   2,90   18,58
85,89   808,14 47,12   16,38   16,73   304,36 279,27 356,19 134,68 356,19
600,36 x   x   x   x   x   x   x
x   x   x   7,07   119,51 2,04   117,47 16,62   8,67   144,03
2,50   146,53 0,68   0,01   77,20   0,36   0,01   0,53   3,12   15,43
0,17   1,19   11,16   0,11   0,98   16,87   0,14   0,81   15,34   13,86
12,77   31,16   24,20   x   x   x   x   x   x
24,87   93,31   179,29 10,96   40,50   133,75 5,34   29,45   162,32 11,98
32,18   167,75 x   x   x   x   x   x
146,53 77,20   0,00   43,04   x   x   x   x   x   x
8,67   5,00   0,35   23,94   9,91   36,21   20,89   1,48   41,41
0,17   4,03   1,04   0,34   16,35   6,02   1,58   5,19   22,89   4,66
4,66   x   0,58   x   x   x   x   x   x   x
x   x   x   x
HIGH   Romania Europe Eastern Europe Y   10384970,00
8680327,00   21,24   30,07   48,69   1843701,45   2610174,33
4226451,22   227,00 4709,00 227,00 4709,00 302,37 125,29 80,30   507,96
461779,98   846596,63   654188,31   292348,00   7455,25 7963,20
2528,17 185833,50   121190,63   412,77 38148,01   19100,27
2115,40 147685,49   102090,36   2,24   1,78   248716,00
8680327,00   227,00 227,00 2,62   7455,25 85,89   654188,31   46,15
21,55   10475,12   160689,56   370298,03   296184,12
112594,37   296184,12   541462,71   467348,79   283759,04
467348,79   6237,81 15514,44   6,24   1,97   10610,09   4,27
3,87   96,13   28652,84   1070,12 27464,42   314,73 27149,69
25,37   3478,20 88244,24   1022,96 89267,20   1,03   2,56   76538,96
0,51   1,28   1307,59 8982,78 22958,97   602,90 5447,19 16516,03
282,65 3679,82 32594,64   292,55 3297,12 26667,20   29498,79
27306,09   14737,13   11444,55   0,50   0,42   14737,13
11444,55   2122,15 766,78 2888,93 60620,50   260287,49
286227,64   31606,44   165128,79   222502,28   13487,33
102857,29   360743,27   20049,56   108370,21   331697,50
687219,85   475577,98   343324,27   199324,62   49438,69
13354,75   62793,44   0,72   0,44   89267,20   44574,94

62793,44 38148,01 232565,19 16,49 8,23 11,60 7,05
56,64 3478,20 2006,67 2888,93 227,00 6799,98 1088,12 51,15 29,51 3,34
16,00 0,17 1365,90 333,59 120,13 4948,35 3310,06 1424,55 1739,54 8733,26
1738,48 469087,28 5,40 0,58 36294,61 505381,89 5,82
38033,10 17,66 8,82 12,42 7,55 7,53 46,02 100,00
HIGH Saudi Arabia Asia Western Asia Y 11670000,00
13277789,00 2,41 24,80 72,79 319994,71 3292891,67
9664902,61 52,48 158,06 183,63 394,17
358337,35 656951,81 507644,58 507645,00 11403,85
11798,02 1490,02 169494,92 103113,67 1061,99 66040,19
12234,25 428,03 103454,73 90879,41 7,23 1,08
700118,00 13277789,00 394,17 394,17 2,97 11403,85 85,89
507644,58 50,67 29,38 19970,77 335037,62 2425793,74
366246,14 95420,32 366246,14 2780802,13 721254,53
450428,71 721254,53 20943,26 146627,55 20,94 2,67
127908,48 18,27 0,88 99,12 52728,51 124,78 4388,09 49,97
4338,12 34,77 5320,39 184969,54 2130,63 187100,17 1,41 9,87
70671,28 0,53 3,73 4754,05 10203,79 5315,92 1601,42 3141,05
2719,24 1878,87 4267,84 3268,60 1194,05 1896,09 2111,24 22378,69 11030,13
16560,70 12860,69 0,74 1,17 16560,70 12860,69
2384,74 861,67 3246,41 225079,33 315082,39 79592,24 92385,19
106055,53 42033,85 90075,01 130445,08 43285,41
75255,68 69149,80 30396,35 779144,47 389486,16
576583,07 454125,08 83027,96 30426,38 113454,34
0,85 0,50 187100,17 70671,28 113454,34 66040,19
283988,55 6,73 2,54 4,08 2,37 84,28 5320,39 3069,48 3246,41
394,17 11621,10 2837,05 45,78 26,41 3,39 24,41 0,17 11888,30
986,58 62,75 23357,77 3597,72 225,16 12895,80 27030,54
6627,77 727882,30 5,48 0,58 65576,61 793458,91 5,98
72204,38 23,58 8,91 14,30 8,32 9,10 35,79 100,00
HIGH Seychelles Africa Eastern Africa 48493,00
48493,00 3,00 23,00 74,00 1454,79 11153,39 35884,82
0,24 0,54 0,68 1,46 1323,42 2426,27 1874,84
69,00 41,65 43,10 8,89 726,18 442,40 2,90 182,71 39,39 5,99
543,47 403,01 2,87 3,69 1125,00 48493,00 1,46 0,00 3,00
41,65 85,89 1874,84 49,43 23,44 0,00 976,10 2800,63 3606,42 403,01
3606,42 3776,73 4582,52 1379,11 4582,52 7788,19 87,62 7,79 2,01 64,97
5,78 0,00 100,00 23199,22 2,24 63,38 0,77 62,61 27,95
19,43 543,12 x 8,00
28,65 25,00 5,13 16,20 16,23 1,65 9,51 34,86 1,48 5,82
23,68 71,74 37,97 68,82 53,44 x x x x x
x 380,69 862,09 351,93 284,41 560,50 256,30 79,42 279,62
414,49 107,02 244,76 336,22 x x x x x x
x 0,38 0,00 0,00 0,00 182,71 x x x x
x x 19,43 11,21 0,00 52,86 22,22 36,76 21,21
0,00 42,03 0,17 17,04 2,30 0,37 18,63 5,03 0,85 19,47
23,95 7,21 4589,73 9,46 0,58 0,00 4589,73 9,46 7,21 0,00
0,00 0,00 3,98 0,16 x x
HIGH Singapore Asia South-Eastern Asia
2185200,00 2230400,00 0,03 15,55 84,41 669,12 346827,20
1882680,64 39,00 13740,00 0,11 16,65 35,77
52,53 47753,07 87547,29 67650,18 67650,00 1915,62
1968,15 437,62 40677,01 30229,72 96,57 20817,38 16333,60
341,04 19859,63 13896,12 2,33 1,08 339998,00
2230400,00 52,53 52,53 2,36 1915,62 85,89 67650,18 46,43
17,49 2438,80 33496,80 86938,09 45047,11 22780,23

45047,11 122873,68 80982,71 58715,83 80982,71
5509,04 18730,63 5,51 1,61 13252,68 3,90 9,22 90,78
152438,13 282,43 5064,21 114,12 4950,09 17,53 893,72 15664,01
361,12 16025,13 0,72 2,44 9092,20 0,41 1,39 159,62 868,60
1279,90 39,06 217,97 478,78 20,71 273,30 1231,15 21,01 133,24 689,53
1871,48 1088,49 3100,98 2408,16 1,66 2,21 3100,98 2408,16 446,54 161,35
607,89 7468,72 25281,65 16953,67 2977,63 9119,31 7857,91 972,48
7719,57 13676,57 2333,05 7332,59 10776,24 53117,84 26508,63
88014,29 58647,19 12674,06 3929,36 16603,42 0,74
0,93 16025,13 9092,20 16603,42 20817,38 18444,59
13,04 7,40 13,51 16,94 49,10 893,72 515,61 607,89 52,53 1875,87
414,01 47,64 27,49 2,80 22,07 0,17 494,31 93,55 23,19 1425,91
509,44 139,63 595,60 1981,90 427,86 81410,57 3,65 0,58 9596,78
91007,35 4,08 10024,64 17,61 9,99 18,24 22,87 11,02
20,27 100,00
HIGH Slovakia Europe Eastern Europe Y 2794918,00
2583635,00 2,79 36,09 61,12 72083,42 932433,87
1579117,71 33,00 9666,00 33,00 9666,00 11,82 44,76 30,00 86,58
78710,68 144302,90 111506,79 42500,00 2219,00 2305,58
450,84 36122,01 23905,00 83,98 9690,15 5680,03 366,86 26431,86
18224,97 2,22 1,78 104574,00 2583635,00 33,00 33,00
1,28 2219,00 85,89 111506,79 47,75 22,37 1575,77 49641,06
112469,47 90485,75 20456,93 90485,75 163686,31
141702,59 71673,77 141702,59 6335,50 6625,29 6,34 1,98
4552,26 4,35 2,33 97,67 40475,53 249,80 6085,07 87,81 5997,26
24,01 1035,26 24854,75 363,92 25218,67 0,98 1,02 12928,70
0,47 0,49 264,47 2421,26 5255,59 91,48 817,73 1671,53 54,29 950,24
8831,54 41,25 447,86 2810,17 5903,98 5374,21 3966,22 3080,08 0,67 0,57
3966,22 3080,08 571,14 206,37 777,50 12273,57 69178,03 68156,53
5301,86 26075,21 25153,95 2616,80 26454,30 96825,29
3693,86 16651,48 38053,58 143932,16 94376,07 96691,77
54089,11 13923,61 3623,97 17547,59 0,68 0,38 25218,67
12228,78 17547,59 9690,15 77017,41 15,41 7,47 10,72
5,92 60,48 1035,26 597,27 777,50 33,00 2071,77 406,24 49,97 28,83
1,59 19,61 0,17 264,47 72,62 21,60 1292,73 829,75 304,19 344,29
2223,88 426,32 142128,90 5,50 0,58 10142,50 152271,41
5,89 10568,82 16,56 8,03 11,52 6,36 6,94 50,58 100,00
HIGH Slovenia Europe Southern Europe Y 1030467,00
982585,00 4,28 34,10 61,61 42054,64 335061,49
605370,62 15,00 13065,00 15,00 13065,00 6,90 16,08
11,50 34,48 31347,23 57469,92 44408,58 19318,00
843,91 878,39 432,85 20284,66 10995,69 30,32 5093,64 3669,19
402,53 15191,02 7326,50 0,93 1,08 53590,00 982585,00
15,00 15,00 1,53 843,91 85,89 44408,58 46,97 19,54 704,61
16488,12 17175,13 19615,49 9141,48 19615,49 34367,86
36808,22 26334,21 36808,22 3497,70 1874,42 3,50 1,75
936,73 1,75 7,33 92,67 54539,81 339,91 7002,69 96,35 6906,34
20,32 393,72 7999,67 111,60 8111,27 0,83 0,44 5001,23 0,51 0,27
36,37 389,58 1067,00 12,30 163,75 698,41 4,77 80,42 1401,65 7,42
68,12 1099,79 1190,46 994,55 1462,32 1135,61 1,23 1,14 1462,32 1135,61
210,57 76,09 286,66 1737,82 11636,53 13585,37 862,96 5352,57
9253,60 269,74 2629,81 14282,03 810,23 3051,86 12695,86 27202,86
15754,28 33415,01 17988,77 4811,76 1205,25 6017,01 0,61
0,52 8111,27 5001,23 6017,01 5093,64 12585,07 23,60 14,55 17,51
14,82 29,52 393,72 227,15 286,66 15,00 799,40 163,53 49,25 28,41
1,88 20,46 0,17 125,71 46,85 19,29 517,71 405,38 180,75 178,99

983,34 192,95 37001,17 3,77 0,58 3477,83 40479,00 4,12
3670,78 20,04 12,36 14,86 12,58 9,07 31,09 100,00
HIGH Spain Europe Southern Europe Y 23662230,00
19779312,00 4,03 20,43 75,54 797106,27 4040913,44
14941292,28 347,00 489643,00 347,00 489643,00 130,73 193,96
283,88 608,57 553248,93 1014289,71 783769,32 489643,00
16987,80 17596,37 9396,88 317740,76 148345,08 562,73
62018,61 36417,34 8834,15 255722,15 111927,73 0,78
1,08 1281485,00 19779312,00 347,00 347,00 1,75 16987,80
85,89 783769,32 45,50 16,28 15786,78 276512,15
237689,93 320977,79 134384,14 320977,79 529988,85
613276,71 426683,07 613276,71 2679,51 34337,53 2,68
1,48 15399,73 1,20 7,22 92,78 64789,16 6108,78
110959,20 2053,85 108905,35 17,83 7925,54 141294,02 2664,67
143958,69 0,73 9,33 73942,27 0,37 4,79 1032,52 5529,46
20307,39 306,26 1775,16 12629,28 179,79 1338,86 25244,00
189,49 932,45 21251,44 19622,29 18139,06 33578,66
26076,49 1,71 1,44 33578,66 26076,49 4835,33 1747,12
6582,45 48174,19 166770,09 238917,09 19043,77 60959,64
152342,65 8828,52 41692,43 238227,05 15773,25 39758,91
223121,81 425367,61 259836,06 727910,88 373537,00
104819,17 25026,98 129846,15 0,66 0,31 143958,69
73942,27 129846,15 62018,61 203511,00 27,16 13,95
24,50 11,70 22,68 7925,54 4572,47 6582,45 347,00 15324,81 2479,80
51,72 29,84 2,26 16,18 0,17 3805,34 1423,10 491,81 14877,93
5653,39 1692,99 5392,37 21095,65 4397,01 617673,72 3,12 0,58
75051,07 692724,79 3,50 79448,08 20,78 10,67 18,74
8,95 11,47 29,38 100,00
HIGH Sweden Europe Northern Europe Y 5081363,00
5131486,00 1,69 18,42 79,89 86722,11 945219,72
4099544,17 36,00 40684,00 36,00 40684,00 14,22 45,37
77,89 137,48 124985,74 229140,52 177063,13 46364,00
4407,26 4544,75 1718,87 71312,75 41044,82 95,17 14559,15
10151,70 1623,70 56753,60 30893,12 1,19 1,08
541064,00 5131486,00 36,00 36,00 0,70 4407,26 85,89
177063,13 46,31 15,93 1667,14 70194,05 87694,27 79621,15
34733,06 79621,15 159555,46 151482,34 106594,25
151482,34 3109,34 16823,53 3,11 1,40 9246,49 1,71 3,45
96,55 105440,02 1262,72 21063,62 340,39 20723,23 16,41
2056,18 33745,15 554,28 34299,44 0,67 3,62 20984,11
0,41 2,21 122,13 1552,52 8371,58 41,55 353,73 3246,61 26,25 455,55
8446,58 27,28 217,12 4514,73 5942,67 5046,63 7210,87 5599,81 1,21 1,11
7210,87 5599,81 1038,37 375,19 1413,55 5829,06 45851,43 100577,81
3826,37 13385,95 42469,61 1365,99 13859,76 82815,10
3625,40 9657,79 49708,27 116575,28 72683,40 141453,28
80650,53 20369,27 5403,59 25772,86 0,50 0,28 34299,44
20984,11 25772,86 14559,15 55866,79 21,50 13,15
16,15 9,12 40,07 2056,18 1186,27 1413,55 36,00 4215,98 937,53 48,77
28,14 0,85 22,24 0,17 925,21 214,37 83,94 6330,05 2378,80 748,34
1167,56 8958,30 1680,89 153163,23 2,98 0,58 14896,71
168059,95 3,28 16577,61 20,41 12,49 15,34 8,66 9,86
33,24 100,00
HIGH Switzerland Europe Western Europe To be included
4446085,00 4705782,00 2,59 20,34 77,07 121879,75
957156,06 3626746,19 51,00 91104,00 19,99
45,94 68,91 134,84 122581,77 224733,25 173657,51 91104,00

4041,64 4176,48 1920,90 93511,33 54983,71 190,60 30527,52
21620,43 1730,30 62983,81 33363,29 1,13 1,08
752248,00 4705782,00 51,00 51,00 1,08 4041,64 85,89
173657,51 46,73 17,12 2383,39 69187,52 83715,13 80479,82
39148,55 80479,82 155286,04 152050,73 110719,45
152050,73 3299,90 24823,42 3,30 1,52 13382,38 1,78
5,26 94,74 159856,11 1456,24 26096,57 460,89 25635,69
17,60 1885,60 33194,16 596,78 33790,94 0,72 5,40 24743,00
0,53 3,96 113,88 950,39 5069,74 23,96 189,70 1707,83 19,74 235,41
5982,13 20,67 133,40 2805,60 3537,12 3338,47 6309,36 4899,72 1,78 1,47
6309,36 4899,72 908,55 328,28 1236,83 5350,77 28252,87 56594,21
1922,12 7177,07 21080,38 987,50 7124,40 53163,09 2077,41 6075,60
30580,12 68594,36 44179,32 122355,64 64840,04
17619,21 4344,28 21963,49 0,47 0,65 33790,94 24743,00
21963,49 30527,52 41025,77 21,76 15,93 14,14 19,66
28,50 1885,60 1087,86 1236,83 51,00 3943,67 919,21 47,81 27,58 1,29
23,31 0,17 925,29 179,59 53,68 3152,24 1227,21 311,55 1122,78 4483,29
930,61 152981,33 3,25 0,58 12694,90 165676,23 3,52
13625,51 20,40 14,93 13,26 18,43 8,22 24,76 100,00
HIGH Taiwan, China Asia Eastern Asia 11500000,00
11500000,00 5,03 36,76 58,22 578450,00 4227400,00
6695300,00 94,87 202,92 127,21 424,99
386356,09 708319,50 547337,80 547338,00 9876,97 10301,96
3077,23 155991,97 85703,76 477,39 28945,05 6440,91 2599,85
127046,91 79262,85 1,66 1,40 752248,00 11500000,00
424,99 424,99 3,70 9876,97 85,89 547337,80 47,14 18,38 20034,24
181534,49 306858,29 260460,77 84996,85 260460,77
508427,01 462029,50 286565,58 462029,50 4421,10 x
4,42 1,75 x 2,67 5,07 94,93 x 1217,77 28493,29
397,83 28095,46 23,07 4608,03 106313,05 1505,39 107818,44
45487,10 0,40 619,99 2904,49 6403,77 655,63 2593,97
5351,80 101,42 885,18 5757,45 200,67 980,27 5137,16 10692,60 5799,08
16319,45 12673,34 x x x x x x
30705,20 90865,29 78557,54 35927,83 90160,77
77466,90 5930,33 29742,30 66523,00 17151,82 48425,52
80075,55 299667,24 150116,15 x x x x
0,25 107818,44 45487,10 0,00 28945,05 x
x x x x x 4608,03 2658,51 424,99 12960,47
5268,93 35,55 20,51 3,28 40,65 0,17 1333,18 146,98 42,57 4215,17
2247,79 454,38 1494,35 6614,42 1346,06 463375,55 4,03 0,58 0,00
463375,55 4,03 1346,06 23,27 9,82 0,00 6,25 0,29 x
x
HIGH Trinidad and Tobago America Caribbean
719533,50 645810,00 3,03 26,64 70,34 19568,04
172043,78 454262,75 3,21 8,26
8,63 20,10 18271,14 33497,09 25884,11 25884,00
554,66 574,76 56,97 5321,73 3819,63 5,65 460,23 183,67 51,33 4861,50
3635,96 2,97 2,43 21530,00 645810,00 20,10 20,10 3,11
554,66 85,89 25884,11 48,97 23,88 984,27 13244,06 39946,38
32825,72 4289,66 32825,72 54174,71 47054,05 18518,00
47054,05 8388,65 1806,08 8,39 2,20 1331,73 6,19 3,02 96,98
33337,98 24,55 655,47 7,28 648,19 26,40 258,78 6832,40 76,74
6909,14 1,07 0,23 2064,05 0,32 0,07 79,38 479,64 629,66 30,79
197,63 366,88 25,29 261,16 668,17 22,00 128,88 442,03 1119,62 807,40
1021,08 792,95 0,91 0,98 1021,08 792,95 147,04 53,13 200,16 3776,22
14189,40 8848,00 1691,89 5935,70 5180,40 1203,60 7522,96 8135,38 1339,68

4095,44 5629,01 30269,35 18749,81 27605,24 18414,27
3975,15 1233,76 5208,91 0,81 0,07 6909,14 2064,05 5208,91 460,23 32411,72
12,75 3,81 9,62 0,85 72,97 258,78 149,29 200,16 20,10 523,90
95,73 49,39 28,50 3,84 18,27 0,17 158,98 30,47 6,53 673,02
224,30 48,62 191,63 913,53 183,46 47237,51 7,31 0,58 3010,75
50248,26 7,78 3194,21 13,75 4,11 10,37 0,92 6,36 64,50
100,00
HIGH United Arab Emirates Asia Western Asia Y
5422900,00 7388315,00 1,39 34,22 64,39 102697,58
2528281,39 4757336,03 16,84 121,36
90,39 228,59 207808,45 380982,16 294395,30 294395,00
6345,58 6574,17 665,82 72444,63 43344,62 331,37 20256,54
3963,61 334,44 52188,09 39381,01 3,07 1,08 421142,00
7388315,00 228,59 228,59 3,09 6345,58 85,89 294395,30 49,17
38,29 11239,26 242995,01 749929,61 265070,61 42115,21
265070,61 1004163,88 519304,89 296349,48 519304,89
13591,24 57238,43 13,59 3,44 42746,80 10,15 1,39
98,61 57001,09 70,12 2534,68 25,88 2508,80 35,78 2960,49
105922,30 1092,66 107014,96 1,45 6,10 27247,59 0,37
1,55 1678,46 2230,96 382,05 643,19 794,38 198,33 95,39 326,63 166,65
228,79 202,08 97,32 5540,45 940,87 7695,54 5976,20 1,39 6,35 7695,54
5976,20 1108,16 400,41 1508,56 79177,19 71949,00 6001,69 37269,80
29787,38 3396,19 4835,46 9852,48 2576,52 14357,98 8293,93 1806,55
221316,00 38800,88 307402,18 246454,91 44265,91
16512,48 60778,39 0,82 0,27 107014,96 27247,59
60778,39 20256,54 304007,40 10,66 2,71 6,05 2,02
78,56 2960,49 1707,99 1508,56 228,59 6773,59 1876,53 43,71 25,22 3,37
27,70 0,17 2965,35 344,13 5,04 3347,27 458,62 13,31 3311,17 3810,33
1182,17 520487,05 7,04 0,58 35129,91 555616,97 7,52
36312,08 19,26 4,90 10,94 3,65 6,54 54,72 100,00
HIGH United Kingdom Europe Northern Europe Y 32512700,00
32694769,00 1,05 18,12 80,83 343295,07 5924292,14
26427181,78 261,00 220985,00 56,30 284,37 502,12
842,78 766166,24 1404638,11 1085402,18 336136,00 28080,45
28923,24 23252,74 649143,95 259691,25 244,38 33136,46
21563,71 23008,36 616007,49 238127,55 0,63 1,08
2707744,00 32694769,00 261,00 261,00 0,80 28080,45 85,89
1085402,18 47,36 16,42 12359,85 461182,02 313652,11
520920,54 261157,87 520920,54 787193,97 994462,40
734699,74 994462,40 2407,71 65194,52 2,41 1,45 25976,31
0,96 4,50 95,50 82818,88 18954,74 322150,28 6076,72
316073,56 16,68 13100,75 218457,26 4199,98 222657,24
0,68 18,44 151762,38 0,46 12,57 1650,10 11884,10 39858,60
468,48 2464,77 17743,76 363,81 3612,64 36028,53 374,83 1912,58
27659,82 35668,24 27493,30 46138,21 35829,97
1,29 1,30 46138,21 35829,97 6643,90 2400,61 9044,51 77678,39
347083,16 496789,97 28755,54 84199,05 220096,50
17467,85 105454,98 374572,54 27531,85 71602,14
300739,49 776678,29 447160,83 1004662,59 582751,42
144671,41 39044,35 183715,76 0,56 0,10 222657,24
151762,38 183715,76 33136,46 403190,57 28,28 19,28
23,34 4,21 24,89 13100,75 7558,19 9044,51 261,00 26855,14
5935,19 48,78 28,14 0,97 22,10 0,17 12251,20 3561,58 1116,93
38055,86 13483,18 3841,97 16185,09 52819,70 11454,79
1005917,20 3,08 0,58 106187,71 1112104,91 3,40
117642,50 20,02 13,65 16,52 2,98 10,58 36,25 100,00

HIGH United States America North America 162993000,00
157538068,00 1,36 19,91 78,74 2142517,72 31365829,34
124045474,74 5250,00 351,37 1505,56 2356,86 4213,80
3830724,30 7022994,56 5426859,43 6761364,00 135304,23
139518,02 72793,08 3182326,56 1716945,06 7504,58
621312,54 265301,21 65288,49 2561014,03 1451643,85
1,31 1,08 20936600,00 157538068,00 4213,80 5250,00 2,67
135304,23 85,89 5426859,43 47,44 16,99 249055,73
2299064,80 3193992,06 2667658,32 1637241,28 2667658,32
5742112,59 5215778,85 4185361,80 5215778,85 3644,90
763119,14 3,64 1,62 424477,30 2,03 7,57 92,43
132898,67 45760,42 786166,96 14131,13 772035,83
16,87 63125,29 1065003,02 19493,52 1084496,54 0,69
144,13 909324,67 0,58 120,85 11058,22 87174,15
204711,21 2791,89 17311,92 58416,96 3977,28 35552,94
215174,99 2421,87 13355,98 94996,14 206045,57
158698,45 231300,55 179623,17 1,12 1,13 231300,55
179623,17 33307,28 12034,75 45342,03 517651,84
2534632,76 2499143,14 186364,12 649616,15 879441,62
189169,40 1024713,21 2182778,38 200130,75 594939,82
1291240,15 5014459,78 3166959,36 5629081,38 3584529,52
810587,72 240163,48 1050751,20 0,67 0,39 1084496,54
909324,67 1050751,20 621312,54 1549893,90 18,89 15,84
18,30 10,82 36,16 63125,29 36418,77 45342,03 5250,00
131630,96 26836,91 47,96 27,67 3,99 20,39 0,17 68133,86
7440,01 1748,08 295620,45 74772,68 14404,52 76156,56
375194,63 74924,30 5290703,14 3,36 0,58 607334,19
5898037,34 3,74 682258,49 18,39 15,42 17,82 10,53 11,57
26,28 100,00
HIGH Uruguay America South America 1765146,00
1538385,00 8,41 18,83 72,76 129378,18 289677,90
1119328,93 21,22 13,90 21,27 56,39
51263,46 93983,02 72623,24 72623,00 1321,27 1377,66
647,11 31661,67 15088,90 135,89 11211,70 4651,56 511,22
20449,98 10437,34 1,04 2,43 53629,00 1538385,00
56,39 56,39 3,67 1321,27 85,89 72623,24 48,28 19,59 2722,34
25877,97 28905,92 64792,17 12367,65 64792,17
57506,22 93392,47 40967,95 93392,47 3738,09 2004,70
3,74 1,86 1007,68 1,88 8,09 91,91 34860,58 285,95 6219,07
75,30 6143,76 21,49 616,43 13244,24 162,33 13406,57 0,87
0,47 6441,31 0,42 0,22 75,82 623,41 1429,78 49,14 305,80 1020,90
17,75 218,70 1725,47 46,78 219,17 1879,60 1871,07 1704,09 2504,89 1945,24
1,34 1,14 2504,89 1945,24 360,70 130,33 491,04 3527,26 17980,96
18494,66 2801,97 9514,37 13439,37 850,32 6186,88 18284,72
2987,24 7580,37 21727,53 44469,24 30942,22 59533,11
35320,99 8572,77 2366,51 10939,27 0,71 0,73 13406,57
6441,31 10939,27 11211,70 51393,63 23,31 11,20 19,02
19,50 26,97 616,43 355,64 491,04 56,39 1242,26 213,80 49,62 28,63
4,54 17,21 0,17 269,37 53,43 15,89 1198,24 365,70 92,67 328,09
1594,83 319,20 93711,68 6,09 0,58 6322,90 100034,58 6,50
6642,11 13,40 6,44 10,94 11,21 6,64 51,38 100,00
HIGH TOTAL 603492660,60
588081640,00 18271439,08 135335126,33
434460968,92 10342,00 3179176,00 3775,00 3463829,00 2997,80
9577,36 8287,53 17864,89 16240810,50 29774819,25 23007814,88
505763,85 523628,74 266780,61 10859563,18 5676621,97

22409,39 2219332,57 1191155,59 244371,22 8640230,61
4485466,37 1,08 1,08 53374868,00 588081640,00 17864,89
16580,03 3,04 505763,85 86,00 23007814,88 45,88 17,00
761901,94 8598923,89 10166035,54 10166035,54 5368139,08
10166035,54 19526861,36 19526861,36 14728964,90 19526861,36
3320,43 1772277,14 3,32 1,59 922679,38
188325,96 3244380,62 17,23 235780,22
4061897,62 4401389,42 0,75 399,47 3000834,66 0,51
272,36
723140,70 597425,15 856406,00 665067,00
1,18 1,11 856406,00 665067,00 123322,46 44559,49
167881,95 1788239,64 7190824,20 8560956,22 868187,36
2782643,66 4580211,36 486642,03 2487265,80 8218706,30
698223,32 2005606,39 6302583,60 17010284,04 10518167,51
20145055,32 11709058,65 2900887,97 784506,93 3685394,89
0,63 0,38 4401389,42 2923547,31 3606536,50 2219531,42
7463118,14 235780,22
135202,00 164404,30 16580,03 493141,58 105579,33
47,81 27,42 3,36 21,41 0,17 172810,47 30055,95 8960,75
661147,44 218657,74 64214,04 205853,33 901209,87
183772,49 19710633,85 3,35 0,58 2130158,25 21840792,10
3,71 1226669,31 20,15 13,39 16,51 10,16 5,62 34,17 100,00

0,00 0,00

SEARO Bangladesh Asia Southern Asia 79094810,00
60827611,00 38,30 21,32 40,38 23296975,01 12968446,67
24562189,32 5731,06 907,79 1178,99 7817,83
7107120,19 13029720,35 10068420,27 10068420,00 63367,00
71184,83 19067,49 1465487,92 841582,66 7971,09
730126,10 329999,01 11096,41 735361,82 511583,65
2,29 1,34 324239,00 60827611,00 7817,83 7817,83 12,85 63367,00
104,17 10068420,27 50,20 20,17 392434,02 1277904,94
3245097,73 2034097,27 835237,91 2034097,27 4915436,69
3704436,23 2505576,87 3704436,23 8080,93 26201,53 8,08
2,75 17297,86 5,33 14,57 85,43 5330,46 1131,76 30152,87
353,30 29799,57 26,33 11287,90 297213,70 3523,73
300737,43 0,49 1,60 322638,00 0,53 1,72 8142,69 33581,30
37200,14 7482,49 28875,42 51494,44 4357,90 18070,96
29657,19 3780,91 20698,72 46083,37 107646,77 72155,33
126950,97 86165,13 1,18 1,19 126950,97 86165,13
18280,94 5773,06 24054,00 384059,57 1008351,62
515663,43 398064,56 854852,83 699818,71 216668,98

526274,04 424678,79 229763,95 641964,73 630257,91
3050489,28 1966317,27 3597530,95 2348100,52 518044,46
157322,73 675367,19 1,11 1,20 300737,43 322638,00
675367,19 730126,10 1675567,51 6,12 6,56 13,74 14,85
58,72 11287,90 23561,16 24054,00 7817,83 70691,99
28025,10 15,97 33,33 11,06 39,64 0,17 30106,59 5099,69
1978,74 143652,58 40139,08 6601,22 35865,86 185992,06
36828,42 3741264,64 6,15 0,58 390362,24 4131626,88
6,79 427190,65 7,28 7,81 16,35 17,67 10,34 40,55 100,00
SEARO Bhutan Asia Southern Asia 408145,90
374512,00 55,78 10,15 34,06 208902,79 38012,97
127558,79 51,39 2,66 6,12 60,17
54703,47 100289,69 77496,58 77497,00 390,00 450,17
144,26 9309,38 5069,53 49,65 3947,07 1457,61 94,61 5362,31 3611,92 2,06
1,34 2409,00 374512,00 60,17 60,17 16,07 390,00 104,14 77496,58
50,14 18,50 3017,12 7215,68 16656,09 11424,56 5378,48 11424,56
26888,89 21657,35 15611,28 21657,35 7179,71 172,96
7,18 2,73 107,14 4,45 17,79 82,21 6432,37 6,54 153,07 1,95
151,13 23,11 69,50 1606,02 20,72 1626,74 0,43 0,01 2517,69 0,67
0,02 41,14 138,39 221,56 10,07 58,00 108,91 21,75 77,45 162,79
8,26 47,05 104,56 357,76 243,63 655,09 444,63 1,83 1,83 655,09
444,63 94,33 29,79 124,12 2043,30 4067,16 3044,47 620,69 1747,19 1557,82
1069,85 2252,68 2184,48 541,16 1459,99 1496,11 10012,44 6550,54 18333,84
11954,88 2640,07 800,98 3441,05 0,92 1,05 1626,74 2517,69 3441,05
3947,07 10124,80 6,05 9,36 12,80 14,68 57,11 69,50 145,06
124,12 60,17 471,11 196,38 14,75 30,79 12,77 41,68 0,17 149,67
23,11 6,78 291,45 84,42 12,95 175,04 380,19 92,17 21749,52
5,81 0,58 1988,93 23738,45 6,34 2081,09 6,85 10,61 14,50
16,63 8,77 42,65 100,00
SEARO India Asia Southern Asia 507486100,00
365100619,00 42,60 25,12 32,28 155532863,69 91713275,49
117854479,81 38261,08 6419,93 5657,02
50338,03 45761844,35 83896714,64 64829279,49 64829279,00
380342,00 430680,03 239773,39 11733481,28 5083754,29
60640,27 4343141,23 1276372,95 179133,12 7390340,05
3807381,34 1,06 1,34 2622984,00 365100619,00 50338,03
50338,03 13,79 380342,00 104,17 64829279,49 50,57 20,00
2545751,60 7607469,30 9143500,21 11241931,20 4866909,87
11241931,20 19296721,11 21395152,11 15020130,78 21395152,11
5285,32 138632,99 5,29 2,78 65689,44 2,50 18,68 81,32
7184,28 14713,08 380325,91 4507,29 375818,61 25,54 67752,45
1730611,82 20755,68 1751367,50 0,48 12,58 1811807,95
0,50 13,02 131909,32 382616,39 393386,87 31497,97
155572,62 175564,54 53685,79 218458,81 339066,68
21990,00 131129,06 182069,95 891246,76 598975,87
814539,57 552850,48 0,91 0,92 814539,57 552850,48
117293,70 37040,98 154334,68 6406262,89 11346617,67
5686879,71 1751688,69 4705237,53 2657949,04 2675552,63
6389349,70 4729184,05 1356543,27 4013734,82 2685186,67
26991416,36 16906637,32 24668338,46 15604706,36 3552240,74
1045515,33 4597756,06 1,26 1,19 1751367,50 1811807,95
4597756,06 4343141,23 8891079,37 9,08 9,39 23,83 22,51
35,20 67752,45 141419,23 154334,68 50338,03
417764,57 158254,87 16,22 33,85 12,05 37,88 0,17
246474,51 44746,34 11202,20 724142,57 298401,62
51518,23 294954,92 1039716,93 221555,53 21616707,64

5,92 0,58 2657503,01 24274210,64 6,65 2879058,53 7,21
7,46 18,94 17,89 11,86 36,63 100,00
SEARO Indonesia Asia South-Eastern Asia
125165200,00 129589805,00 28,50 22,36 49,14 36933094,43
28976280,40 63680430,18 9085,54 2028,34
3056,66 14170,54 12882310,46 23617569,17 18249939,82
18249940,00 135000,00 149170,54 42224,99 3282350,38
1761463,63 21242,44 1528621,81 498366,34 20982,55
1753728,58 1263097,29 2,57 1,34 1058424,00 129589805,00
14170,54 14170,54 10,93 135000,00 104,17 18249939,82
48,50 23,38 687269,50 3156680,65 8459115,85 4557589,59
1595550,74 4557589,59 12303066,00 8401539,73 5439500,89
8401539,73 9493,85 100485,22 9,49 2,97 69089,78 6,53
8,29 91,71 8167,49 5003,37 139300,54 1318,23 137982,31 27,58
24048,24 663199,27 6335,95 669535,22 0,52 5,47
868323,20 0,67 7,09 21079,25 64641,26 53847,15
15061,07 72352,76 73325,74 10444,88 35242,36
60089,06 12047,32 56305,50 101857,84 215525,31
168022,35 200896,16 136353,77 0,93 0,81 200896,16
136353,77 28929,05 9135,70 38064,75 1022813,64
1952143,67 792270,79 831731,34 2298498,39 1128553,81
508938,67 1045422,37 818604,65 713282,69 1883177,42
1503516,31 6745461,91 4924861,47 6287602,22 3996631,47
905414,72 267774,31 1173189,03 0,91 1,18 669535,22
868323,20 1173189,03 1528621,81 4161870,48 5,44 7,06
9,54 12,42 65,54 24048,24 50195,72 38064,75 14170,54
161301,52 72887,01 14,91 31,12 8,79 45,19 0,17 22887,92
3591,75 478,56 45971,15 11352,17 1398,66 26639,19 57789,54
14015,17 8415554,90 6,49 0,58 678103,26 9093658,16
7,02 692118,43 7,36 9,55 12,90 16,81 7,61 45,77 100,00
SEARO Korea, Dem. People's Rep. Of Asia Eastern Asia
15030100,00 16133714,00 43,82 12,73 43,45 7069793,47
2053821,79 7010098,73 1739,17 143,77
336,48 2219,42 2017655,87 3699035,77 2858345,82 2858346,00
16807,00 19026,42 7889,96 351011,74 141558,12 1780,09
97086,57 10104,37 6109,88 253925,16 131453,75 1,07
1,34 16133714,00 2219,42 2219,42 13,76 16807,00 104,17
2858345,82 48,86 20,04 108449,81 336893,43 374200,14
463521,40 144051,92 463521,40 819543,38 908864,64
589395,17 908864,64 5079,69 0,00 5,08 2,76 x 2,32
14,77 85,23 x 962,74 26959,56 261,78 26697,78 27,73
2993,97 83025,77 814,09 83839,86 76742,70
0,48 2087,11 7216,45 7516,51 2245,67 11280,66 11600,35
765,51 3843,51 14369,13 1312,75 8157,53 20846,18 29202,19
25817,73 24124,03 16373,65 0,83 0,63 24124,03
16373,65 3473,86 1097,03 4570,90 102792,26 218822,20
111109,12 114639,46 353619,19 183754,97 37986,43
114364,92 182043,27 70610,07 264645,95 308751,99
888161,14 651205,80 733713,19 412995,79 105654,70
27670,72 133325,42 0,83 0,60 83839,86 76742,70
133325,42 97086,57 517870,09 10,23 9,36 16,27 11,85
52,29 2993,97 6249,28 4570,90 2219,42 20704,81 9242,14 14,46 30,18
10,72 44,64 0,17 1571,89 150,34 33,58 5143,73 2299,05 560,20 1733,42
7629,50 1554,25 910418,89 5,64 0,58 77062,09 987480,98
6,12 78616,34 8,49 7,77 13,50 9,83 7,96 52,44 100,00
SEARO Maldives Asia Southern Asia 171261,20

280618,00 8,32 19,13 72,55 23347,42 53682,22
203588,36 5,74 3,76 9,77 19,27
17521,33 32122,44 24821,88 24822,00 292,00 311,27
45,50 4145,54 2360,37 27,78 2139,64 679,98 17,72 2005,90 1680,39 5,16
1,34 4030,00 280618,00 19,27 19,27 6,87 292,00 104,06 24821,88
52,54 18,37 1012,64 5362,97 28156,53 7649,93 2152,13 7649,93 34532,14
14025,54 8527,74 14025,54 12305,75 495,92 12,31 2,27
404,36 10,03 4,30 95,70 14361,16 5,23 119,16 1,52 117,64
22,49 52,07 1171,34 15,13 1186,47 0,42 0,02 1033,61 0,37 0,01
29,73 61,85 116,30 13,46 25,83 60,97 3,60 20,81 75,56 2,84
10,58 40,64 189,96 76,56 274,88 186,57 1,45 2,44 274,88 186,57
39,58 12,50 52,08 1491,63 1905,58 1491,03 878,95 909,02 815,64 180,41
610,17 930,04 267,31 432,92 570,94 5954,07 1991,14 8615,89 4852,11 1240,69
325,09 1565,78 0,56 0,76 1186,47 1033,61 1565,78 2139,64 8100,05 3,44
2,99 4,53 6,20 82,84 52,07 108,70 52,08 19,27 367,89 187,84
14,16 29,55 5,24 51,06 0,17 106,54 7,09 1,31 119,48 20,39
2,98 114,07 140,87 42,32 14067,86 5,01 0,58 905,02 14972,88
5,34 947,34 7,92 6,90 10,46 14,29 6,33 54,10 100,00
SEARO Myanmar (Burma) Asia South-Eastern Asia
29549730,00 22039745,00 48,85 16,94 34,21 10766415,43
3733532,80 7539796,76 2648,54 261,35
361,91 3271,80 2974359,76 5452992,90 4213676,33 4213676,00
22960,00 26231,80 7174,07 449034,88 245726,69 1419,50
98249,50 24869,53 5754,57 350785,38 220857,16 1,70
1,34 76186,00 22039745,00 3271,80 3271,80 14,84 22960,00
104,18 4213676,33 51,69 22,58 169133,52 518396,69
938513,10 751182,47 278178,94 751182,47 1626043,31
1438712,69 965709,16 1438712,69 7377,78 5620,83 7,38 3,12
3244,21 4,26 13,93 86,07 3456,76 1043,80 28592,35 273,22 28319,13
27,13 4089,96 110963,87 1070,57 112034,44 0,51 0,39
138604,59 0,63 0,48 2364,40 9761,22 9020,83 4210,70 16511,20
18629,45 1170,25 5276,38 12990,12 2927,90 12483,73 27162,15
42064,29 35242,35 47428,74 32191,19 1,13 0,91
47428,74 32191,19 6829,74 2156,81 8986,55 117977,64
291203,48 128657,60 224812,29 508532,99 273946,69
59152,55 152363,30 165699,30 157940,00 377794,81
364397,30 1276727,83 923949,52 1439548,69 843957,17
207295,01 56545,13 263840,14 1,20 0,45 112034,44
138604,59 263840,14 98249,50 825984,03 6,89 8,52
16,23 6,04 62,32 4089,96 8536,94 8986,55 3271,80 25782,19 9883,49
15,86 33,11 12,69 38,33 0,17 2373,77 510,73 85,96 4853,38 1630,73
239,19 2913,15 6563,84 1573,18 1440285,87 6,53 0,58 152499,60
1592785,47 7,23 154072,78 7,03 8,70 16,56 6,17 9,67
51,86 100,00
SEARO Nepal Asia Southern Asia 17915100,00
7086194,00 64,38 15,11 20,52 4562091,70 1070723,91
1454087,01 1122,27 74,95 69,80 1267,02
1151837,64 2111702,35 1631769,99 1631770,00 7382,00 8649,02
10365,87 493545,02 220990,75 2061,77 87760,00 46544,26
8304,10 405785,02 174446,49 0,75 1,34 33657,00
7086194,00 1267,02 1267,02 17,88 7382,00 104,17 1631769,99 19,99
27,86 25328,33 205650,37 183678,57 303860,61
203049,36 303860,61 414657,26 534839,31 434028,05
534839,31 5851,62 1969,48 5,85 3,26 872,41 2,59 13,01 86,99
4749,66 297,45 7563,54 89,61 7473,92 25,13 1315,00 33041,51 396,16
33437,67 0,47 0,16 121843,13 1,72 0,58 1455,90 6560,25

7841,83 454,89 3428,72 4846,44 736,55 2805,89 4342,16 395,09 2224,87 3818,84
16129,19 8882,74 28754,60 19516,54 1,78 2,20 28754,60
19516,54 4140,66 1307,61 5448,27 71433,47 191202,21
113453,43 25583,94 100441,79 71872,40 35900,95
81974,30 60852,93 23651,63 66941,49 55515,70
450436,68 247257,92 803024,14 543258,15 115635,48
36398,30 152033,77 2,15 1,24 33437,67 121843,13
152033,77 87760,00 139764,73 8,06 29,38 36,66 21,16
4,72 1315,00 2744,79 5448,27 1267,02 5945,54 618,73 22,12 46,17 21,31
10,41 0,17 6932,21 1120,10 307,59 23502,26 7863,98 1232,29 8154,84
31777,00 6628,69 541467,99 7,64 0,58 87875,52
629343,51 8,88 94504,21 5,31 19,36 24,16 13,94 15,02
22,21 100,00
SEARO Sri Lanka Asia Southern Asia 8940520,00
8180693,00 24,98 27,87 47,15 2043537,11 2279959,14
3857196,75 80,00 1461,00 502,71 159,60 185,15 847,45
770411,56 1412421,19 1091416,37 1091416,00 8522,00 9369,45
1937,76 147806,64 90025,65 577,69 35983,60 8501,73 1360,07
111823,04 81523,93 2,69 1,34 80707,00 8180693,00
847,45 847,45 10,36 8522,00 104,17 1091416,37 47,57 22,28 40314,88
189850,49 523290,58 266581,72 93995,64 266581,72
753455,94 496747,09 324161,00 496747,09 9210,17 7433,25
9,21 2,81 5162,55 6,40 7,01 92,99 9865,55 314,10 8333,82 100,69
8233,14 26,21 1518,11 39792,39 486,65 40279,05 0,49 0,40
51005,37 0,62 0,50 1066,59 5023,77 7169,00 422,90 2230,14 4504,95
281,61 2441,88 7985,90 196,37 1284,17 5711,11 12634,72 8769,70 14349,96
9739,72 1,14 1,11 14349,96 9739,72 2066,39 652,56 2718,96 51584,67
147782,78 102391,99 25590,81 74013,62 67082,31
14123,34 68880,68 104552,07 16612,55 49580,31
82408,12 355463,32 211516,96 403719,69 234912,82
58135,64 15739,16 73874,79 0,90 0,44 40279,05
51005,37 73874,79 35983,60 295604,27 5,35 6,77
9,80 4,78 73,30 1518,11 3168,74 2718,96 847,45 9819,23 4284,94 15,46
32,27 8,63 43,64 0,17 3310,40 573,04 114,68 4271,32 1137,08 233,17
3921,66 5486,12 1561,69 498308,78 6,09 0,58 42699,63
541008,41 6,61 44261,32 7,45 9,43 13,66 6,65 8,18
54,64 100,00
SEARO Thailand Asia South-Eastern Asia
40735640,00 37613439,00 31,43 22,84 45,73 11821903,88
8590909,47 17200625,65 94267,00 2908,19
601,36 825,63 4335,18 3941074,59 7225303,41 5583189,00
5583189,00 39184,00 43519,18 10159,41 776491,89
458958,17 4145,50 240292,27 46809,33 6013,91 536199,63
412148,83 3,32 1,34 501745,00 37613439,00 4335,18 4335,18
11,53 39184,00 104,18 5583189,00 46,67 20,63 202335,94
808261,07 2734333,74 1130787,86 461099,97 1130787,86
3744930,75 2141384,87 1471696,98 2141384,87 9956,36 49955,56
9,96 2,69 36474,68 7,27 6,71 93,29 13339,51 2278,36
57870,80 638,40 57232,40 25,12 6980,00 175337,60 1955,81
177293,41 0,47 2,37 266431,23 0,71 3,55 3010,35 9137,41
16401,40 3835,63 9524,99 13971,51 675,04 4327,22 18044,04
1142,69 5023,06 17478,56 35632,69 23008,88 65382,66
44377,01 1,83 1,93 65382,66 44377,01 9415,10 2973,26
12388,36 148175,26 283924,74 213544,34 207217,82
331048,20 205831,80 33625,19 130863,78 215684,25
77129,59 199287,40 247502,03 1110158,07 595301,38

2037036,65 1148152,15 293333,28 76926,19 370259,47
0,98 0,64 177293,41 266431,23 370259,47 240292,27
1087108,50 4,73 7,11 9,89 6,42 71,85 6980,00 14569,31
12388,36 4335,18 45700,13 19815,64 15,27 31,88 9,49
43,36 0,17 4992,45 1079,55 241,08 9879,19 4856,40 859,32 6152,36 15022,03
3514,95 2144899,82 5,70 0,58 214009,97 2358909,80 6,27
217524,92 7,52 11,29 15,70 10,19 9,22 46,09 100,00
SEARO Timor-Leste Asia South-Eastern Asia
521688,00 521688,00 39,28 16,32 44,40 204919,05 85139,48
231629,47 50,41 5,96 11,12 67,49
61352,79 112480,11 86916,45 86916,00 543,00 610,49
132,53 8503,20 4354,46 45,60 3144,35 870,08 86,93 5358,84 3484,38 1,86
1,34 1821,00 521688,00 67,49 67,49 12,94 543,00 104,09 86916,45
49,88 21,56 3366,13 11708,48 23052,33 16959,30 4772,18
16959,30 38126,95 32033,92 19846,79 32033,92
7308,38 133,09 7,31 2,89 80,47 4,42 12,21 87,79 3490,59 15,23
406,92 3,89 403,03 26,46 96,81 2561,89 24,73 2586,62
2257,08 0,43 53,79 230,33 300,88 49,00 230,55 305,36 23,88
143,00 268,97 37,06 190,78 316,31 765,74 589,82 837,33 568,32 1,09
0,96 837,33 568,32 120,58 38,08 158,65 2682,33 6630,30 4316,60 2797,99
7121,75 4724,20 1192,68 3960,35 3776,76 2210,89 5997,19 4976,17 22245,96
16278,75 24325,99 15685,51 3502,94 1050,93 4553,87
0,60 2257,08 4553,87 3144,35 x x x x x
x 202,07 67,49 812,56 543,00 0,00 24,87 8,31
66,83 0,17 152,85 15,82 3,45 337,73 62,23 11,43 169,82 403,77
95,22 32129,13 6,16 0,58 2632,14 34761,27 6,66 2727,36
0,00 6,49 13,10 9,05 7,85 x x
SEARO TOTAL 825018295,10
647748638,00 252463843,98 151563784,34
243721680,84 80,00 95728,00 0,00 0,00 62106,11 10609,46
11698,64 84414,21 76740192,00 140690352,01 249405624,01
674789,00 759203,21 338915,23 18721167,87 8855844,31
99961,37 7170492,13 2244575,17 238953,86 11550675,74
6611269,14 1,34 1,34 4706202,00 647748638,00 84414,21
84414,21 13,03 674789,00 104,17 108715272,01 49,28 20,67
4178413,49 14125394,07 20810440,83 20810440,83 8490377,14
20810440,83 39114248,39 39114248,39 26794184,70 39114248,39
6038,49 284183,62 6,04 2,83 151197,75 3,21 15,55 84,45
7265,48 120204,00
3173924,41 0,49 23,06 3663204,55 0,57 26,61

1351395,35 477040,50 1324194,00 898767,00 0,98 1,88
1324194,00 898767,00 190683,94 60217,39 250901,33
8311316,65 15452651,41 7672822,49 3583626,54 9236022,49
5295907,40 3584391,69 8516316,29 6708190,58 2648553,12
7505017,03 5884579,25 40906527,06 26451868,07 40083146,44
49836577,80 5771973,09 3339050,71 9111023,80 1,41 1,11
3171337,79 3663204,55 7449206,58 7170492,13 17635152,43
120107,19 250901,00 250742,67
84414,21 759361,54 303939,14 15,82 33,04 11,12 40,03
0,17 319058,80 56917,58 14453,91 962164,83
367847,15 62669,63 380794,35 1350901,86 287461,57
39401709,96 6,08 0,58 5266171,76 44667881,72 6,90
5578488,23 7,10 8,20 16,68 16,05 12,49 39,48 100,00

WPRO	American Samoa		Oceania	Polynesia				17850,00	
17850,00		0,00	13,10	86,90	0,00	2338,35	15511,65		
		0,00	0,25	1,07	1,32	1200,46	2200,85	1700,65	1701,00
14,00	16,00	5,22	296,49	149,25	1,09	68,97	13,34	4,13	227,52
135,91	1,48	1,40	638,00	17850,00		1,32	1,32	7,40	14,00
78,43	1700,65	51,26	22,18	67,69	310,47	476,81	451,88	152,14	451,88
854,97	830,04	530,30	830,04	4789,75	30,56	4,79	2,12	17,04	2,67
9,82	90,18	35742,30		1,64	40,15	0,40	39,75	24,24	5,63
136,56	1,37	137,94			82,76	0,46		5,68	18,62
14,09	2,44	8,23	9,95	1,72	8,61	15,76	1,75	6,37	12,25
42,98	27,78	26,09	16,69	0,61	0,60	x	x	x	x
268,00	570,85	205,87	138,34	275,47	155,28	81,77	259,27	202,52	111,88
229,91	185,04	1373,05	812,02	x	x	x	x		
0,39	137,94	82,76	0,00	68,97	x	x	x	x	x
x	5,63	4,98		1,32	20,30	8,37	27,76	24,52	6,51
41,21	0,17	5,43	0,46	0,08	15,26	4,37	0,69	5,91	19,86
4,28	834,32	4,67	0,58		834,32	4,67	4,28	16,53	9,92
0,00	8,27	0,51	x	x					
WPRO	Cambodia		Asia	South-Eastern Asia					
8641671,00		9101786,00		34,53	27,91	37,56	3142846,71		
2540308,47		3418630,82						562,57	271,81
235,89	1070,27	972970,99		1783780,16		1378375,58		1378376,00	
7261,00	8331,27	2388,75	193553,86		105728,07		1109,52	73013,17	
14438,45		1279,23	120540,69		91289,62		3,12	1,40	25291,00
9101786,00		1070,27	1070,27	11,76	7261,00	79,78	1378375,58		52,79
22,87	56502,64		166030,49		532092,04		246899,47		
105217,31		246899,47		754625,18		469432,60		327750,44	
469432,60		8290,96	2096,87	8,29	2,44	1478,52	5,85	9,33	90,67
2778,69	391,71	10587,95		101,57	10486,38		26,77	2872,93	76910,47
744,95	77655,42		0,85	0,22	64333,21		0,71	0,18	690,13
2684,80	2842,91	524,62	2603,28	3072,77	298,75	1657,21	3282,35	475,65	2712,79
5293,70	8474,72	8003,08	12631,05		8078,90	1,49	1,01	12631,05	
8078,90	1818,87	541,29	2360,16	33996,73		80657,96		40627,97	
29023,56		82348,05		46298,31		14785,88		47937,28	
44077,66		27719,77		87240,02		76693,82		255001,73	
217940,12		380064,25		220004,74		54729,25		14740,32	
69469,57		0,76	0,80	77655,42		64333,21		69469,57	
73013,17		184961,22		10,29	8,53	9,21	9,68	62,30	2872,93
2538,09	2360,16	1070,27	8509,20	2027,92	33,76	29,83	12,58	23,83	0,17
1539,42	228,82	35,62	2727,57	677,08	99,34	1780,11	3437,76	866,17	
470298,76		5,17	0,58	40153,41		510452,17		5,61	41019,58
15,21	12,60	13,61	14,30	8,04	36,23	100,00			

WPRO China Asia Eastern Asia 820726500,00
767180569,00 25,33 27,42 47,25 194326838,13 210360912,02
362492818,85 34784,50 22508,62
25012,00 82305,13 74822841,92 137175210,19 105999026,05
105999026,00 612040,00 694345,13 341081,71 15486849,04
7538711,78 61779,88 3966241,54 1021199,60 279301,83
11520607,50 6517512,18 1,30 1,40 14722731,00 767180569,00
82305,13 82305,13 10,73 612040,00 79,78 105999026,05
47,67 17,91 3923479,04 10963388,26 15642436,62 16744155,10
7877986,94 16744155,10 30529303,93 31631022,41 22764854,25
31631022,41 3979,42 585878,67 3,98 1,94 300189,29 2,04
17,31 82,69 19190,70 67264,36 1639492,03 21436,57
1618055,46 24,06 242156,17 5825107,18 77173,08
5902280,26 0,77 113,27 2859879,64 0,37 54,88 68767,00
282802,74 673985,81 63373,34 362417,71 834885,69
19375,86 141259,16 687816,34 22432,88 197281,71
708093,81 1280317,95 845652,98 1199610,13 767278,09
0,94 0,91 1199610,13 767278,09 172743,86 51407,63
224151,49 3402727,96 8545109,63 9097043,47 3356310,30
11391892,62 12305509,79 1014906,28 4285438,01 8340580,73
1485386,61 6883030,42 10433248,47 33830224,94 19926704,38
31697657,82 18079902,70 4564462,73 1211353,48 5775816,21
0,75 0,52 5902280,26 2859879,64 5775816,21 3966241,54
13126804,76 19,33 9,37 18,92 12,99 39,39 242156,17
213932,98 224151,49 82305,13 684126,62 145732,34
35,40 31,27 12,03 21,30 0,17 66216,34 20291,69 5774,12
274743,02 191165,23 37477,25 88432,74 478400,66
94094,35 31725116,76 4,14 0,58 3338421,77 35063538,52
4,57 3432516,11 16,83 8,16 16,47 11,31 9,79 37,44 100,00
WPRO Cook Islands Oceania Polynesia 6820,00 7954,00
29,00 15,00 56,00 2306,66 1193,10 4454,24
0,41 0,13 0,31 0,85 770,81 1413,16 1091,99 1092,00 6,00 6,85
1,87 128,53 78,74 0,39 25,24 6,77 1,48 103,28 71,97 2,30
1,40 7954,00 0,85 0,85 10,66 6,00 75,43 1091,99 47,00
21,20 39,85 127,22 306,98 193,11 86,56 193,11 474,06 360,19 253,64
360,19 5960,01 0,00 5,96 2,10 0,00 3,86 11,49 88,51 0,00
0,76 18,04 0,18 17,85 23,49 2,51 58,97 0,59 59,56
42,91 0,54 0,00 1,57 6,83 7,18 0,60 2,83 4,60 0,30
1,91 6,53 0,33 1,46 4,82 15,75 7,78 9,97 6,38 0,63
0,82 x x x x 73,89 205,74 97,10 35,40
99,41 71,07 14,58 57,16 80,23 24,02 59,78 74,30 470,50 207,05
x x x x 59,56 42,91 0,00
25,24 x x x x x x 2,51 2,22
0,85 9,07 3,49 27,69 24,47 9,35 38,49 0,17 1,80 0,31
0,07 6,88 2,66 0,53 2,13 9,72 1,97 362,15 4,55 0,58
362,15 4,55 1,97 16,45 11,85 0,00 6,97 0,54 x x
WPRO Fiji Oceania Malenesia 379017,00
347277,00 17,61 14,14 68,26 61155,48 49104,97
237051,28 10,95 5,25 16,36 32,56
29597,82 54262,67 41930,24 41930,00 277,00 309,56
71,40 4776,68 2284,21 25,06 1564,90 314,37 46,34 3211,78 1969,84 1,59
1,40 4376,00 347277,00 32,56 32,56 9,38 277,00 79,76 41930,24
49,90 26,80 1624,60 7423,91 12183,45 10825,54 2378,25 10825,54
21231,96 19874,05 11426,76 19874,05 6113,84 267,54
6,11 2,61 153,52 3,51 9,58 90,42 12600,89 11,56 312,18
3,36 308,82 26,71 109,62 2928,34 31,86 2960,20 0,85 0,04 1185,48

0,34	0,01	174,60	564,22	314,28	35,57	124,85	124,37	38,18	207,70
275,65	35,17	129,09	172,35	1045,47	559,47	553,99	354,33	0,53	0,63
553,99	354,33	79,77	23,74	103,51	8250,19	17210,60		4850,72	2105,99
4149,64	2042,60	1838,82	6112,56	3930,00	2171,91	4403,68	2816,65	34014,19	
16775,86		18023,94		10624,73		2595,45	711,86	3307,30	0,95
0,45	2960,20	1185,48	3307,30	1564,90	10856,17		13,94	5,58	15,58
7,37	57,53	109,62	96,84	103,51	32,56	302,88	63,87	36,19	31,97
10,75	21,09	0,17	123,85	9,15	1,12	314,84	83,82	12,17	133,37
402,71	88,99	19963,04		5,75	0,58	1911,62	21874,66		6,30
2000,61	13,53	5,42	15,12	7,15	9,15	49,63	100,00		
WPRO	French Polynesia			Oceania	Polynesia				
123365,20		102373,00		6,83	16,64	76,54	6992,08	17034,87	
78356,29						1,25	1,82	5,41	8,48
7709,91	14134,83		10922,37		10922,00		82,00	90,48	
								1,40	3448,00
102373,00		8,48	8,48	8,28	82,00	80,10	10922,37		
0,00	0,00				0,00	0,00	0,00	0,00	0,00
0,00	0,00	0,00	0,00	0,00	0,00			33680,76	
x	x	x	x	x	32,31	x	x		
			x	x	x	x	x	x	x
x	x	x	x	x	x	x	180,32	115,33	
x	x	x	x		x	x	x	x	x
x	x	x	x	x	x	x	x	x	x
x	x	x				0,00	0,00	0,00	x
x	x	x	x	x	x	32,31	28,55		8,48
119,03	49,69	27,15	23,98	7,13	41,74	0,17			
		0,00	0,00	0,00	0,00	0,00	0,58		0,00
0,00	0,00	x	x	x	x	x	x	x	
WPRO	Guam	Oceania	Micronesia				84082,48		76703,00
0,23	15,54	84,23	176,42	11919,65		64606,94			
	0,03	1,28	4,46	5,76	5240,78	9608,10	7424,44	7424,00	61,00
66,76	12,32	813,05	466,85	2,56	176,02	47,13	9,76	637,03	419,72
1,93	1,40	6311,00	76703,00		5,76	5,76	7,52	61,00	79,53
7424,44	50,33	22,27	290,12	1358,49	2729,83	2012,31	525,81	2012,31	4378,44
3660,92	2174,42	3660,92	5708,30	360,25	5,71	2,15	224,61	3,56	9,05
90,95	82278,40		5,67	138,66	1,38	137,28	24,21	24,21	586,18
5,89	592,08	0,77	0,05	249,09	0,32		19,79	82,59	82,42
6,13	18,83	23,50	6,85	39,03	89,71	3,78	13,04	27,41	162,65
101,73	122,90	78,61	0,76	0,77	122,90	78,61	17,70	5,27	22,96
954,38	2489,47	1096,82	368,05	698,50	383,20	325,26	1147,66	1075,74	261,78
542,69	457,45	5003,74	2788,46	3780,93	2154,58	544,45	144,36	688,81	0,90
0,23	592,08	249,09	688,81	176,02	1954,93	13,52	5,69	15,73	4,02
61,04	24,21	21,39	22,96	5,76	65,19	13,83	37,14	32,81	8,84
21,21	0,17	30,43	2,72	0,52	55,51	18,21	4,14	33,32	75,10
18,00	3678,92	4,80	0,58	398,13	4077,05	5,32	416,13	14,52	6,11
16,89	4,32	10,21	47,95	100,00					
WPRO	Hong Kong, China			Asia	Eastern	Asia			
3864082,00		3851142,00		0,17	11,08	88,75	6546,94	426706,53	
3417888,53						1,17	45,66	235,83	282,66
256967,10		471106,35		364036,72		364037,00		3072,00	3354,66
									1,40
346586,00		3851142,00		282,66	282,66	7,34	3072,00	79,77	
364036,72				0,00	0,00				0,00
0,00	0,00	0,00	0,00	0,00	0,00	0,00	0,00	0,00	0,00
	89995,64		x	x	x	x	x	1215,59	x
x							x	x	x

x x x x x x x x x x
x 5647,91 3612,44 x x x x
x x x x x x x x x x
x x x x x x x x
0,00 0,00 0,00 0,00 x x x x x x
1215,59 1073,91 282,66 4428,58 1856,41 27,45 24,25 6,38 41,92
0,17 0,00 0,00 0,00
0,00 0,00 0,58 0,00 0,00 0,00 x x x
x x x x
WPRO Kiribati Oceania Micronesia 39000,00
39000,00 15,00 10,00 75,00 5850,00 3900,00 29250,00
1,05 0,42 2,02 3,48 3166,09 5804,50 4485,30 4458,00
31,00 35,00 9,44 666,58 273,17 4,32 268,98 42,17 5,12 397,60
231,00 1,39 1,40 200,00 39000,00 3,48 3,48 8,93 31,00
79,49 4485,30 52,46 32,55 182,72 1009,07 1433,09 1449,88 264,97 1449,88
2624,87 2641,66 1456,75 2641,66 6730,43 13,46 6,73 3,06 7,35 3,67
8,26 91,74 5128,21 0,86 27,47 0,25 27,22 31,65 12,31 389,63
3,58 393,21 1,01 0,00 162,90 0,42 0,00 29,66 55,28 11,70
25,45 42,06 18,64 9,66 29,00 22,31 11,44 27,29 20,42 162,55
91,63 57,00 36,46 0,35 0,40 57,00 36,46 8,21 2,44 10,65
1445,88 1742,04 187,47 1366,76 1362,64 303,40 464,81 856,94 336,48 648,75
876,38 337,43 6080,95 3071,52 2132,44 1222,13 307,07 81,88 388,95 1,00
0,69 393,21 162,90 388,95 268,98 1427,62 14,98 6,21 14,82 10,25
53,75 12,31 10,88 10,65 3,48 34,71 8,04 35,47 31,34 10,03
23,16 0,17 14,32 0,93 0,08 47,29 10,58 1,28 15,28 58,30
12,21 2653,87 6,80 0,58 224,82 2878,69 7,38 237,03 13,66 5,66
13,51 9,34 8,23 49,59 100,00
WPRO Lao People's Dem. Rep. Asia South-Eastern Asia
3494100,00 1757733,00 61,44 12,94 25,62 1079951,16
227450,65 450331,19 193,31 24,34
31,07 248,72 226110,30 414535,55 320322,92 320323,00
1402,00 1650,72 959,14 78037,81 42511,28 418,81 28189,80
6134,64 540,32 49848,00 36376,64 2,70 1,40 19136,00
1757733,00 248,72 248,72 14,15 1402,00 79,76 320322,92 52,66
24,93 13097,92 34954,72 98031,18 52691,19 40019,82
52691,19 146083,83 100743,83 88072,47 100743,83
8310,92 1590,38 8,31 2,73 1067,24 5,58 11,46 88,54 10886,75
102,03 3009,87 27,61 2982,26 29,23 554,82 16216,92 150,14 16367,06
0,93 0,18 25916,03 1,47 0,28 471,81 1364,81 1353,06 397,69
1430,22 1435,28 217,48 807,47 1460,18 295,13 1000,81 1603,74 4593,97 3342,20
5107,13 3266,55 1,11 0,98 5107,13 3266,55 735,43 218,86 954,29 23349,81
40702,26 19235,68 21594,79 45033,99 21940,61
10825,88 23448,10 19108,05 17028,29 32967,60
23545,04 144406,28 98487,57 160536,83 96258,46
23117,30 6449,32 29566,62 1,68 1,60 16367,06 25916,03
29566,62 28189,80 704,32 11,20 17,74 20,24 19,30 31,52
554,82 490,15 954,29 248,72 1186,59 -107,10 46,76 41,31 20,96 -9,03
0,17 1082,72 98,32 15,29 1182,60 170,02 21,82 1186,13 1359,90 422,64
101166,48 5,76 0,58 17089,51 118255,98 6,73 17512,15
13,84 21,92 25,00 23,84 14,81 0,60 100,00
WPRO Macau, China Asia Eastern Asia 372797,00
387800,00 0,40 9,66 89,94 1551,20 37461,48 348787,32
0,28 4,01 24,07 28,35 25774,88 47253,95
36514,41 36514,00 309,00 338,00
1,40 55154,00 387800,00
28,35 28,35 7,31 309,00 79,68 36514,41 0,00

0,00				0,00	0,00	0,00	0,00	0,00	0,00
0,00	0,00	0,00	0,00	0,00			142222,80		x
x	x	x	x	122,41	x	x
		x	x	x	x	x	x	x	x
x	x	x	x	x	x	544,90	348,52
x	x	x	x		x	x	x	x	x
x	x	x	x	x	x	x	x	x	x
x	x	x				0,00	0,00	0,00	0,00
x	x	x	x	x	x	122,41	108,14		28,35
445,49	186,59	27,48	24,27	6,36	41,88	0,17
		0,00	0,00	0,00	0,00	0,00	0,58		0,00
0,00	0,00	x	x	x	x	x	x	x
WPRO	Malaysia		Asia	South-Eastern Asia
12969470,00	15258418,00	10,28	27,01	62,72	1568565,37
4121298,70	9570079,77					280,77	440,98
660,34	1382,09	1256443,33	2303479,44	1779961,39	1779961,00
12173,00	13555,09	2751,42	211648,97	118621,35	1120,55
71713,02	14724,60	1630,87	139935,95	103896,75	2,88
1,40	336664,00	15258418,00	1382,09	1382,09	9,06	12173,00
79,78	1779961,39	50,86	22,10	70289,28	269001,07
793659,31	395619,97	122058,01	395619,97	1132949,65
734910,32	461348,35	734910,32	7425,08	24997,57	7,43
2,22	17511,42	5,20	7,81	92,19	22064,15	537,42	14490,40
156,88	14333,52	26,67	4816,23	128453,66	1405,92	129859,58
0,85	2,87	62971,93	0,41	1,39	2430,95	9261,92	9637,70	1028,76
3870,88	5621,09	432,27	3879,06	8368,58	468,09	2697,75	6209,76	21678,77
12336,61	18956,75	12124,86	0,87	0,98	18956,75
12124,86	2729,77	812,37	3542,14	117938,90	274469,88
143758,58	62125,32	128957,44	88589,09	21370,52
110440,30	117371,93	35101,89	96900,69	96782,91
660940,76	335198,34	577952,09	329444,74	83225,10
22072,80	105297,90	0,69	0,47	129859,58	62971,93
105297,90	71713,02	365067,89	11,46	5,56	9,29	6,33
67,36	4816,23	4254,90	3542,14	1382,09	14267,85	3814,63	33,76	29,82
9,69	26,74	0,17	7122,52	1233,49	250,26	10018,48	2365,99	348,79
8439,43	12500,73	3476,07	738386,38	4,84	0,58	60862,19
799248,57	5,24	64338,25	16,25	7,88	13,17	8,97	8,05
45,68	100,00
WPRO	Marshall Islands	Oceania	Micronesia	10670,00
10670,00	11,00	16,30	72,70	1173,70	1739,21	7757,09
	0,21	0,19	0,54	0,93	846,75	1552,38	1199,57	1200,00	9,00
9,00	5,18	309,15	120,96	1,80	106,31	12,73	3,38	202,84	108,23
1,14	1,40	239,00	10670,00	0,93	0,93	8,73	9,00	84,35
1199,57	51,97	27,97	48,41	251,72	294,56	359,79	114,82	359,79	594,69
659,92	414,95	659,92	5573,46	13,32	5,57	2,81	6,60	2,76	9,25
90,75	22399,25	1,40	38,77	0,36	38,41	27,44	3,37	92,40
0,87	93,27	0,87	0,00	68,87	0,65	0,00	14,46	26,80	11,01
6,85	13,06	8,19	4,77	13,50	9,78	4,48	10,37	7,17	67,57
38,77	15,60	9,98	0,23	0,26	15,60	9,98	2,25	0,67	2,91
695,35	818,78	171,74	369,20	420,01	134,19	226,60	402,01	147,45	254,07
345,30	119,91	2405,32	1317,10	555,14	338,90	79,94	22,71	102,65	0,96
1,00	93,27	68,87	102,65	106,31	288,82	15,68	11,58	17,26	17,88
37,60	3,37	2,98	2,91	0,93	9,99	2,72	33,70	29,78	9,32
27,20	0,17	7,03	0,46	0,05	15,53	2,81	0,25	7,51	18,42
4,30	664,23	6,23	0,58	59,33	723,55	6,78	63,63	12,89	9,52
14,19	14,69	8,79	39,92	100,00

WPRO	Micronesia		Oceania	Micronesia				37920,00	
37929,00		0,90	5,20	93,90	341,36	1972,31	35615,33		
		0,06	0,21	2,46	2,73	2481,45	4549,33	3515,39	3515,00
30,00	32,73	12,90	741,09	302,70	3,66	215,95	30,97	9,24	525,13
271,73	1,07	1,40	408,00	37929,00		2,73	2,73	7,20	30,00
79,10	3515,39	50,55	27,42	137,97	822,55	905,12	1177,29	294,83	1177,29
1865,63	2137,80	1255,34	2137,80	4918,75	20,07	4,92	2,53	9,74	2,39
8,63	91,37	10756,94		3,55	98,95	0,93	98,03	27,61	11,97
x	x				181,35	0,48	0,00	25,35	63,89
20,00	11,97	30,66	14,82	6,87	28,35	28,75	6,29	21,39	21,21
143,47	79,56	55,43	35,45	0,39	0,45	x	x	x	x
1207,97	1973,15	301,77	644,91	985,84	233,35	326,85	845,33	398,06	364,04
701,90	321,33	4990,24	2477,91	x	x	x	x		
0,57	0,00	181,35	0,00	215,95	x	x	x	x	x
x	11,97	10,58		2,73	43,31	18,03	27,65	24,42	6,30
41,63	0,17	12,76	1,01	0,09	32,14	7,94	0,90	13,80	40,38
8,99	2146,80	5,66	0,58	0,00	2146,80	5,66	8,99	0,00	8,45
0,00	10,06	0,42	x	x					
WPRO	Mongolia		Asia	Eastern Asia				1295470,00	
1146161,00		25,32	21,58	53,11	290207,97		247341,54		
608726,11		52,00	284,00						52,00
47272,73		86666,67		66969,70		155080,00		914,00	1035,00
199,89	19258,30		11930,08		81,99	5744,11	1567,73	117,90	13514,19
10362,35		3,29	1,40	13137,00		1146161,00		52,00	120,41
4,54	914,00	79,74	66969,70		50,94	26,73	6133,92	24433,88	
82634,26		36587,84		12664,90		36587,84		113202,06	
67155,65		43232,70		67155,65		9876,63	1297,49	9,88	2,67
947,13	7,21	7,45	92,55	11461,74		67,21	1947,49	17,49	1930,00
28,72	361,78	10388,84		94,15	10482,98		0,91	0,12	7598,15
0,66	0,09	477,88	1328,62	1145,84	389,74	1163,94	712,69	156,77	546,58
1249,84	248,52	863,60	785,89	3979,68	2494,05	1893,52	1211,10	0,48	0,49
1893,52	1211,10	272,67	81,14	353,81	22784,39		40603,24		16197,33
19234,31		37296,13		10316,53		7593,12	16096,23		16130,55
12909,10		28197,26		10897,66		128756,02		73805,11	
61261,58		35839,52		8821,67	2401,25	11222,92		0,98	0,50
10482,98		7598,15	11222,92		5744,11	32107,49		9,26	6,71
9,91	5,07	69,04	361,78	319,61	353,81	120,41	1000,22	198,41	36,17
31,95	12,04	19,84	0,17	427,12	24,96	2,82	1031,84	329,32	41,60
453,02	1375,03	303,46	67459,10		5,89	0,58	6486,84	73945,95	
6,45	6790,30	14,18	10,28	15,18	7,77	9,18	43,42	100,00	
WPRO	Nauru	Oceania	Micronesia				2883,00	2883,00	
	0,00	0,00	0,00					0,00	0,00
0,00	0,00	0,00	0,00	0,00	0,00	1,93	1,93	0,80	60,37
27,43	0,31	18,52	1,69	0,49	41,85	25,74	1,60	1,40	118,00
2883,00	0,00	0,00	0,00	1,93	66,95	0,00	53,75	32,84	0,00
63,39	101,30	88,95	25,74	88,95	164,69	152,34	89,13	152,34	5712,56
6,74	5,71	2,20	4,15	3,51	0,00	100,00	40929,59		0,19
6,20	0,05	6,15	32,37	0,91	29,46	0,24	29,69		
17,29	0,60	0,00	3,40	4,29	0,96	1,52	1,74	0,60	1,06
2,28	0,99	1,08	1,59	0,67	11,47	6,56	4,21	2,70	0,37
0,41	x	x	x	x		164,69	137,01	14,04	82,50
58,42	9,11	51,24	69,19	15,63	60,60	53,74	11,62	450,33	243,85
x	x	x	x				29,69	17,29	0,00
18,52	x	x	x	x	x	x	0,91	0,80	
0,00	2,73	1,02	x	29,40	x	x	0,17	1,77	0,07
0,00	4,57	0,65	0,04	1,84	5,23	1,17	153,52	5,32	0,58

153,52	5,32	1,17	19,34	11,26	0,00	12,07	0,76	x	x
WPRO	New Caledonia		Oceania Malenesia					115876,80	
117190,00		1,87	22,87	75,26	2191,45	26801,35		88197,19	
			0,39	2,87	6,09	9,35	8496,02	15576,04	
12036,03		12036,00		93,00	103,00				
					1,40	2682,00	117190,00		9,35
9,35	7,97	93,00	79,36	12036,03				0,00	0,00
		0,00	0,00	0,00	0,00	0,00	0,00	0,00	0,00
0,00	0,00	0,00			22885,91		x	x	x
x		36,99	0,00						
x	x	x	x	x	x	x	x	x	x
x	x	x	x	169,37	108,33			x	x
x	x		x	x	x	x	x	x	x
x	x	x	x	x	x	x	x	x	x
x				0,00	0,00	0,00	0,00	x	x
x	x	x	x	36,99	32,68		9,35	135,02	56,01
27,40	24,20	6,92	41,48	0,17					
0,00	0,00	0,00	0,00	0,00	0,58		0,00	0,00	0,00
x	x	x	x	x	x	x			
WPRO	Niue	Oceania Polynesia					663,00	663,00	0,00
0,00	100,00	0,00	0,00	663,00					0,00
0,00	0,05	0,05	41,59	76,25	58,92	59,00	1,00	1,05	0,21
11,27	5,91	0,03	2,09	0,44	0,18	9,18	5,47	1,48	1,40
663,00	0,05	0,05	6,90	1,00	150,83	58,92	47,37	21,04	2,17
21,04	31,66	30,10	6,05	30,10	54,86	53,30	29,26	53,30	8275,17
0,00	8,28	3,50	0,00	4,78	5,00	95,00	0,00	0,07	1,61
0,02	1,60	22,86	0,21	4,78	0,06	4,84			3,25
0,49	0,00	0,23	1,16	1,10	0,09	0,47	0,66	0,07	0,45
1,41	0,06	0,28	0,83	2,54	1,61	0,97	0,62	0,38	0,39
x	x	x	x		10,83	34,97	15,06	5,22	15,27
9,53	3,30	13,19	17,56	3,55	9,73	12,08	74,49	39,65	x
x	x	x				4,84	3,25	0,00	2,09
x	x	x	x	x	x	0,21	0,18		0,05
1,23	0,79	17,01	15,02	3,72	64,25	0,17	0,18	0,03	0,00
0,56	0,23	0,06	0,21	0,81	0,17	53,47	8,07	0,58	
53,47	8,07	0,17	9,06	6,08	0,00	3,92	0,32	x	x
WPRO	Norfolk Island		Oceania	Australia and New Zealand					
978,00	978,00	6,00	14,00	80,00	58,68	136,92	782,40		
	0,01	0,01	0,05	0,08	71,95	131,90	101,92	102,00	1,00
1,08									
1,40		978,00	0,08	0,08	8,09	1,00	102,25	101,92	
0,00	0,00				0,00	0,00	0,00	0,00	0,00
0,00	0,00	0,00	0,00	0,00	0,00	x		0,00	x
x	x	x	x	0,31	x	x			
		x	x	x	x	x	x	x	x
x	x	x	x	x	x	1,43	0,91		
x	x	x	x		x	x	x	x	x
x	x	x	x	x	x	x	x	x	x
x	x	x				0,00	0,00	0,00	0,00
x	x	x	x	x	x	0,31	0,27		0,08
1,35	0,69	22,84	20,17	5,85	51,14	0,17			
		0,00	0,00	0,00	0,00	0,00	0,58		0,00
0,00	0,00	x	x	x	x	x	x	x	
WPRO	Northern Mariana Islands				Oceania Micronesia				
27970,00		27970,00		1,90	10,00	88,10	531,43	2797,00	24641,57
				0,10	0,30	1,70	2,09	1904,25	3491,12

2697,69	2698,00	22,00	24,09	4,77	302,87	161,47	0,97	58,56	13,45
3,80	244,31	148,02	1,54	1,40	1182,00	27970,00		2,09	2,09
7,49	22,00	78,66	2697,69	46,64	25,32	97,70	557,12	885,64	810,86
177,15	810,86	1540,46	1465,68	831,97	1465,68	5507,56	65,10	5,51	2,34
37,43	3,17	8,23	91,77	42259,56		2,06	56,73	0,54	56,19
27,28	8,83	240,81	2,31	243,13	0,00		92,01	0,33	0,00
5,18	28,02	10,57	2,44	13,38	7,94	1,07	9,72	9,88	1,03
6,99	6,94	55,19	24,42	40,88	26,15	0,74	1,07	40,88	26,15
5,89	1,75	7,64	234,72	853,05	157,39	131,11	441,69	123,38	50,68
295,61	132,97	66,21	259,61	108,51	1754,15	752,60	1299,40	805,90	187,11
54,00	241,11	0,86	0,21	243,13	92,01	241,11	58,56	830,87	15,78
5,97	15,65	3,80	58,79	8,83	7,80	7,64	2,09	24,26	5,53
36,40	32,16	8,64	22,81	0,17	5,49	0,84	0,07	10,12	6,02
0,48	6,35	16,30	3,76	1469,44	5,25	0,58	139,36	1608,80	5,75
143,12	15,11	5,72	14,99	3,64	8,90	51,65	100,00		
WPRO	Palau	Oceania	Micronesia				10470,00		11610,00
20,00	10,00	70,00	2322,00	1161,00	8127,00				
0,42	0,12	0,56	1,10	1000,57	1834,38	1417,48	1417,00	9,00	10,10
3,27	172,68	78,51	0,54	30,51	5,05	2,73	142,17	73,46	1,07
1,40	268,00	11610,00		1,10	1,10	9,48	9,00	77,52	1417,48
47,04	25,14	51,77	226,28	252,18	327,78	83,73	327,78	530,23	605,83
361,78	605,83	4566,97	12,24	4,57	2,39	5,82	2,17	11,70	88,30
23083,55		0,81	23,30	0,21	23,09	28,51	3,66	104,46	0,95
105,41	0,00		45,13	0,39	0,00	5,67	14,68	6,94	2,30
7,68	4,13	1,00	5,81	6,72	0,65	2,72	3,24	34,01	13,49
15,30	9,79	x	x	x	x	x	x		264,46
454,29	106,91	122,57	248,57	68,69	46,39	173,45	96,84	38,92	99,37
55,21	1148,42	x	x	x	x	x			
105,41	45,13	0,00	30,51	x	x	x	x	x	x
3,66	3,24		1,10	13,34	5,34	27,47	24,27	8,25	40,00
0,17	2,43	0,35	0,04	5,78	2,80	0,33	2,78	8,68	1,90
607,73	5,23	0,58	0,00	607,73	5,23	1,90	17,35	7,43	0,00
5,02	0,31	x	x						
WPRO	Papua New Guinea			Oceania	Malenesia				
3378679,00		2598557,00		56,15	13,23	30,63	1459089,76		
343789,09		795938,01						261,18	36,79
54,92	352,88	320802,02		588137,04		454469,53		454470,00	
2073,00	2425,88	1261,19	78054,37		31941,74		513,86	32703,49	
5424,82	747,33	45350,87		26516,92		1,41	1,40	23591,00	
2598557,00		352,88	352,88	13,58	2073,00	79,78	454469,53		53,09
25,20	18732,95		52243,25		77280,29		77032,39		30242,28
77032,39		148256,48		148008,58		101218,48		148008,58	
5705,34	1345,95	5,71	2,73	701,59	2,97	15,15	84,85	9078,50	101,22
2862,10	26,57	2835,53	28,01	820,22	22977,25		215,31	23192,56	
0,89	0,21	18116,23		0,70	0,16	1131,42	2489,86	1159,75	682,60
1465,12	1051,06	350,69	989,69	962,39	394,87	804,57	815,85	6505,93	3132,58
4938,43	3158,65	0,76	1,01	4938,43	3158,65	711,13	211,63	922,76	54094,01
77007,19		18150,38		37855,21		47724,29		16954,00	
16876,95		29979,39		14063,87		24293,03		27846,03	
13031,17		228382,16		108027,08		173357,07		108926,09	
24963,42		7298,05	32261,47		1,24	1,26	23192,56		18116,23
32261,47		32703,49		41734,84		15,64	12,22	21,76	22,06
28,32	820,22	724,62	922,76	352,88	2227,74	330,02	36,82	32,53	15,84
14,81	0,17	1310,75	70,39	8,17	4013,40	595,52	74,64	1383,86	4633,80
998,93	149007,52		5,73	0,58	18647,13		167654,64		6,45
19646,06		13,83	10,81	19,24	19,51	11,72	24,89	100,00	

WPRO Philippines Asia South-Eastern Asia
42664760,00 42428367,00 22,86 19,12 58,03 9699124,70
8112303,77 24621181,37 1736,14 868,02
1698,86 4303,02 3911837,58 7171702,23 5541769,91 5541770,00
33848,00 38151,02 10244,87 833884,46 470520,63
4166,90 285779,95 74106,71 6077,97 548104,50 396413,93
2,61 1,40 361489,00 42428367,00 4303,02 4303,02 10,14 33848,00
79,78 5541769,91 50,80 24,96 218587,82 844759,47
2284142,63 1261882,73 472941,42 1261882,73 3347489,92
2325230,02 1536288,71 2325230,02 7889,74 28520,56 7,89
2,51 19460,86 5,38 8,82 91,18 8519,98 1369,54 41340,18
393,09 40947,10 29,90 13392,27 400407,94 3843,90
404251,84 0,95 3,44 247338,66 0,58 2,11 10904,78
25589,33 20492,84 5977,52 18804,48 14119,33 3642,13
11456,07 25326,98 3091,74 10885,80 19310,53 72813,51
43954,91 62360,70 39886,29 0,86 0,91 62360,70
39886,29 8979,94 2672,38 11652,32 543364,93 769785,53
295018,28 321826,27 604505,52 223316,29 183582,50
336254,76 323778,70 191536,19 376748,36 293278,34
2412260,44 1293807,49 2065966,19 1174048,22 297499,13
78661,23 376160,36 0,89 0,67 404251,84 247338,66
376160,36 285779,95 1011699,21 12,08 7,39 11,24 8,54
60,76 13392,27 11831,41 11652,32 4303,02 38330,11
8803,41 34,94 30,87 11,23 22,97 0,17 13300,85 1774,49 254,84
22579,32 4184,09 663,43 15160,29 26984,55 6996,04
2332226,07 5,50 0,58 217420,69 2549646,76 6,01
224416,73 15,86 9,70 14,75 11,21 8,80 39,68 100,00
WPRO Samoa Oceania Polynesia 71941,62 41142,00
30,21 24,89 44,91 12429,00 10240,24 18476,87
2,22 1,10 1,27 4,60 4177,64 7659,00 5918,32 5918,00
33,00 37,00 16,15 1092,46 573,98 5,26 323,58 51,04 10,89 768,88
522,94 2,13 1,40 807,00 41142,00 4,60 4,60 11,17 33,00
80,21 5918,32 51,80 22,58 238,05 745,12 1628,91 1090,12 567,52 1090,12
2612,07 2073,29 1550,68 2073,29 6348,92 51,24 6,35 2,39 31,95 3,96
10,82 89,18 19614,99 1,78 46,87 0,53 46,34 26,03 12,99
338,08 3,87 341,95 0,83 0,01 375,49 0,91 0,01 24,83 72,19
60,25 12,64 33,84 41,15 10,09 40,33 78,28 10,03 27,38 51,73
177,30 131,17 105,15 67,26 0,59 0,51 105,15 67,26 15,14 4,51
19,65 1187,99 2205,18 821,78 704,16 1117,48 595,64 482,62 1193,57 1004,69
591,38 932,28 742,57 5687,28 3782,28 3372,94 1939,39 485,70 129,94 615,64
1,50 0,79 341,95 375,49 615,64 323,58 416,63 13,09 14,38 23,57
12,39 36,58 12,99 11,47 19,65 4,60 29,42 0,37 44,14 39,00
15,62 1,24 0,17 26,08 1,66 0,27 45,74 7,96 1,36 27,83
54,16 13,61 2086,90 5,07 0,58 355,84 2442,74 5,94 369,45 14,00
15,37 25,20 13,25 15,12 17,06 100,00
WPRO Solomon Islands Oceania Malenesia 202500,00
334194,00 37,26 12,13 50,61 124520,68 40537,73
169135,58 22,29 4,34 11,67 38,30
34815,54 63828,49 49322,02 49322,00 267,00 305,30
95,40 6053,13 2165,41 44,25 2576,41 219,84 51,15 3476,73 1945,58 1,27
1,40 1551,00 334194,00 38,30 38,30 11,46 267,00 79,89 49322,02
53,25 29,94 2039,50 7993,24 10346,96 11406,26 2135,83 11406,26
20379,70 21439,00 12168,57 21439,00 6098,16 94,58
6,10 3,00 48,02 3,10 10,94 89,06 4641,02 14,81 464,76 4,10
460,66 31,10 105,49 3281,12 29,20 3310,32 0,99 0,02 1421,85 0,43
0,01 240,36 406,85 160,12 99,95 197,65 113,46 112,47 202,78 120,12

76,27	163,64	137,76	1036,00	641,11	295,98	189,31	0,29	0,30	295,98
189,31	42,62	12,68	55,31	11482,50		12557,97		2510,47	4997,56
6268,81	1815,22	5458,87	6167,99	1890,72	4027,00	5172,99	2210,39	36748,74	
22193,90		10498,99		6553,62	1511,85	439,09	1950,95	0,58	0,77
3310,32	1421,85	1950,95	2576,41	12179,47		16,24	6,98	9,57	12,64
54,56	105,49	93,19	55,31	38,30	343,18	106,21	30,74	27,16	11,16
30,95	0,17	53,63	4,22	0,50	241,67	41,14	4,85	58,01	284,43
56,85	21495,85		6,43	0,58	1127,65	22623,49		6,77	1184,49
14,63	6,28	8,62	11,39	5,24	53,84	100,00			
WPRO	Tokelau	Oceania	Polynesia				440,00	1100,00	
		0,00	0,00					0,00	0,00
0,00	0,00	0,00	0,00	0,00	0,00	1,00	1,00	0,13	8,24
4,38	0,03	2,20	0,48	0,10	6,04	3,90	1,83	1,40	
1100,00	0,00	0,00	0,00	1,00	90,91	0,00	49,93	21,66	0,00
21,66	39,62	30,40	3,90	30,40	61,29	52,06	25,57	52,06	5571,74
0,00	5,57	1,97	0,00	3,60	0,00	x	0,00	0,03	0,80
0,01	0,79	26,33	0,35	9,14	0,12	9,26			2,38
0,22		0,13	0,47	0,49	0,06	0,21	0,31	0,09	0,42
0,71	0,08	0,29	0,45	1,14	1,26	0,64	0,41	x	x
x	x	x	x		6,35	13,72	6,44	3,36	6,87
4,40	4,08	12,09	8,77	4,38	9,22	6,25	33,91	34,79	x
x	x	x			x	9,26	2,38	0,00	2,20
x	x	x	x	x	x	0,35	0,31		0,00
1,31	0,65	26,57	23,47	0,00	49,96	0,17	0,17	0,02	0,00
0,48	0,15	0,02	0,19	0,64	0,14	52,20	4,75	0,58	
52,20	4,75	0,14	17,74	4,56	0,00	4,22	0,26	x	x
WPRO	Tonga	Oceania	Polynesia				28598,00		28598,00
19,37	30,90	49,80	5539,43	8836,78	14241,80				
0,99	0,95	0,98	2,92	2654,34	4866,30	3760,32	3758,00	23,00	25,92
8,23	518,07	283,49	2,11	137,69	27,99	6,12	380,39	255,50	2,05
1,40	512,00	28598,00		2,92	2,92	10,21	23,00	80,43	3760,32
51,90	20,40	151,44	469,30	998,76	697,16	294,15	697,16	1619,49	1317,90
914,88	1317,90	5662,97	28,99	5,66	2,17	17,88	3,49	11,74	88,26
17903,35		2,11	48,70	0,49	48,21	22,85	9,03	206,25	2,10
208,34	0,00		174,50	0,61	0,00	7,80	25,69	30,97	2,12
8,17	15,34	1,62	7,39	26,86	1,96	6,48	23,50	59,22	34,24
41,80	26,74	x	x	x	x	x	x		373,77
782,63	433,86	133,39	268,86	234,78	80,26	222,75	324,83	135,83	235,47
322,83	1781,53	890,20	x	x	x	x			0,48
208,34	174,50	0,00	137,69	x	x	x	x	x	x
9,03	7,97		2,92	33,89	13,97	26,63	23,53	8,61	41,23
0,17	13,16	0,62	0,14	30,79	5,01	1,19	13,82	36,20	8,30
1326,20	4,64	0,58		1326,20	4,64	8,30	15,71	13,16	0,00
10,38	0,63	x	x						
WPRO	Tuvalu	Oceania	Polynesia				3615,00	3615,00	68,00
6,00	26,00	2458,20	216,90	939,90					0,44
0,02	0,06	0,53	480,07	880,13	680,10	0,00	3,00	3,53	1,49
85,33	37,98	0,43	25,03	3,02	1,06	60,30	34,96	1,38	1,40
49,00	3615,00	0,53	0,00	14,61	3,00	82,99	680,10	50,76	23,94
0,00	71,81	99,06	100,77	34,96	100,77	170,88	172,58	106,77	172,58
4726,85	2,32	4,73	1,99	1,34	2,74	0,00	x	13554,63	
0,32	8,44	0,08	8,36	26,13	1,14	29,81	0,29	30,10	0,00
22,09	0,61		2,40	6,05	4,29	1,13	2,79	2,87	0,71
3,50	5,72	0,70	2,49	3,92	14,76	10,62	5,28	3,38	x
x	x	x	x	x		115,39	182,88	61,67	61,46
88,58	43,20	34,04	101,45	79,52	39,62	79,92	59,98	483,27	301,53

x   x   x   x   x   30,10   22,09   0,00
25,03   x   x   x   x   x   x   1,14   1,01
0,00   4,01   1,86   28,47   25,15   0,00   46,38   0,17   1,84   0,13
0,02   3,96   1,20   0,21   1,98   5,23   1,20   173,78   4,81   0,58
173,78   4,81   1,20   17,32   12,71   0,00   14,40   0,69   x   x
WPRO   Vanuatu Oceania Malenesia   117678,10
126321,00   56,78   14,08   29,14   71725,06   17786,00   36809,94
                                    12,84   1,90   2,54   17,28   15710,70
28802,96   22256,83   22257,00   101,00   118,28   48,36   3130,56
1495,54 15,41   906,58   109,50   32,95   2223,98 1386,03 1,65   1,40   855,00
126321,00   17,28   17,28   13,68   101,00   79,96   22256,83   51,73
25,43   893,96   2568,14 4370,71 3726,39 1508,85 3726,39 7832,81 7188,49 4970,95
7188,49 6200,72 53,02   6,20   2,74   29,58   3,46   12,98   87,02   6768,47
6,98   186,53   1,77   184,77   26,47   39,87   1055,48 10,11   1065,59 0,84
0,01   1074,47 0,85   0,01   65,87   139,31   96,78   34,78   66,56   66,24
17,36   59,65   81,16   20,91   45,43   57,32   360,86   189,51   172,00   110,01
0,48   0,58   172,00   110,01   24,77   7,37   32,14   3179,85 4206,01 1472,61
1882,53 2125,17 1053,65 836,09   1743,98 1191,32 1206,76 1510,40 932,07   12235,65
6005,02 5832,05 3486,01 839,82   233,56   1073,38 0,85   0,72   1065,59 1074,47
1073,38 906,58   3068,47 13,60   13,72   13,70   11,57   47,40   39,87   35,23
32,14   17,28   121,37   28,99   32,85   29,02   14,24   23,88   0,17   46,37
2,13   0,40   123,47   18,27   3,26   48,64   142,82   31,78   7220,28 5,72
0,58   620,41   7840,69 6,21   652,20   13,59   13,70   13,69   11,56   8,32
39,14   100,00
WPRO   Vietnam Asia   South-Eastern Asia   54995350,00
50567891,00   37,22   27,44   35,34   18821369,03   13875829,29
17870692,68   3369,03 1484,71 1233,08 6086,82
5533469,62   10144694,31   7839081,97   7839082,00   40342,00
46428,82   13320,31   949547,05   537592,17   4122,30
261336,92   56028,27   9198,01 688210,13   481563,90   2,33
1,40   271158,00   50567891,00   6086,82 6086,82 12,04   40342,00
79,78   7839081,97   49,80   22,47   303150,51   906339,39
2194842,87   1354492,45   564293,00   1354492,45   3404332,77
2563982,35   1773782,90   2563982,35   6732,20 18254,91   6,73
2,39   11769,31   4,34   11,33   88,67   5362,26 3323,05 95490,59
939,83   94550,76   28,45   15961,47   454151,68   4514,25
458665,93   0,91   2,46   306585,43   0,61   1,64   3368,78 17541,43
22901,00   6849,47 34665,35   36674,87   1061,60 6708,42 23058,83
1803,41 11447,84   44452,07   82283,66   43524,90   80383,63
51413,87   0,98   1,18   80383,63   51413,87   11575,24
3444,73 15019,97   162533,34   520698,38   312362,87
352525,35   1090778,43   531290,31   51735,06   199517,37
265631,00   119111,89   391524,00   577835,57   2407753,23
1043043,84   2352155,54   1232097,51   338710,40   82550,53
421260,93   0,83   0,52   458665,93   306585,43   421260,93
261336,92   1116133,14   13,47   9,01   12,37   7,68   57,47   15961,47
14101,16   15019,97   6086,82 45510,01   9360,56 35,07   30,98
13,37   20,57   0,17   6410,71 1035,05 143,87   16833,67   5628,55 807,94
7493,72 22731,54   5017,39 2568999,74   5,08   0,58   243488,82
2812488,56   5,56   248506,21   16,31   10,90   14,98   9,29   8,84
39,68   100,00
WPRO   Wallis and Futuna   Oceania Polynesia   3104,00
4482,00 80,00   4,00   16,00   3585,60 179,28   717,12
0,64   0,02   0,05   0,71   645,90   1184,14 915,02   915,00   4,00   4,71
                                                                               1,40
4482,00 0,71   0,71   15,85   4,00   89,25   915,02   0,00

0,00 x 0,00 0,00 0,00 0,00
0,00 0,00 0,00 0,00 0,00 0,00 x x
x x x 1,41 x x
2504,93 10125,48 22508,31 4968,55 19724,13 34819,57
911,47 5423,07 32052,44 1292,21 7552,49 53067,48 56432,38
43552,55 4,54 2,90 0,00 0,00 4,54 2,90 0,65 0,19
0,85 x x x x x x x x x
x x x x x x x x x x
x 0,00 0,00 x 0,00 x x x x
x x 1,41 1,25 0,71 5,96 2,59 23,74 20,97
11,92 43,37 0,17
x x x x x
x x x x x
WPRO TOTAL 953688321,20
895618926,00 230699448,18 240491297,21
424430409,85 52,00 284,00 0,00 0,00 41295,20 25732,57
29243,70 96245,05 87495503,12 160408422,39 123951962,76
124039391,00 714503,93 810748,98 372504,45 17870000,41
8866067,09 73422,05 4731229,56 1194524,80 299082,39
13138770,85 7671542,28 1,40 1,40 16198581,00 895618926,00
96245,05 96312,94 10,75 714503,93 79,78 123951962,76
48,17 18,28 4615840,05 13061137,61 19886217,87 20204139,73
9230545,88 20204139,73 37563195,53 37881117,39 26907523,54
37881117,39 4194,10 679385,45 4,19 1,97 359671,40 2,22
73211,14 282697,00
7032340,52 0,79 127,19 3597941,10 0,40 65,07

1540473,52 1007954,58 1393953,00 891581,00 0,90 0,88
1393953,00 891581,00 200729,23 59735,93 260465,16
4390706,28 10395472,42 9954906,28 4213647,62 13447167,69
13251495,63 1332002,48 5068785,66 9151675,82 1923097,45
7939976,75 11534086,63 40182461,31 23159116,49 36360548,62
20485276,44 5235919,00 1372513,52 6608432,52 0,74 0,53
7032340,52 3597941,10 6829424,76 4731229,56 15914234,90
282697,00 249748,80 260465,16
96312,94 800100,51 171341,77 35,33 31,21 12,04 21,42
0,17 97757,17 24782,30 6488,44 334084,50 205329,61
39566,56 124702,28 552602,96 112432,67 37993550,06
4,24 0,58 3819674,00 41813224,06 4,67 3708053,23 16,82
8,60 16,33 11,32 8,87 38,06 100,00
ALL 3499926316,70 3096487323,10
800259413,82 666426250,02 1459400626,88 11237,00
5590901,00 3775,00 3463829,00 143246,44 71307,61
100698,64 315038,54 286398674,72 525064236,99 546421807,87
124039391,00 2596016,69 2911055,51 1219963,97 66072948,36
33158271,85 311289,66 22314227,55 6837934,55 908660,47
43758137,93 26328196,78 1,51 1,51 84815577,00 3096487323,10
315038,54 313773,12 10,17 2596016,69 83,84 405708101,32
49,72 19,18 15610782,94 51992918,50 98961244,56 96658768,63
32941955,69 96658768,63 166564946,00 164164184,83 100545657,13
164262470,07 5379,16 3437463,31 5,38 2,18 2710637,63 3,20
842409,62
19944466,38 0,64 546,30 15217824,54 0,49 416,83

5155916,03 3325951,80 4675587,43 2990537,78 0,91 0,90
4675587,43 2990537,78 673284,59 200366,03 873650,62

21955514,19 51305355,66 37294097,29 12731159,60 34149453,99
29052661,05 8602448,55 25724767,93 36978768,66 8708444,60
24716754,13 32111135,19 142191317,01 90782383,16 128944678,39
81627204,23 18568033,69 5469022,68 24037056,37 0,78 0,72
19927530,17 15140382,14 25482895,94 22269879,90 82645280,99
842409,62 912722,71 873650,62
313773,12 2948861,89 879956,44 28,57 30,95 10,64 29,84
0,17 1092266,92 184611,87 44761,32 3510821,53
1198763,80 244340,83 1291799,23 4791032,27 1009750,03
165272220,10 5,34 0,58 13893418,58 179165638,69 5,79
13699669,54 11,12 8,45 14,22 12,43 7,65 46,13 100,00
ALL* 3096487323,10

2596016,69
84815577,00
3096487323,10 2596016,69
15610782,94 51992918,50 98961244,56 96658768,63 32941955,69
96658768,63 166564946,00 164164184,83 100545657,13 164262470,07
5379,16 4562363,91 5,38 2,18 1433548,53 3,20
0,64
546,30 416,83

954722,16 21955514,19 51305355,66
37294097,29 12731159,60 34149453,99 29052661,05 8602448,55
25724767,93 36978768,66 8708444,60 24716754,13 32111135,19
142191317,01 90738539,54 135452033,56 113219090,56 19505092,83
7585679,07 26991200,84 x

0,17 1092266,92 184611,87 44761,32
3510821,53 1198763,80 244340,83 1291799,23 4791032,27
1009750,03 165272220,10 5,34 0,58 15600914,08 180873134,19
5,84 15407165,04 8,52
